# Evolution of the essential gene *MN1* during the macroevolutionary transition toward patterning the vertebrate hindbrain

**DOI:** 10.1073/pnas.2416061122

**Published:** 2025-05-27

**Authors:** Elio Escamilla-Vega, Louk W. G. Seton, Stella Kyomen, Andrea P. Murillo-Rincón, Julian Petersen, Diethard Tautz, Markéta Kaucká

**Affiliations:** ^a^Max Planck Institute for Evolutionary Biology, Plön 24306, Germany; ^b^Department of Orthodontics, University Leipzig Medical Center, Leipzig 04103, Germany

**Keywords:** *MN1*, gene evolution, hindbrain patterning, retinoic acid, co-evolution and co-development of brain and skull

## Abstract

The tight link between brain and skull formation is a fundamental aspect of vertebrate evolution and embryogenesis. Their developmental synchronization is essential for structural and functional integration. The brain and skull shape coevolution is evident along the vertebrate phylogeny; however, the genetic basis underlying their close evolutionary and developmental relationship remains little explored. Here, we reveal the evolution and function of the *MN1* gene that was previously found to be associated with significant shape variation in the mouse skull and the formation of cranial bones. We show that the vertebrate *MN1* gene evolved from an ancestral deuterostome sequence. In vertebrates, the *MN1* gene structure, synteny, and spatiotemporal expression pattern are remarkably conserved, indicating that the gene carries out a core function. Using a newly generated mouse knock-out model, we demonstrate in vivo that *Mn1* integrated into an ancient molecular machinery and controls the expression of the *Cyp26* genes in the developing hindbrain, thereby tuning the retinoic acid levels and patterning of the developing central nervous system. This study thus showcases the emergence of a novel gene function from an ancestral sequence and its role in generating a macroevolutionary innovation. The data expand our knowledge of brain and skull codevelopment and coevolution and highlight the role of this regulatory loop in craniofacial human syndromes.

## Significance

The origin of the complex brain and skull in vertebrates is a major evolutionary innovation. The emergence of novel traits is driven by genome duplications and regulatory changes; however, the role of novel genes in this process remains less understood. Here, we investigate the emergence of *MN1*, a gene previously associated with cranial bone formation. We show that *MN1* emerged from an ancestral deuterostome sequence and, upon structural evolution, was incorporated into the ancient molecular program driving vertebrate embryonic development. *MN1* integrated into the retinoic acid/*Hox* signaling machinery controlling patterning of the hindbrain. These findings demonstrate how novel genes incorporate into existing pathways and fuel macroevolutionary transitions. Understanding *MN1* function in development additionally sheds light on several neurodevelopmental and craniofacial syndromes.

Morphological innovations are fundamental drivers of evolution and speciation. Several major novelties appeared at the origin of vertebrates and enabled an array of changes, such as the shift to a predatory lifestyle, water-to-land transition, and adaptation to diverse environments across the entire planet. Among the key vertebrate innovations is a complex brain protected by a bony skull. These two structures exhibit close developmental and evolutionary relationships. Both arise during embryogenesis, and their formation and growth are tightly coordinated to achieve proper integration and functionality. Disruption of the synchronized development results in an array of pathological conditions, such as microcephaly, craniosynostosis, and holoprosencephaly, manifesting both in the nervous system and the skull (1). From an evolutionary perspective, the shape and size covariation of the brain and skull is evident along the vertebrate phylogeny, suggesting the existence of a coevolving genetic basis (2, 3).

The origin of evolutionary novelties is often underpinned by a complex set of changes. The genetic underpinnings comprise, for instance, gene or genome duplication, mutations, deletions, and *cis*-regulatory evolution, which jointly form new or alter the existing molecular machineries (4). Such genetic changes lead to developmental alterations, which ultimately manifest in novel phenotypes or adaptations of existing traits. Different selection pressures act upon the individual changes and their manifestation during development and determine whether genetic changes will persist in populations, with most conserved changes frequently being part of core developmental programs. However, an additional and rather unexplored mechanism is the acquisition of novel genes in this process.

Novel genes evolve either from ancient coding sequences by mechanisms such as fast divergence after gene or genome duplication events, gene fusion and fission, horizontal gene transfer, retroposition, and exonification, where new exons arise typically as a consequence of intronic mutations, (5, 6) or arise from noncoding sequences by de novo gene birth (7). New genes can complement the existing genetic networks, rewire them, or form new molecular machineries, consequently driving the origin of new traits or lineage-specific adaptations (8–10). Hence, investigating the trends of gene evolution allows to uncover the genomic history of major adaptations.

Here, we investigate the origin and function of Meningioma-1 (*MN1*), a gene that was initially studied due to its implication in meningioma and acute myeloid leukemia (11, 12). To simulate its oncogenic potential, a mouse knock-out model with a targeted disruption of *Mn1* was generated; however, the mutants exhibited severe defects of intramembranous cranial bone formation (13, 14). Interestingly, a later study addressing the genetic basis of craniofacial shape variation found that the *Mn1* gene had an unusually large effect on skull shape in a population of outbred mice and suggested that *Mn1* originated at the base of bony vertebrates (Euteleostomi) (15). These observations jointly indicated that *Mn1* might represent a crucial link to the evolutionary innovation — the bony skull. However, intramembranous ossification, a bone-forming mechanism that generates the vertebrate neurocranium, evolved prior to the origin of Euteleostomi (16). Here, we revisit the origin of *MN1* and set out to understand its function in the development and evolution of the brain and the skull.

## Results

### *MN1* Evolved From an Ancestral Deuterostome Sequence.

**Fig. 1.**
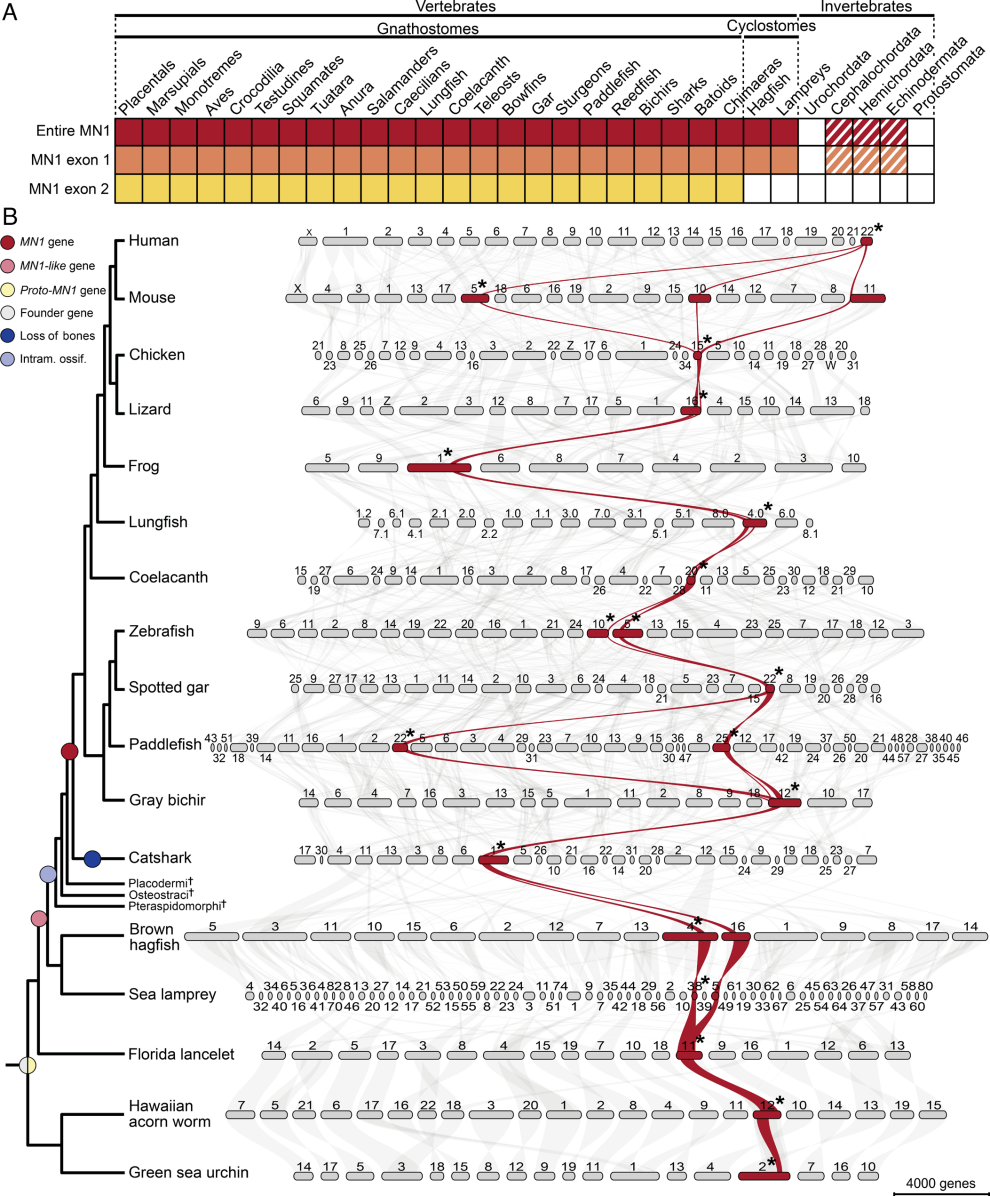
Assessment of the evolutionary origin of the *MN1* gene. (*A*) Summary of *MN1* genes identified in Bilateria. Colored boxes indicate the presence of homologous *MN1* genes, white boxes the absence of *MN1,* and dashed-colored boxes presumptive distant homologs. (*B*) Group-scale synteny analysis. Dendrogram indicating the relative evolutionary relationships between species and events during deuterostome evolution (*Left*). Chromosomal-level synteny analysis of representative species covering major deuterostome lineages (*Right*). The human *MN1* genomic region spanning 50 upstream and downstream genes was highlighted in red. Asterisks denote the chromosomes in which *MN1* homologs are located. Chromosomes are scaled by gene rank order.

Due to the essential role of *Mn1* in intramembranous ossification, the bone-forming mechanism that existed already in Ordovician agnathans (13, 16, 17), we reassessed the *MN1* evolutionary origin using refined methods and a broader repertoire of available genomes. BLASTp and HMMER search retrieved homologs of the human *MN1* gene from all gnathostome lineages, including previously unreported homologs in Chondrichthyes, cartilaginous fish (Fig. 1*A* and *SI Appendix*, Table S1). In cyclostomes, the only extant lineage of jawless fish, *MN1-like* genes that are homologous to the human *MN1* exon 1 were also identified in both lampreys and hagfish (Fig. 1*A* and *SI Appendix*, Table S1). In the search for the ancestral sequence, we screened for amino acid sequence conservation across invertebrate bilaterians and recovered 14 hits from cephalochordates, hemichordates, and echinoderms (Echinoidea and Asteroidea) (Fig. 1*A* and *SI Appendix*, Table S1). These invertebrate hits (hereinafter referred to as proto-MN1) match to less than 10% of the human MN1 protein corresponding to exon 1 (*SI Appendix*, Table S2). Subsequently, we performed a phylogenetic analysis to reconstruct the evolutionary relationships between the invertebrate proto-MN1 and the vertebrate MN1 sequences (*SI Appendix*, Fig. S1). The inferred phylogenetic tree largely reflects the known phylogenetic relationships, with all vertebrate MN1 sequences grouped together as a clade with high bootstrap support and the 14 invertebrate proto-MN1 amino acid sequences clustered independently from vertebrates. Strict collinear synteny is rarely conserved between vertebrates (gnathostomes and cyclostomes) and invertebrate lineages due to the large evolutionary distance between these groups and the accumulation of intrachromosomal inversions and rearrangements (18–21). Hence, we performed a group-scale synteny analysis taking 100 genes surrounding the human *MN1* locus as the reference and mapped it across representative species of all major deuterostome lineages (Fig. 1*B*). The macrosynteny analysis successfully recapitulated the chromosomal position of all *MN1* homologs in gnathostomes, cyclostomes, and invertebrate deuterostomes supporting the hypothesis of a shared ancestry regardless of their limited homology (Fig. 1*B* and *SI Appendix*, Table S1).

To further elucidate the evolutionary history of the identified *MN1* genes in gnathostomes, cyclostomes, and invertebrate deuterostomes, we searched for evidence of conserved gene structure (*SI Appendix*, Fig. S2). Across gnathostomes, *MN1* exhibits a conserved gene structure and comprises two coding exons separated by a large intron (*SI Appendix*, Fig. S2). The large exon 1 encodes for the vast majority of the protein. In contrast, the short exon 2 contributes to less than 10% of the final protein. Cyclostome *MN1-like* genes exhibit a similar gene structure, although possessing a much shorter exon 2 compared to gnathostomes (18bp in Atlantic hagfish, 73bp in sea lamprey, and 185bp in gnathostomes). Interestingly, the cyclostome *MN1-like* exon 2 is not homologous to the gnathostome *MN1* exon 2 (Fig. 1*A*). Given the distinct genome duplication events in gnathostomes and cyclostomes (22), inferring direct phylogenetic relationships (orthology and paralogy) between the *MN1* genes and the exact events that lead to the emergence of the gnathostome *MN1* exon 2 remain challenging. The invertebrate *proto-MN1* genes possess between 1-7 coding exons of varying length, all showcasing a large exon 1 that resembles the vertebrate *MN1* exon 1.

In summary, the performed analyses indicate that the ancestral *MN1* evolved in the common ancestor of deuterostomes. The gene exhibits distinct exon–intron structures across deuterostomes, indicating lineage-specific structural evolution of *MN1* at the onset of vertebrates and, additionally, in the common ancestor of gnathostomes. Notably, the structure and sequence of the *MN1* gene are remarkably conserved within gnathostomes, indicating that the gene was under strong purifying selection after its emergence in the current form. The general similarity of the *MN1* gene structure between cyclostomes and gnathostomes supports that the modern vertebrate *MN1* arose in their last shared ancestor. We further identified *proto-MN1* genes in invertebrate deuterostomes, with short regions homologous to the gnathostome *MN1* exon 1. The *proto-MN1* sequences indicate the presence of an ancestral founder gene of deuterostome origin (7, 23), rendering the *MN1* gene more ancient than initially assumed (15).

### In Silico Modeling of *MN1* Protein Structure.

To explore the structure and properties of the MN1 protein across deuterostomes and investigate whether the gnathostome-specific structural evolution of *MN1* may have impacted its functionality, we modeled the MN1 protein structure in silico. Previous in silico search for known DNA motifs or protein domains in the human MN1 protein identified two polyglutamine stretches and proline-rich regions but failed to recover any recognized functional domains or homology to any other known protein family (11, 12).

First, we searched for deeply conserved regions using the multiple sequence alignment of vertebrate and invertebrate MN1 amino acid sequences (*SI Appendix*, Fig. S3 and Dataset S1). This alignment uncovered a high degree of similarity in the conserved C-terminus domain in gnathostomes (*SI Appendix*, Fig. S3). Interestingly, the C-terminus region encoded by the short exon 2 is the only empirically demonstrated MN1 functional domain discovered in a human developmental disorder (24, 25). We detected six remarkably conserved regions encoded by the large exon 1 across vertebrates. Four of the conserved regions also align to the invertebrate proto-MN1 amino acid sequences and correspond to the above-detected sequence homology BLASTp hits (Fig. 1*A* and *SI Appendix*, Fig. S3).

Previous studies suggested that the human MN1 is an intrinsically disordered protein with a C-terminal ordered region (25). We applied a PONDR prediction tool (Prediction of Natural Disordered Regions) to vertebrate MN1 protein sequences and uncovered additional ordered domains beyond the C-terminus (*SI Appendix*, Fig. S4*A*) that match the conserved regions from the multiple sequence alignment (*SI Appendix*, Fig. S3). Subsequently, we employed AlphaFold to reveal the contribution of the conserved ordered regions to the protein assembly in 3D. In silico predictions suggest the conserved regions adopt secondary helical structures (*SI Appendix*, Fig. S4*B*) and may be functional domains. Interestingly, one of the predicted gnathostome helices falls in the short exon 2 (helix 5 – H5), potentially representing the predicted C-terminus functional domain. Overall, the tertiary structure of the vertebrate MN1 appears to possess a core formed by the conserved helices (5 in gnathostomes, 2-6 in cyclostomes) surrounded by a large outer disordered region (*SI Appendix*, Fig. S4*B*), a three-dimensional structure typically associated with signaling, regulation and essential for protein-protein interactions (26). Despite displaying less than 10% protein similarity to vertebrates (*SI Appendix*, Table S2), the three-dimensional structure of the invertebrate proto-MN1 proteins is similar to the vertebrate MN1 (*SI Appendix*, Fig. S5). The most striking difference is the presence of a basic helix-loop-helix DNA-binding domain in proto-MN1 of hemichordates, cephalochordates, and Asteroidea (*SI Appendix*, Fig. S5 and Table S3), a hallmark of transcription factors in eukaryotes (27, 28).

Due to the implication of *MN1* exon 2 in human developmental disorders, we have further explored this region across vertebrates (24, 25) (*SI Appendix*, Fig. S6). Out of the 26 identified pathogenic or likely pathogenic variants in the human *MN1* gene, 11 are located in exon 2. Additionally, 2 of the pathogenic variants are found in the predicted H5 (*SI Appendix*, Fig. S6*A*). Both PONDR and AlphaFold predictions suggest the presence of a secondary helical domain (H5) in the gnathostome exon 2, a structure not present in the cyclostome MN1-like proteins (*SI Appendix*, Fig. S6 *B* and *C*).

Altogether, we identified highly conserved MN1 ordered regions that are under strong selection, thereby remained unchanged over evolutionary timescales (23). PONDR and AlphaFold predictions further indicate the presence of a functional C-terminus domain encoded by exon 2 in gnathostomes and that this unknown protein domain may be essential for the *MN1* function (for more details, see the Discussion). The predicted proto-MN1 protein structure additionally supports the vertebrate *MN1* evolved from an ancestral deuterostome founder gene. However, the presence of a DNA-binding domain in basal deuterostomes together with the lack of homologous sequences to the vertebrate MN1 exon 2, suggest the gene underwent structural and functional divergence at the onset of vertebrates.

### *MN1* Embryonic Expression Pattern Is Conserved in Vertebrates.

**Fig. 2.**
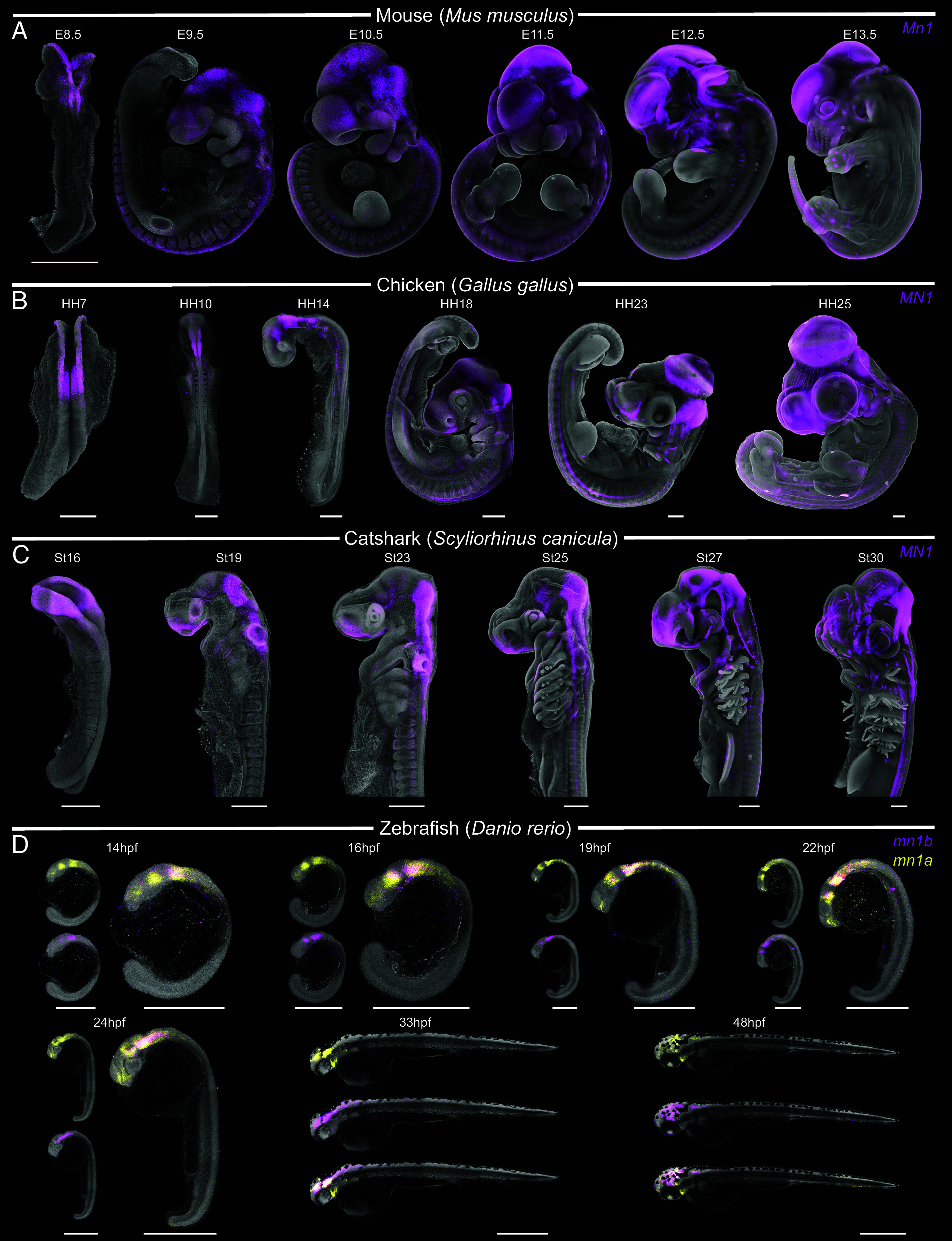
Expression pattern of *MN1* during embryogenesis across gnathostome species. *MN1* expression during mouse (*A*), chicken (*B*), small-spotted catshark (*C*), and zebrafish (*D*) development. Note the sparse *Mn1* expression signal in migrating cranial neural crest cells (CNCCs) laterally to the cephalic portion of the neural tube at E8.5. In chicken, *MN1* expression can be observed in the presumptive hindbrain at HH7, before cranial neural crest cell migration (HH9-10). *MN1* transcripts are not detected in the trunk or tail regions of any of the analyzed catshark embryos. Zebrafish possess two *mn1* paralogs (*mn1a* and *mn1b*) with spatially distinct expression profiles. Both *mn1* paralogs are expressed in the CNS and partially overlap in the developing hindbrain during the early stages (14 to 24 hpf). In the consecutive stages (33 to 48 hpf), *mn1a* exhibits more restricted expression within the facial mesenchyme while *mn1b* remains expressed in the CNS. (Scale bar, 500um.) At least three embryos were assayed per species and stage.

Because functionally and structurally conserved genes tend to exhibit conserved expression patterns (29), we examined the *MN1* expression along vertebrate embryogenesis. First, we employed single mRNA in situ hybridization (HCR) in four distant representatives of the gnathostome lineage (mouse, chicken, zebrafish, and small-spotted catshark) along consecutive embryonic stages, starting with the onset of neurulation and early brain morphogenesis till the stage when the general blueprint of the future head is established (Fig. 2).

In the early developmental stages, *MN1* is predominantly expressed within the developing central nervous system (CNS) (Fig. 2) across gnathostomes, with a particularly strong expression in the anterior neural ridge (fundamental for forebrain patterning) (30, 31) and the hindbrain. In the subsequent developmental stages following brain segmentation, *MN1* is additionally expressed in the facial mesenchyme in all analyzed gnathostome species. Interestingly, *MN1* transcripts were additionally detected outside of the craniofacial region, specifically in the developing skeletal elements of the forelimbs (*SI Appendix*, Fig. S7 *A* and *B*) and pectoral fins (*SI Appendix*, Fig. S7*C*). To further explore the cellular sources of *MN1* during embryogenesis, we took advantage of available single-cell transcriptomic datasets of developing mouse, human, and zebrafish and screened for *MN1*-expressing cell types (*SI Appendix*, Tables S4–S6). We found an overall matching cell type representation involved in establishing the *MN1* expression patterns. Radial glia, neuroblasts, differentiated neurons, and isthmic organizer cells establish the *MN1* expression in the CNS across gnathostomes. Moreover, neural crest cells, mesenchymal progenitors, and committed pharyngeal and head mesenchymal cells are among the conserved *MN1*-expressing cell types in the mouse and zebrafish (*SI Appendix*, Tables S4 and S5). Single-cell transcriptomic studies also reveal high *MN1* expression levels in the human developing brain (*SI Appendix*, Table S6), providing additional support for the conserved expression patterns across gnathostomes.

We subsequently explored the expression of the cyclostome *MN1-like* gene across corresponding embryonic stages (Tahara St21-St29) in the arctic lamprey (*SI Appendix*, Fig. S8) (32). We visualized the *MN1-like* expression pattern together with *CYP26B1/C1a* and *WNT1*, markers of the developing hindbrain and midbrain in lamprey, respectively (33, 34). *MN1-like* is expressed in the entire developing CNS and expands further to the craniofacial mesenchyme of the first pharyngeal arch (st24), upper and lower lips, and branchial basket at later stages (St26-St29) (*SI Appendix*, Fig. S8). In summary, the *MN1-like* expression in lamprey generally recapitulates the expression pattern in gnathostomes (Fig. 2).

To better comprehend the *MN1* evolution in deuterostomes, we have explored the *proto-Mn1* expression patterns using available single-cell transcriptomic datasets and HCR (*SI Appendix*, Fig. S9 and Table S7). In the sea urchin, *proto-MN1* is predominantly expressed in the animal pole domain and oral ectoderm, with faint expression in early ectoderm and endoderm (*SI Appendix*, Fig. S9), suggesting *proto-MN1* being primarily involved in early embryogenesis. In cephalochordates, *proto-MN1* is mostly expressed in neural and non-neural ectodermal tissues and in the endoderm (*SI Appendix*, Table S7). Taken together, the highly conserved *MN1* spatiotemporal expression pattern across vertebrates suggests the gene carries out a core function. In contrast, *proto-MN1* is expressed in ectodermal and endodermal tissues in sea urchins and cephalochordates. The observed difference in expression domains suggests that the invertebrate *proto-MN1* and vertebrate *MN1* genes perform different functions. However, further experimental evidence using gene knock-out strategies will be required to fully assess the role of *proto-MN1* in nonvertebrate lineages.

### *Mn1* Knock-Out Mouse Model Recapitulates Abnormal Cranial Skeletogenesis.

The original *Mn1* mouse mutant line from Meester-Smoor and colleagues (13), where severely affected intramembranous ossification was observed, was unavailable to us. Therefore, we generated *Mn1* knock-out line (*Mn1^−/−^*, mutant) using the CRISPR/Cas9 genome-editing technology. The line carries a 17bp deletion in the proximity and downstream of the start codon, causing a frameshift and resulting in a truncated Mn1 protein (*SI Appendix*, Fig. S10 *A* and *B*). We observed expected Mendelian proportions in E9.5-E13.5 embryos, with a slight decrease of mutant E15.5-E18.5 embryos and a significantly smaller proportion of mutant pups at the time of weaning, indicating perinatal lethality of *Mn1* mutants (*SI Appendix*, Fig. S10*C* and Table S8). We collected E15.5 and E18.5 embryos of all genotypes and subjected them to microcomputed tomography (μCT). Morphometric analysis of segmented skull bones revealed several abnormalities (*SI Appendix*, Figs. S11 and S12 and Dataset S2). Mutant embryos showed incomplete palate fusion (*SI Appendix*, Fig. S13 and Dataset S3) and abnormal shape of multiple cranial bones, such as the alisphenoid, squamosal, vomer, and supraoccipital, with overall reduced bone surfaces (*SI Appendix*, Figs. S11 and S12). Interestingly, heterozygous *Mn1* (*Mn1^+/-^*) individuals display intermediate phenotypes, such as varying degrees of incomplete palate fusion and reduced surface of cranial bones, supporting a dose-dependent effect of *Mn1* (13, 14). µCT analysis of the rare *Mn1* mutants that survived till adulthood shows that the mutants display similar craniofacial defects, with abnormal palate shape, deformed alisphenoid, and suture closure failures (coronal and interfrontal sutures) (*SI Appendix*, Fig. S14). Although this *Mn1* knock-out line exhibits a milder phenotype compared to the original mouse knock-out model (13), the representation of affected bones, abnormal palatal fusion, and lack of malformations present in the appendicular skeleton (*SI Appendix*, Figs. S11–S13) are consistent with the previously reported findings (*SI Appendix*, Table S9), rendering this line suitable for in vivo investigation of *Mn1* biology beyond the skeletal phenotype.

### *Mn1* Regulates Retinoic Acid Signaling in the Developing Hindbrain.

**Fig. 3.**
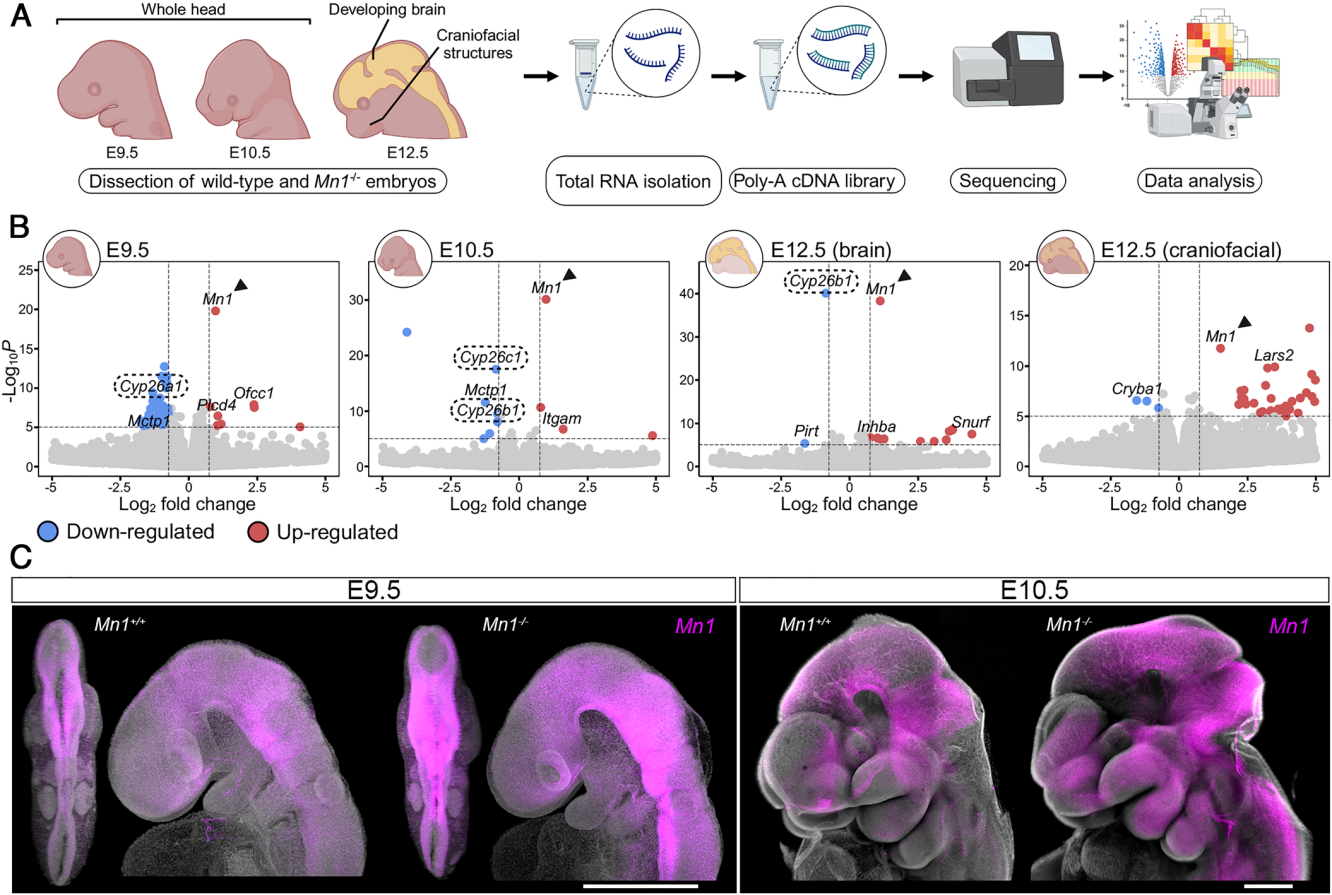
Gene expression analysis in WT and *Mn1^−/−^* embryos. (*A*) Overview of the RNA-seq experimental design. *Mn1^+/+^* and *Mn1^−/−^* embryos were collected from *Mn1^+/-^* intercrosses. n = 4 per genotype and embryonic stage were included in the analysis. The outliers were removed (*SI Appendix*, Fig. S15), and a minimum n = 3 per condition were further considered in bioinformatic analyses. At E12.5, brain and craniofacial structures were microdissected and processed independently to account for differences between nervous and mesenchymal tissues. (*B*) Volcano plots of statistically significant DEGs. Dots indicate genes significantly upregulated (red) or downregulated (blue) in *Mn1^−/−^*. The most relevant genes are highlighted, with genes involved in retinoic acid degradation surrounded by a dashed box and *Mn1* marked by a black arrowhead. (*C*) HCR images confirm the *Mn1* expression differences between WT and *Mn1^−/−^* embryos. (Scale bar, 500 μm.) At least three embryos were assayed per genotype and developmental stage.

To address the *Mn1* function during embryogenesis and identify molecular components or signaling pathways downstream of *Mn1*, we utilized the generated *Mn1* knock-out mouse model. We dissected the developing heads of E9.5, E10.5, and E12.5 embryos, where the E12.5 heads were additionally separated into brain and remaining craniofacial tissues and processed independently (Fig. 3*A*). Four independent samples from each genotype and developmental stage underwent bulk mRNA sequencing (RNA-seq). The acquired data were analyzed to extract differentially expressed genes (DEGs) between *Mn1^+/+^* (wild type, WT) and *Mn1^−/−^* (mutant) embryos (Fig. 3*B* and *SI Appendix*, Fig. S15). Pairwise comparison yielded slightly different DEGs (*SI Appendix*, Table S10 and Dataset S4) for each developmental stage, however, the top upregulated gene in mutants of all stages was *Mn1* (Fig. 3*B*, *SI Appendix*, Fig. S15 and Table S10), indicating the attempt for transcriptional compensation in mutant embryos (35, 36). The obtained results were corroborated by HCR, confirming visibly higher *Mn1* levels in the mutants compared to their WT littermates (Fig. 3*C*).

**Fig. 4.**
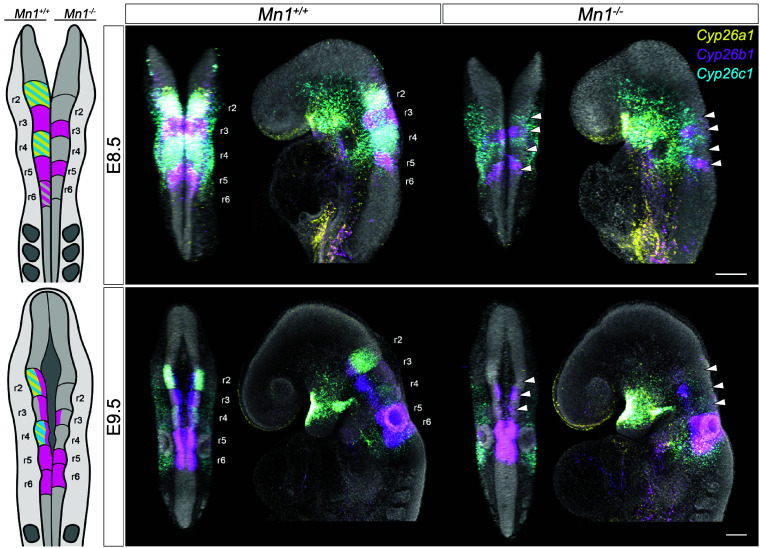
*Cyp26* gene expression is downregulated in the *Mn1* mutants. Schematic representation and HCR of *Cyp26* gene expression domains in the developing hindbrain in E8.5 (*Top*) and E9.5 (*Bottom*) *Mn1* mutant embryos compared to their WT littermates. The cartoons represent WT (*Left* side) and *Mn1* knock-out (*Right* side) embryos. White arrowheads point at the main differences in gene expression in r2-r5 segments. Note the decreased *Cyp26b1* expression in r2/r5 and the absent *Cyp26a1,c1* expression in r2/r4 in the *Mn1* mutants. (Scale bars, 100 μm.) At least three embryos were assayed per genotype and developmental stage.

Notably, the three members of the *Cyp26* gene family were downregulated in mutant samples (Fig. 3*B* and *SI Appendix*, Fig. S15 and Table S10). Due to the known expression of *Cyp26* genes (*Cyp26a1*, *Cyp26b1,* and *Cyp26c1*) in the developing CNS and their consistent downregulation in the analyzed stages, these genes were selected for further validation. The *Cyp26* genes are known to be involved in hindbrain segmentation, which occurs in embryonic stages E8.5-E9.5 in the mouse model; therefore, WT and mutant samples from these two developmental stages were selected for validation (Fig. 4).

The most remarkable difference in expression was found in the developing hindbrain, with a prominent decrease of *Cyp26b1* expression in rhombomere (r) r2-r4 and an absence of *Cyp26a1* and *Cyp26c1* expression in r2 and r4 in *Mn1* mutants (Fig. 4). The expression downregulation persists in subsequent developmental stages following hindbrain segmentation (E10.5-E12.5), with an additional loss of *Cyp26b1* expression domain in the midbrain of *Mn1* mutants (*SI Appendix*, Fig. S16). The expression of mutant *Mn1* exhibits an apparent effect on the *Cyp26* gene expression specifically in the developing CNS, while the expression of *Cyp26* genes in facial and limb mesenchyme and oral ectoderm remain unaffected (Fig. 4 and *SI Appendix*, Fig. S16). In summary, RNA-seq analysis and validations using HCR indicate that *Mn1* is essential for the establishment of the *Cyp26* gene expression domains in the developing hindbrain.

### *Mn1* Is Involved in Hindbrain Segmentation and Rhombomere Segmental Patterning.

**Fig. 5.**
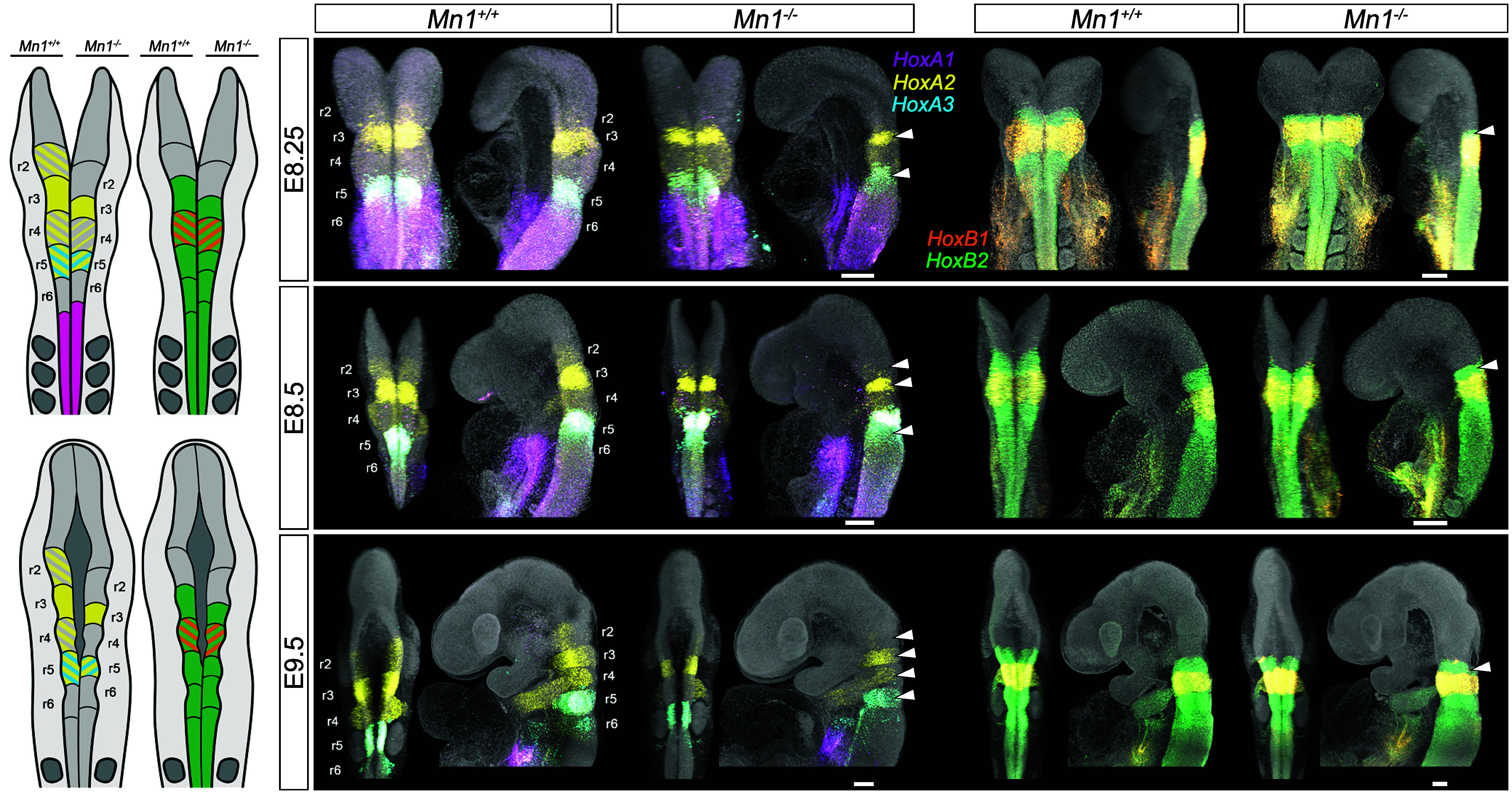
Hox gene expression during hindbrain segmental patterning is affected in *Mn1* mutants. Schematic representation and HCR of *HoxA* and *HoxB* gene expression domains in the developing hindbrain in E8.25 (*Top*), E8.5 (*Middle*), and E9.5 (*Bottom*) *Mn1* mutant embryos compared to their WT littermates. The *Left* side of the cartoons represents WT and the right side *Mn1* knock-out embryos. White arrowheads point at the main differences in the rhombomeric segments. Note the reduced expression of *HoxA2* and *HoxB1* in the significantly smaller r3 segment in the *Mn1* mutants. (Scale bar, 100 μm.) At least three embryos were assayed per genotype and developmental stage.

*Cyp26* genes encode for retinoic acid (RA)-degrading enzymes that are part of a conserved molecular machinery and establish *Hox* gene expression patterns (33, 37). The RA/*Hox* program is essential for the correct segmentation and patterning of the hindbrain in vertebrates (33, 38). To understand how the downregulation of *Cyp26* expression in the *Mn1* mutant embryos impacts hindbrain patterning and segmental expression of *Hox* genes, we measured the height of rhombomeres r3-r5 (*SI Appendix*, Fig. S17 and Dataset S5) and mapped the *HoxA* and *HoxB* expression domains in WT and mutant embryos (E8.25-E9.5) (Fig. 5).

**Fig. 6.**
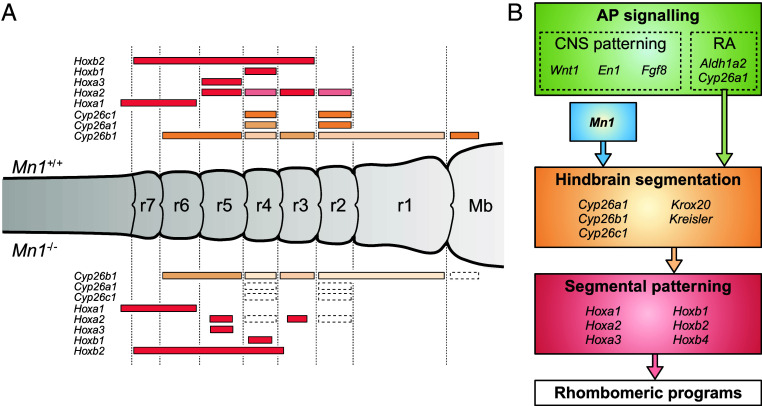
Summary of the *Mn1* mutation effect in the developing hindbrain. (*A*) Schematic representation of the mouse developing hindbrain showing the main differences in the expression domains of genes involved in the RA/*Hox* program between WT and *Mn1* mutants. (*B*) The interactions between signaling components known to orchestrate hindbrain segmentation and the suggested *Mn1* integration into the ancient RA/*Hox* program. AP, anteroposterior; Mb, midbrain.

We detect morphological differences in the rhombomere size in E9.5 embryos, where r3 and r5 exhibit significantly smaller height in the mutants (*SI Appendix*, Fig. S17). At E8.25-E9.5, mutant embryos show decreased or absent expression of *HoxA2* in r2-r5 and reduced *HoxA3* expression in r5 (Fig. 5). *HoxA1* expression appears unaffected. In mutant embryos, we furthermore observe a strong *HoxB1* expression in r4; however, the shape of the expression domain slightly varies among the mutant embryos (Fig. 5). A thin *HoxB2*+ expression domain defines the significantly shorter r3 segment in the *Mn1* mutant embryos and coincides with the reduced *HoxA2+* domain in this segment. Altogether, the rhombomeric segments defined by the *Hox* genes are visibly affected in the *Mn1* mutant embryos and supported by the observed morphological differences. The observed changes in the *Hox* program within the hindbrain are additionally reflected in the migrating neural crest cells, exhibiting a decreased expression of *HoxA2* and *HoxB2* (Fig. 5). These findings provide evidence for the role of *Mn1* in hindbrain segmentation and patterning via the RA signaling pathway (Fig. 6).

### Altered Neural Crest Cell Migratory Streams and Cranial Nerve Formation in *Mn1* Mutants.

CNCCs originating from the prospective hindbrain give rise to a substantial part of the skull (39, 40), including bones that are most affected by the *Mn1* mutation (*SI Appendix*, Table S9). To evaluate whether altered hindbrain patterning in *Mn1* mutant embryos also affects the specification and migration of CNCCs and, to assess whether the altered cranial bones in *Mn1* mutants could be caused by potential differences in CNCCs migration, we visualized the CNCCs in E8.0-E9.5 mutant and WT embryos (*SI Appendix*, Fig. S18). The anatomical domains occupied by CNCCs at the time of their specification and delamination appear comparable between WT and knock-out embryos. However, the migratory streams of CNCCs (E9.0-E9.5) exhibit different morphology and are visibly thinner in the *Mn1* mutants compared to the WT controls (*SI Appendix*, Fig. S18). Moreover, due to the reduced size of r3 in *Mn1* mutants (*SI Appendix*, Fig. S17), the trigeminal and hyoid CNCCs migratory streams are positioned closer to each other than in the WT littermates.

Sox10 staining at E9.5 embryos suggests impaired morphology of the trigeminal ganglia in *Mn1* mutants (*SI Appendix*, Fig. S18), structure derived from the hindbrain r2. Hence, to determine whether the molecular and morphological changes resulting from *Mn1* mutation impact the cranial nerves and ganglia, we stained for developing peripheral nervous system in E9.5-E10.5 embryos (*SI Appendix*, Fig. S19). Nine out of twelve cranial nerves arise from the folding neural plate region of the prospective hindbrain. We observed a range of phenotypic changes in cranial nerves, from reduced size of the trigeminal nerve to complete absence of the glossopharyngeal nerve, pointing at the role of *Mn1* in cell specification within the neural plate and in the development of peripheral innervation (*SI Appendix*, Fig. S19).

## Discussion

Morphological innovations are lineage-specific traits fulfilling novel functions, adaptations to the environment, and driving diversification and speciation. These innovations arise through gene co-option and cis-evolution, producing new molecular networks and developmental programs or rewiring the existing ones (4). The third fundamental mechanism underlying evolutionary innovations is the emergence of novel genes (7, 41).

Recently, the link between novel genes (irrespective of the mechanism underlying their emergence) and evolutionary novelties has been demonstrated by several studies (7, 42–48), highlighting the need to comprehend how novel genetic components are incorporated into existing developmental programs to generate novelties. Here, we investigate the evolution and function of the vertebrate *MN1*, a gene that evolved from a founder gene of deuterostome origin. The vertebrate *MN1* exhibits structural differences from its distant invertebrate homologs (*proto-MN1*). However, both contain several conserved protein domains and are located in genomic regions known to be derived from the same ancestral chromosome. Studies in comparative genomics have identified chordate linkage groups (CLG), where physical gene linkage is conserved in the absence of collinear synteny, due to the accumulation of inversions and other chromosomal rearrangements over time. All identified *MN1* genes in gnathostomes, cyclostomes, and invertebrate deuterostomes are located in genomic regions of the same CLG-G (18, 19, 49), further supporting their shared ancestry. *proto-MN1* contains a basic helix-loop-helix DNA-binding domain, a hallmark of transcription factors (27). Contrary to that, the vertebrate *MN1* gene appears to have lost the DNA-binding domain and may thus act as a transcriptional cofactor (50). While further in vivo functional validations in invertebrate deuterostomes are required to understand the function of *proto-MN1*, this study showcases how ancient genetic components evolve to elaborate developmental programs and fuel evolutionary innovations (51, 52).

The gnathostome *MN1* gene exhibits further structural differences from the cyclostome *MN1-like* gene. Whether a structural evolution occurred in the gnathostome ancestor or the differences arose from divergence due to the distinct genome duplication events after the gnathostome-cyclostome split cannot be ascertained. The emergence of the gnathostome-specific *MN1* exon 2 may represent a case of episodic evolution. After the structural evolution of the gene domain encoding for the C-terminus, the whole gene became fixed and maintained under strong selection in gnathostomes. This indicates that the integrity of MN1 over evolutionary time is essential for its functionality and central to the formation of a specific trait. While the function of the MN1 C-terminus remains unknown, mutations affecting this region are causative of a human congenital syndrome, “MN1 C-terminal truncation syndrome” (MCTT), manifested by neurodevelopmental disorders resulting in intellectual disability, brain abnormalities, and distinctive facial features (midface hypoplasia, cleft palate, and other skeletal anomalies) (24, 53, 54). This suggests that the conserved domain encoded by exon 2 is essential for MN1 functionality and contributes to generating novelties within the central nervous and skeletogenic systems. Additionally, *MN1* is located in a craniofacial and neurodevelopmental disorders hotspot locus (Ch22q12 in humans) (55, 56), where deletions of varying lengths affect both exons and manifest by a broader phenotypic spectrum. However, the majority of cases are highly associated with intellectual disabilities and cleft palate (57, 58). Notably, deletions affecting the *MN1* gene in human pathological conditions are heterozygous. Homozygous deletions are considered embryonically lethal in humans, with a probability of *MN1* being loss-of-function intolerant (pLI) = 0.992 (gnomAD), highlighting its essential role in prenatal development as a key component of both neurodevelopmental and skeletogenic processes.

*MN1* exhibits a dynamic spatiotemporal expression pattern, a hallmark of essential developmental genes with pleiotropic effects. Although *Mn1* has been previously linked to cranial bone formation and osteoblast differentiation (13, 17), we find initial high expression levels within the developing CNS across vertebrates. *MN1* is most prominently expressed in the neuroectoderm of the prospective hindbrain and during its segmental organization. During early vertebrate development, the hindbrain is transiently divided into eight rhombomeres (r1-8) — segments, which will later acquire distinctive molecular and cellular programs (59, 60). The hindbrain is also the site of origin of CNCC populations: trigeminal stream emerging from r1/2, hyoid stream from r4, and postotic streams originating from r6/7 populate different niches in the developing embryo and contribute to the formation of cranial bones (39, 40). Previous studies showed the importance of brain segmentation for CNCC specification and migration. For instance, in *Cyp26a1/c1^−/−^* embryos, CNCCs arising from r4 are reduced in number and CNCCs arising from the midbrain fail to reach the frontonasal prominence (61). Additionally, disorganized CNCC migration originating from the r6 domain was observed in *Cyp26b1^−/−^* embryos (62). In line with the observed disruption of hindbrain segmentation and patterning in the *Mn1* mutants, we detect morphological changes in trigeminal and hyoid neural crest streams originating from r1/2 and r4, aligning well with previous observations, jointly indicating that, to some extent, the altered CNCC biology might underlie the craniofacial mutant phenotype. Additionally, alisphenoid and squamosal bones, formed by CNCCs emerging from the r1-2, are among the most affected bones in *Mn1* mutant embryos (13, 39) (*SI Appendix*, Figs. S11 and S12 and Table S9).

Nine out of the twelve cranial nerves arise from the closing neural tube of the prospective hindbrain area (60). We observed that these nerves, most prominently the 9th (glossopharyngeal) and 5th (trigeminal), exhibit morphological differences between control and *Mn1* mutant embryos. A prominent feature of MCTT syndrome is rhombencephalosynapsis, a rare cerebellar malformation characterized by complete or partial fusion of the cerebellar hemispheres (24). Interestingly, the cerebellum develops from the first rhombomere (63), a rhombomeric segment devoid of *Cyp26a1* and *Cyp26c1* expression in the *Mn1* mutant embryos. Altogether, these results indicate that *Mn1* is especially important for the formation of structures arising from the emerging hindbrain and that abnormal patterning and/or reduced size of the hindbrain segments results in differences in migratory streams and forming cranial nerves, leading to abnormal craniofacial development.

*Mn1* mutant embryos display cleft palate and abnormal cranial bones such as the alisphenoid and squamosal (13) (*SI Appendix*, Figs. S11–S13 and Table S9). Strikingly, a phenotype closely resembling the *Mn1* mutants can be observed in WT mouse embryos treated with (RA) between E8.5-E10.5 (64, 65). The RA signaling pathway represents an ancient molecular machinery that emerged early in animal evolution (37, 66). The RA signaling pathway is reportedly involved in metamorphosis of echinoderms (67) and known to orchestrate the establishment of the anteroposterior axis in chordates by regulating the expression of nested *Hox* genes (61, 68, 69). Across vertebrates, the ancient RA/*Hox* developmental program is additionally involved in hindbrain segmentation, a vertebrate-specific trait of the forming nervous system (33, 37). The importance of adequate RA levels is further underlined by the existence of a self-regulatory loop. Increased RA levels induce the expression of RA-degrading enzymes (CYP26) and inhibit the expression of RA-producing enzymes (ALDH1A2), creating a negative feedback loop (33, 70, 71). One of the most evident transcriptomic differences between WT and *Mn1* mutant embryos is the significantly upregulated expression of mutant *Mn1* and downregulation of *Cyp26* genes. These results imply that *Mn1* is part of the RA self-regulatory loop, and compensating mechanisms are in place to attempt to restore the physiological balance. The morphometric analysis of the cranial bones and palatal closure shows intermediate phenotypes in heterozygotes (*Mn1^+/-^*) (*SI Appendix*, Figs. S11–S13), thus supporting that the *Mn1* effect is dose-dependent. Altogether, we present evidence that *Mn1* is involved in the autoregulatory loop and modulates the dynamics of the RA signaling pathway.

Disbalance of RA levels has been previously achieved by pharmacological inhibition of CYP26 and ALDH1A2 enzymes, genetic ablation of *CYP26* genes, and direct administration of RA by injection. The collective evidence from the three experimental strategies showed abnormal hindbrain-derived neural crest cells (61, 62, 64), hypoplastic cranial ganglia with nerve branching defects (62, 64, 72–74), and disrupted rhombomere segmental identity manifested by spatial changes in *Hox* gene expression along the nervous system (33, 38). These phenotypes strikingly correlate with the observations from the generated *Mn1* mutant line, where the downregulation of *Cyp26b1* and absence of *Cyp26a1* and *Cyp26c1* expression domains in the developing hindbrain accompany the high expression of the mutant loss-of-function *Mn1*. Moreover, mutations in the *Cyp26(a1,b1,c1)* genes manifest in human patients by calvarial mineralization defects, craniosynostosis, hypertelorism, and learning disabilities (75), clinical features within the neuro-skeletal systems resembling the phenotype of patients with *MN1* deletions. From yet another clinical perspective, RA-based therapies are used to treat acute promyelocytic leukemia. Interestingly, a high expression of WT *MN1* is a negative prognostic factor, and a patient cohort exhibiting high *MN1* levels is resistant to the RA therapies (76). Based on our data, we deduce that high *MN1* levels in patient leukemic cells increase *Cyp26* expression and induce rapid degradation of RA, reducing its therapeutic effect. Altogether, the presented experimental and clinical evidence jointly highlight the molecular link between *MN1* and RA/*Cyp26* molecular pathway and its involvement in correct hindbrain patterning and skull development. Whether cyclostome *MN1-like* (despite the structural differences, particularly in exon 2) fulfills a comparable function in hindbrain patterning will require in vivo functional validation using CRISPR/Cas9 tool in lamprey or hagfish, an approach that still represents a challenge in this phylogenetic node.

The emergence of the complex brain and the skull are macroevolutionary innovations of the vertebrate lineage that were driven by processes such as genome duplications and changes in gene regulation. However, an additional mechanism underpinning novelties, the emergence of novel genes, was largely unexplored. Growing evidence highlights how novel genes fueled the formation of new molecular and developmental programs or rewired the existing ones and powered the emergence of lineage-specific traits. Here, we demonstrate that *MN1* originated from an ancestral deuterostome founder gene and underwent structural changes in vertebrates and gnathostomes, where it was incorporated into the ancient molecular programs controlling developmental signaling and patterning. *MN1* integration into the conserved RA/*Hox* signaling pathway represents a developmental novelty and part of the genetic basis underlying the codevelopment and coevolution of the brain and skull. The elucidation of *MN1* biology additionally enables us to understand the developmental and genetic basis of various human neurodevelopmental and craniofacial syndromes and suggests the mechanisms behind RA-treatment resistance in the leukemic patient cohort.

## Methods

### Animal Information.

All animal work and experimental procedures were approved by the Local Ethics Committee Germany (Kiel, Germany) and conducted according to the Federation of European Laboratory Animal Science Associations guidelines and the German animal welfare law (Tierschutzgesetz § 11). The mouse strains C57Bl/6J and C57Bl/6NCrl, strain codes 632 and 027, respectively, were obtained from Charles River Laboratories (Germany) and housed at the Max Planck Institute for Evolutionary Biology’s Animal Facility. The housing and experimental procedures are approved by the MLLEV (Ministerium für Landwirtschaft, ländliche Räume, Europa und Verbraucherschutz des Landes Schleswig-Holstein), under permits PLÖ-0004697, 4-1/17, and 53-6/20. Detailed information on the collection of embryonic samples and their processing is provided in *SI Appendix*, *Supplementary Methods*.

### Generation of *Mn1* Mutant Mouse Strain.

The *Mn1* mutant mouse line was generated using the CRISPR/Cas9 genome editing system described elsewhere. Briefly, recombinant Cas9 and sgRNA targeting the first exon of *Mn1* (5′-CCGGGCCTCCTACTGGCCCCGTGG-3′) were microinjected into fertilized C57Bl/6N mice oocytes that were then transferred into the oviducts of pseudopregnant foster mothers. F0 mice were screened for successful mutations by Sanger sequencing analysis. The generated line carries a 17bp deletion downstream of the start codon, creating a shift in the reading frame, and is predicted to generate a truncated MN1 protein. See *SI Appendix*, *Supplementary Methods* for genotyping strategy.

### Phylogenetic Analysis.

BLASTp (Basic Local Alignment Search Tool protein) searches were performed to screen for the presence or absence of homologous MN1 amino acid sequences in NCBI (https://www.ncbi.nlm.nih.gov/) and Ensembl (https://www.ensembl.org/index.html) using the human MN1 sequence (NP_002421.3) as a query. Additionally, InterPro (https://www.ebi.ac.uk/interpro/) and UniProt (https://www.uniprot.org/) databases were also checked for MN1 entries. A total of 92 MN1 homologs were retrieved from genomes along vertebrate phylogeny and 14 invertebrate distant homologs (proto-MN1) were retrieved from invertebrate deuterostome genomes (*SI Appendix*, Table S1). Proteomes of representative species were downloaded from UniProt and/or NCBI and assessed locally by a hidden Markov model using HMMER3.4 (http://hmmer.org/) with default parameters (*SI Appendix*, Table S2), which overall supported the BLASTp results. Furthermore, searches in the Echinoderm database Echinobase (https://www.echinobase.org) corroborated the BLASTp hits observed in this lineage (*SI Appendix*, Table S2). Following previous studies in comparative genomics (8), we set a cutoff value of e < 10^−3^-10^−4^ to consider a hit nonrandom in protein similarity searches. Reverse BLASTp was performed to verify the accuracy of the hits (*SI Appendix*, Table S2). Detailed information on all sequences, species, and genomes used in this study is provided in *SI Appendix*, Table S1.

Protein sequence alignments were used because of their increased sequence conservation. A total of 106 amino acid sequences (92 vertebrate and 14 invertebrate) were selected for further phylogenetic analysis (*SI Appendix*, Table S1). Multiple sequence alignment was performed using the MUSCLE algorithm (77) with default settings in MEGA11 (78). Geneious Prime 2024.0.2 was used for alignment visualization. Based on the unmodified alignment, a maximum likelihood phylogenetic tree was computed in IQ-TREE 1.6.12 (79). The 14 invertebrate proto-MN1 sequences were included in the analysis as an outgroup, and the best-fit model was predicted by the ModelFinder function (80), which was JTT+F + R5. 500 bootstrap iterations were implemented to evaluate branch-node support. The resulting tree was visualized, and the midpoint rooted in iTOL (https://itol.embl.de/).

### Group-Scale Synteny Analysis.

Chromosomal-level synteny was performed using GENESPACE v1.3.1 (81) with OrthoFinder v2.5.5 (82) and MCScanX v1.0.0 (83). The genomic.gff.gz and translated.cds.faa.gz files from 14 representative vertebrate species and 3 invertebrate species were retrieved from NCBI (genome assemblies are provided in *SI Appendix*, Table S1) and filtered to remove any unplaced scaffolds. Due to inherent genomic differences of the compared species (as a result of large evolutionary distances, independent genome duplication events and intrachromosomal rearrangements and inversions), we performed the analysis in two steps: First, we compared gnathostomes-cyclostomes and, subsequently, cyclostomes-invertebrates. Finally, the results were merged together using the brown hagfish (*Eptatretus atami*) as anchoring species. Detailed parameters together with species names are specified in *SI Appendix*, *Supplementary Methods*.

### In Silico Predictions.

16 MN1 protein sequences from 14 representative vertebrate species and 14 proto-MN1 protein sequences from 12 invertebrate species were characterized further. The degree of disorder per residue was calculated by the PONDR VL-XT algorithm (Prediction of Natural Disordered Regions — http://www.pondr.com/). Scores above 0.5 are predicted to be disordered, whereas scores below 0.5 are considered ordered regions. AlphaFold v2.3.2 (https://github.com/deepmind/alphafold) was run using the default parameters (84), querying the suggested databases. The –max_template_date function limiting the use of templates generated before May 2010 was used to obtain unbiased models. The prediction with the highest confidence score from each species was selected for further cross-species comparisons. The selected 16 vertebrate MN1 and the 14 invertebrate proto-MN1 amino acid sequences were further screened for protein domains against the CDD v3.21-62456 PSSMs database of NCBI’s Conserved Domains Database repository (https://www.ncbi.nlm.nih.gov/Structure/cdd/cdd.shtml), with default parameters (*SI Appendix*, Table S3).

### In Situ Hybridization Chain Reaction.

Hybridization chain reaction (HCR) in all embryonic samples was performed following the manufacturer’s protocol (Molecular Instruments, Inc., USA), with minor modifications (*SI Appendix*, *Supplementary Methods*). Images were captured using a Zeiss LSM980 with Airyscan2 confocal microscope, Plan-Apochromat 5×/0.16 M27 (Zeiss 420630-9900), Plan-Apochromat 10×/0.45 M27 (Zeiss 420640-9900) and LD C-Apochromat 40×/1.1 W Korr UV-VIS-IR (Zeiss 421867-9970-000) objectives were used. Images were exported from ZEN 3.9 software. Probe sets for each gene were either designed and purchased from Molecular Instruments, Inc., or designed using the HCR 3.0 probe maker (https://github.com/rwnull/insitu_probe_generator) and purchased from Integrated DNA Technologies (IDT, USA). Detailed information on each specific gene probe set can be found in *SI Appendix*, Table S11. All HCR buffers were prepared in-house following the manufacturer’s protocols.

### Immunofluorescence Staining.

Fixed embryonic samples designated for the whole-mount staining were bleached to reduce autofluorescence as described above (HCR). The following primary antibodies were used: mouse anti-Tuj1 (Promega G7121; 1:500) and goat anti-Sox10 (R&D Systems AF2864; 1:1000). Secondary antibodies were produced in donkey and conjugated with Alexa Fluor 647 and 555 (Abcam; 1:1000). See *SI Appendix, Supplementary Methods* for detailed information.

### Bulk RNA Sequencing and Analyses.

The head was dissected from E9.5, E10.5, and E12.5 embryos originating from crossing heterozygous *Mn1* parents. At E12.5, brains and the remaining craniofacial structures were microdissected and processed independently. The otic vesicle was used as a morphological landmark to separate the head from the rest of the body. The samples were snap-frozen in liquid nitrogen and stored at −80°C till further use. The remaining parts of the embryos were used for genotyping and sex identification. Only male embryos were used for the transcriptomic analysis to avoid sex-related variability. WT and *Mn1* mutant embryos were included in the analysis. Each sample was processed and sequenced individually, n = 4 samples per genotype. Details on mRNA extraction can be found in *SI Appendix*, *Supplementary Methods*. Sequencing was carried out on NovaSeq X Plus (PE150bp strategy), which generated around 40 million reads per sample. Subsequent bioinformatic analyses were performed in R v4.1.3. Hisat2 v2.2.1 (https://daehwankimlab.github.io/hisat2/) was used to align the reads to the house mouse genome (GCF_000001635.26), Stringtie v2.2.1 (https://ccb.jhu.edu/software/stringtie/) to assemble the transcriptome, and DESeq2 v1.34.0 (https://bioconductor.org/packages/release/bioc/html/DESeq2.html) to analyze differential gene expression. WT and *Mn1* mutant samples were compared within individual embryonic stages. Genes were considered to be differentially expressed if pAdj<0.005 and |FoldChange|>0.75. Volcano plots were created using the R package *EnhancedVolcano* v1.12.0 (https://github.com/kevinblighe/EnhancedVolcano). The RNA-seq dataset has been deposited at the Sequence Read Archive (SRA) under BioProject PRJNA1139193.

### Microcomputed Tomography (µCT) Analysis.

Samples were processed and scanned using established procedures and settings (*SI Appendix*, *Supplementary Methods*). NRECON reconstruction software (Bruker Corporation, Kontich, Belgium) was used to transform the 2D projection images into 3D volumes, and scanning artifacts were corrected (misalignment, ring artifacts, and beam hardening). Avizo3D Pro Software (ThermoFisher Scientific, Konrad-Zuse-Zentrum, Berlin, Germany) was used to segment out the skeletal elements and analyze the reconstructed images. All segmentations were performed by the same operator. For quantitative analysis, Avizo3D was used to retrieve the surface measurements from each segmented bone. One-way ANOVA with Tukey’s multiple comparison test was performed in R v4.1.3 using genotypes as independent variables and statistical significance was set at *P* < 0.05 (*P < 0.05, **P < 0.01, and ***P < 0.001). Bonferroni correction was applied to adjust for multiple testing comparisons. Violin plots were created in R Studio using the package *ggplot2* v3.5.1 (https://ggplot2.tidyverse.org).

Adult mice were sacrificed by CO_2_ overdose and their skin, organs, and soft tissues were manually removed before fixation. Skeletons were then fixed for 1 wk in 4% PFA in the supine position prior to scanning (for detailed settings see *SI Appendix, Supplementary Methods*).

### Statistics.

Where statistical analysis was performed, details about the test (type of test, sample sizes, and *P*-values) are indicated in the respective figure legends. Comprehensive information on the statistical analyses can be found in the accompanying *SI Appendix*.

## Supplementary Material

Appendix 01 (PDF)

Dataset S01 (PDF)

Dataset S02 (XLSX)

Dataset S03 (XLSX)

Dataset S04 (XLSX)

Dataset S05 (XLSX)

## Data Availability

All genomes and sequences used in this study are available at NCBI or Ensembl, and their accession numbers can be found in *Methods* and *SI Appendix*, Table S1. Interactive online resources from publicly available single-cell transcriptomic datasets were used to collect information of *MN1*-expressing cell types in the mouse (*SI Appendix*, Table S4), zebrafish (*SI Appendix*, Table S5), human (*SI Appendix*, Table S6), and cephalochordates (*SI Appendix*, Table S7); references can be found in the figure legend. The raw multiple sequence alignment of the 106 MN1 protein sequences is provided in Dataset S1. Microcomputed tomography source data of the palatal shelve distances and bone surface are provided in Datasets S2 and S3, respectively. Due to the large number and size of the raw files, tomographic reconstructions can be provided upon request. Processed RNA-seq data after DESeq2 are provided in Dataset S4. The RNA-seq dataset has been deposited at the Sequence Read Archive (SRA) under BioProject PRJNA1139193 (85). Source data of the rhombomere measurements can be found in Dataset S5. All other relevant information supporting the findings of this study is available within the article and its *SI Appendix* or from the corresponding author upon request. All analyses in this study were performed using readily available published programs and software packages listed in *Methods*.
