# Supplementary material for: Evolution of the essential gene MN1 during the macroevolutionary transition toward patterning the vertebrate hindbrain
Source: Proc Natl Acad Sci U S A. 2025 May 27;122(22):e2416061122. doi: 10.1073/pnas.2416061122 (PMC12146709; doi:10.1073/pnas.2416061122)
Supplement: Supplementary file 1 — Appendix 01 (PDF) [file pnas.2416061122.sapp.pdf]

PNAS

Supporting information for

Evolution of the essential gene *MN1* during the macroevolutionary transition towards patterning the vertebrate hindbrain

Authors

Elio Escamilla-Vega[1], Louk W.G. Seton[1], Stella Kyomen[1], Andrea P. Murillo-Rincón[1], Julian Petersen[2], Diethard Tautz[1], Markéta Kaucká[1*]

***Corresponding author**

Markéta Kaucká, Max Planck Institute for Evolutionary Biology, August-Thienemann-Str. 2, 24306 Plön, Germany. email: kaucka@evolbio.mpg.de

This PDF file includes:

- Figures S1 to S19
- Tables S1 to S11
- Supplementary Methods
- SI References

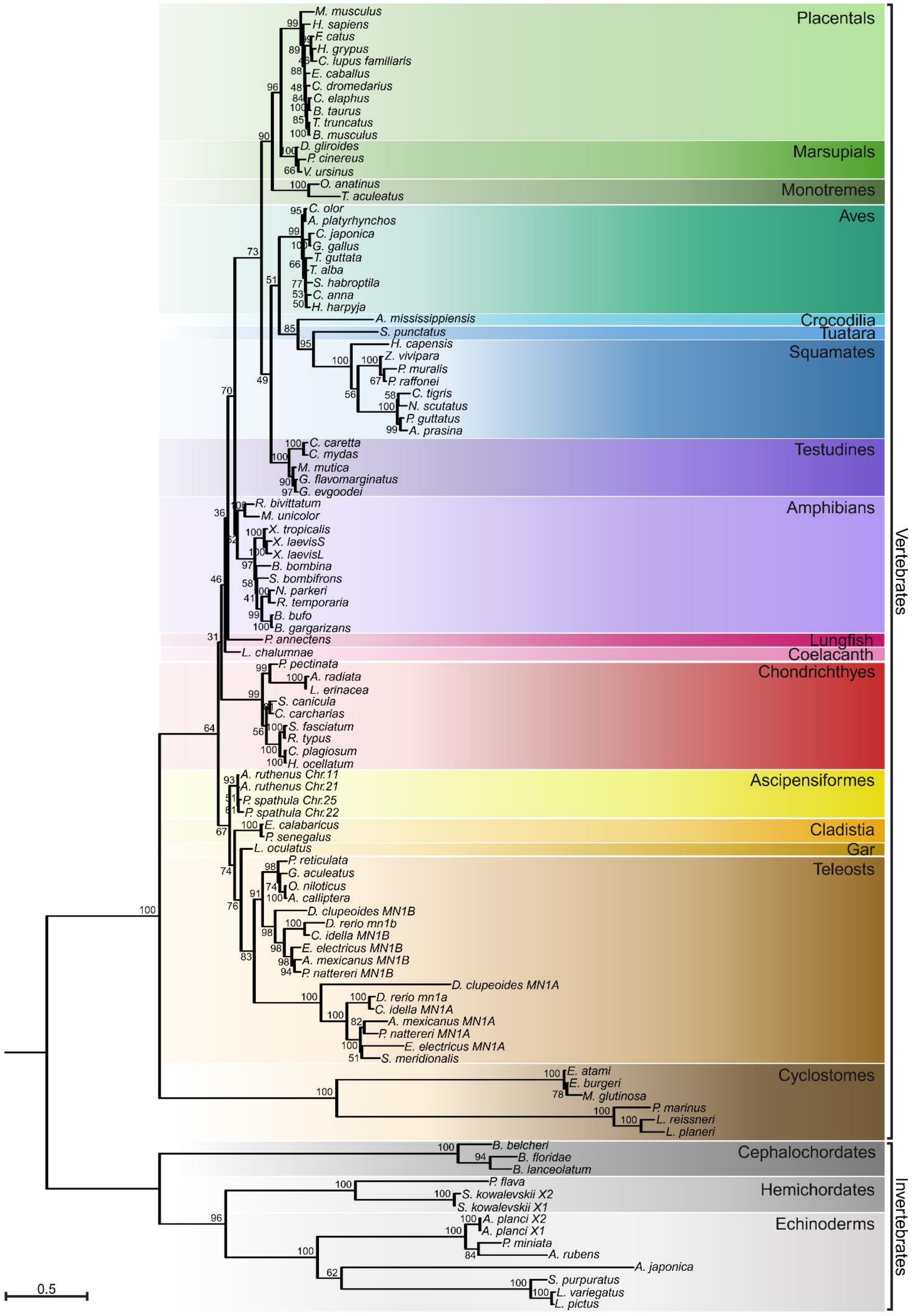
Placentals
Marsupials
Monotremes
Aves
Crocodilia
Tuatara
Squamates
Testudines
Amphibians
Lungfish
Coelacanth
Chondrichthyes
Ascipensiformes
Cladistia
Gar
Teleosts
Cyclostomes
Cephalochordates
Hemichordates
Echinoderms
Vertebrates
Invertebrates
M. musculus
H. sapiens
F. catus
H. grypus
C. lupus familiaris
E. caballus
C. dromedarius
C. elaphus
B. taurus
T. truncatus
B. musculus
D. gliroides
P. cinereus
V. ursinus
O. anatinus
T. aculeatus
C. olor
A. platyrhynchos
C. japonica
G. gallus
T. guttata
T. alba
S. habroptila
C. anna
H. harpyja
A. mississippiensis
S. punctatus
H. capensis
Z. vivipara
P. muralis
P. raffonei
C. tigris
N. scutatus
P. guttatus
A. prasina
C. caretta
C. mydas
M. mutica
G. flavomarginatus
G. evgoodei
R. bivittatum
M. unicolor
X. tropicalis
X. laevisS
X. laevisL
B. bombina
S. bombifrons
N. parkeri
R. temporaria
B. bufo
B. gargarizans
P. annectens
L. chalumnae
P. pectinata
A. radiata
L. erinacea
S. canicula
C. carcharias
S. fasciatum
R. typus
C. plagiosum
H. ocellatum
A. ruthenus Chr.11
A. ruthenus Chr.21
P. spathula Chr.25
P. spathula Chr.22
E. calabaricus
P. senegalus
L. oculatus
P. reticulata
G. aculeatus
O. niloticus
A. calliptera
D. clupeoides MN1B
D. rerio mn1b
C. idella MN1B
E. electricus MN1B
A. mexicanus MN1B
P. nattereri MN1B
D. clupeoides MN1A
D. rerio mn1a
C. idella MN1A
A. mexicanus MN1A
P. nattereri MN1A
E. electricus MN1A
S. meridionalis
E. atami
E. burgeri
M. glutinosa
P. marinus
L. reissneri
L. planeri
B. belcheri
B. floridae
B. lanceolatum
P. flava
S. kowalevskii X2
S. kowalevskii X1
A. planci X2
A. planci X1
P. miniata
A. rubens
A. japonica
S. purpuratus
L. variegatus
L. pictus
0.5

Fig. S1: Maximum-likelihood phylogenetic tree of the predicted MN1 sequences.

The tree was computed using the JTT+F+R5 model with 500 bootstrap iterations, with branch support values shown as a percentage. For better visualization, the tree was midpoint rooted with the invertebrate proto-MN1 sequences and major clades are colour-coded. Scale bar indicates evolutionary distance. Accession numbers of all sequences are provided in Table S1.

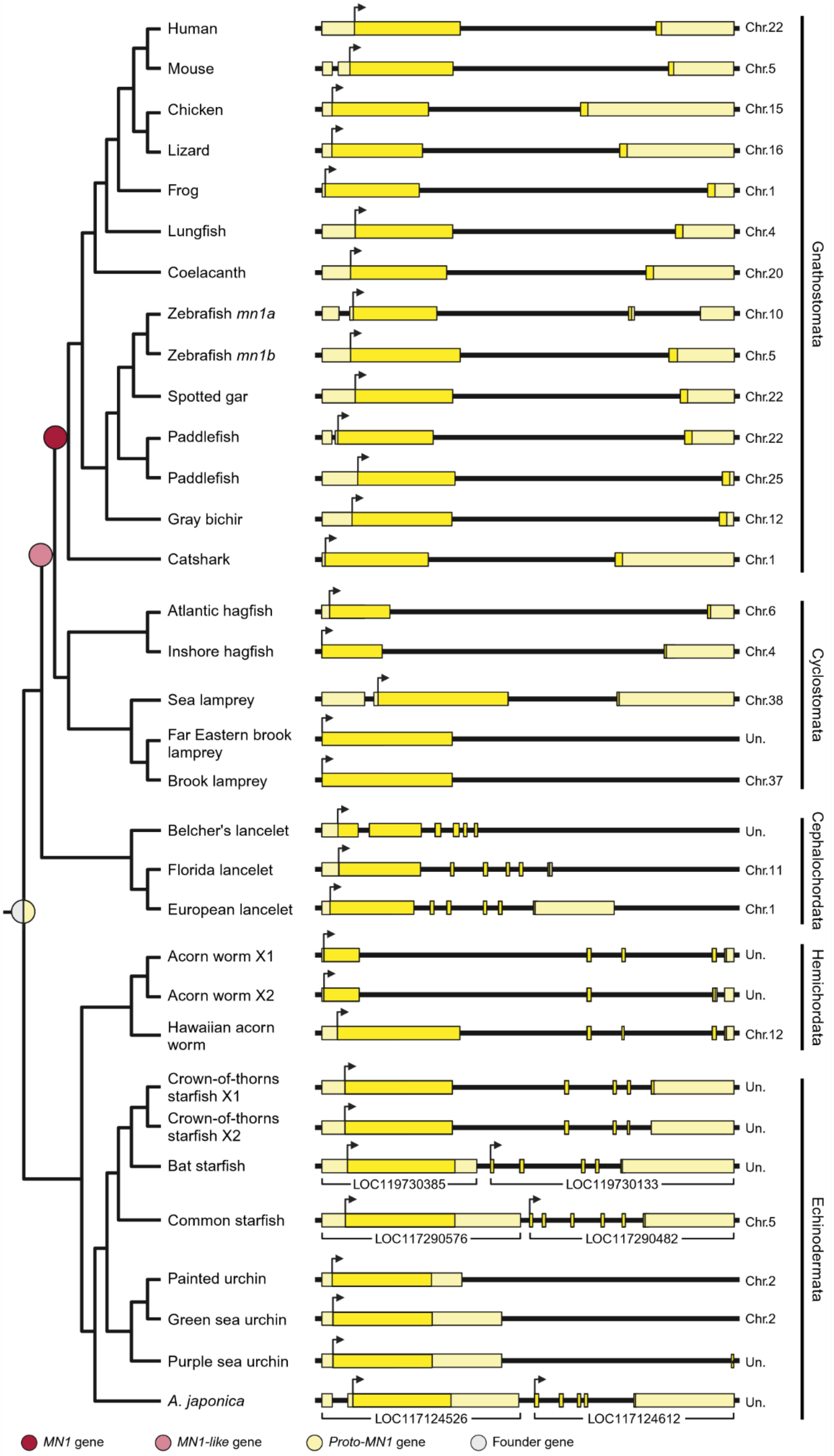

Human
Mouse
Chicken
Lizard
Frog
Lungfish
Coelacanth
Zebrafish *mn1a*
Zebrafish *mn1b*
Spotted gar
Paddlefish
Paddlefish
Gray bichir
Catshark
Atlantic hagfish
Inshore hagfish
Sea lamprey
Far Eastern brook lamprey
Brook lamprey
Belcher's lancelet
Florida lancelet
European lancelet
Acorn worm X1
Acorn worm X2
Hawaiian acorn worm
Crown-of-thorns starfish X1
Crown-of-thorns starfish X2
Bat starfish
Common starfish
Painted urchin
Green sea urchin
Purple sea urchin
A. japonica
Chr.22
Chr.5
Chr.15
Chr.16
Chr.1
Chr.4
Chr.20
Chr.10
Chr.5
Chr.22
Chr.22
Chr.25
Chr.12
Chr.1
Chr.6
Chr.4
Chr.38
Un.
Chr.37
Un.
Chr.11
Chr.1
Un.
Un.
Chr.12
Un.
Un.
Un.
Chr.5
Chr.2
Chr.2
Un.
Un.
LOC119730385
LOC119730133
LOC117290576
LOC117290482
LOC117124526
LOC117124612
Gnathostomata
Cyclostomata
Cephalochordata
Hemichordata
Echinodermata
MN1 gene
MN1-like gene
Proto-MN1 gene
Founder gene

Fig. S2: Gene structure analysis of the *MN1* gene.

Dendrogram indicating the relative evolutionary relationships between species and events related to *MN1* evolution in deuterostomes (left). X1 and X2 denote different isoforms of the same gene. *MN1* gene structure in the major deuterostome lineages (right). Vertebrate *MN1* genes comprise two coding exons separated by a large intron, the *proto-MN1* genes possess between 1-7 coding exons. Exon 1 of all *proto-MN1* genes resembles the large exon 1 found in vertebrate *MN1*. Yellow bars represent exons and black lines introns. UTR regions are coloured in light yellow. LOC119730385 and LOC119730133 (Bat starfish); LOC117290576 and LOC117290482 (Common starfish); LOC117124526 and LOC117124612 (*A. japonica*) are homologous to Crown-of-thorns starfish exons 1 and 2-5, respectively. The Far Eastern brook lamprey *MN1-like* gene is in a particularly short unlocalized scaffold, which might have isolated the second exon from the rest of the gene. Due to the large intronic lengths, only exons are drawn to scale. Un, unplaced scaffold.

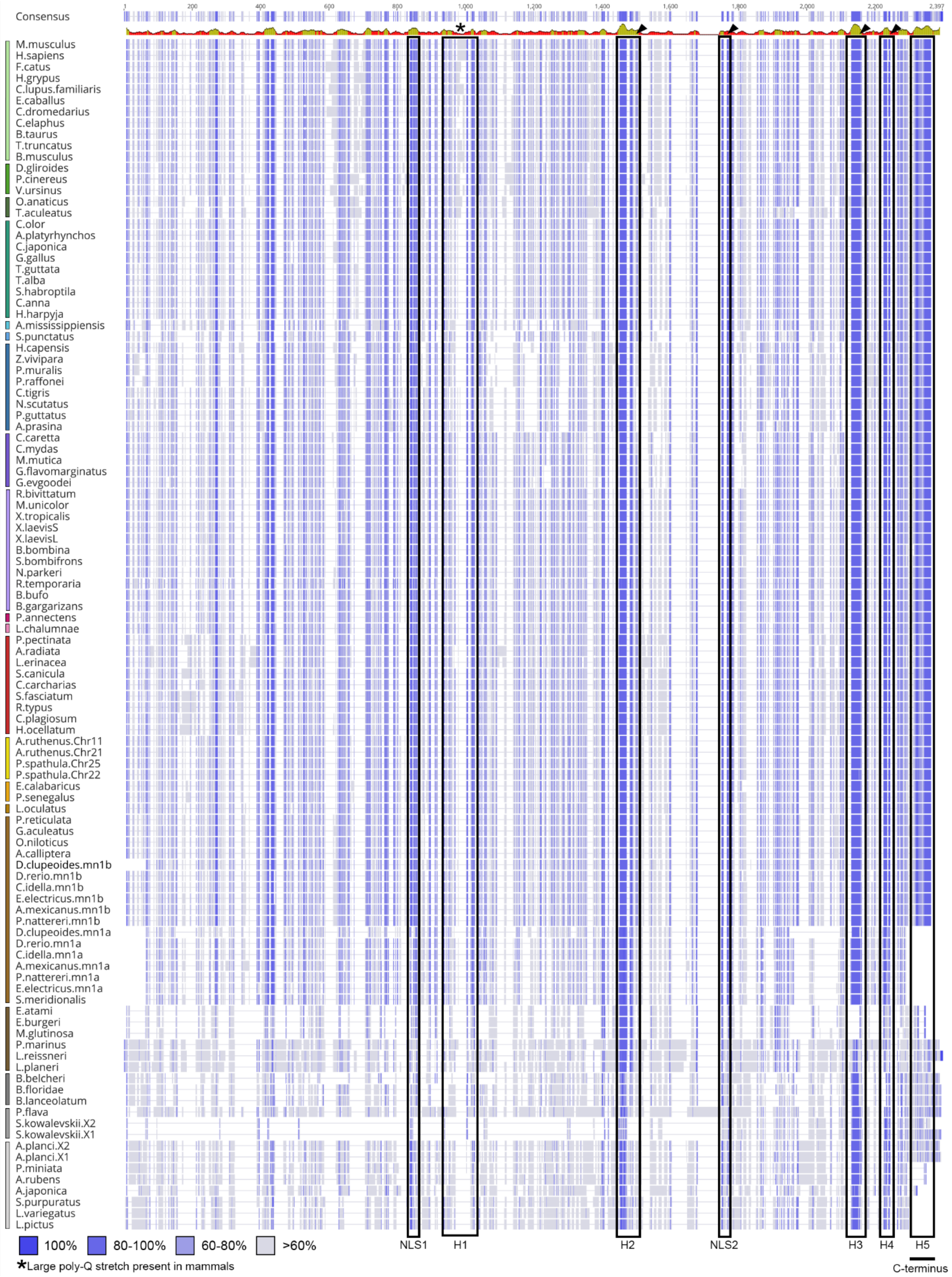
Consensus
1
200
400
600
800
1,000
1,200
1,400
1,600
1,800
2,000
2,200
2,397
M.musculus
H.sapiens
F.catus
H.grypus
C.lupus.familiaris
E.caballus
C.dromedarius
C.elaphus
B.taurus
T.truncatus
B.musculus
D.gliroides
P.cinereus
V.ursinus
O.anaticus
T.aculeatus
C.olor
A.platyrhynchos
C.japonica
G.gallus
T.guttata
T.alba
S.habroptila
C.anna
H.harpyja
A.mississippiensis
S.punctatus
H.capensis
Z.vivipara
P.muralis
P.raffonei
C.tigris
N.scutatus
P.guttatus
A.prasina
C.caretta
C.mydas
M.mutica
G.flavomarginatus
G.evgoodei
R.bivittatum
M.unicolor
X.tropicalis
X.laevisS
X.laevisL
B.bombina
S.bombifrons
N.parkeri
R.temporaria
B.bufo
B.gargarizans
P.annectens
L.chalumnae
P.pectinata
A.radiata
L.erinacea
S.canicula
C.carcharias
S.fasciatum
R.typus
C.plagiosum
H.ocellatum
A.ruthenus.Chr11
A.ruthenus.Chr21
P.spathula.Chr25
P.spathula.Chr22
E.calabaricus
P.senegalus
L.oculatus
P.reticulata
G.aculeatus
O.niloticus
A.calliptera
D.clupeoides.mn1b
D.rerio.mn1b
C.idella.mn1b
E.electricus.mn1b
A.mexicanus.mn1b
P.nattereri.mn1b
D.clupeoides.mn1a
D.rerio.mn1a
C.idella.mn1a
A.mexicanus.mn1a
P.nattereri.mn1a
E.electricus.mn1a
S.meridionalis
E.atami
E.burgeri
M.glutinosa
P.marinus
L.reissneri
L.planeri
B.belcheri
B.floridae
B.lanceolatum
P.flava
S.kowalevskii.X2
S.kowalevskii.X1
A.planci.X2
A.planci.X1
P.miniata
A.rubens
A.japonica
S.purpuratus
L.variegatus
L.pictus
100%
80-100%
60-80%
>60%
NLS1
H1
H2
NLS2
H3
H4
H5
*Large poly-Q stretch present in mammals
C-terminus

Fig. S3: Multiple sequence alignment scheme of the putative MN1 amino acid sequences.

Protein sequences are coloured based on the percentage of similarity per amino acid residue and the mean pairwise identity over all pairs in the column is shown as a histogram on top (green: 100%, brown: 99-30%, red: <30%). Thick lines on the left define major clades colour-coded following Fig. S1. Rectangles indicate highly conserved regions with nuclear localization signals (NLS1-2) or secondary helical structures predicted by AlphaFold (H1-5). Asterisk (*) indicates a large poly-Q stretch present in mammals. Arrowheads indicate the four shared conserved domains between vertebrates and invertebrates. Note the highly conserved C-terminus domain present in gnathostomes, where H5 is located.

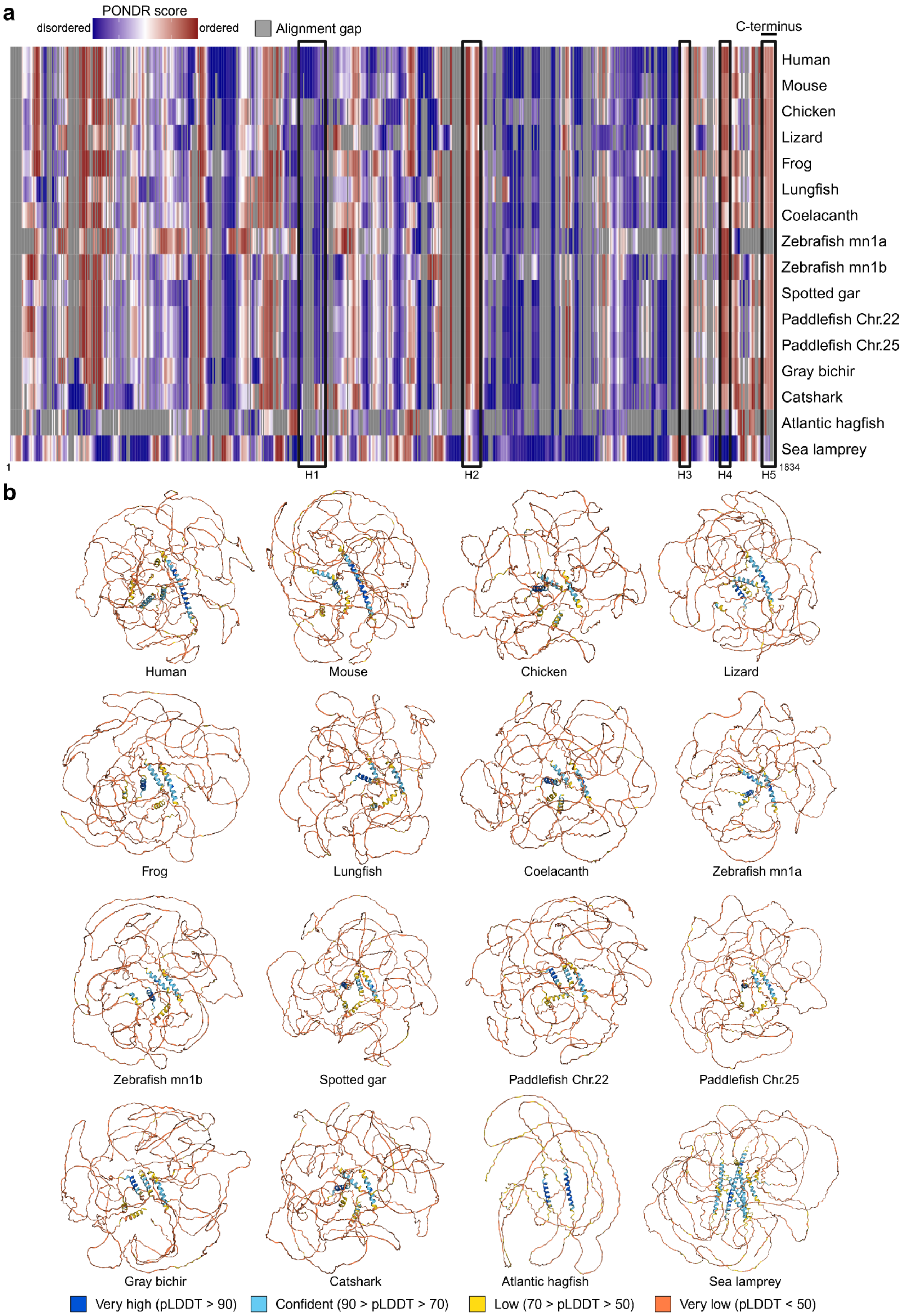

a
PONDR score
disordered
ordered
Alignment gap
C-terminus
Human
Mouse
Chicken
Lizard
Frog
Lungfish
Coelacanth
Zebrafish mn1a
Zebrafish mn1b
Spotted gar
Paddlefish Chr.22
Paddlefish Chr.25
Gray bichir
Catshark
Atlantic hagfish
Sea lamprey
1
1834
H1
H2
H3
H4
H5
b
Human
Mouse
Chicken
Lizard
Frog
Lungfish
Coelacanth
Zebrafish mn1a
Zebrafish mn1b
Spotted gar
Paddlefish Chr.22
Paddlefish Chr.25
Gray bichir
Catshark
Atlantic hagfish
Sea lamprey
Very high (pLDDT > 90)
Confident (90 > pLDDT > 70)
Low (70 > pLDDT > 50)
Very low (pLDDT < 50)

Fig. S4: Structural predictions of the vertebrate MN1 proteins.

a. PONDR structural predictions demonstrate the degree of disorder per amino acid residue of MN1 proteins. PONDR scores were plotted on a sequence alignment of the selected vertebrate species for clearer interpretation of the results. **b.** AlphaFold 3D protein predictions. Amino acids are coloured based on their confidence score (pLDDT), with higher values indicating robust predictions. All structures are oriented with the first helix (H1) positioned to the right of the helical core.

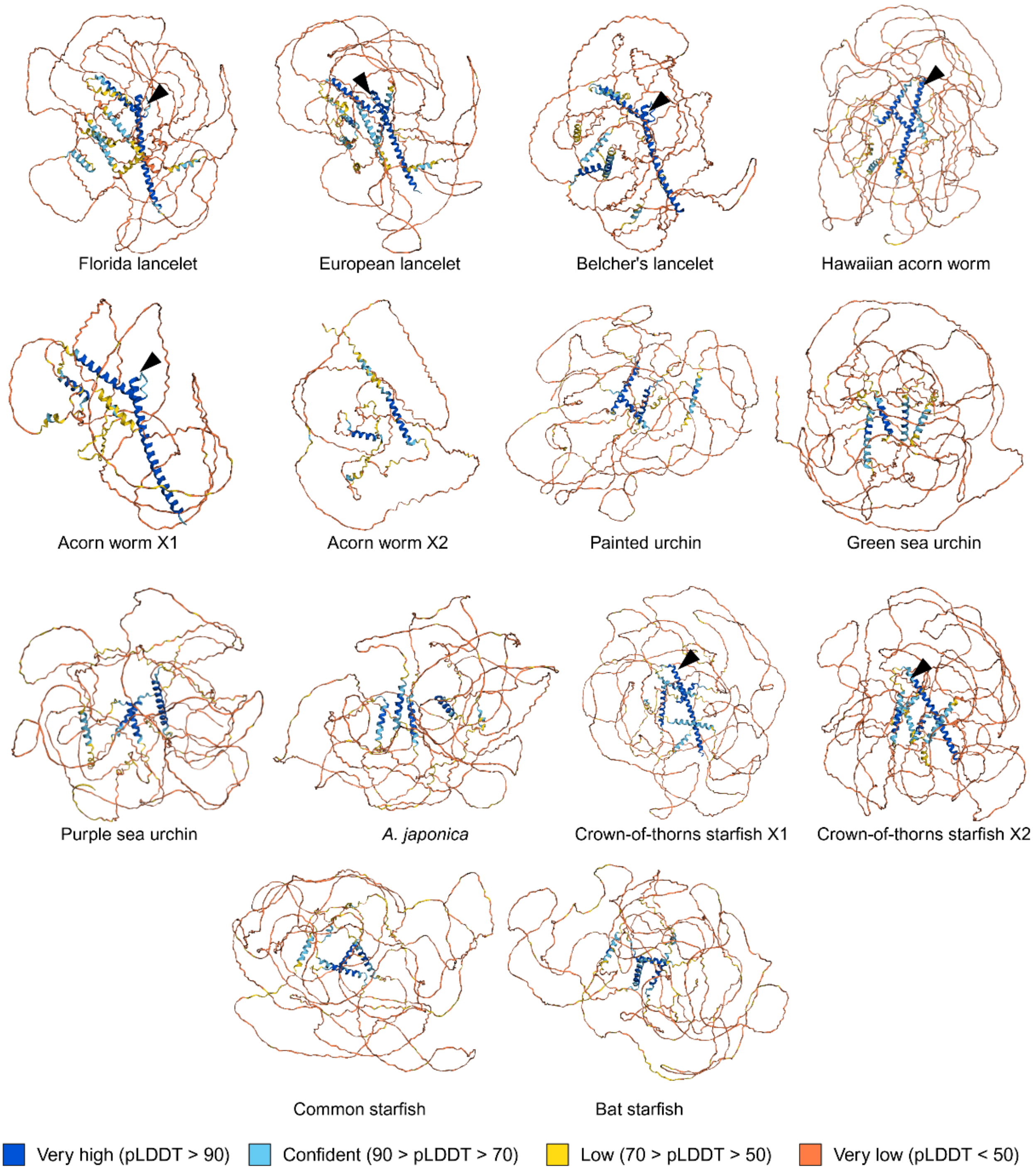

Florida lancelet
European lancelet
Belcher's lancelet
Hawaiian acorn worm
Acorn worm X1
Acorn worm X2
Painted urchin
Green sea urchin
Purple sea urchin
A. japonica
Crown-of-thorns starfish X1
Crown-of-thorns starfish X2
Common starfish
Bat starfish
Very high (pLDDT > 90)
Confident (90 > pLDDT > 70)
Low (70 > pLDDT > 50)
Very low (pLDDT < 50)

Fig. S5: Structural predictions of the invertebrate proto-MN1 proteins.

AlphaFold 3D protein predictions of the 14 invertebrate proto-MN1 proteins. Amino acids are coloured based on their confidence score (pLDDT), with higher values indicating robust predictions. Black arrowheads indicate a basic helix-loop-helix DNA-binding domain.

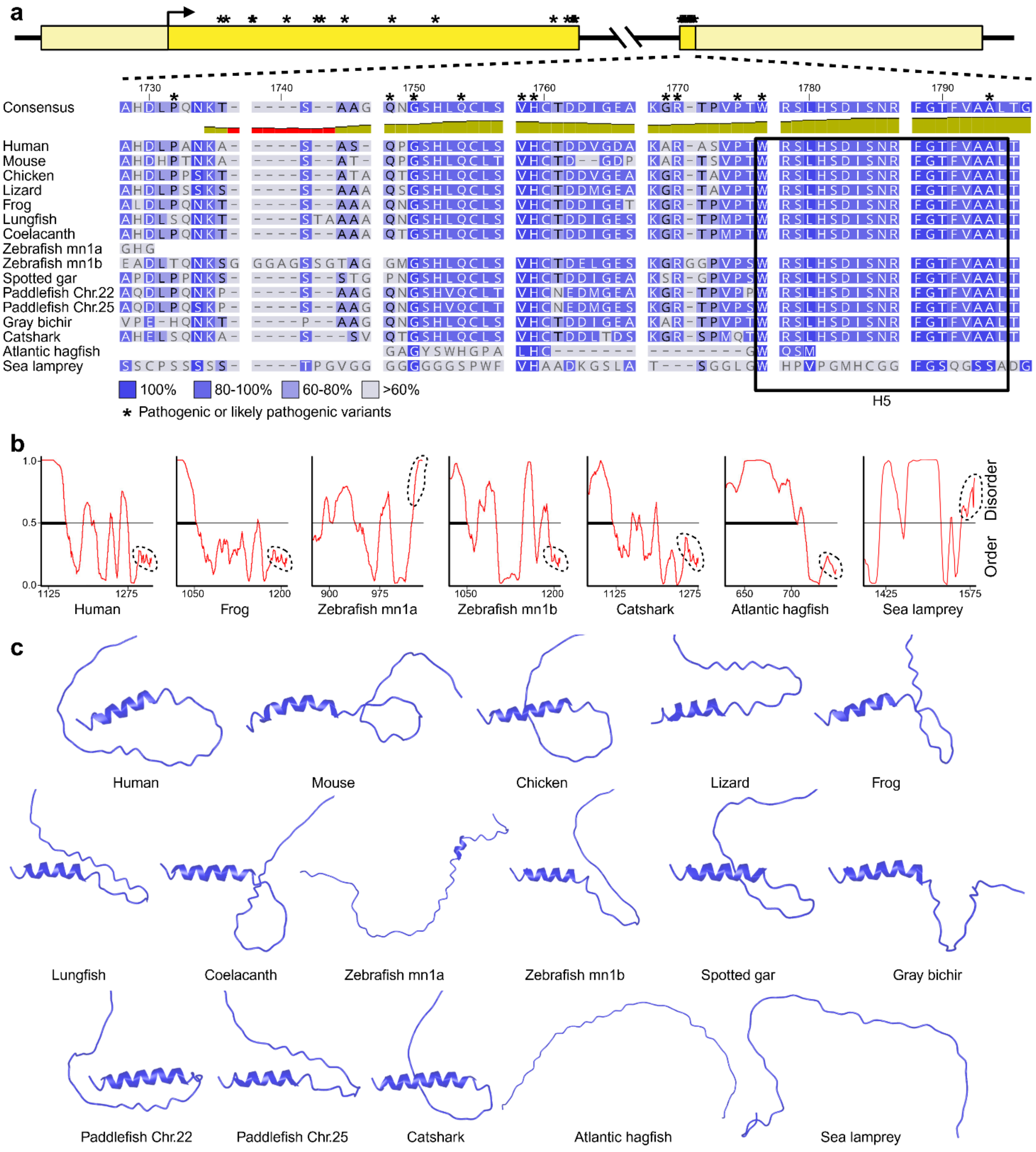
a
1730
1740
1750
1760
1770
1780
1790
Consensus
Human
Mouse
Chicken
Lizard
Frog
Lungfish
Coelacanth
Zebrafish mn1a
Zebrafish mn1b
Spotted gar
Paddlefish Chr.22
Paddlefish Chr.25
Gray bichir
Catshark
Atlantic hagfish
Sea lamprey
100%
80-100%
60-80%
>60%
H5
* Pathogenic or likely pathogenic variants
b
1.0
0.5
0.0
1125
1275
Human
1050
1200
Frog
900
975
Zebrafish mn1a
1050
1200
Zebrafish mn1b
1125
1275
Catshark
650
700
Atlantic hagfish
1425
1575
Sea lamprey
Disorder
Order
c
Human
Mouse
Chicken
Lizard
Frog
Lungfish
Coelacanth
Zebrafish mn1a
Zebrafish mn1b
Spotted gar
Gray bichir
Paddlefish Chr.22
Paddlefish Chr.25
Catshark
Atlantic hagfish
Sea lamprey

Fig. S6: Vertebrate MN1 C-terminus conservation and structural characterization.

a. Schematic representation of the human *MN1* gene (top) and vertebrate C-terminus sequence conservation (bottom). Asterisks denote the 26 identified pathogenic or likely pathogenic variants in the human *MN1* gene (gnomAD); 15 of these variants fall in exon 1 and 11 in the short exon 2 - 2 of these variants fall in the predicted helix 5 (H5). The multi-species sequence alignment shows the high degree of conservation of the gnathostome exon 2, absent in cyclostomes. Note that the zebrafish mn1a paralog lost most of exon 2 and represents a teleost-specific secondary lost (Fig. S3). **b.** PONDR score prediction of selected vertebrate species confirms the ordered nature of the C-terminus domain in gnathostomes, with the exception of the zebrafish mn1a which does not posses a homologous exon 2. Due to the short exon 2 in cyclostomes (5 amino acids in the hagfish and 23 in the lamprey) and zebrafish mn1a (3 amino acids), we used the last 60 amino acids for structural predictions, which mostly include the end of exon 1. **c.** AlphaFold 3D protein predictions of the C-terminal region. All structures are oriented with the last amino acid positioned to the right of the models. The short helix in the zebrafish mn1a does not represent H5 but rather H4 from exon 1. Note the absence of any structured domain in cyclostome species.

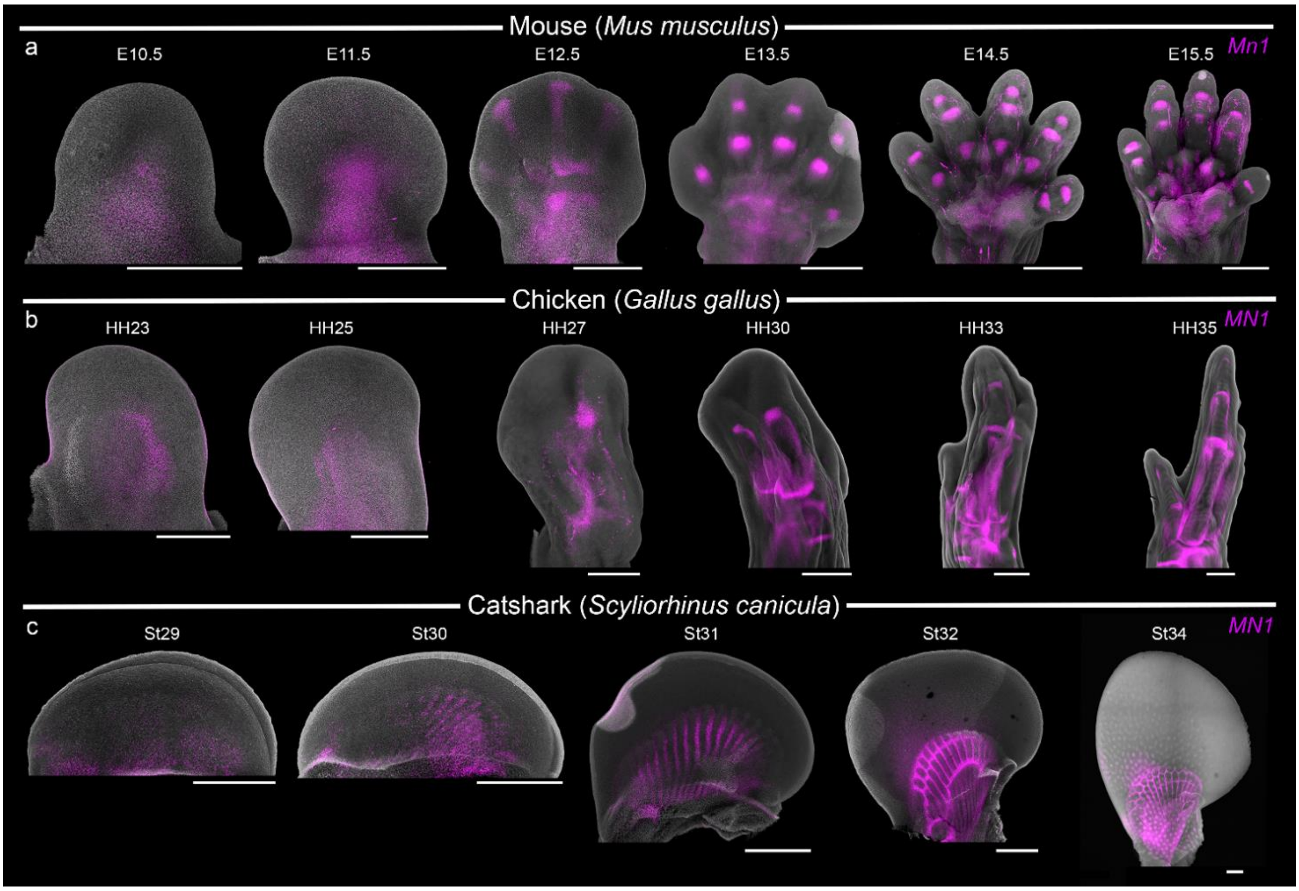

Mouse (Mus musculus)
a
E10.5
E11.5
E12.5
E13.5
E14.5
E15.5
Mn1
Chicken (Gallus gallus)
b
HH23
HH25
HH27
HH30
HH33
HH35
MN1
Catshark (Scyliorhinus canicula)
c
St29
St30
St31
St32
St34
MN1

Fig. S7: *MN1* expression during forelimb, wing and pectoral fin development in gnathostome species.

MN1 expression in developing mouse forelimb **(a)**, chicken wing **(b)** and small-spotted catshark pectoral fin **(c)**. Scale bars: 500um. At least 3 samples were assayed per species and stage.

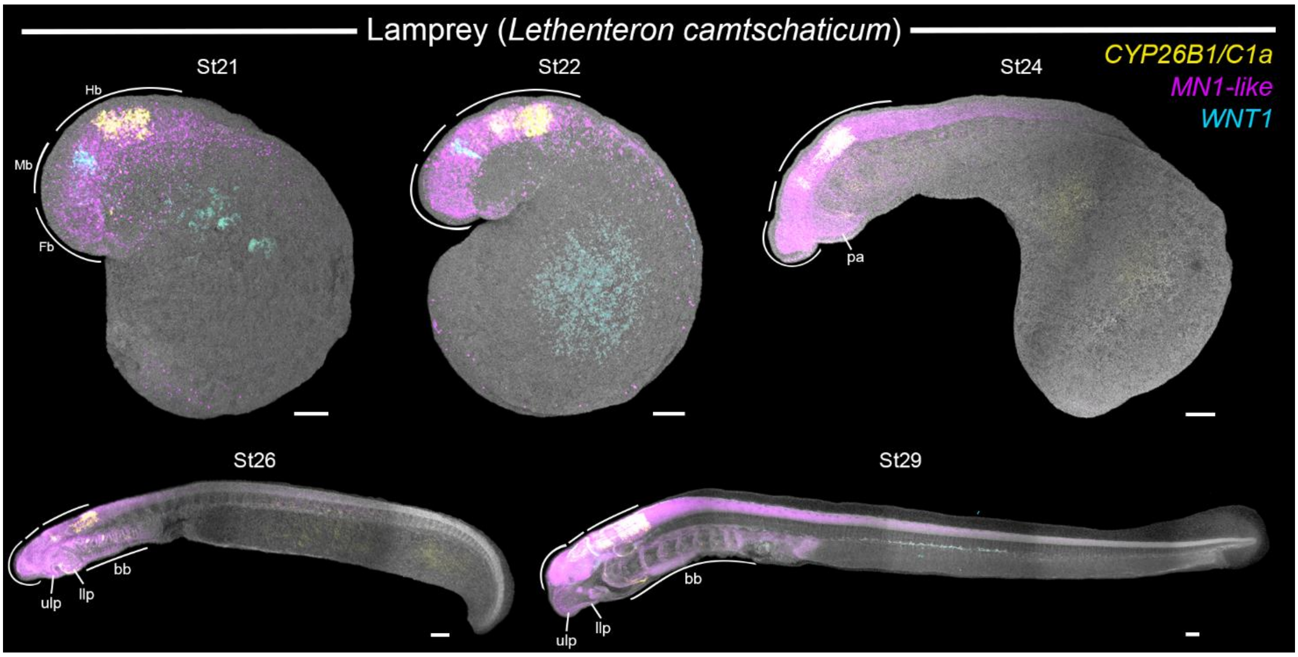
Lamprey (Lethenteron camtschaticum)
CYP26B1/C1a
MN1-like
WNT1
St21
St22
St24
St26
St29
Hb
Mb
Fb
pa
ulp
llp
bb

Fig. S8: *MN1-like* expression during arctic lamprey development.

Lateral view HCR images showing the expression of *MN1-like* (magenta), *CYP26B1/C1a* (yellow) and *WNT1* (cyan). Main brain regions are delineated by white lines, as well as key craniofacial structures. At least 3 embryos were assayed per developmental stage. bb, branchial basket; Fb, forebrain; Hb, hindbrain; llp, lower lip; Mb, midbrain; pa, first pharyngeal arch; St, stage; ulp, upper lip. Scale bars: 100um.

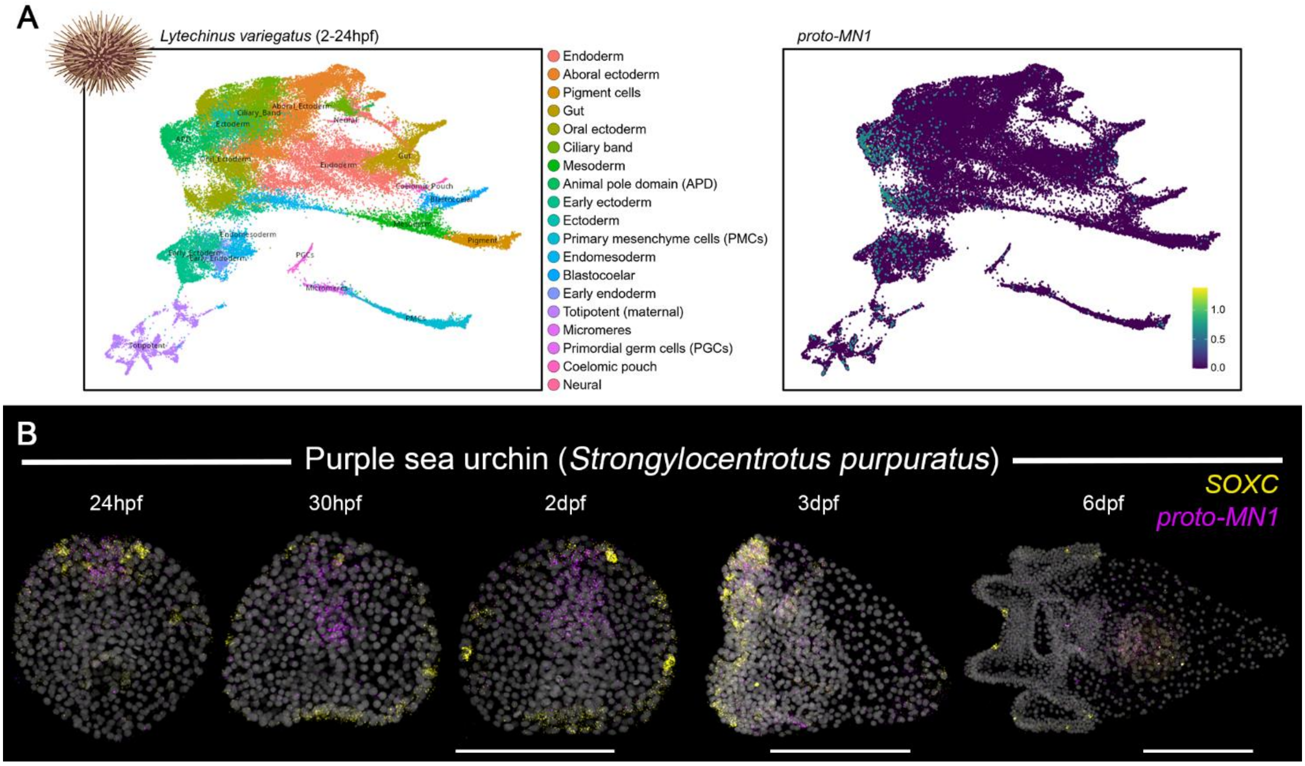
A
Lytechinus variegatus (2-24hpf)
Endoderm
Aboral ectoderm
Pigment cells
Gut
Oral ectoderm
Ciliary band
Mesoderm
Animal pole domain (APD)
Early ectoderm
Ectoderm
Primary mesenchyme cells (PMCs)
Endomesoderm
Blastocoelar
Early endoderm
Totipotent (maternal)
Micromeres
Primordial germ cells (PGCs)
Coelomic pouch
Neural
proto-MN1
1.0
0.5
0.0
B
Purple sea urchin (Strongylocentrotus purpuratus)
24hpf
30hpf
2dpf
3dpf
6dpf
SOXC
proto-MN1

Fig. S9: *proto-MN1* expression during sea urchin development.

a. Uniform Manifold Approximation and Projection (UMAP) plot of the green sea urchin (*L. variegatus*) single-cell RNAseq developmental atlas over the first 24 hours of embryonic development (1); cells are coloured by their cell-type annotation (left). Feature plot showing the expression of *proto-MN1* (right), primarily expressed in the animal pole domain, oral ectoderm and early ectoderm. **b.** HCR images showing the expression of *proto-MN1* (magenta) and a neural marker, *SOXC* (yellow) (2, 3), during purple sea urchin (*S. purpuratus*) development. From 1-3dpf embryos are oriented with the animal pole domain on top. The first 3 days of *S. purpuratus* development are equivalent to the first 24 hours in *L. variegatus*. At least 5 embryos were assayed per developmental stage. dpf, days post-fertilization; hpf, hours post-fertilization. Scale bars: 100um.

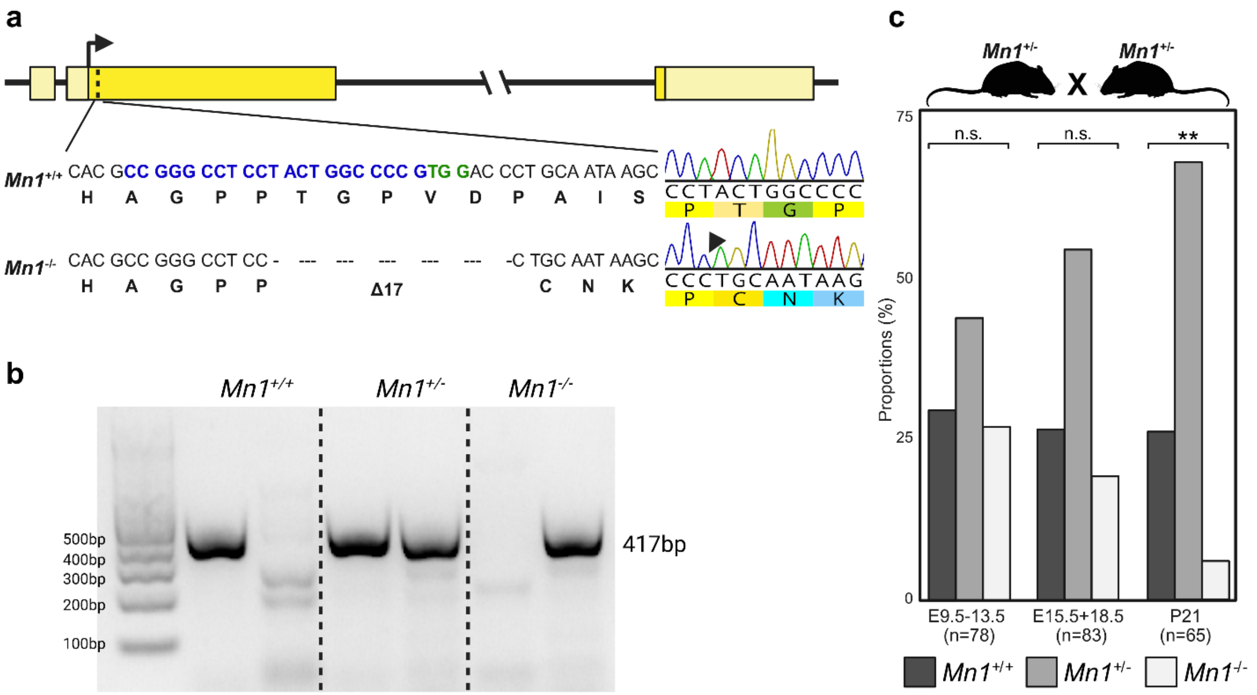
a
Mn1+/+ CAC GCC GGG CCT CCT ACT GGC CCC GTG GAC CCT GCA ATA AGC
H A G P P T G P V D P A I S
Mn1-/- CAC GCC GGG CCT CC - --- --- --- --- --- -C TGC AAT AAGC
H A G P P Δ17 C N K
CCTACTGGCCCC
P T G P
CCCTGCAATAAG
P C N K
b
Mn1+/+
Mn1+/-
Mn1-/-
500bp
400bp
300bp
200bp
100bp
417bp
c
Mn1+/- X Mn1+/-
n.s.
n.s.
**
Proportions (%)
0
25
50
75
E9.5-13.5 (n=78)
E15.5+18.5 (n=83)
P21 (n=65)
Mn1+/+
Mn1+/-
Mn1-/-

Fig. S10: Generation of the new *Mn1* mutant mouse line.

a. Schematic representation of the CRISPR/Cas9 strategy to generate the *Mn1*$^{-/-}$ line. Exons are indicated with yellow boxes and introns by black lines. UTR regions are coloured in light yellow. Amino acid and nucleotide sequences of *Mn1*$^{+/+}$ and *Mn1*$^{-/-}$ alleles are shown. Single guide RNA (sgRNA) targeting site is indicated by a dashed line and binding site marked in blue, with the protospacer adjacent motif sequence in green. Sanger sequencing results are shown with an arrowhead indicating the mutated site. **b.** Representative genotyping result for a *Mn1*$^{+/-}$ mating. *Mn1*$^{+/+}$ allele PCR in the right wells and *Mn1*$^{-/-}$ allele PCR in the left ones. Detailed information on the genotyping strategy can be found in Supplementary Methods. **c.** Mendelian proportions from *Mn1*$^{+/-}$ intercrosses. 10 liters per gestational period or postpartum timepoint were taken for analysis. P21 pups represent those who survived to weaning age at five weeks post birth. The segregation ratios were analyzed by chi-square test against the expected Mendelian ratios of 1:2:1. *$p \leq 0.05$, **$p \leq 0.01$, ***$p \leq 0.001$. n.s., not significant.

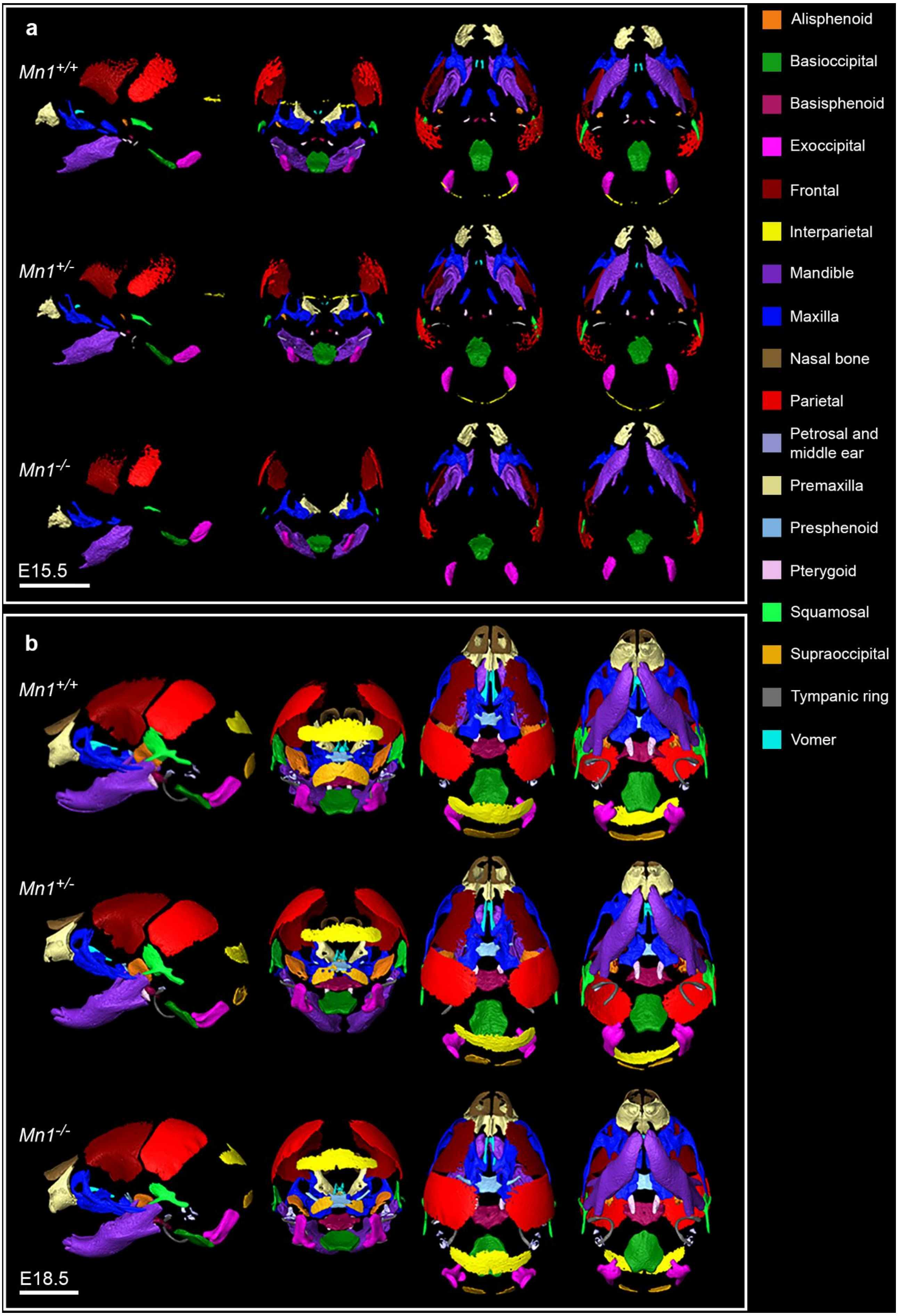
a
Mn1+/+
Mn1+/-
Mn1-/-
E15.5
b
Mn1+/+
Mn1+/-
Mn1-/-
E18.5
Alisphenoid
Basioccipital
Basisphenoid
Exoccipital
Frontal
Interparietal
Mandible
Maxilla
Nasal bone
Parietal
Petrosal and middle ear
Premaxilla
Presphenoid
Pterygoid
Squamosal
Supraoccipital
Tympanic ring
Vomer

Fig. S11: Skull phenotype in the newly generated *Mn1* mutant line.

µCT-based 3D reconstructions of mouse developing skulls at E15.5 **(a)** and E18.5 **(b)**. The following projections are shown from left to right: lateral, posterior, top and ventral. *Mn1*$^{+/+}$ (wild-type); *Mn1*$^{+/-}$(heterozygous); *Mn1*$^{-/-}$ (knock-out, mutant). Scale bars: 1mm.

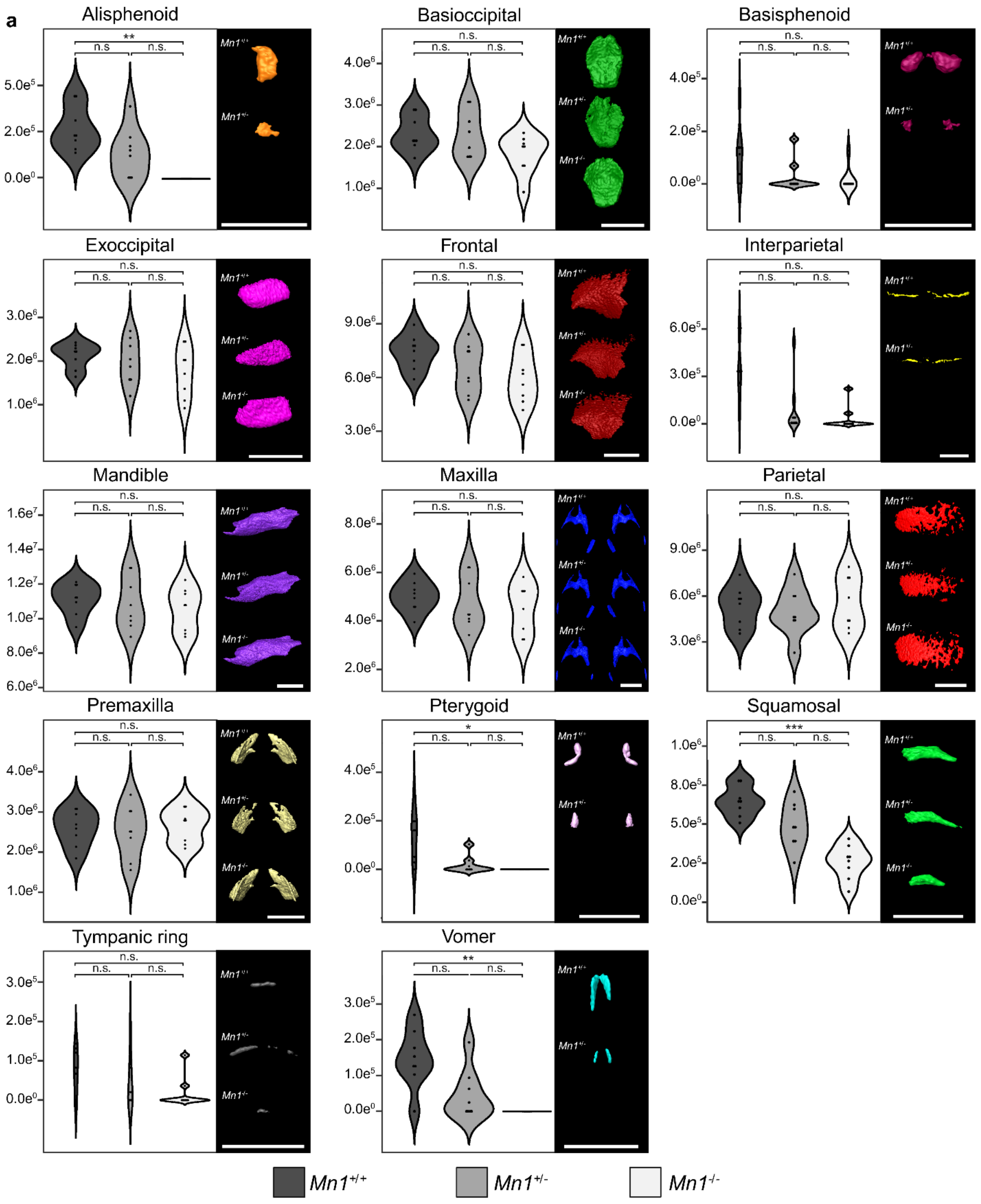
a
Alisphenoid
Basioccipital
Basisphenoid
Exoccipital
Frontal
Interparietal
Mandible
Maxilla
Parietal
Premaxilla
Pterygoid
Squamosal
Tympanic ring
Vomer
Mn1+/+
Mn1+/-
Mn1-/-

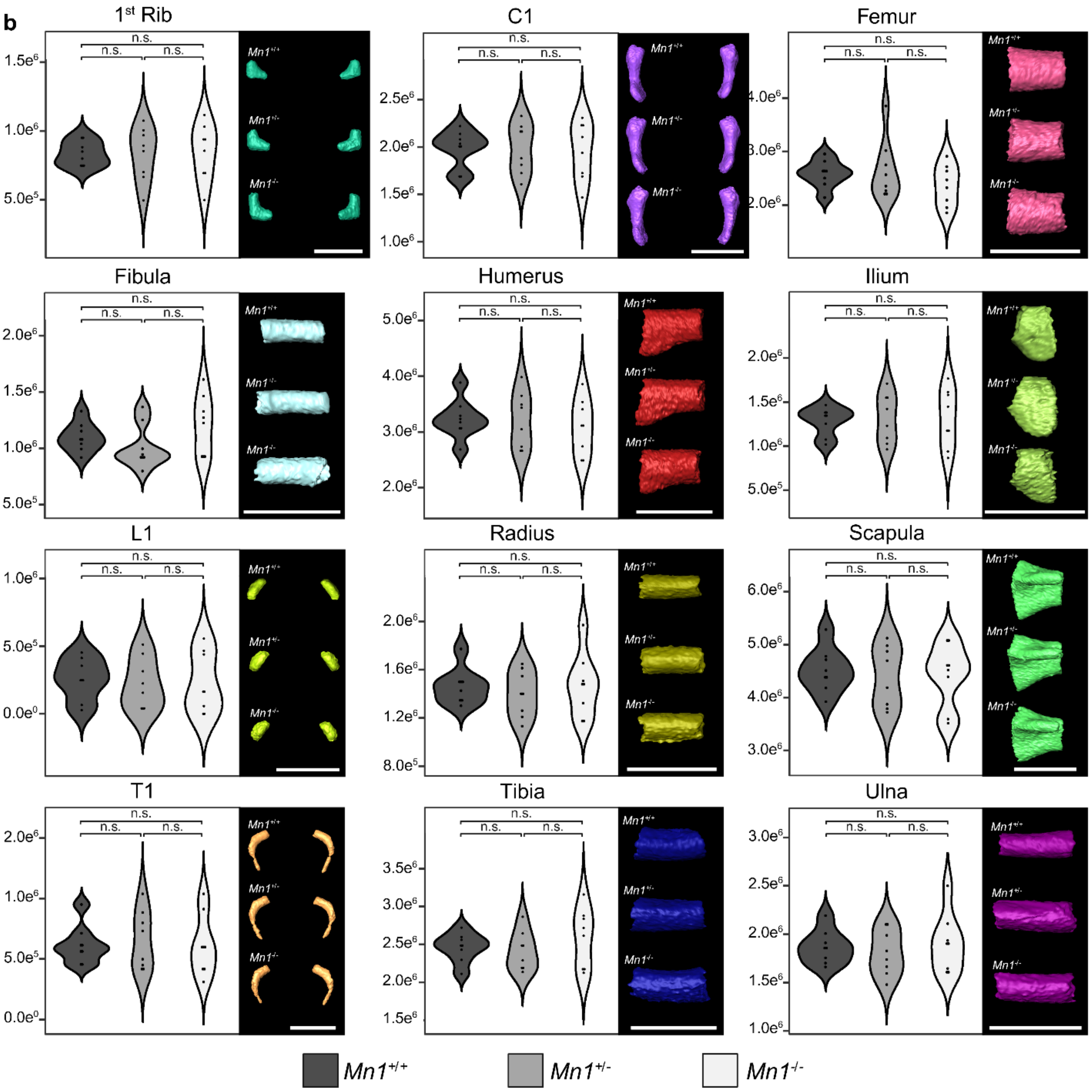

b
1st Rib
C1
Femur
Fibula
Humerus
Ilium
L1
Radius
Scapula
T1
Tibia
Ulna
n.s.
Mn1+/+
Mn1+/-
Mn1-/-

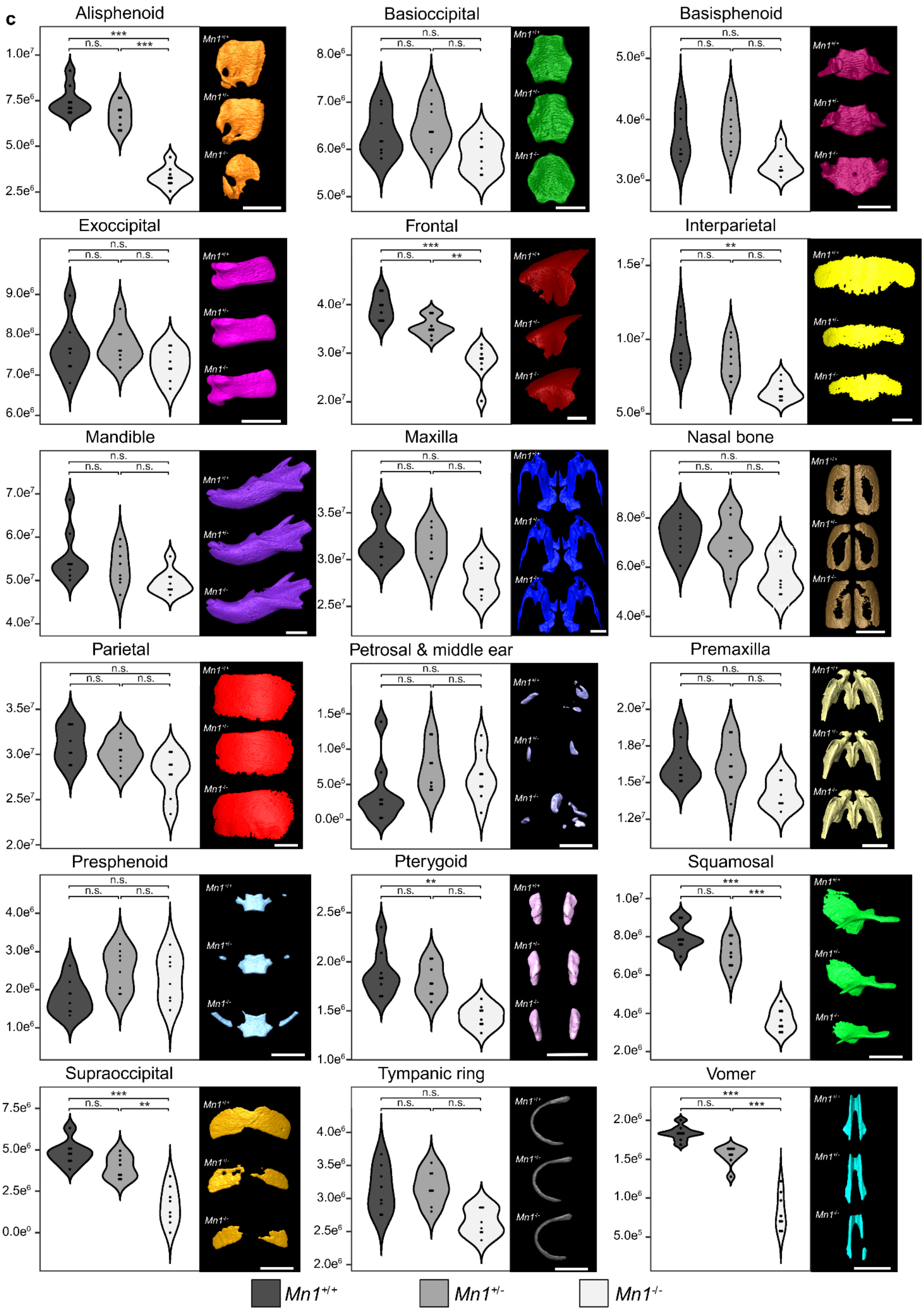
c
Alisphenoid
Basioccipital
Basisphenoid
Exoccipital
Frontal
Interparietal
Mandible
Maxilla
Nasal bone
Parietal
Petrosal & middle ear
Premaxilla
Presphenoid
Pterygoid
Squamosal
Supraoccipital
Tympanic ring
Vomer
n.s.
***
**
Mn1+/+
Mn1+/-
Mn1-/-

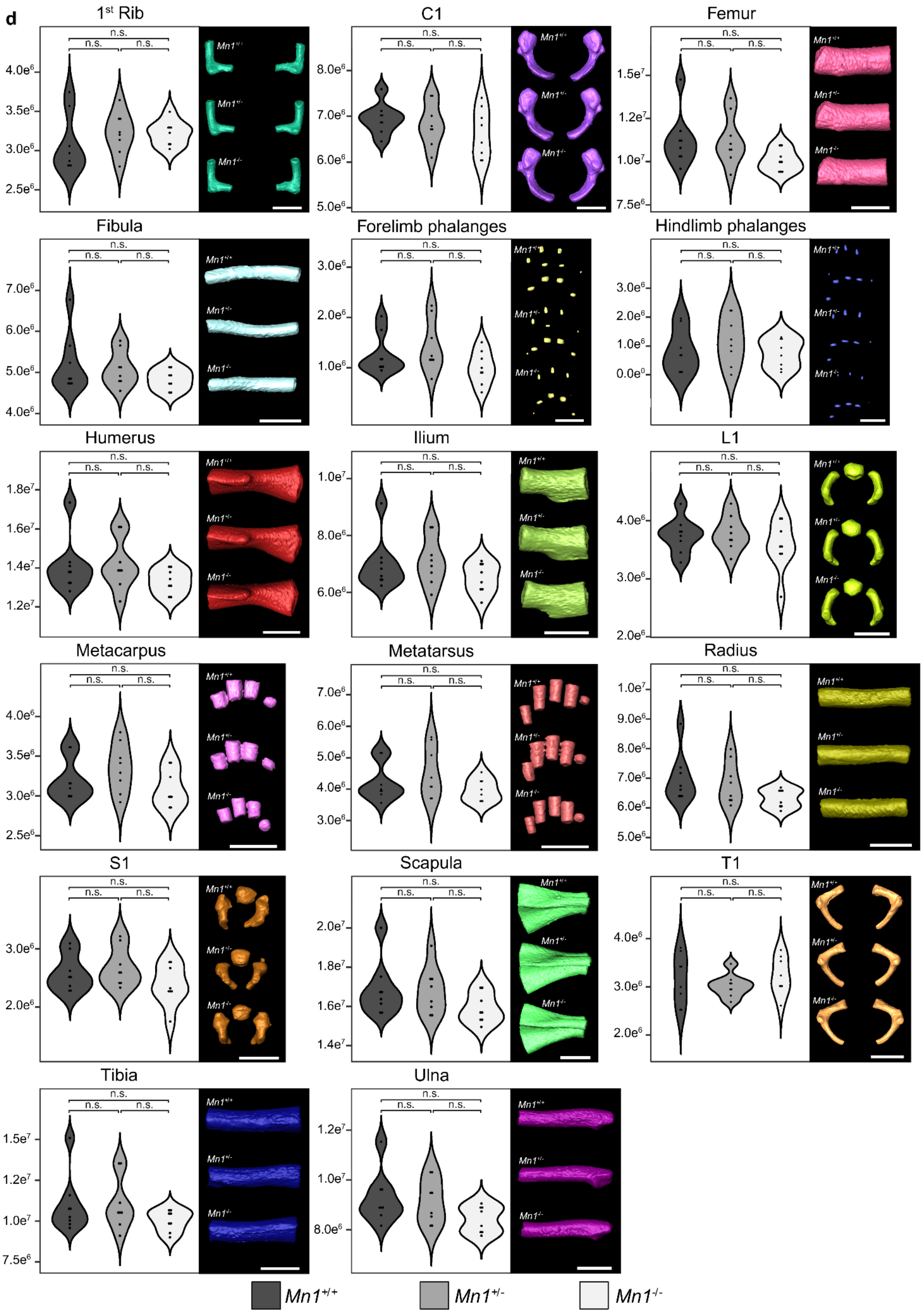
d
1st Rib
n.s.
n.s. n.s.
4.0e6 3.5e6 3.0e6 2.5e6
C1
8.0e6 7.0e6 6.0e6 5.0e6
Femur
1.5e7 1.2e7 1.0e7 7.5e6
Fibula
7.0e6 6.0e6 5.0e6 4.0e6
Forelimb phalanges
3.0e6 2.0e6 1.0e6
Hindlimb phalanges
3.0e6 2.0e6 1.0e6 0.0e0
Humerus
1.8e7 1.6e7 1.4e7 1.2e7
Ilium
1.0e7 8.0e6 6.0e6
L1
4.0e6 3.0e6 2.0e6
Metacarpus
4.0e6 3.5e6 3.0e6 2.5e6
Metatarsus
7.0e6 6.0e6 5.0e6 4.0e6 3.0e6
Radius
1.0e7 9.0e6 8.0e6 7.0e6 6.0e6 5.0e6
S1
3.0e6 2.0e6
Scapula
2.0e7 1.8e7 1.6e7 1.4e7
T1
4.0e6 3.0e6 2.0e6
Tibia
1.5e7 1.2e7 1.0e7 7.5e6
Ulna
1.2e7 1.0e7 8.0e6
Mn1+/+
Mn1+/-
Mn1-/-

Fig. S12: µCT analysis of the new *Mn1* mutant line at E15.5 and E18.5.

3D reconstruction of individual skull bones **(a)** and appendicular skeleton **(b)** at E15.5 and E18.5 **(c, d)**. Violin plots represent surface area in μm^2. Statistical significance was calculated by one-way ANOVA with Tukey's multiple comparison test and adjusted for multiple testing by Bonferroni correction. Scale bars: 1mm.

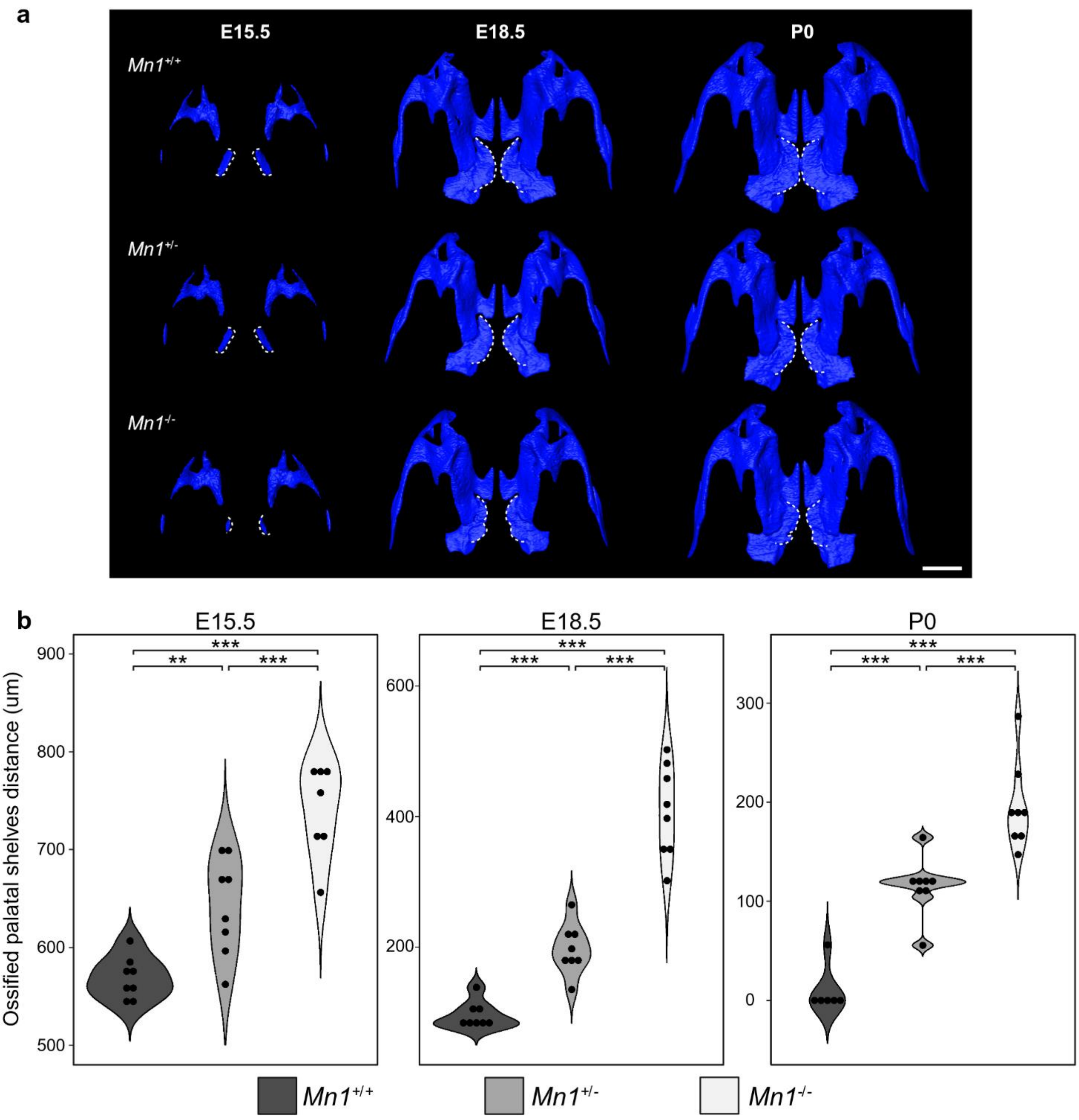
a
E15.5
E18.5
P0
Mn1+/+
Mn1+/-
Mn1-/-
b
E15.5
E18.5
P0
Ossified palatal shelves distance (um)
900
800
700
600
500
**
***
600
400
200
300
200
100
0
Mn1+/+
Mn1+/-
Mn1-/-

Fig. S13: Palate abnormalities in the newly generated *Mn1* mutant line.

a. Delayed ossification and malformations of the palatine bone. The palatine, segmented together with the maxilla, is surrounded by a dashed white line. Note in the *Mn1*$^{-/-}$ the smaller size at E15.5 denoting a delayed ossification and the abnormal shape at E18.5 and P0, with failure to fuse by P0. Scale bar: 1mm. **b.** Progression of the ossification in the palatine. The distance between the ossified palatal shelves at their closest point was measured and shows a statistically significant difference, with fusion failure in the *Mn1*$^{-/-}$. Distances were analyzed by one-way ANOVA with Tukey's multiple comparison test. *$p \leq 0.05$, **$p \leq 0.01$, ***$p \leq 0.001$. n.s., not significant.

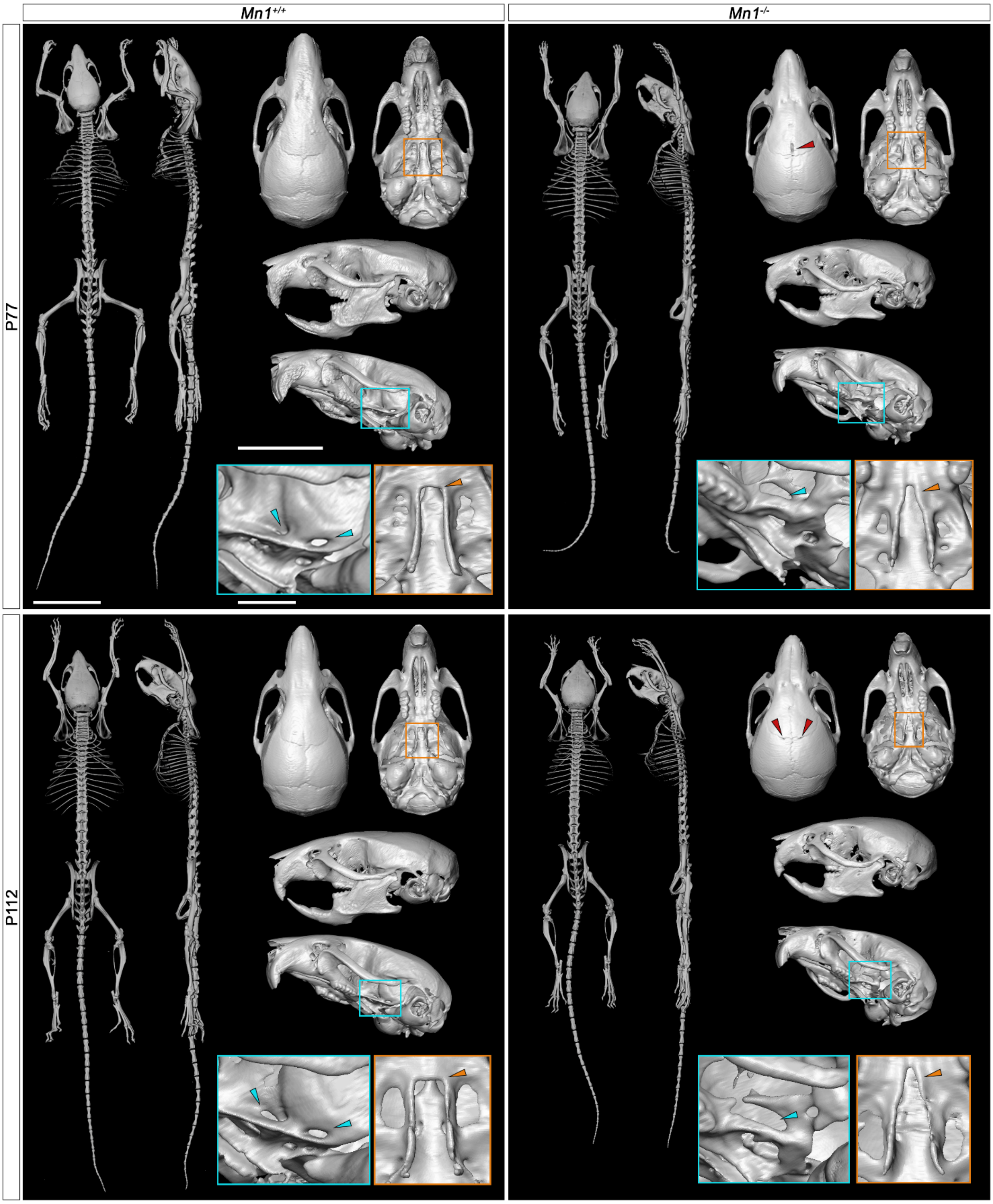

$Mn1^{+/+}$
$Mn1^{-/-}$
P77
P112

Fig. S14: Skeletal phenotypes in adult *Mn1* mutant mice.

µCT-based 3D reconstructions of representative adult *Mn1*$^{+/+}$ and *Mn1*$^{-/-}$ mice at 11 weeks (P77, males) and 16 weeks (P112, females) old. Cyan insets represent magnifying areas of the alisphenoid bone; cyan arrowheads indicate the foramen ovale and foramen rotundum. Note the enlarged foramens or failure to establish a clear difference in between them in the *Mn1*$^{-/-}$ mice. Orange insets represent magnifying areas of the palate; orange arrowheads indicate the posterior palate. Note the different shape of the posterior palate in the *Mn1*$^{-/-}$ mice. Red arrowheads indicate suture failures. Note the smaller size of the *Mn1*$^{-/-}$ mice compared to their wild-type counterparts. Adult *Mn1* mutant mice are quite exceptional and, hence, no statistical analysis could be performed. Scale bars: 2cm (whole mouse), 1cm (skulls), 2mm (insets).

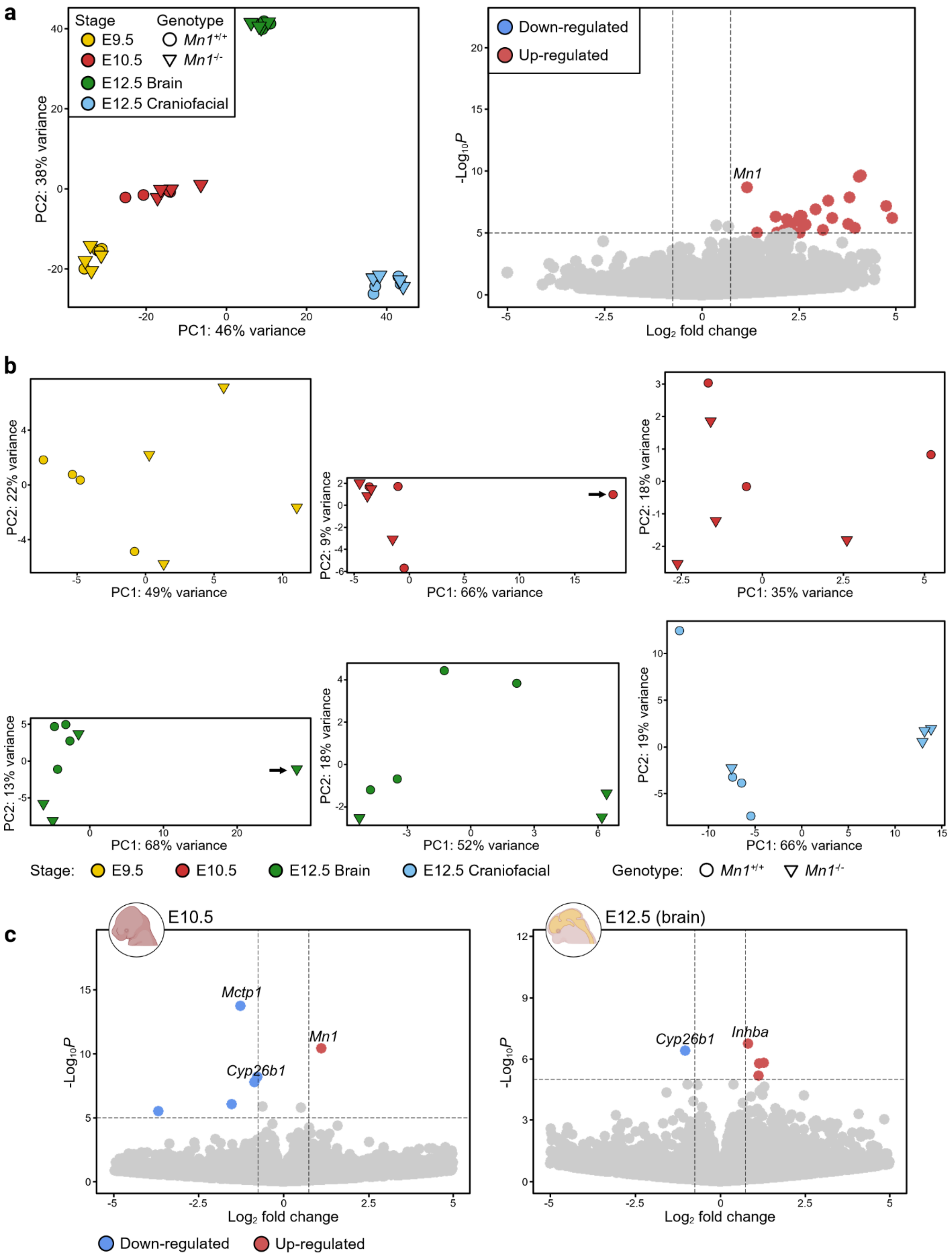
a
Stage
Genotype
E9.5
E10.5
E12.5 Brain
E12.5 Craniofacial
Mn1+/+
Mn1-/-
PC1: 46% variance
PC2: 38% variance
Down-regulated
Up-regulated
Mn1
Log2 fold change
-Log10P
b
PC1: 49% variance
PC2: 22% variance
PC1: 66% variance
PC2: 9% variance
PC1: 35% variance
PC2: 18% variance
PC1: 68% variance
PC2: 13% variance
PC1: 52% variance
PC2: 18% variance
PC1: 66% variance
PC2: 19% variance
Stage: E9.5 E10.5 E12.5 Brain E12.5 Craniofacial
Genotype: Mn1+/+ Mn1-/-
c
E10.5
Mctp1
Mn1
Cyp26b1
E12.5 (brain)
Cyp26b1
Inhba
Log2 fold change
-Log10P
Down-regulated
Up-regulated

Fig. S15: Additional information of the RNA-seq analysis of *Mn1* mutant embryos.

a. Principal component analysis (PCA) and volcano plot of all samples combined. Note that samples cluster based on their embryonic stage and not their genotype. Comparison of *Mn1*$^{+/+}$ with *Mn1*$^{-/-}$ embryos irrespective of their embryonic stage shows *Mn1* as the only differentially expressed gene across all stages. **b.** PCAs by embryonic stage to remove the effect of embryonic development progression. Arrows indicate outliers, which were removed from subsequent bioinformatics analyses. **c.** Volcano plots of E10.5 (whole heads) and E12.5 brains including the outliers. Note that removing (Fig. 3b) or including the outliers show comparable results.

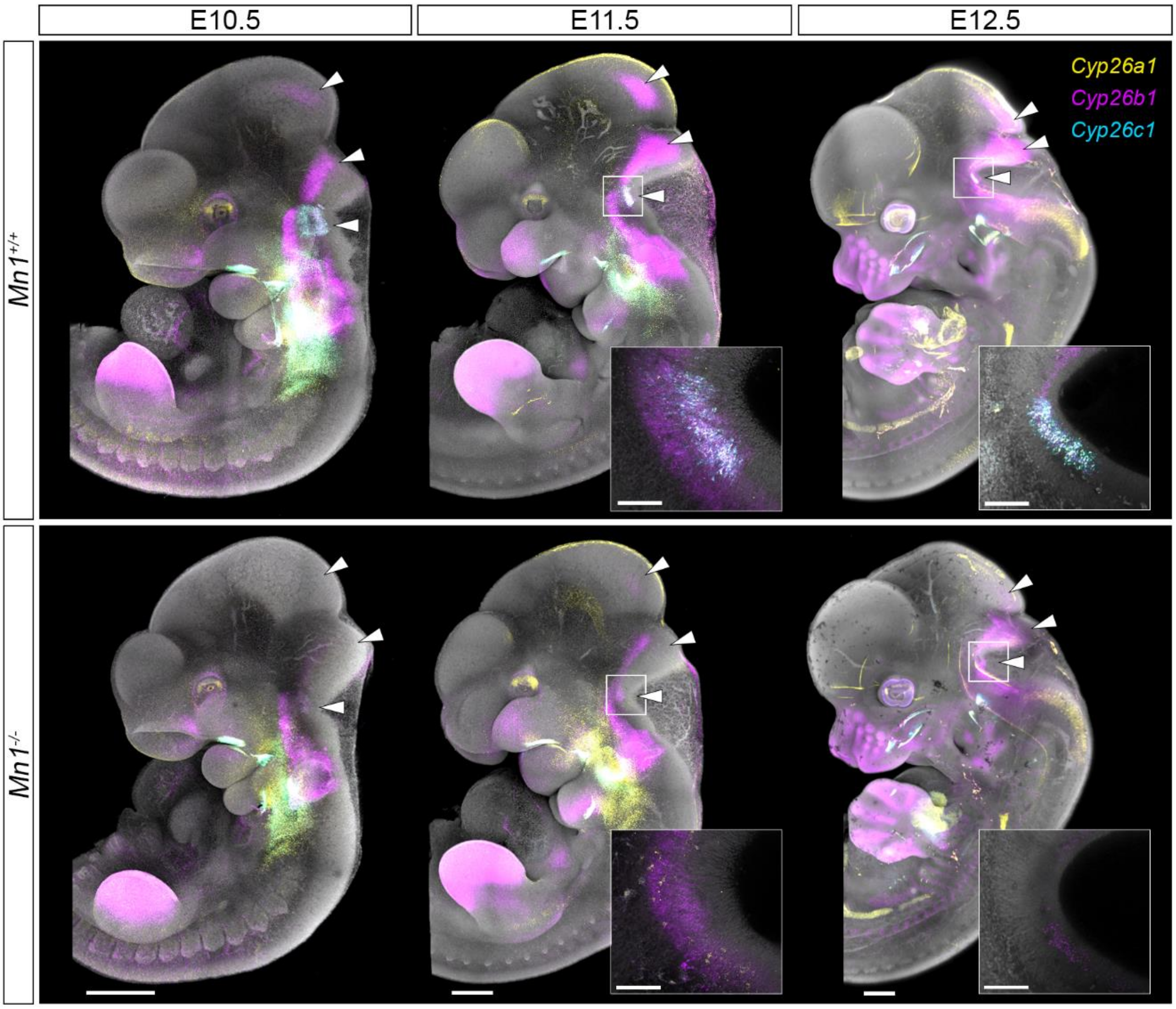
E10.5
E11.5
E12.5
Mn1+/+
Mn1-/-
Cyp26a1
Cyp26b1
Cyp26c1

Fig. S16: *Cyp26* expression is downregulated in E10.5-E12.5 *Mn1* mutants.

Lateral view HCR images of *Cyp26* genes expression at E10.5, E11.5 and E12.5 *Mn1* mutant and WT embryos. White arrowheads indicate main differences. Note the absent *Cyp26b1* expression in the midbrain in the *Mn1* mutants. Scale bars: 500um. Insets represent magnified area of the pontine flexure at E11.5 and E12.5 highlighting differences in *Cyp26a1,c1* expression the in r1 segment. Scale bars: 100um. At least 3 embryos were assayed per genotype and developmental stage.

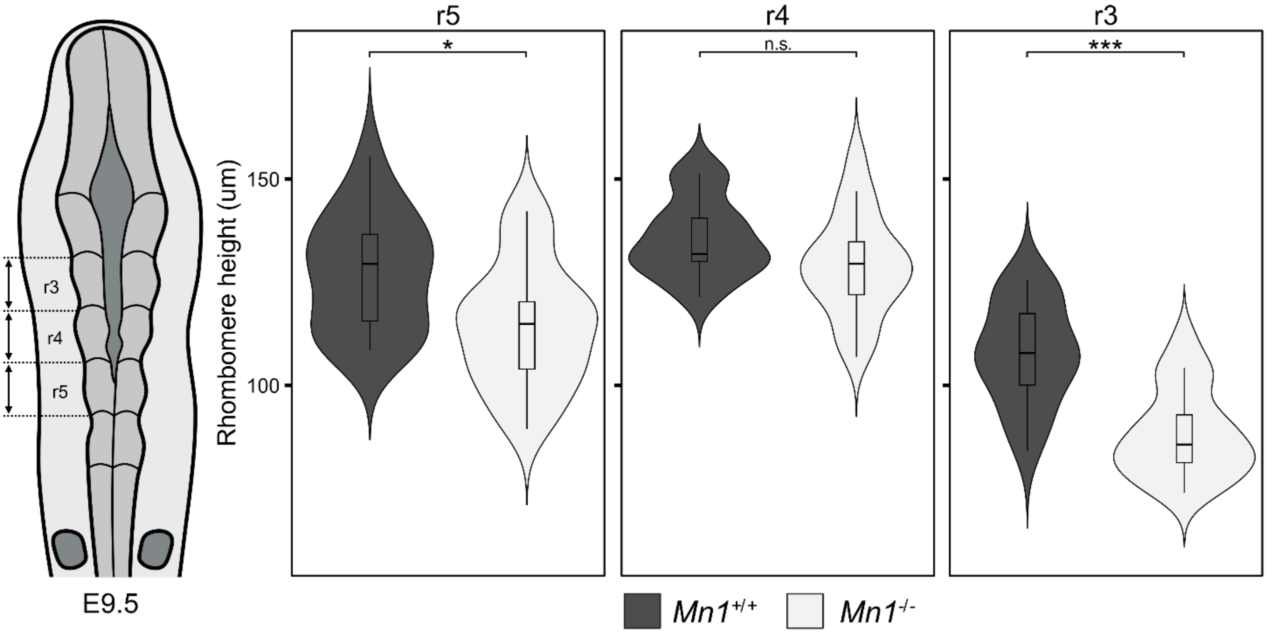
r3
r4
r5
E9.5
r5
r4
r3
*
n.s.
***
Rhombomere height (um)
150
100
Mn1+/+
Mn1-/-

Fig. S17: Differences in rhombomere height in *Mn1* mutant E9.5 embryos.

Rhombomeres r3-r5 were selected for measurements since their corresponding segment boundaries can be clearly distinguished in DAPI stained samples. We measured the height of the rhombomeres in all embryos used for 3D confocal microscopy (n=17 $Mn1^{+/+}$ and n=18 $Mn1^{-/-}$) as indicated in the scheme (left). Statistical significance was calculated by unpaired two-samples t-test. *p ≤ 0.05, **p ≤ 0.01, ***p ≤ 0.001. n.s., not significant.

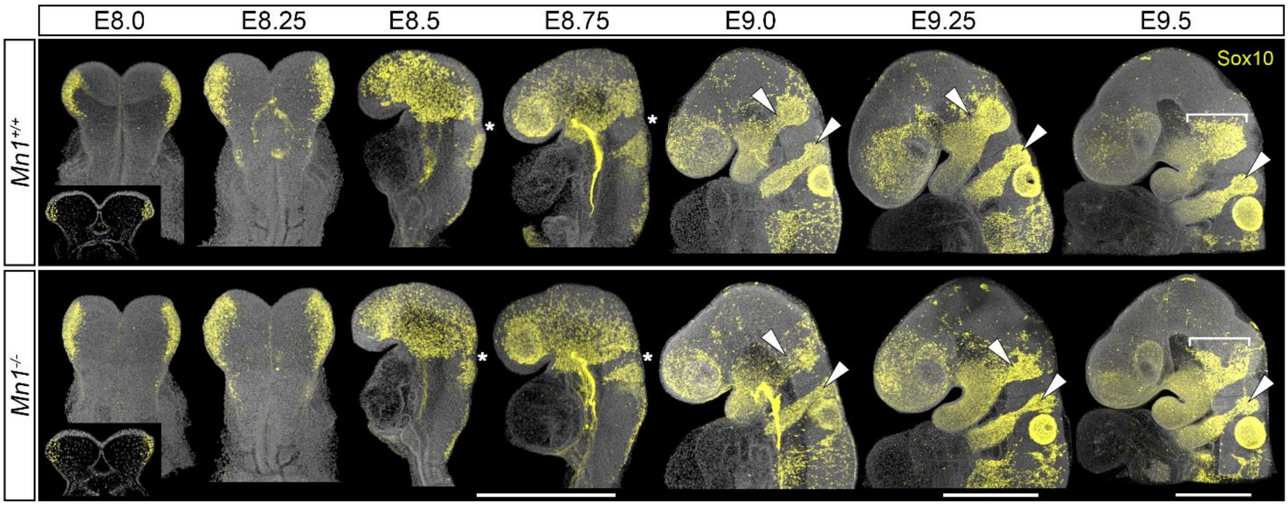
E8.0
E8.25
E8.5
E8.75
E9.0
E9.25
E9.5
Sox10
Mn1+/+
Mn1-/-

Fig. S18: Differences in cranial neural crest cells in WT and *Mn1* mutant embryos.

Sox10 immunofluorescence labelling of cranial neural crest cells from specified delaminating stage (E8.0-E8.25), migratory stage (E8.5-E9.0) and neural crest-derived ectomesenchyme (E9.25-E9.5). Insets represent a cross-section of the 3D confocal stack at E8.0 indicating no major differences in neural crest delamination between *Mn1* mutants and wild-types. White asterisks denote r3, separating the trigeminal (r1/2) and hyoid (r4) streams. Note the close proximity of the trigeminal and hyoid streams in $Mn1^{-/-}$ embryos as a consequence of the reduced r3 size (Fig. S17). White arrowheads indicate differences in the neural crest streams. Note the thinner streams in *Mn1* mutant embryos. White brackets highlight the glial cells of the trigeminal nerve. Scale bars: 500um. A total of 30 $Mn1^{+/+}$ and 25 $Mn1^{-/-}$ embryos were evaluated.

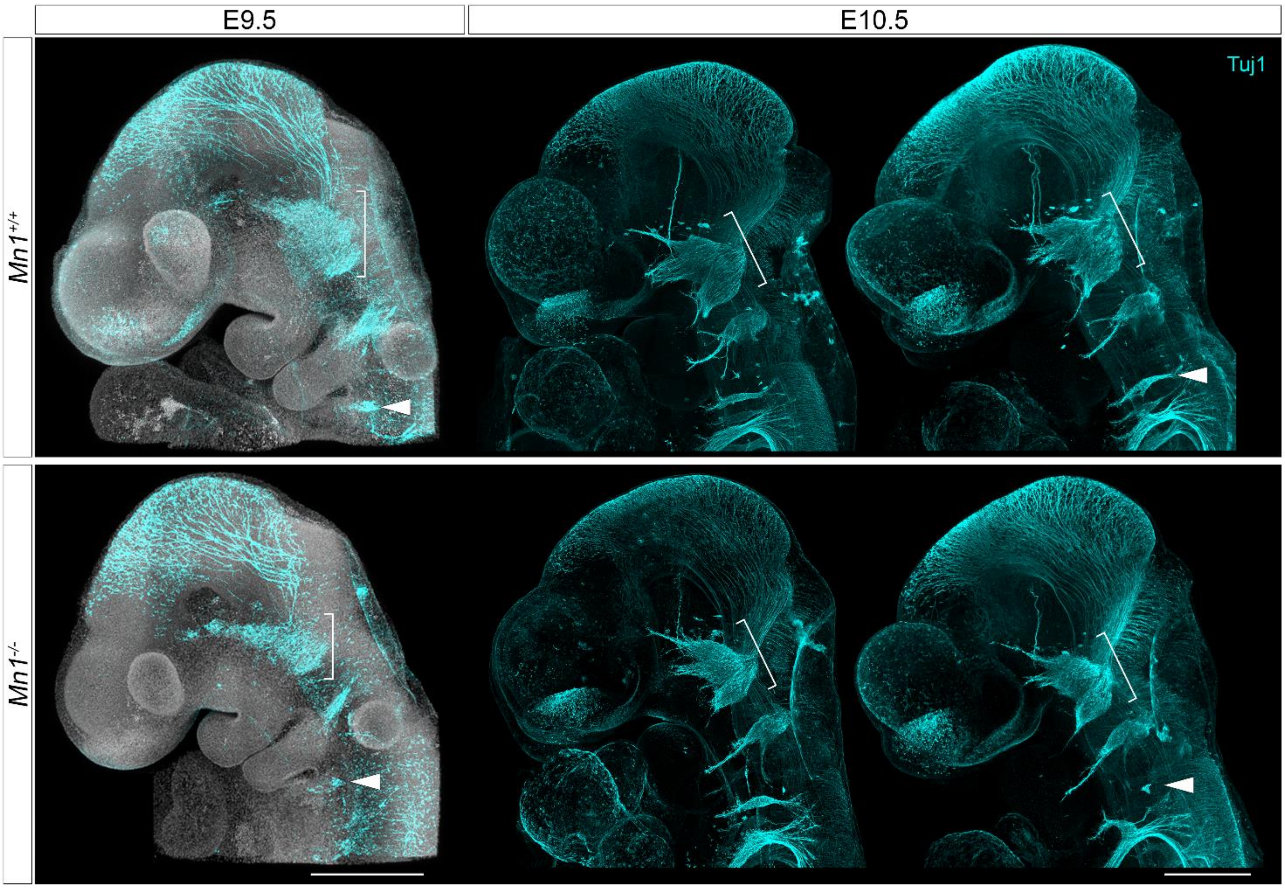

E9.5
E10.5
Tuj1
Mn1+/+
Mn1-/-

Fig. S19: Differences in cranial nerve development between WT and Mn1 mutant embryos.

Lateral view of Tuj1 immunofluorescence labelling the cranial nerves at E9.5 and E10.5. White arrowheads indicate differences in the 9th (glossopharyngeal) nerve. White brackets highlight the 5th (trigeminal) nerve. Note the differences in size of the trigeminal nerve that is smaller in the *Mn1* mutants. Scale bars: 500um. At least 5 embryos per genotype and developmental stage were assayed.

Table S1: List of all sequences used for phylogenetic analyses.

Species (common name)	Genome assembly	Accession number	Gene length (bp)			Protein (aa)	No. of exons	Chr. No.
			Full	CDS	Intronic			
A. calliptera (Eastern happy)	GCF_900246225.1	XP_026043257.1	14724	3750	10503	1249	2	12
*A. japonica****	GCF_011630105.1	XP_033126669.1	6887	3557	0	1185	2	Un.
A. mexicanus (Mexican tetra)	GCF_023375975.1	XP_007231342.3	24985	3753	18582	1250	2	22
A. mexicanus (Mexican tetra)*	GCF_023375975.1	XP_007250356.3	11197	3297	3969	1098	3	20
A. mississippiensis (American alligator)	GCF_030867095.1	XP_059569304.1	57957	3492	33173	1163	2	10
A. planci (Crown-of-thorns starfish)	GCF_001949145.1	XP_022103845.1	75168	4176	67138	1391	5	Un.
A. planci (Crown-of-thorns starfish)	GCF_001949145.1	XP_022103846.1	75168	4163	64561	1388	4	Un.
A. platyrhynchos (Mallard)	GCF_015476345.1	XP_038043438.1	42067	3618	32668	1205	2	16
A. prasina (Asian vine snake)	GCF_028640845.1	XP_058014462.1	25060	3552	18663	1183	2	15
A. radiata (Thorny skate)	GCF_010909765.2	XP_032899264.1	32140	3876	26568	1291	2	25
A. rubens (Common starfish)***	GCF_902459465.1	XP_033627923.1	7160	3947	0	1315	1	5
A. ruthenus (Sterlet)	GCF_902713425.1	XP_058889305.1	27309	3690	20778	1228	2	11
A. ruthenus (Sterlet)	GCF_902713425.1	XP_058850765.1	26590	3684	20785	1227	2	21
B. belcheri (Belcher's lancelet)	GCF_001625305.1	XP_019619803.1	5485	3018	1841	1005	6	Un.
B. bombina (Fire-bellied toad)	GCF_027579735.1	XP_053555779.1	61179	3630	50495	1209	2	2
B. bufo (Common toad)	GCF_905171765.1	XP_040272477.1	44217	3639	36518	1212	2	2
B. floridae (Florida lancelet)	GCF_000003815.2	XP_035690823.1	7993	3387	3957	1128	6	11
B. gargarizans (Asiatic toad)	GCF_014858855.1	XP_044131329.1	46338	3630	37798	1209	2	1
B. lanceolatum (European lancelet)	GCA_927797965.1	CAH1230539.1	10311	3411	3719	1136	6	1
B. musculus (Blue whale)	GCF_009873245.2	XP_036679459.1	46425	3933	38820	1310	2	14
B. taurus (Cattle)	GCF_002263795.1	XP_024833571.1	51029	3930	41726	1309	2	17
C. anna (Hummingbird)	GCF_003957555.1	XP_030316173.1	35622	3603	27066	1200	2	15
C. carcharias (Great white shark)**	GCF_017639515.1	XP_041058498.1	29493	3771	24177	1256	3	13
C. caretta (Loggerhead turtle)	GCF_023653815.1	XP_048677844.1	49156	3549	38774	1182	2	15
C. dromedarius (Arabian camel)	GCF_000803125.2	XP_031298819.1	46334	3954	38403	1317	2	32
C. elaphus (Red deer)*	GCF_910594005.1	XP_043757405.1	49591	3888	40924	1295	3	5
C. idella (Grass carp)	GCF_019924925.1	XP_051751088.1	16888	3703	10892	1233	2	5
C. idella (Grass carp)*	GCF_019924925.1	XP_051764998.1	16866	3105	7953	1034	6	10
C. japonica (Japanese quail)	GCF_001577835.2	XP_015733794.1	35586	3624	26644	1207	2	15
C. lupus familiaris (dog)	GCF_014441545.1	XP_038293041.1	44478	4011	39825	1336	2	26
C. mydas (Green sea turtle)	GCF_015237465.2	XP_027676597.2	190170	3519	39034	1172	2	15
C. olor (Mute swan)	GCF_009769625.2	XP_040432842.1	41969	3618	32867	1205	2	17
C. plagiosum (Bambooshark)	GCF_004010195.1	XP_043571554.1	135263	3852	21708	1283	2	25
C. tigris (Tiger rattlesnake)	GCF_016545835.1	XP_039218688.1	19349	3516	11649	1171	2	Un.
D. clupeoides (Denticle herring)	GCF_900700375.1	XP_028848160.1	15971	3675	10433	1224	2	1
D. clupeoides (Denticle herring)*	GCF_900700375.1	XP_028829142.1	6422	2823	2012	940	3	3
D. gliroides (Monito del monte)	GCF_019393635.1	XP_043835340.1	63929	4071	58595	1356	2	1
D. rerio (Zebrafish)	GCF_000002035.6	XP_001919775.2	29183	3636	22261	1211	2	5
D. rerio (Zebrafish)*	GCF_000002035.6	XP_700922.5	21565	3117	13760	1038	4	10
E. atami (Brown hagfish)	GCA_035128595.1	CM068955.1	-	2221	95674	739	2	4
E. burgeri (Inshore hagfish)	GCA_900186335.2	ENSEBUP00000014840.1	82247	2247	79959	748	2	Un.
E. caballus (Horse)	GCF_002863925.1	XP_023502694.1	45697	3921	37410	1306	2	8
E. calabaricus (Reedfish)	GCF_900747795.2	XP_028681260.2	19809	3735	13575	1244	2	18
E. electricus (Electric eel)	GCF_013358815.1	XP_026884848.2	16246	3729	10208	1242	2	9
E. electricus (Electric eel)*	GCF_013358815.1	XP_026877012.2	8833	3078	3042	1026	3	17
F. catus (Domestic cat)	GCF_018350175.1	XP_044897548.1	45055	3987	40491	1328	2	D3
G. aculeatus (stickleback)	GCF_016920845.1	XP_040051283.1	16238	3774	10238	1257	2	13
G. evgoodei (Goode's desert tortoise)	GCF_007399415.2	XP_030439028.1	47840	3414	39407	1137	2	13
G. flavomarginatus (Bolson tortoise)	GCF_025201925.1	XP_050779985.1	49105	3450	39506	1149	2	15
G. gallus (Chicken)	GCF_016699485.2	XP_003642254.4	36932	3633	27620	1210	2	15

H. capensis (Graceful crag lizard)	GCF_027244095.1	XP_053135727.1	32023	3621	25027	1206	2	Un.
H. grypus (Gray seal)*	GCF_012393455.1	XP_035939754.1	63190	3948	38627	1315	3	Un.
H. harpyja (Harpy eagle)	GCF_026419915.1	XP_052652193.1	118228	3624	33543	1207	2	9
H. ocellatum (Epaulette shark)	GCF_020745735.1	XP_060699555.1	133194	3864	21885	1287	2	24
H. sapiens (Human)	GCF_000001405.40	NP_002421.3	53480	3963	45668	1320	2	22
L. chalumnae (Coelacanth)	GCF_037176945.1	XP_005990304.1	197380	3666	39639	1221	2	20
L. erinacea (Little skate)	GCF_028641065.1	XP_055511849.1	109394	3831	26518	1276	2	25
L. oculatus (Spotted gar)	GCF_040954835.1	XP_006640342.1	35726	3675	29013	1224	2	LG22
L. pictus (Painted urchin)***	GCF_015342785.2	XP_054748843.1	5051	3557	0	1185	1	2
L. planeri (Brook lamprey)	GCA_029582275.1	CAL5933266.1	4635	4635	0	1544	1	37
L. reissneri (Far Eastern brook lamprey)	GCF_015708825.1	XP_061437803.1	5089	4689	0	1562	1	Un.
L. variegatus (Green sea urchin)***	GCF_018143015.1	XP_041455844.1	6478	3551	0	1183	1	2
M. glutinosa (Atlantic hagfish)	GCF_040869285.1	XP_067977160.1	179329	2211	176016	736	2	6
M. musculus (House mouse)*	GCF_000001635.27	NP_001074704.1	39746	3894	32734	1297	3	5
M. mutica (Yellowpond turtle)	GCF_020497125.1	XP_044846281.1	193127	3522	40008	1173	2	16
M. unicolor (Tiny Cayenne caecilian)	GCF_901765095.1	XP_030076003.1	112784	3645	108725	1214	2	11
N. parkeri (Mountain slow frog)	GCF_000935625.1	XP_018413277.1	64061	3642	60241	1213	2	Un.
N. scutatus (Mainland tiger snake)	GCF_900518725.1	XP_026541285.1	18357	3528	14750	1175	2	Un.
O. anatinus (Platypus)**	GCF_004115215.2	XP_028905047.1	67461	4014	58348	1337	3	21
O. niloticus (Nile tilapia)	GCF_001858045.2	XP_003445632.1	16763	3750	10645	1249	2	LG12
P. annectens (African lungfish)	GCF_019279795.1	XP_043924238.1	86138	3672	79324	1223	2	4
P. cinereus (Koala)	GCF_002099425.1	XP_020852318.1	67048	4068	60926	1355	2	Un.
P. flava (Hawaiian acorn worm)	GCF_041260155.1	XP_070573047.1	74018	4752	66340	1583	5	12
P. guttatus (Corn snake)	GCF_001185365.1	XP_034284863.1	22423	3528	15794	1175	2	Un.
P. marinus (Sea lamprey)*	GCF_010993605.1	XP_032835998.1	146073	4782	126928	1593	3	38
P. miniata (Bat starfish)***	GCF_015706575.1	XP_038059179.1	5597	3908	0	1302	1	Un.
P. muralis (Common wall lizard)	GCF_004329235.1	XP_028564804.1	41892	3537	34030	1178	2	16
P. nattereri (Red-bellied piranha)	GCF_015220715.1	XP_017569974.1	21293	3747	15016	1248	2	20
P. nattereri (Red-bellied piranha)*	GCF_015220715.1	XP_017549173.1	12953	3117	5133	1039	4	18
P. pectinata (Smalltooth sawfish)	GCF_009764475.1	XP_051888238.1	120395	3792	23626	1263	2	17
P. raffonei (Aeolian wall lizard)	GCF_027172205.1	XP_053224155.1	36236	3537	32287	1178	2	16
P. reticulata (Guppy)	GCF_000633615.1	XP_008417303.1	20162	3750	14001	1249	2	LG9
P. senegalus (Gray bichir)	GCF_016835505.1	XP_039628265.1	18810	3729	13611	1242	2	12
P. spathula (American paddlefish)	GCF_017654505.1	XP_041083177.1	26817	3678	21615	1225	2	25
P. spathula (American paddlefish)*	GCF_017654505.1	XP_041079134.1	25661	3678	19782	1225	3	22
R. bivittatum (Two-lined caecilian)	GCF_901001135.1	XP_029475839.1	103136	3642	94041	1213	2	11
R. temporaria (Common frog)	GCF_905171775.1	XP_040206779.1	83398	3675	78325	1224	2	1
R. typus (Whale shark)	GCF_021869965.1	XP_048467142.1	137623	3891	20660	1296	2	24
S. bombifrons (Plains spadefoot toad)	GCF_027358695.1	XP_053325584.1	26526	3627	22096	1208	2	1
S. canicula (Small-spotted catshark)	GCF_902713615.1	XP_038636815.1	248643	3828	71086	1275	2	1
S. fasciatum (Zebra shark)	GCF_022316705.1	XP_048411793.1	28279	3888	20603	1295	2	25
S. habroptila (Kakapo)	GCF_004027225.2	XP_030357190.1	34971	3612	26379	1203	2	11
S. kowalevskii (Acorn worm)	GCF_000003605.2	XP_002731338.2	57504	1608	55477	535	5	Un.
S. kowalevskii (Acorn worm)	GCF_000003605.2	XP_006812236.1	57504	1509	55181	502	4	Un.
S. meridionalis (Chinese catfish)*	GCF_014805685.1	XP_046716792.1	10282	3114	4028	1037	4	11
S. punctatus (Tuatara)**	GCA_003113815.1	ENSSPUT00000010422.1	35474	3423	32002	1140	3	Un.
S. purpuratus (Purple sea urchin)	GCF_000002235.3	XP_003727909.1	50499	3885	46205	1192	2	Un.
T. aculeatus (Australian equidna)**	GCF_015852505.1	XP_038619506.1	76650	3935	67554	1310	3	21
T. alba (Barn owl)	GCF_018691265.1	XP_032848803.2	40104	3630	31245	1209	2	Un.

T. guttata (Zebra finch)	GCF_003957565.2	XP_004176629.4	34572	3627	25856	1208	2	15
T. truncatus (Common dolphin)	GCF_011762595.1	XP_019805859.1	47739	3939	39186	1312	2	13
V. ursinus (Common wombat)	GCF_900497805.2	XP_027721393.1	64836	4083	60198	1360	2	Un.
X. laevis (African clawed frog)	GCF_017654675.1	XP_018116817.1	52142	3657	46061	1218	2	1L
X. laevis (African clawed frog)	GCF_017654675.1	XP_018099537.1	48202	3648	42969	1215	2	1S
X. tropicalis (Western clawed frog)	GCF_000004195.4	NP_001093672.1	45977	3633	40416	1210	2	1
Z. vivipara (Common lizard)	GCF_963506605.1	XP_060125269.1	42329	3582	33715	1193	2	17

The most recent and curated version of the inshore hagfish (*E. burgeri*) and tuatara (*S. punctatus*) genomes were retrieved from Ensembl to recover *MN1* homologs. The brown hagfish *MN1* ortholog was retrieved from the whole-genome shotgun contigs (*E. atami* - GCA_035128595.1) using the inshore hagfish *MN1* gene as a query using the BLASTn (BLAST nucleotide) tool and then translated to protein using the online tool Expasy (https://web.expasy.org/translate/). UTR regions of the brown hagfish *MN1* gene are not annotated, hence, the full length cannot be reliably assessed. *The gene is split in more than two exons but the coding region spawns across two of them. In these cases, only the intron between the two coding exons was included in the table. **The gene is split in more than two coding exons. Both intronic regions were summed up in such cases. ***The coding region is restricted to a single exon; hence, no intronic length could be included. Underlined species correspond to the 14 invertebrate *proto-MN1* sequences identified and analyzed in this study. Un: unplaced scaffold.

Table S2: Detailed information of BLAST and HMMER searches for MN1 homologs screening in Bilateria.

BLAST results

Forward query	Subject database	Forward BLAST hit	Forw. BLAST e-values	Forw. BLAST max score	Forw. BLAST query cover	Forw. BLAST Per. identity	Rev. BLAST hit	Rev. BLAST e-values	Rev. BLAST max scores	Rev. BLAST query cover	Rev. BLAST Per. Identity
Human MN1 (NP_002421.3)	NCBI. Human (taxid:9606)	Transcriptional activator MN1 (NP_002421.3) (Homo sapiens)	0.000000	2592	100%	100%	Transcriptional activator MN1 (NP_002421.3) (Homo sapiens)	0.00	2592	100%	100%
Human MN1 (NP_002421.3) exon 1	NCBI. Human (taxid:9606)	Transcriptional activator MN1 (NP_002421.3) (Homo sapiens)	0.000000	2466	100%	100%					
Human MN1 (NP_002421.3) exon 2	NCBI. Human (taxid:9606)	Transcriptional activator MN1 (NP_002421.3) (Homo sapiens)	0.000000	129	100%	100%					
Human MN1 (NP_002421.3)	Ensembl. Human (GRCh38)	ENSP00000304956 - MN1	0.000000	1716		100%	ENSP00000304956 - MN1	0.00	1716		100%
Human MN1 (NP_002421.3) exon 1	Ensembl. Human (GRCh38)	ENSP00000304956 - MN1	0.000000	1591		100%					
Human MN1 (NP_002421.3) exon 2	Ensembl. Human (GRCh38)	ENSP00000304956 - MN1	0.000000	129		100%					
Human MN1 (NP_002421.3)	NCBI. House mouse (taxid:10090)	Transcriptional activator MN1 (NP_001074704.1) (Mus musculus)	0.000000	1898	100%	89.24%	MN1 protein (CAA57693.2) (Homo sapiens)	0.00	1900	100%	89.01%
Human MN1 (NP_002421.3) exon 1	NCBI. House mouse (taxid:10090)	Transcriptional activator MN1 (NP_001074704.1) (Mus musculus)	0.000000	1793	100%	89.37%					
Human MN1 (NP_002421.3) exon 2	NCBI. House mouse (taxid:10090)	Transcriptional activator MN1 (NP_001074704.1) (Mus musculus)	0.000000	108	100%	86.67%					
Human MN1 (NP_002421.3)	Ensembl. Mouse (GRCm39)	ENSMUSP00000092034 - Mn1	0.000000	1320		88.33%	ENSP00000304956 - MN1	0.00	1562		88.78%
Human MN1 (NP_002421.3) exon 1	Ensembl. Mouse (GRCm39)	ENSMUSP00000092034 - Mn1	0.000000	1407		88.41%					
Human MN1 (NP_002421.3) exon 2	Ensembl. Mouse (GRCm39)	ENSMUSP00000092034 - Mn1	0.000000	108		86.67%					
Human MN1 (NP_002421.3)	NCBI. Chicken (taxid:9031)	Transcriptional activator MN1 (XP_040540679.1) (Gallus gallus)	0.000000	1144	100%	59.99%	Transcriptional activator MN1 (Homo sapiens)	0.00	1185	100%	59.88%
Human MN1 (NP_002421.3) exon 1	NCBI. Chicken (taxid:9031)	Transcriptional activator MN1 (XP_040540679.1) (Gallus gallus)	0.000000	1039	100%	59.18%					
Human MN1 (NP_002421.3) exon 2	NCBI. Chicken (taxid:9031)	Transcriptional activator MN1 (XP_040540679.1) (Gallus gallus)	0.000000	107	100%	83.61%					
Human MN1 (NP_002421.3)	Ensembl. Chicken (bGalGal1.mat.broiler.GRCg7b)	ENSGALP00010035027 - MN1	0.000000	453		62.86%	ENSP00000304956 - MN1	2.21E-129	431		55.44%
Human MN1 (NP_002421.3) exon 1	Ensembl. Chicken (bGalGal1.mat.broiler.GRCg7b)	ENSGALP00010035027 - MN1	0.000000	360		58.77%					
Human MN1 (NP_002421.3) exon 2	Ensembl. Chicken (bGalGal1.mat.broiler.GRCg7b)	ENSGALP00010035027 - MN1	0.000000	107		83.61%					
Human MN1 (NP_002421.3)	NCBI. Common wall lizard (taxid:64176)	Transcriptional activator MN1 (XP_028564804.1) (Podarcis muralis)	0.000000	251	28%	52.37%	MN1 protein (CAA57693.2) (Homo sapiens)	0.00	773	100%	49.02%
Human MN1 (NP_002421.3) exon 1	NCBI. Common wall lizard (taxid:64176)	Transcriptional activator MN1 (XP_028564804.1) (Podarcis muralis)	0.000000	161	25%	47.79%					
Human MN1 (NP_002421.3) exon 2	NCBI. Common wall lizard (taxid:64176)	Transcriptional activator MN1 (XP_028564804.1) (Podarcis muralis)	0.000000	92.8	100%	77.05%					
Human MN1 (NP_002421.3)	Ensembl. Common wall lizard (PodMur_1.0)	ENSPMRP00000035856 - MN1	0.000000	263		54.69%	ENSP00000304956 - MN1	1.11E-74	273		52.27%
Human MN1 (NP_002421.3) exon 1	Ensembl. Common wall lizard (PodMur_1.0)	ENSPMRP00000035856 - MN1	0.000000	262		54.69%					
Human MN1 (NP_002421.3) exon 2	Ensembl. Common wall lizard (PodMur_1.0)	ENSPMRP00000035856 - MN1	0.000000	92.8		77.05%					
Human MN1 (NP_002421.3)	NCBI. Xenopus tropicalis (taxid:8363)	Transcriptional activator MN1 (NP_001093672.1) (Xenopus tropiclais)	0.000000	1357	100%	59.88%	MN1 protein (CAA57693.2) (Homo sapiens)	0.00	1304	100%	58.70%
Human MN1 (NP_002421.3) exon 1	NCBI. Xenopus tropicalis (taxid:8363)	Transcriptional activator MN1 (NP_001093672.1) (Xenopus tropiclais)	0.000000	1258	100%	59.11%					
Human MN1 (NP_002421.3) exon 2	NCBI. Xenopus tropicalis (taxid:8363)	Transcriptional activator MN1 (NP_001093672.1) (Xenopus tropiclais)	0.000000	102	97%	81.36%					
Human MN1 (NP_002421.3)	Ensembl. Xenopus tropicalis (UCB_Xtro_10.0)	ENSXETP00000004796 - mn1	0.000000	975		57.93%	ENSP00000304956 - MN1	0.00	1203		58.89%
Human MN1 (NP_002421.3) exon 1	Ensembl. Xenopus tropicalis (UCB_Xtro_10.0)	ENSXETP00000004796 - mn1	0.000000	876		56.75%					
Human MN1 (NP_002421.3) exon 2	Ensembl. Xenopus tropicalis (UCB_Xtro_10.0)	ENSXETP00000004796 - mn1	0.000000	102		81.36%					
Human MN1 (NP_002421.3)	NCBI. Lungfishes (taxid:7878)	Transcriptional activator MN1 (XP_043924238.1) (Protopterus annectens)	0.000000	1283	100%	57.73%	MN1 protein (CAA57693.2) (Homo sapiens)	0.00	1236	100%	57.07%
Human MN1 (NP_002421.3) exon 1	NCBI. Lungfishes (taxid:7878)	Transcriptional activator MN1 (XP_043924238.1) (Protopterus annectens)	0.000000	1182	99%	56.29%					
Human MN1 (NP_002421.3) exon 2	NCBI. Lungfishes (taxid:7878)	Transcriptional activator MN1 (XP_043924238.1) (Protopterus annectens)	0.000000	102	100%	76.19%					
Human MN1 (NP_002421.3)	NCBI. Coelacanth (taxid:7897)	PREDICTED: probable tumor suppressor protein MN1 isoform X3 (XP_005990304.1) (Latimeria chalumnae)	0.000000	1374	100%	60.93%	MN1 protein (CAA57693.2) (Homo sapiens)	0.00	1302	100%	60.19%
Human MN1 (NP_002421.3) exon 1	NCBI. Coelacanth (taxid:7897)	Transcriptional activator MN1 isoform X1 (XP_005990303.1) (Latimeria chalumnae)	0.000000	1272	100%	60.22%					
Human MN1 (NP_002421.3) exon 2	NCBI. Coelacanth (taxid:7897)	Transcriptional activator MN1 isoform X2 (XP_005990304.1) (Latimeria chalumnae)	0.000000	100	100%	80.33%					
Human MN1 (NP_002421.3)	Ensembl. Coelacanth (LatCha1)	ENSLACP00000008128 - MN1	0.000000	915		56.15%	ENSP00000304956 - MN1	0.00	1142		60.22%
Human MN1 (NP_002421.3) exon 1	Ensembl. Coelacanth (LatCha1)	ENSLACP00000008128 - MN1	0.000000	866		56.73%					
Human MN1 (NP_002421.3) exon 2	Ensembl. Coelacanth (LatCha1)	ENSLACP00000008128 - MN1	0.000000	49.3		41.67%					
Human MN1 (NP_002421.3)	NCBI. Zebrafish (taxid:7955)	Transcriptional activator MN1 (XP_001919775.2) (Danio rerio)	0.000000	994	100%	50.70%	MN1 protein (CAA57693.2) (Homo sapiens)	5.00E-117	980	100%	50.44%
		Transcriptional activator MN1 (XP_700922.5) (Danio rerio)	0.000000	582	93%	39.13%	MN1 protein (CAA57693.2) (Homo sapiens)	1.00E-57	512	99%	37.81%

Human MN1 (NP_002421.3) exon 1	NCBI. Zebrafish (taxid:7955)	Transcriptional activator MN1 (XP_001919775.2) (Danio rerio)	0.000000	918	100%	50.08%					
		Transcriptional activator MN1 (XP_700922.5) (Danio rerio)	0.000000	583	98%	38.98%					
Human MN1 (NP_002421.3) exon 2	NCBI. Zebrafish (taxid:7955)	Transcriptional activator MN1 (XP_001919775.2) (Danio rerio)	0.000000	79.3	77%	80.85%					
Human MN1 (NP_002421.3)	Ensembl. Zebrafish (GRCz11)	ENSDARP00000107756 - mn1b	0.000000	350		50.90%	ENSP00000304956 - MN1	1.49E-117	398		50.56%
		ENSDARP00000100417 - mn1a	0.000000	203		40.91%	ENSP00000304956 - MN1	3.17E-58	221		38.56%
Human MN1 (NP_002421.3) exon 1	Ensembl. Zebrafish (GRCz11)	ENSDARP00000107756 - mn1b	0.000000	300		48.67%					
		ENSDARP00000100417 - mn1a	0.000000	201		40.46%					
Human MN1 (NP_002421.3) exon 2	Ensembl. Zebrafish (GRCz11)	ENSDARP00000107756 - mn1b	0.000000	79.3		80.85%					
Human MN1 (NP_002421.3)	NCBI. Spotted gar (taxid:7918)	PREDICTED: probable tumor suppressor protein MN1 (XP_006640342.1) (Lepisosteus oculatus)	0.000000	1238	100%	58.88%	MN1 protein (CAA57693.2) (Homo sapiens)	0.00	1238	100%	58.55%
Human MN1 (NP_002421.3) exon 1	NCBI. Spotted gar (taxid:7918)	Transcriptional activator MN1 (XP_006640342.2) (Lepisosteus oculatus)	0.000000	1141	100%	58.64%					
Human MN1 (NP_002421.3) exon 2	NCBI. Spotted gar (taxid:7918)	Transcriptional activator MN1 (XP_006640342.1) (Lepisosteus oculatus)	0.000000	97.4	100%	78.69%					
Human MN1 (NP_002421.3)	Ensembl. Spotted gar (LepOcu1)	ENSLOCP00000009509 - mn1	0.000000	809		55.10%	ENSP00000304956 - MN1	0.00	1046		57.36%
Human MN1 (NP_002421.3) exon 1	Ensembl. Spotted gar (LepOcu1)	ENSLOCP00000009509 - mn1	0.000000	707		53.84%					
Human MN1 (NP_002421.3) exon 2	Ensembl. Spotted gar (LepOcu1)	ENSLOCP00000009509 - mn1	0.000000	97.4		78.69%					
Human MN1 (NP_002421.3)	NCBI. Paddlefishes (taxid:7911)	Transcriptional activator MN1-like (XP_041083177.1) (Polyodon spathula)	0.000000	1304	100%	58.40%	MN1 protein (CAA57693.2) (Homo sapiens)	0.00	1253	100%	57.78%
		Transcriptional activator MN1-like (XP_041079134.1) (Polyodon spathula)	0.000000	1291	100%	58.11%	MN1 protein (CAA57693.2) (Homo sapiens)	0.00	1242	100%	57.78%
Human MN1 (NP_002421.3) exon 1	NCBI. Paddlefishes (taxid:7911)	Transcriptional activator MN1-like (XP_041083177.1) (Polyodon spathula)	0.000000	1212	100%	58.60%					
		Transcriptional activator MN1-like (XP_041079134.1) (Polyodon spathula)	0.000000	1200	100%	58.14%					
Human MN1 (NP_002421.3) exon 2	NCBI. Paddlefishes (taxid:7911)	Transcriptional activator MN1-like (XP_041079134.1) (Polyodon spathula)	0.000000	90.5	100%	73.77%					
		Transcriptional activator MN1-like (XP_041083177.1) (Polyodon spathula)	0.000000	88.2	100%	70.49%					
Human MN1 (NP_002421.3)	NCBI. Bichirs (taxid:8289)	Transcriptional activator MN1-like (XP_039628265.1) (Polypterus senegalus)	0.000000	1220	100%	57.22%	MN1 protein (CAA57693.2) (Homo sapiens)	0.00	1161	100%	56.53%
Human MN1 (NP_002421.3) exon 1	NCBI. Bichirs (taxid:8289)	Transcriptional activator MN1-like (XP_039628265.1) (Polypterus senegalus)	0.000000	1129	100%	56.48%					
Human MN1 (NP_002421.3) exon 2	NCBI. Bichirs (taxid:8289)	Transcriptional activator MN1-like (XP_039628265.1) (Polypterus senegalus)	0.000000	90.9	85%	86.27%					
Human MN1 (NP_002421.3)	NCBI. Scyliorhinus (taxid:7829)	Transcriptional activator MN1 isoform X2 (XP_038636815.1) (Scyliorhinus canicula)	0.000000	1122	100%	53.28%	MN1 protein (CAA57693.2) (Homo sapiens)	0.00	1085	100%	53.00%
Human MN1 (NP_002421.3) exon 1	NCBI. Scyliorhinus (taxid:7829)	Transcriptional activator MN1 isoform X1 (XP_038636734.1) (Scyliorhinus canicula)	0.000000	1025	100%	52.14%					
Human MN1 (NP_002421.3) exon 2	NCBI. Scyliorhinus (taxid:7829)	Transcriptional activator MN1 isoform X2 (XP_038636815.1) (Scyliorhinus canicula)	0.000000	99.4	100%	76.67%					
Human MN1 (NP_002421.3)	NCBI. Hagfishes (taxid:7761)	Transcriptional activator MN1-like (XP_067977160.1) (Myxine glutinosa)	0.000000	65.5	5%	50.00%					
Human MN1 (NP_002421.3) exon 1	NCBI. Hagfishes (taxid:7761)	Transcriptional activator MN1-like (XP_067977160.1) (Myxine glutinosa)	0.000000	65.5	5%	50.00%					
Human MN1 (NP_002421.3) exon 2	NCBI. Hagfishes (taxid:7761)	No significant similarity found									
Human MN1 (NP_002421.3)	Ensembl. Inshore hagfish (Eburgeri_3.2)	ENSEBUG00000009356	0.000000	67.4		65.30%	ENSP00000304956 - MN1	2.41E-11	69.3		47.77%
Human MN1 (NP_002421.3) exon 1	Ensembl. Inshore hagfish (Eburgeri_3.2)	ENSEBUG00000009356	0.000000	67		65.31%					
Human MN1 (NP_002421.3) exon 2	Ensembl. Inshore hagfish (Eburgeri_3.2)	No significant similarity found									
Human MN1 (NP_002421.3)	NCBI. Lampreys (taxid:7745)	Neurogenic protein mastermind-like (XP_032835998.1) (Petromyzon marinus)	0.000000	63.5	2%	87.50%	Meningioma (disrupted in balanced translocation) 1 (ABQ01232.1) (Homo sapiens)	6.00E-10	68.2	3%	60.71%
		Trithorax group protein osa-like (XP_061437803.1) (Lethenteron reissneri)	0.000000	63.2	2%	87.50%	Meningioma (disrupted in balanced translocation) 1 (ABQ01232.1) (Homo sapiens)	2.00E-09	66.6	3%	56.90%
		Unnamed protein product (CAL5933266.1) (Lampetra planeri)	0.000000	63.5	2%	87.50%					
Human MN1 (NP_002421.3) exon 1	NCBI. Lampreys (taxid:7745)	Neurogenic protein mastermind-like (XP_032835998.1) (Petromyzon marinus)	0.000000	63.5	3%	87.50%					
		Trithorax group protein osa-like (XP_061437803.1) (Lethenteron reissneri)	0.000000	63.2	3%	87.50%					
		Unnamed protein product (CAL5933266.1) (Lampetra planeri)	0.000000	3	64%	87.50%					
Human MN1 (NP_002421.3) exon 2	NCBI. Lampreys (taxid:7745)	No significant similarity found									
Human MN1 (NP_002421.3)	Ensembl. Sea lamprey (Pmarinus_7.0)	No significant similarity found									
Human MN1 (NP_002421.3) exon 1	Ensembl. Sea lamprey (Pmarinus_7.0)	No significant similarity found									
Human MN1 (NP_002421.3) exon 2	Ensembl. Sea lamprey (Pmarinus_7.0)	No significant similarity found									
Human MN1 (NP_002421.3)	NCBI. Tunicata (taxid:7712)	No significant similarity found									
Human MN1 (NP_002421.3) exon 1	NCBI. Tunicata (taxid:7712)	No significant similarity found									

Human MN1 (NP_002421.3) exon 2	NCBI. Tunicata (taxid:7712)	No significant similarity found									
Human MN1 (NP_002421.3)	Ensembl. C. intestinalis KH	No significant similarity found									
Human MN1 (NP_002421.3) exon 1	Ensembl. C. intestinalis KH	No significant similarity found									
Human MN1 (NP_002421.3) exon 2	Ensembl. C. intestinalis KH	No significant similarity found									
Human MN1 (NP_002421.3)	Ensembl. C. savignyi CSAV2.0	No significant similarity found									
Human MN1 (NP_002421.3) exon 1	Ensembl. C. savignyi CSAV2.1	No significant similarity found									
Human MN1 (NP_002421.3) exon 2	Ensembl. C. savignyi CSAV2.0	No significant similarity found									
Human MN1 (NP_002421.3)	NCBI. Cephalochordata (taxid:7735)	No significant similarity found by forward BLAST. The following sequences were labelled as part of the "MN1 family by Uniprot and InterPro									
		Hypp316 (CAH1230539.1) (Branchiostoma lanceolatum)									
		Uncharacterized protein LOC109466517 (XP_019619803.1) (Branchiostoma belcheri)									
		Uncharacterized protein LOC118425828 (XP_035690823.1) (Branchiostoma floridae)									
Human MN1 (NP_002421.3) exon 1	NCBI. Cephalochordata (taxid:7735)	No significant similarity found by forward BLAST. The following sequences were labelled as part of the "MN1 family by Uniprot and InterPro									
		Hypp316 (CAH1230539.1) (Branchiostoma lanceolatum)									
		Uncharacterized protein LOC109466517 (XP_019619803.1) (Branchiostoma belcheri)									
		Uncharacterized protein LOC118425828 (XP_035690823.1) (Branchiostoma floridae)									
Human MN1 (NP_002421.3) exon 2	NCBI. Cephalochordata (taxid:7735)	No significant similarity found									
Human MN1 (NP_002421.3)	EnsemblMetazoa. Branchiostoma lanceolatum (BraLan2)	No significant similarity found									
Human MN1 (NP_002421.3) exon 1	EnsemblMetazoa. Branchiostoma lanceolatum (BraLan2)	No significant similarity found									
Human MN1 (NP_002421.3) exon 2	EnsemblMetazoa. Branchiostoma lanceolatum (BraLan2)	No significant similarity found									
Human MN1 (NP_002421.3)	NCBI. Hemichordata (taxid:10219)	PREDICTED: mucin-21-like isoform X2 (XP_006812236.1) (Saccoglossus kowalevskii)	0.000004	50.4	8%	37.61%	MN1 protein (CAA57693.2) (Homo sapiens)	6.00E-06	52.8	19%	37.61%
		PREDICTED: mucin-21-like isoform X1 (XP_002731338.2) (Saccoglossus kowalevskii)	0.000008	50.1	8%	37.61%	MN1 protein (CAA57693.2) (Homo sapiens)	1.00E-05	52.4	18%	37.61%
		myb-like protein AA (XP_070573047.1) (Ptychodera flava)	0.000200	47.8	15%	31.37%					
Human MN1 (NP_002421.3) exon 1	NCBI. Hemichordata (taxid:10219)	PREDICTED: mucin-21-like isoform X2 (XP_006812236.1) (Saccoglossus kowalevskii)	0.000020	50.1	9%	37.61%					
		PREDICTED: mucin-21-like isoform X1 (XP_002731338.2) (Saccoglossus kowalevskii)	0.000030	49.7	9%	37.61%					
		myb-like protein AA (XP_070573047.1) (Ptychodera flava)	0.000400	45.2	15%	32.09%					
Human MN1 (NP_002421.3) exon 2	NCBI. Hemichordata (taxid:10219)	No significant similarity found									
Human MN1 (NP_002421.3)	EnsemblMetazoa. Saccoglossus kowalevskii Skow_1.1	No significant similarity found									
Human MN1 (NP_002421.3) exon 1	EnsemblMetazoa. Saccoglossus kowalevskii Skow_1.2	No significant similarity found									
Human MN1 (NP_002421.3) exon 2	EnsemblMetazoa. Saccoglossus kowalevskii Skow_1.1	No significant similarity found									
Human MN1 (NP_002421.3)	NCBI. Echinodermata (taxid:7586)	Uncharacterized protein F59B2.12-like (XP_041455844.1) (Lytechinus variegatus)	0.000020	52.8	6%	32.18%	No significant similarity found				
		Uncharacterized protein F59B2.12-like (XP_054748843.1) (Lytechinus pictus)	0.000020	52.4	6%	32.18%	No significant similarity found				
		Putative GPI-anchored protein pfl2 (XP_038059179.1) (Patiria miniata) LOC119730385	0.000100	50.1	8%	36.52%	MN1 protein (CAA57693.2) (Homo sapiens)	1.00E-04	50.4	7%	36.52%
Human MN1 (NP_002421.3) exon 1	NCBI. Echinodermata (taxid:7586)	Uncharacterized protein F59B2.12-like (XP_041455844.1) (Lytechinus variegatus)	0.000002	52.8	7%	32.18%					
		Uncharacterized protein F59B2.12-like (XP_054748843.2) (Lytechinus pictus)	0.000003	52.4	7%	32.18%					
		Putative GPI-anchored protein pfl2 (XP_038059179.1) (Patiria miniata) LOC119730385	0.000020	50.1	8%	40.62%					
Human MN1 (NP_002421.3) exon 2	NCBI. Echinodermata (taxid:7586)	No significant similarity found									
Human MN1 (NP_002421.3)	EnsemblMetazoa. Acanthaster planci OKI-Apl_1.0	Mucin-5AC-like isoform X1 (XP_022103845.1) (Acanthaster planci)	0.099000	78		45.50%	MN1 protein (CAA57693.2) (Homo sapiens) Reverse BLAST performed in NCBI	4.00E-03	45.8	7%	31.40%
		Mucin-5AC-like isoform X2 (XP_022103846.1) (Acanthaster planci)	0.099000	78		45.50%	Meningioma (disrupted in balanced translocation) 1 (ABQ01232.1) (Homo sapiens) Reverse BLAST performed in NCBI	4.00E-03	45.8	7%	31.40%

Human MN1 (NP_002421.3) exon 1	EnsemblMetazoa. Acanthaster planci OKI-Apl_1.0	LOC110986351 Mucin-5AC-like isoform X1 (XP_022103845.1) (Acanthaster planci)	0.097000	78		45.50%					
		LOC110986351 Mucin-5AC-like isoform X1 (XP_022103846.1) (Acanthaster planci)	0.097000	78		45.50%					
Human MN1 (NP_002421.3) exon 2	EnsemblMetazoa. Acanthaster planci OKI-Apl_1.0	No significant similarity found									
Human MN1 (NP_002421.3)	EnsemblMetazoa. Asterias rubens eAstRub1.3	Flocculation protein FLO11-like (XP_033627923.1) (Asterias rubens) LOC117290576	0.066000	78		56.00%	MN1 protein (CAA57693.2) (Homo sapiens)<br>Reverse BLAST performed in NCBI	9.00E-03	44.3	5%	40.70%
Human MN1 (NP_002421.3) exon 1	EnsemblMetazoa. Asterias rubens eAstRub1.3	ENSASRP00000000250	0.064000	78		56.00%					
Human MN1 (NP_002421.3) exon 2	EnsemblMetazoa. Asterias rubens eAstRub1.3	No significant similarity found									
Human MN1 (NP_002421.3)	EnsemblMetazoa. Anneissia japonica ASM1163010v1	No significant similarity found. The following sequence is highly similar to the rest of invertebrate hits and was added to the analysis<br>Uncharacterized protein LOC117124526 (XP_033126669.1) (Anneissia japonica)					Meningioma (disrupted in balanced translocation) 1 (ABQ01232.1) (Homo sapiens)<br>Reverse BLAST performed in NCBI	1.10E-02	43.9	8%	31.85%
Human MN1 (NP_002421.3) exon 1	EnsemblMetazoa. Anneissia japonica ASM1163010v1	No significant similarity found									
Human MN1 (NP_002421.3) exon 2	EnsemblMetazoa. Anneissia japonica ASM1163010v1	No significant similarity found									
Human MN1 (NP_002421.3)	EnsemblMetazoa. Patiria miniata Pmin_3.0	Putative GPI-anchored protein pfl2 (XP_038059179.1) (Patiria miniata) LOC119730385	0.032000	82		43.60%					
Human MN1 (NP_002421.3) exon 1	EnsemblMetazoa. Patiria miniata Pmin_3.0	Putative GPI-anchored protein pfl2 (XP_038059179.1) (Patiria miniata) LOC119730385	0.031000	82		43.60%					
Human MN1 (NP_002421.3) exon 2	EnsemblMetazoa. Patiria miniata Pmin_3.0	No significant similarity found									
Human MN1 (NP_002421.3)	EnsemblMetazoa. Strongylocentrotus purpuratus Spur_5.0	No significant similarity found									
Human MN1 (NP_002421.3) exon 1	EnsemblMetazoa. Strongylocentrotus purpuratus Spur_5.0	No significant similarity found									
Human MN1 (NP_002421.3) exon 2	EnsemblMetazoa. Strongylocentrotus purpuratus Spur_5.0	No significant similarity found									
Human MN1 (NP_002421.3)	EnsemblMetazoa. Lytechinus variegatus Lvar_3.0	Uncharacterized protein F59B2.12-like (XP_041455844.1) (Lytechinus variegatus)	0.057000	80		57.70%					
Human MN1 (NP_002421.3) exon 1	EnsemblMetazoa. Lytechinus variegatus Lvar_3.0	Uncharacterized protein F59B2.12-like (XP_041455844.1) (Lytechinus variegatus)	0.055000	80		57.70%					
Human MN1 (NP_002421.3) exon 2	EnsemblMetazoa. Lytechinus variegatus Lvar_3.0	No significant similarity found									
Human MN1 (NP_002421.3)	Ensembl. Fruit fly BDGP6.46	No significant similarity found									
Human MN1 (NP_002421.3) exon 1	Ensembl. Fruit fly BDGP6.46	No significant similarity found									
Human MN1 (NP_002421.3) exon 2	Ensembl. Fruit fly BDGP6.46	No significant similarity found									
Human MN1 (NP_002421.3)	Ensembl. Nematode, N2 WBcel235	No significant similarity found									
Human MN1 (NP_002421.3) exon 1	Ensembl. Nematode, N2 WBcel235	No significant similarity found									
Human MN1 (NP_002421.3) exon 2	Ensembl. Nematode, N2 WBcel235	No significant similarity found									
Human MN1 (NP_002421.3)	Ensembl. Saccharomyces cerevisiae R64-1-1	No significant similarity found									
Human MN1 (NP_002421.3) exon 1	Ensembl. Saccharomyces cerevisiae R64-1-1	No significant similarity found									
Human MN1 (NP_002421.3) exon 2	Ensembl. Saccharomyces cerevisiae R64-1-1	No significant similarity found									
Human MN1 (NP_002421.3)	NCBI. Protostomia (taxid:33317)	No significant similarity found									
Human MN1 (NP_002421.3) exon 1	NCBI. Protostomia (taxid:33317)	No significant similarity found									
Human MN1 (NP_002421.3) exon 2	NCBI. Prostostomia (taxid:33317)	No significant similarity found									
Hagfish MN1-like (ENSEBUG00000009356)	NCBI. Human (taxid:9606)	Truncated transcriptional activator MN1 (WOF00475.1) (Homo sapiens)	0.000000	70.1	12%	48.89%	No significant similarity found				
Hagfish MN1-like (ENSEBUG00000009356)	NCBI. Lampreys (taxid:7745)	Neurogenic protein mastermind-like (XP_032835998.1) (Petromyzon marinus)	0.000000	68.6	10%	94.12%	No significant similarity found				
		Trithorax group protein osa-like (XP_061437803.1) (Lethenteron reissneri)	0.000000	68.2	10%	94.12%	No significant similarity found				
Hagfish MN1-like (ENSEBUG00000009356)	Ensembl. Sea lamprey (Pmarinus_7.0)	No significant similarity found									
Hagfish MN1-like (ENSEBUG00000009356)	NCBI. Cephalochordata (taxid:7735)	No significant similarity found									
Hagfish MN1-like (ENSEBUG00000009356)	NCBI. Hemichordata (taxid:10219)	No significant similarity found									
Hagfish MN1-like (ENSEBUG00000009356)	NCBI. Echinodermata (taxid:7586)	No significant similarity found									

Neurogenic protein mastermind-like (XP_032835998.1) (Petromyzon marinus)	Ensembl. Human (GRCh38)	ENSP00000304956 - MN1	0.000000	63.2		87.50%	No significant similarity found				
Neurogenic protein mastermind-like (XP_032835998.1) (Petromyzon marinus)	Ensembl. Inshore hagfish (Eburgeri_3.2)	ENSEBUG00000009356	0.000000	68.2		94.11%	No significant similarity found				
Neurogenic protein mastermind-like (XP_032835998.1) (Petromyzon marinus)	NCBI. Hagfishes (taxid:7761)	No significant similarity found									
Acanthaster planci Mucin-5AC_like (XP_022103845.1)	NCBI. Cephalochordata (taxid:7735)	Uncharacterized protein LOC109466517 (XP_019619803.1) (Branchiostoma belcheri)	0.000000	115	15%	36.99%	Mucin-5AC-like isoform X1 (XP_022103845.1) (Acanthaster planci)	7.00E-25	116	20%	36.99%
		Uncharacterized protein LOC118425828 (XP_035690823.1) (Branchiostoma floridae)	0.000000	111	13%	39.00%	Mucin-5AC-like isoform X1 (XP_022103845.1) (Acanthaster planci)	1.00E-23	112	15%	39.00%
		Hypp316 (CAH1230539.1) (Branchiostoma lanceolatum)	0.000000	110	14%	38.57%	Mucin-5AC-like isoform X1 (XP_022103845.1) (Acanthaster planci)	2.00E-23	111	16%	38.57%
Acanthaster planci Mucin-5AC_like (XP_022103845.1)	NCBI. Hemichordata (taxid:10219)	PREDICTED: mucin-21-like isoform X1 (XP_002731338.2) (Saccoglossus kowalevskii)	0.000000	203	13%	54.23%	Mucin-5AC-like isoform X1 (XP_022103845.1) (Acanthaster planci)	5.00E-55	203	36%	54.23%
		PREDICTED: mucin-21-like isoform X2 (XP_006812236.1) (Saccoglossus kowalevskii)	0.000000	151	11%	51.18%	Mucin-5AC-like isoform X1 (XP_022103845.1) (Acanthaster planci)	1.00E-37	151	32%	51.18%
Acanthaster planci Mucin-5AC_like (XP_022103845.1)	NCBI. Echinodermata (taxid:7586)	Putative GPI-anchored protein pfl2 (XP_038059179.1) (Patiria miniata) LOC119730385	0.000000	1966	93%	78.67%	Mucin-5AC-like isoform X1 (XP_022103845.1) (Acanthaster planci)	0.00	1959	99%	78.98%
		Uncharacterized protein LOC119730133 (XP_038058834.1) (Patiria miniata) LOC119730133	0.000000	203	7%	90.57%	Mucin-5AC-like isoform X1 (XP_022103845.1) (Acanthaster planci)	1.00E-60	203	85%	90.57%
		Flocculation protein FLO11-like (XP_033627923.1) (Asterias rubens) LOC117290576	0.000000	1495	93%	64.07%	Mucin-5AC-like isoform X1 (XP_022103845.1) (Acanthaster planci)	0.00	1496	99%	63.44%
		Uncharacterized protein LOC117290482 (XP_033627791.1) (Asterias rubens)	0.000000	176	7%	78.50%	Mucin-5AC-like isoform X1 (XP_022103845.1) (Acanthaster planci)	7.00E-51	176	75%	78.50%
		Uncharacterized protein F59B2.12-like (XP_054748843.1) (Lytechinus pictus)	0.000000	254	47%	33.76%	Mucin-5AC-like isoform X1 (XP_022103845.1) (Acanthaster planci)	1.00E-57	221	53%	33.71%
		Uncharacterized protein F59B2.12-like (XP_041455844.1) (Lytechinus variegatus)	0.000000	252	49%	33.56%	Mucin-5AC-like isoform X1 (XP_022103845.1) (Acanthaster planci)	2.00E-57	221	54%	33.71%
		Uncharacterized protein LOC117124526 (XP_033126669.1) (Anneissia japonica)	0.000000	201	61%	30.22%	Mucin-5AC-like isoform X1 (XP_022103845.1) (Acanthaster planci)	7.00E-45	181	66%	30.14%
		Max-like protein X (XP_033126799.1) (Anneissia japonica)	0.000000	119	7%	56.73%	Mucin-5AC-like isoform X1 (XP_022103845.1) (Acanthaster planci)	2.00E-30	119	57%	56.73%
Human MN1 (NP_002421.3)	Echinobase	PREDICTED: uncharacterized protein LOC100890828 (XP_003727909.1) (Strongylocentrotus purpuratus)	0.000003	54.7							
		Uncharacterized protein F59B2.12-like (XP_041453693.1) (Lytechinus variegatus)	0.000010	52.8							
		Putative GPI-anchored protein pfl2 (XP_038059179.1) (Patiria miniata)	0.000080	50.1							
		Mucin-5AC-like isoform X1 (XP_022103845.1) (Acanthaster planci)	0.005000	44.7							
		Mucin-5AC-like isoform X1 (XP_022103846.1) (Acanthaster planci)	0.005000	44.3							
Human MN1 (NP_002421.3) exon 2	Echinobase	No significant similarity found									

HMMER results

Genome/Proteome screened	Sequence hit	e-value	bit score	bias score
GCF017654505_1 (Polyodon spathula)	Transcriptional activator MN1-like (XP_041083177.1)	0.00	2254.6	106.5
	Transcriptional activator MN1-like (XP_041079134.1)	0.00	2246.2	102.9
GCA016984155_1 (Amia calva)	MN1 protein (MBN3309335.1)	0.00	2245.5	115.6
GCF019279795_1 (Protopterus annectens)	Transcriptional activator MN1 (XP_043924238.1)	0.00	2108.9	104.7
UP000005640 (Homo sapiens)	Transcriptional activator MN1 (Q10571)	0.00	1918.5	122.1
UP000000589 (Mus musculus)	Transcriptional activator MN1 (D3YWE6)	0.00	1910.0	111.5
GCF028641065_1 (Leucoraja erinacea)	Transcriptional activator MN1 isoform X2 (XP_055511849.1)	0.00	1766.8	105.3
	Transcriptional activator MN1 isoform X1 (XP_055511848.1)	0.00	1697.3	104.8
GCA026652325_1 (Pleurodeles waltl)	Hypothetical protein NDU88_005769 (KAJ1092659.1)	0.00	1615.7	68.9
UP000694388 (Eptatretus burgeri)	Androgen receptor (A0A8C4QH12)	6.00E-83	266.5	31.7
GCF_015708825.1 (Lethenteron reissneri)	Trithorax group protein osa-like (XP_061437803.1)	4.00E-17	65.2	41.9
GCF_010993605.1 (Petromyzon marinus)	Neurogenic protein mastermind-like (XP_032835998.1)	4.40E-14	55.1	49.2
GCF000003605_2 (Saccoglossus kowalevskii)	PREDICTED: mucin-21-like isoform X2 (XP_006812236.1)	1.70E-59	82.4	20.2
	PREDICTED: mucin-21-like isoform X1 (XP_002731338.2)	7.50E-22	82.2	20.2
UP000694845 (Acanthaster planci)	Mucin-5AC-like isoform X1 (A0A8B7ZG54)	8.60E-22	63.2	55.5
UP000887568 (Patiria miniata)	Uncharacterized protein (A0A914A6T0)	6.30E-16	62.7	54.6
GCF_902459465.1 (Asterias rubens)	Flocculation protein FLO11-like (XP_033627923.1)	8.20E-07	33	100.7
GCF_011630105.1 (Anneissia japonica)	No significant similarity found			
UP000515135 (Branchiostoma belcheri)	No significant similarity found			
UP000838412 (Branchiostoma lanceolatum)	No significant similarity found			
GCF_015342785.2 (Lytechinus pictus)	No significant similarity found			
GCF_018143015.1 (Lytechinus variegatus)	No significant similarity found			
UP000001940 (Caenorhabditis elegans)	No significant similarity found			
UP000008144 (Ciona intestinalis)	No significant similarity found			
UP000000803 (Drosophila melanogaster)	No significant similarity found			
UP000245300 (Sea lamprey)	No significant similarity found			
UP000001554 (Branchiostoma floridae)	No significant similarity found			
UP000515154 (Octopus vulgaris)	No significant similarity found			
UP000001307 (Oikopleura dioica)	No significant similarity found			
UP000007110 (Strongylocentrotus purpuratus)	No significant similarity found			

Table S3: Protein domains identified in representative MN1 and proto-MN1 sequences.

Species	Protein domain (accession)	E-value	Position
Human	No known conserved domains identified		
Mouse	No known conserved domains identified		
Chicken	No known conserved domains identified		
Lizard	No known conserved domains identified		
Frog	No known conserved domains identified		
Lungfish	PABP-1234 superfamily (cl31127)	2.70e-03	367-542
Coelacanth	No known conserved domains identified		
Zebrafish (mn1a)	No known conserved domains identified		
Zebrafish (mn1b)	No known conserved domains identified		
Spotted gar	PABP-1234 superfamily (cl31127)	5.31e-03	620-707
Paddlefish (Chr.22)	Med15 superfamily (cl26621)	5.28e-04	492-665
Paddlefish (Chr.25)	Med15 superfamily (cl26621)	2.77e-03	486-665
Gray bichir	No known conserved domains identified		
Catshark	No known conserved domains identified		
Atlantic hagfish	No known conserved domains identified		
Sea lamprey	No known conserved domains identified		
Belcher's lancelet	Basic helix-loop-helix (bHLH) domain (cd00083)	6.32e-11	923-966
Florida lancelet	Basic helix-loop-helix (bHLH) domain (cd00083)	7.09e-11	1043-1086
European lancelet	Basic helix-loop-helix (bHLH) domain (cd00083)	2.46e-09	1051-1094
Acorn worm (X1)	Basic helix-loop-helix (bHLH) domain (cd00083)	8.82e-10	450-493
Acorn worm (X2)	Basic helix-loop-helix (bHLH) domain (cd00083)	1.05e-06	450-486
Hawaiian acorn worm	Basic helix-loop-helix (bHLH) domain (cd00083)	1.14e-08	1498-1541
Crown-of-thorns starfish (X1)	Basic helix-loop-helix (bHLH) domain (cd00083)	4.68e-07	1306-1349
Crown-of-thorns starfish (X2)	Basic helix-loop-helix (bHLH) domain (cd00083)	4.67e-07	1306-1349
Bat starfish	PTZ00395 superfamily (cl33180)	5.07e-05	157-396
Common starfish	Serine-rich family protein (cl41472)	8.54e-03	111-364
Purple sea urchin	No known conserved domains identified		
Painted urchin	No known conserved domains identified		
Green sea urchin	Serine-rich family protein (cl41472)	4.26e-03	37-183
A. japonica	No known conserved domains identified		

Summary of the different known protein domains identified in representative vertebrate MN1 and invertebrate proto-MN1 amino acid sequences.

Table S4: *MN1*-expressing cell types in the developing mouse.

	Pijuan-Sala *et al.* (4)	La Manno *et al.* (5)	Soldatov *et al.* (6)	Kelly *et al.* (7)	Cao *et al.* (8)
E8.5	Paraxial mesoderm<br>Pharyngeal mesoderm<br>Neural crest<br>Fb, Mb, Hb<br>Spinal cord	Neural crest<br>Neural tube<br>Paraxial mesoderm<br>Mesenchyme<br>Radial glia<br>Fb, Mb, Hb	Cranial neural crest		
E9.5		Neural crest<br>Neural tube<br>Paraxial mesoderm<br>Mesenchyme<br>Radial glia<br>Fb, Mb, Hb<br>Mb-Hb boundary	Trunk neural crest<br>(basal expression)		Chondrocyte prog.<br>Jaw/tooth prog.<br>Neural tube<br>Notochord cells<br>Oligodendrocyte prog.<br>Radial glia
E10.5		Mesenchyme<br>Radial glia<br>Fb, Mb, Hb<br>Mb-Hb boundary<br>Neuroblasts			Chondrocyte prog.<br>Jaw/tooth prog.<br>Neural tube<br>Notochord cells<br>Oligodendrocyte prog.<br>Radial glia<br>Chondrocyte/osteoblasts<br>Connective tissues prog.<br>Granule neurons<br>Inhibitory neurons prog.<br>Inhibitory neurons<br>Isthmic organizer cells<br>Myocytes<br>Neural prog.<br>Premature neurons
E11.5		Mesenchyme<br>Radial glia<br>Fb, Md, Hb<br>Mb-Hb boundary<br>Neuroblasts<br>Fibroblasts		Basal keratinocypes<br>Chondrocyte prog.<br>Chondro-committed mesenchyme<br>Myocytes<br>Tendon prog.<br>Tendon-committed mesenchyme<br>Tendon	Chondrocyte prog.<br>Jaw/tooth prog.<br>Neural tube<br>Notochord cells<br>Oligodendrocyte prog.<br>Radial glia<br>Chondrocyte/osteoblasts<br>Connective tissue prog.<br>Granule neurons<br>Inhibitory neurons prog.<br>Inhibitory neurons<br>Isthmic organizer cells<br>Myocytes<br>Neural prog. Cells<br>Premature neurons<br>Cholinergic neurons<br>Excitatory neurons<br>Inhibitory interneurons
E12.5		Radial glia (Fb, ventral Mb)<br>Fb, Mb, Hb<br>Mb-Hb boundary<br>Neuroblasts (Mb)<br>Glutamat. neuroblasts (Fb)<br>Glycin. neuroblasts (Hb)<br>Glutamat. neuroblasts (Hb)<br>Fibroblasts<br>Neurons (Fb, Mb, Hb)<br>GABAer. neurons (Fb)<br>Glutamat. neurons (Mb)<br>Intermediate prog.			Chondrocyte prog.<br>Jaw/tooth prog.<br>Neural tube<br>Notochord cells<br>Oligodendrocyte prog.<br>Radial glia<br>Granule neurons<br>Neural prog. Cells<br>Inhibitory interneurons
E15.5		Radial glia (dorsal Fb)<br>GABAer. neuroblasts (Fb)<br>Fibroblasts<br>Neurons (Fb, Mb, Hb)<br>GABAer. neurons (Fb, Mb, Hb)<br>Glutamat. Neurons (Fb, Mb, Hb)<br>Intermediate prog. (Fb)		Chondrocyte prog.<br>Chondro-committed mesenchyme<br>Tendon prog.<br>Tendon	
E18.5		Neuroblasts (Hb)		Chondrocyte prog.	

		Glutamat cortical/hippoc. neurons<br>Glutamat. Neurons (Mb)<br>GABAer. neurons (Fb)<br>Glycin. Neurons (Hb)		Chondro-committed mesenchyme<br>Tendon prog.<br>Tendon	

MN1-expressing cell types and tissues according to single-cell transcriptomic profiles during mouse embryogenesis. Since the information was acquired from online interactive resources, normalization between datasets was not performed. Ant, anterior; CNS, central nervous system; Diff, differentiating; Fb, forebrain; GABAer, gabaergic; Glutamat, glutamatergic; Glycin, glycinergic; Hb, hindbrain, Mb, midbrain; prog, progenitors.

Table S5: *MN1*-expressing cell types in the developing zebrafish.

	**Raj *et al.*** (9)	**ZebraHub** (10)		**Tambalo *et al.*** (11)	**Wagner *et al.*** (12)	**Liu *et al.*** (13)	
	Mn1a & *Mn1b*	*Mn1a*	*Mn1b*	*Mn1a*	*Mn1a*	*Mn1a*	*Mn1b*
10hpf		Neural keel (Mb, Hb)<br>Hb. neural plate<br>Ant. neural plate<br>Paraxial mesoderm	Neural keel (Mb, Hb)<br>Hb. neural plate<br>Mesoderm<br>Ectodermal cells		Neural crest<br>Spinal cord<br>CNS (Fb, Mb, Hb)	Neural plate<br>Ant. neural keel	Neural plate<br>Ant. neural keel<br>Mesoderm
12hpf	Hb, Mb<br>Neural crest<br>Head mesenchyme<br>Mesoderm<br>Diencephalon<br>Mb-Hb boundary	Neural keel (Hb)<br>Mb-Hb boundary<br>Telencephalon<br>Neural crest	Neural keel (Hb)<br>Mb-Hb boundary<br>Neural keel<br>Paraxial mesoderm			Neural crest<br>Neural rod	Neural crest<br>Neural rod<br>Ant. neural rod<br>Lateral plate mesoderm
14hpf	Hb, Mb<br>Neural crest<br>Head mesenchyme<br>Mesoderm<br>Mb-Hb boundary<br>Progenitors (Mb, Hb)<br>Hb. Up. rhombic lip<br>Otic placode<br>Epibranchial placode	Mb-Hb boundary<br>Presumptive diencephalon<br>Neural crest	Mb-Hb boundary<br>Neural keel<br>Paraxial mesoderm		Neural crest<br>Spinal cord<br>CNS (Fb, Mb, Hb)		
16hpf	Hb, Mb<br>Neural crest<br>Head mesenchyme<br>Mesoderm<br>Diencephalon<br>Hb. Up. rhombic lip<br>Otic placode<br>Epibranchial placode	Mb-Hb boundary<br>Neural crest<br>Telencephalon<br>Head mesenchyme	Mb-Hb boundary<br>Neural keel<br>Neural crest<br>Head mesenchyme	Progenitors<br>Neural crest<br>Mesendoderm<br>Pharyngeal arch			
18hpf	Hb, Mb, Fb<br>Neural crest<br>Head mesenchyme<br>Mesoderm<br>Mb-Hb boundary<br>Hb. Up. rhombic lip<br>Otic placode<br>Pharyngeal arch<br>Somites				Neural crest<br>Spinal cord<br>Midbrain<br>Hindbrain<br>Diff. neurons<br>Pharyngeal arch	CNS<br>Neurons<br>Otic placode<br>Neural crest	CNS<br>Neurons<br>Otic placode<br>Neural crest<br>Periderm
24hpf	Hb, Mb, Fb<br>Head mesenchyme<br>Mesoderm<br>Pharyngeal arch<br>Progenitors<br>Cartilage	Mb-Hb boundary<br>Head mesenchyme<br>Hindbrain<br>Diencephalon<br>Otic vesicle<br>Somites	Head mesenchyme<br>Diencephalon<br>Otic vesicle<br>Somites<br>Neurons	Pharyngeal arch<br>Otic vesicle<br>Ventral midbrain	Midbrain<br>Hindbrain<br>Diff. neurons<br>Pharyngeal arch<br>Mesoderm<br>Diencephalon<br>Telencephalon	Spinal cord<br>CNS<br>GABA. neurons<br>Neural stem cells<br>Otic vesicle<br>Pharyngeal arch	Spinal cord<br>CNS<br>Neural stem cells<br>Otic vesicle<br>Pharyngeal arch<br>Notochord
2dpf	Mesoderm<br>Pharyngeal arch<br>Cartilage<br>Radial glia<br>Differentiating neurons<br>Glutamat. Neurons<br>Glial progenitors	Muscle cells<br>Hypothalamus<br>Pharyngeal arch<br>Optic tectum<br>Pectoral fin cartilage<br>Chondrocranium<br>Neurons<br>Mesenchyme<br>Diencephalon	Muscle cells<br>Pharyngeal arch<br>Optic tectum<br>Pectoral fin cartilage<br>Chondrocranium<br>Neurons<br>Mesenchyme<br>Diencephalon<br>Somites<br>Radial glia cells				
3dpf	Mesoderm<br>Pharyngeal arch<br>Glia progenitors	Pharyngeal arch<br>Optic tectum	Pharyngeal arch<br>Pectoral fin cartilage				

	Retina, cones<br>Otic vesicle<br>Dorsal habenula	Pectoral fin cartilage<br>Neurons<br>Mesenchymal cells<br>Radial glia cells<br>Head mesenchyme<br>Forebrain<br>Photoreceptor cells	Neurons<br>Mesenchymal cells<br>Diencephalon<br>Radial glia cells<br>Head mesenchyme<br>Telencephalon<br>Forebrain			
5dpf	Cartilage<br>Retina, cones<br>Otic vesicle<br>Dorsal habenula<br>Muscle cells	Pharyngeal arch<br>Pectoral fin<br>Cartilage element<br>Mesoderm<br>Myotome<br>CNS	Pharyngeal arch<br>Cartilage element<br>Mesoderm<br>CNS<br>Neurons<br>Radial glia cells			

MN1-expressing cell types and tissues according to single-cell transcriptomic profiles during zebrafish embryogenesis. Since the information was acquired from online interactive resources, normalization between datasets was not performed. Ant, anterior; CNS, central nervous system; Diff, differentiating; dpf, days post-fertilization; Fb, forebrain; Hb, hindbrain; hpf, hours post-fertilization; Mb, midbrain; Up, upper.

Table S6: *MN1*-expressing cell types during human development.

	**Han *et al.*** (14)	**La Manno *et al.*** (15)	**Rayon *et al.*** (16)
4-7 weeks			Central nervous system prog.<br>Central nervous system neurons<br>Dorsal interneurons (dl1-6)<br>Ventral interneurons (V0-3)<br>Motor neurons
7-26 weeks	Mesenchymal prog.<br>Neurons	Radial glia cells<br>GABAergic neurons<br>Floor plate<br>Dopaminergic neurons<br>Neuroblasts<br>Serotoninergic neurons<br>Oligodendrocyte precursor cells<br>Oculomotor and trochlear nucleus	

MN1-expressing cell types and tissues according to single-cell transcriptomic profiles during human embryogenesis. Since the information was acquired from online interactive resources, normalization between datasets was not performed. Prog, progenitor.

Table S7: *MN1*-expressing cell types during cephalochordate development.

	**Grau-Bové *et al.***<br>*(B. lanceolatum)* (17)	**Dai *et al.***<br>*(B. floridae)* (18)	**Ma *et al.***<br>*(B. floridae)* (19)
Blastula (B)		Animal pole<br>Animal-vegetal border<br>Vegetal pole	
Early gastrula (G0)		Neural ectoderm<br>Epithelial neural border anterior<br>Epithelial ectoderm ventrolateral<br>Epithelial ectoderm	
Mid-gastrula (G4)		Epithelial head<br>Epithelial ectoderm<br>Neural	
Early neurula (N0)		Neural<br>Epithelial ectoderm<br>Ventral mesoderm down<br>Endoderm<br>Epithelial	
Mid-neurula (N4)	Anterior epidermis<br>Posterior epidermis<br>Epidermal/neural 1<br>Neural<br>Endoderm<br>Endostyle / Club-shaped gland	Endoderm<br>Mesoderm<br>Neural (CNS)	
Late neurula (T1)		Endoderm<br>Mesoderm<br>Epithelial ectoderm<br>Neuromesoderm<br>Neural (CNS)	

proto-MN1-expressing cell types and tissues according to single-cell transcriptomic profiles in different species of cephalochordates during embryogenesis. The *proto-MN1* gene was filtered out from the final gene-cell matrix in Ma *et al.* (19) either due to its low expression levels or insufficient sequencing depth. Since the information was acquired from online interactive resources, normalization between datasets was not performed. CNS, central nervous system.

Table S8: Chi-square statistical comparison of the Mendelian proportions in the new *Mn1* mutant mouse line.

Developmental stages	Genotype	Observed	Expected	Chi-square
E9.5-E13.5	*Mn1*$^{-/-}$	21	20	X^2=0.92<br>Df=2
	Mn1$^{+/-}$	34	38	
	Mn1$^{+/+}$	23	20	
		Total 78		
E15.5-E18.8	*Mn1*$^{-/-}$	16	21	X^2=1.62<br>Df=2
	Mn1$^{+/-}$	45	41	
	Mn1$^{+/+}$	22	21	
		Total 83		
P21	*Mn1*$^{-/-}$	4	16	X^2=12.7225<br>Df=2
	Mn1$^{+/-}$	44	33	
	Mn1$^{+/+}$	17	16	
		Total 65		

Detailed information about the statistical comparison by Chi-square of the observed Mendelian proportions in the new *Mn1* mutant line. α (0.05) = 5.99. Df, degrees of freedom.

Table S9: Comparison of bones affected in the original and the newly created *Mn1* mutant lines.

Embryonic origin	Ossification	Bones	*Mn1*$^{-/-}$ (this study)	*Mn1*$^{-/-}$ (Meester-Smoor and colleagues) (20)
Cranial neural crest cells	Intramem.	Alisphenoid	Del.Ossf.<br>Misshaped	Ag
		Frontal	Hy	Hy
		Mandible	N	N
		Maxilla	N	N
		Nasal bone	N	N
		Palatine	Hy	Hy
		Premaxilla	N	N
		Pterygoid	Del.Ossf.<br>Hy	Hy
		Squamosal	Misshaped	Ag
		Tympanic ring	N	N
		Vomer	Del.Ossf.<br>Misshaped	Ag
	Endochon.	Presphenoid	N*	Hy
Mesoderm	Intramem.	Exoccipital	N	N
		Interparietal	Del.Ossf.<br>Hy	Hy
		Parietal	N	Hy
		Petrosal and middle ear	N	N
		Supraoccipital	Del.Ossf.<br>Misshaped	Hy
	Endochon.	Basioccipital	N	N
		Basisphenoid	Del.Ossf.	Hy

The bones affected in the newly generated *Mn1*$^{-/-}$ line match the affected bones from the *Mn1* mutant line from Meester-Smoor and colleagues (20). The zygomatic bone was segmented and analyzed together with the maxilla (dark blue in Fig. S12). *The presphenoid bone shows enlarged wings in the newly generated *Mn1*$^{-/-}$ mice. Ag, agenic; Del.Ossf, delayed ossification; Endochon, endochondral; Hy, hypoplastic; Intramem, intramembranous; N, normal development.

Table S10: Differentially expressed genes summary.

Genes	Up.	Down.	Brief description
Fam83b	yellow		Potential proto-oncogene involved in EGFR signaling
Gprin3	yellow		Vertebrate-specific G protein thought to be involved in neurite growth. *Gprin3*$^{-/-}$ mice show dopamine-dependent behavior abnormalities
Kcna1	yellow		Voltage-gated potassium channel. *Kcna1*$^{-/-}$ mice display frequent seizures and are used as models for human epilepsy
Ofcc1	yellow		Long non-coding RNA proposed to be involved in eye and craniofacial development. *Ofcc1*$^{-/-}$ mice show asymptomatic hyper-γ-glutamyl-transpeptidasemia
Plcd4	yellow		Phospholipase enzyme. *Plcd4*$^{-/-}$ mice are either sterile or produce fewer smaller litters
Sv2b	yellow		Regulates vesicle trafficking, exocytosis and synaptic protein content
Cyp26a1		yellow	Negative enzymatic regulator of the retinoic acid signaling pathway. *Cyp26a*$^{-/-}$ mice are embryonically lethal
Fendrr		yellow	Long non-coding RNA involved in heart, lungs and gastrointestinal development. *Fendrr*$^{-/-}$ mice are embryonically or perinatally lethal
Olfr77		yellow	Olfactory receptor implicated in smell
Pdzrn4		yellow	Predicted to promote metal ion binding. *Pdzrn4* knock-down indicate this gene to be a suppressor of prostate cancer growth and development
Six2		yellow	Transcription factor involved in craniofacial and renal development. *Six2*$^{-/-}$ mice exhibit shortened snout and alterations of the cranial base
Tbx15		yellow	Transcription factor playing a role in limb, vertebral column and head development. *Tbx15*$^{-/-}$ display shorter faces.
Tbx18		yellow	Transcriptional repressor involved in vertebral column, ureter and heart development.
Itgam	red		Integrin important for monocytes and neutrophiles adhesion to the activated endothelium
Cyp26c1		red	Negative enzymatic regulator of the retinoic acid signaling pathway. *Cyp26c*$^{-/-}$ mice survive to adulthood without any alterations due to functional redundancy with *Cyp26a1*
Cemip		red	Involved in Wnt/β-catenin signaling pathway and hyaluronic acid degradation. *Cemip*$^{-/-}$ mice show spatial memory impairment
Inhba	green		Activin associated with tumor aggressiveness
Snurf	green		Involved in pre-mRNA splicing. Located in chromosome 15 within the Prader-Willi Syndrome region
Pirt		green	Phosphoinositide interacting regulator. *Pirt*$^{-/-}$ mice have impaired response to cold
Lars2	blue		Synthetase which catalyzes the attachment of leucine to its cognate tRNA. *larsb*$^{-/-}$ zebrafish show liver failure and anemia, together with increased autophagy
Rmrp	blue		Long non-coding RNA involved in human ribosomopathies and cancer development
Scarna2	blue		Small Cajal body-specific RNA
Cryba1		blue	Component of the eye lens. *Cryba1*$^{-/-}$ develop congenital cataract and persistence of fetal vascularization
Gje1		blue	Gap junction protein predicted to be involved in cell-cell signaling
Kcp		blue	Regulates the expression and function of BMPs. *Kcp*$^{-/-}$ mice aggravate cardiac aging
Mn1	yellow, red, green, blue		Transcriptional coactivator involved in craniofacial development. *Mn1*$^{-/-}$ show impaired intramembranous ossification
Mctp1		yellow, red	Proposed calcium sensor located at the cell membrane. *Mctp1*$^{-/-}$ mice show no significant phenotype
Cyp26b1		green, red	Negative enzymatic regulators of the retinoic acid signaling pathway. *Cyp26b1*$^{-/-}$ mice are embryonically lethal

A total of 28 statistically significant DEGs were retrieved from the RNA-seq analysis. Colour code: yellow E9.5, red E10.5, green E12.5 brains, blue E12.5 craniofacial structures. Up, upregulated; Down, downregulated.

Table S11. Summary of HCR probes used in this study.

Species	Gene	Accession number	Genome assembly	Obtained from
House mouse (*M. musculus*)	*Mn1*	NM_001081235.3	GCF_000001635.27	Molecular Instruments
House mouse (*M. musculus*)	*Cyp26b1*	NM_001177713.1	GCF_000001635.27	Molecular Instruments
House mouse (*M. musculus*)	*Cyp26a1*	XM_017318053.2	GCF_000001635.27	HCR 3.0 probe maker + IDT
House mouse (*M. musculus*)	*Cyp26c1*	XM_017318259.3	GCF_000001635.27	HCR 3.0 probe maker + IDT
House mouse (*M. musculus*)	*HoxA1*	NM_010449.5	GCF_000001635.27	HCR 3.0 probe maker + IDT
House mouse (*M. musculus*)	*HoxA2*	NM_010451.3	GCF_000001635.27	HCR 3.0 probe maker + IDT
House mouse (*M. musculus*)	*HoxA3*	NM_010452.4	GCF_000001635.27	HCR 3.0 probe maker + IDT
House mouse (*M. musculus*)	*HoxB1*	NM_008266.6	GCF_000001635.27	HCR 3.0 probe maker + IDT
House mouse (*M. musculus*)	*HoxB2*	NM_134032.2	GCF_000001635.27	HCR 3.0 probe maker + IDT
Chicken (*G. gallus*)	*MN1*	XM_040684745.2	GCF_016699485.2	Molecular Instruments
Zebrafish (*D. rerio*)	*mn1a*	XM_695830.9	GCF_000002035.6	Molecular Instruments
Zebrafish (*D. rerio*)	*mn1b*	XM_001919740.7	GCF_000002035.6	Molecular Instruments
Small-spotted catshark (*S. canicula*)	*MN1*	XM_038780887.1	GCF_902713615.1	Molecular Instruments
Arctic lamprey (*L. camtschaticum*)*	*MN1-like*	XP_032835998.1	GCA_018977245.1	HCR 3.0 probe maker + IDT
Arctic lamprey (*L. camtschaticum*)*	*WNT1*	XP_032822233.1	GCA_018977245.1	HCR 3.0 probe maker + IDT
Arctic lamprey (*L. camtschaticum*)*	*CYP26B1C1A*	XP_032808017.1	GCA_018977245.1	HCR 3.0 probe maker + IDT
Purple sea urchin (*S. purpuratus*)	*proto-MN1*	XP_003727909.1	GCA_000002235.3	HCR 3.0 probe maker + IDT
Purple sea urchin (*S. purpuratus*)	*SOXC*	XM_792991.5	GCA_000002235.4	HCR 3.0 probe maker + IDT

*The arctic lamprey genome is poorly annotated and, hence, there is no entry for the genes. We provide the accession number for the sea lamprey sequences used to BLASTn and obtain the arctic lamprey homologs.

Supplementary Methods

Embryo collection and fixation

Mice (*M. musculus*) were mated overnight, and the presence of a vaginal plug was checked the following morning. The noon of the day of the positive plug was considered embryonic day E0.5. Mice were sacrificed by cervical dislocation, and embryos were dissected under a stereomicroscope in sterile ice-cold 1X Dulbecco's Phosphate Buffered Saline (PBS) (Sigma D5652). The embryos were fixed in freshly prepared 4% paraformaldehyde (PFA, in PBS) at 4°C for 1-24h on slow rotation, depending on the developmental stage and experimental application. For whole-mount immunofluorescence and *in situ* hybridization, samples were dehydrated in increasing methanol series (25%, 50%, 75% and 100%) in 0.1% PBS-Tween (PBST) (Tween 20; Sigma P9416) at 4°C on a slow rotation and stored in methanol at -20°C till further use. Embryos for µCT scanning were prepared according to the protocol described below.

Fertilized chicken eggs were incubated at 37°C in a humidified incubator till the desired Hamburger-Hamilton (HH) stages were reached (21). Zebrafish embryos from the AB genetic background were collected early in the morning after an overnight group mating of adult zebrafish in a breeding tank with a mesh at the bottom. Embryos were manually dechorionated and raised in embryo water at 28°C in a humidified incubator till they reached the desired developmental stage (22). Embryos were euthanized by a tricaine (Ethyl-3-aminobenzoat-methansulfonat, Merck, E10521) overdose, fixed and processed as described above. Small-spotted catsharks were housed at Ozeaneum in a 3800L recirculating aquaculture system where they can mate *ad libitum*. Deposited eggs were collected and shipped to the facilities of the Max Planck Institute for Evolutionary Biology, where they were maintained at 17-18°C in seawater with constant oxygen supply till the required embryonic stages were reached (23). Catshark embryos were removed from the eggs and euthanized by an overdose of tricaine.

Chicken, zebrafish and catshark embryos were fixed and processed similarly to the mouse samples, as described above. All embryos were collected from different breeding pairs (mouse, catshark) or egg batches (chicken, zebrafish) to ensure replication of results from a variety of breeding. All embryology work was performed under the current animal guidelines complied with Directive 2010/63/EU and the German Animal Welfare Act (§ 4(3) TierSchG).

The collection of chicken (*G. gallus*), zebrafish (*D. rerio*) and small-spotted catshark (*S. canicula*) embryonic stages coming from the first and second third of the embryonic development does not require an ethical permit. Short-term housing of the eggs has been approved by a local veterinary officer (Veterinäramt Kreis Plön). Fertilized chicken eggs were purchased from LOHMANN Deutschland Gmbh & Co. KG (Ankum, Germany). Fertilized zebrafish eggs were provided by Prof. Dr. Olivia Roth and Dr. Ralf Schneider (Christian-Albrechts-Universität zu Kiel, Germany). Fertilized small-spotted catshark eggs were provided by Dr. Timo Moritz and Ann-Katrin Koch (Ozeaneum, Deutsches Meeresmuseum, Stralsund, Germany). Fixed arctic lamprey embryos (*L. camtschaticum*) were provided by Dr. Juan Pascual-Anaya (University of Málaga, Spain). Fixed purple sea urchin embryos (*S. purpuratus*) were provided by Dr. Marian Hu (Christian-Albrechts-Universität zu Kiel, Germany).

Genotyping strategy for the newly generated *Mn1* mutant mouse strain

Genotyping was performed by lysing tissue from weaned pup earmarking or embryonic tail clippings in 45µl DirectPCR Lysis Reagent (Viagen 102-T) containing 5µl of 10mg/ml Proteinase K (Sigma P6556) at 55°C overnight. Reaction was neutralized by 20 min incubation at 95°C and directly used for PCR as follows: denaturation 95°C 15min; 35 cycles of 95°C for 30s, 63°C for 30s, and 72°C for 1min; elongation 72°C for 10min. PCR primers were designed to discriminate between wild-type and knock-out alleles by hybridizing in the mutated region: *Mn1*_WT_F (5'-TCCTACTGGCCCCGTGGA-3'), *Mn1*_KO_F (5'-TTCCACGCCGGGCCTCCCTGCA-3'), and *Mn1*_R (5'-TTCCATAGTTCGGGCACCTC-3').

Phylogenetic analyses

The following 17 species were selected for the group-scale synteny analysis: human (*H. sapiens*), house mouse (*M. musculus*), chicken (*G. gallus*), common wall lizard (*P. muralis*), Western clawed frog (*X. tropicalis*), African lungfish (*P. annectens*), coelacanth (*L. chalumnae*), zebrafish (*D. rerio*), spotted gar (*L. oculatus*), American paddlefish (*P. spathula*), gray bichir (*P. senegalus*), small-spotted catshark (*S. canicula*), brown hagfish (*E. atami*), sea lamprey (*P. marinus*), Florida lancelet (*B. floridae*), Hawaiian acorn worm (*P. flava*) and green sea urchin (*L. variegatus*).

The *MN1* gene structure from 29 representative species (17 vertebrates and 12 invertebrates) was retrieved from Ensembl or NCB and compared against each other. The species include the above-mentioned ones, without the brown hagfish, and: Atlantic hagfish (*M. glutinosa*), Inshore hagfish (*E. burgeri*), Far Eastern brook lamprey (*L. reissneri*), brook lamprey (*L. planeri*), Belcher's lancelet (*B. belcheri*), European lancelet (*B. lanceolatum*), acorn worm (*S. kowalevskii*), crown-of-thorns starfish (*A. planci*), bat starfish (*P. miniata*), common starfish (*A. rubens*), painted urchin (*L. pictus*), purple sea urchin (*S. purpuratus*) and *A. japonica.* Due to genome duplication in zebrafish and American paddlefish, both paralogs were included in the analysis; the two *proto-MN1* predicted isoforms of the acorn worm and crown-of-thorns starfish were analyzed. A total of 33 sequences were analyzed.

The following parameters were used to run GENESPACE: ploidy=1, blkSize=1, blkRadius=50, nGaps=150, nSecondaryHits=0, synBuff=1000. The brown hagfish was selected for the group-scale synteny analysis while the Atlantic hagfish (*M. glutinosa*) was used for any other single-gene evolutionary analyses. The brown hagfish genome and its annotation files were obtained from Zenodo (https://zenodo.org/records/10227719).

In situ hybridization chain reaction (HCR)

Fixed embryos were stored at -20°C in methanol for at least 24h prior to staining. To reduce autofluorescence, samples were bleached in Dent's Bleach solution (2 volumes Dent's Fix solution, 1 volume Vaprox® – Steris PB006EUR) overnight at 4°C on slow rotation. Samples were washed in 100% methanol and incubated overnight at 4°C in Dent's Fix solution (80% methanol, 20% DMSO – Roth A994.1). Subsequently, the samples were rehydrated in decreasing methanol series (75%, 50%, 25%, 0%) in 0.1% PBST, post-fixed 20min at room temperature (RT) in 4% PFA, pre-hybridized in 30% probe hybridization buffer for 30min at 37°C and incubated in 2 pmol HCR probes diluted in 30% hybridization buffer overnight at 37°C. Samples were then washed 4x15 min in 30% probe wash buffer at 37°C and 0.1% 5X SSC-Tween (SSCT) at RT (20X SSC; Fisher bioreagents

BP1325-4) and incubated in 30 pmol of fluorescent-labelled hairpins diluted in amplification buffer at RT overnight, protected from light. The nuclear stain was performed by 1X SYBR™ Gold Nucleic Acid Gel Stain solution (Invitrogen S11494) or 1X DAPI solution (4′,6-diamidino-2-phenylindole; ThermoFisher D21490). Finally, samples were washed for 10 min in 0.1% PBST and cleared using either BABB (24) or iDISCO (https://idisco.info/), depending on sample size.

Immunofluorescence staining

After bleaching, embryos were washed 3x20 min in 0.1% PBST and incubated in primary antibodies diluted in blocking solution (20% DMSO, 5% donkey normal serum (Interchim UP77719A-K) in PBS), rotating at RT for 5-7 days, depending on the sample size. After 3x20min washes in 0.1% PBST, embryos were incubated in secondary antibodies diluted in blocking solution, rotating at RT for 3 days. Samples were cleared using BABB and imaged using Zeiss LSM980 with Airyscan2 confocal microscope. DAPI was used to stain the nuclei.

Bulk mRNA sequencing

The embryonic tissues were disrupted using a sterile plastic pestle in RLT buffer containing 1% β-mercaptoethanol (Gibco 31350-010) and homogenized using a 20-gauge needle (B|Braun 4606108V). Total RNA was extracted using the RNeasy Mini kit (Qiagen 74004). On-column DNase digestion was performed using the RNase-Free DNase Set (Qiagen 79254), following the manufacturer's protocol. Subsequent steps and sequencing were performed by Novogene Co. Ltd (Cambridge, UK).

Micro-computed tomography (μCT) analysis

After fixation in 4% PFA, embryos were stored in 30% ethanol till scanning. Eight individuals per genotype (wild-type *Mn1*$^{+/+}$, heterozygous *Mn1*$^{+/-}$, knock-out *Mn1*$^{-/-}$) and developmental stage (E15.5 and E18.5) were analyzed. Samples were mounted in 0.5% agarose (Lonza 50004) in thin-wall polyethylene tubes to avoid movement artifacts, and scanned using the Bruker SkyScan 1276 with 60kV acceleration voltage and 200μA current, filtered by a 0.5mm aluminium plate. The exposure time of 572 ms, 900 projections over 360° and averaging=4 were used for scanning. The voxel resolution of the reconstructed data was 6.3µm and 10µm for E15.5 and E18.5 embryos, respectively.

Adult mice were scanned using the same settings as for the embryonic samples with the following modifications: 239 ms exposure time, 520 projections and averaging=2. The voxel resolution for the reconstructed adult mice data was 40.916µm. Tomographic reconstructions and segmentation of the skeletal elements were performed as for the embryonic stages.

SI References

1. A. J. Massri, *et al.*, Developmental single-cell transcriptomics in the Lytechinus variegatus sea urchin embryo. *Development* **148**, dev198614 (2021).

2. E. Anishchenko, M. I. Arnone, S. D'Aniello, SoxB2 in sea urchin development: implications in neurogenesis, ciliogenesis and skeletal patterning. *EvoDevo* **9**, 5 (2018).

3. L. M. Angerer, S. Yaguchi, R. C. Angerer, R. D. Burke, The evolution of nervous system patterning: insights from sea urchin development. *Development (Cambridge, England)* **138**, 3613 (2011).

4. B. Pijuan-Sala, *et al.*, A single-cell molecular map of mouse gastrulation and early organogenesis. *Nature* **566**, 490–495 (2019).

5. G. La Manno, *et al.*, Molecular architecture of the developing mouse brain. *Nature* **596**, 92–96 (2021).

6. R. Soldatov, *et al.*, Spatiotemporal structure of cell fate decisions in murine neural crest. *Science* **364**, eaas9536 (2019).

7. N. H. Kelly, N. P. T. Huynh, F. Guilak, Single cell RNA-sequencing reveals cellular heterogeneity and trajectories of lineage specification during murine embryonic limb development. *Matrix Biol* **89**, 1–10 (2020).

8. J. Cao, *et al.*, The single-cell transcriptional landscape of mammalian organogenesis. *Nature* **566**, 496–502 (2019).

9. B. Raj, *et al.*, Emergence of Neuronal Diversity during Vertebrate Brain Development. *Neuron* **108**, 1058-1074.e6 (2020).

10. M. Lange, *et al.*, Zebrahub – Multimodal Zebrafish Developmental Atlas Reveals the State-Transition Dynamics of Late-Vertebrate Pluripotent Axial Progenitors. [Preprint] (2023). Available at: https://www.biorxiv.org/content/10.1101/2023.03.06.531398v2 [Accessed 23 July 2024].

11. M. Tambalo, R. Mitter, D. G. Wilkinson, A single cell transcriptome atlas of the developing zebrafish hindbrain. *Development* **147**, dev184143 (2020).

12. D. E. Wagner, *et al.*, Single-cell mapping of gene expression landscapes and lineage in the zebrafish embryo. *Science* **360**, 981–987 (2018).

13. C. Liu, *et al.*, Spatiotemporal mapping of gene expression landscapes and developmental trajectories during zebrafish embryogenesis. *Dev Cell* **57**, 1284-1298.e5 (2022).

14. X. Han, *et al.*, Construction of a human cell landscape at single-cell level. *Nature* **581**, 303–309 (2020).

15. G. La Manno, *et al.*, Molecular Diversity of Midbrain Development in Mouse, Human, and Stem Cells. *Cell* **167**, 566-580.e19 (2016).

16. T. Rayon, R. J. Maizels, C. Barrington, J. Briscoe, Single-cell transcriptome profiling of the human developing spinal cord reveals a conserved genetic programme with human-specific features. *Development* **148**, dev199711 (2021).

17. X. Grau-Bové, *et al.*, An amphioxus neurula stage cell atlas supports a complex scenario for the emergence of vertebrate head mesoderm. *Nat Commun* **15**, 4550 (2024).

18. Y. Dai, *et al.*, Evolutionary origin of the chordate nervous system revealed by amphioxus developmental trajectories. *Nat Ecol Evol* **8**, 1693–1710 (2024).

19. P. Ma, *et al.*, Joint profiling of gene expression and chromatin accessibility during amphioxus development at single-cell resolution. *Cell Reports* **39**, 110979 (2022).

20. M. A. Meester-Smoor, *et al.*, Targeted Disruption of the Mn1 Oncogene Results in Severe Defects in Development of Membranous Bones of the Cranial Skeleton. *Mol Cell Biol* **25**, 4229–4236 (2005).

21. V. Hamburger, H. L. Hamilton, A series of normal stages in the development of the chick embryo. 1951. *Dev Dyn* **195**, 231–272 (1992).

22. C. B. Kimmel, W. W. Ballard, S. R. Kimmel, B. Ullmann, T. F. Schilling, Stages of embryonic development of the zebrafish. *Dev Dyn* **203**, 253–310 (1995).

23. W. W. Ballard, J. Mellinger, H. Lechenault, A series of normal stages for development of *Scyliorhinus canicula* , the lesser spotted dogfish *(Chondrichthyes: Scyliorhinidae)*. *J Exp Zool* **267**, 318–336 (1993).

24. K. Becker, N. Jährling, S. Saghafi, R. Weiler, H.-U. Dodt, Chemical clearing and dehydration of GFP expressing mouse brains. *PLoS One* **7**, e33916 (2012).
