# Supplementary material for: Evolution of the essential gene MN1 during the macroevolutionary transition toward patterning the vertebrate hindbrain
Source: Proc Natl Acad Sci U S A. 2025 May 27;122(22):e2416061122. doi: 10.1073/pnas.2416061122 (PMC12146709; doi:10.1073/pnas.2416061122)
Supplement: Supplementary file 2 — Dataset S01 (PDF) [file pnas.2416061122.sd01.pdf]

Data S1: Multiple sequence alignment of the 106 MN1 proteins

>S.purpuratus

---MSAYSYGR------------GAASQFTGGGGRGQVSYQTNANPAAARPSSEHSYGAMAYPHIPSINE-SAE-FG-----------
------G-N--YAAPLNSYQRQSQNQS-----QR--------MN--S-NILGGDNRTFPAPN---MEAS-----------------------------------
------------------AY------------NRHSNTAMQSGY------------------SSTGASTPNRRSSVPPNVGINSLSNMQRSNSY-------
------------SGSSTDPAYSNRNSGMAQ---SSYPQPNM------------
SNQSQHYGEGFAGPYA----SQHQQSAAY---------------------------------------NRTNSMMK----SSEMWD---------------
--RTNQT---RLGQASFSNPHS-V--NTHFP-HKTASNQYH-HSGMQYPSSQTQN--TNTQMYAANNTSGMHTSA-------
-------------------------------GMNMS----GDGSTS---
TSDLGIRGNQCVNSGAM----------MGQAYGNNQAMYSSSMGARMR--------MPNQRSSVSGGGM--------------
YNSMSGGN------------------------------QVPG-----SLQKLRHYGPMPN--SSSGYSRTSTRP--------
GNMQTNVGTVRNTAGSASNTMGMQGGW------------QANNSASQ-------------SGYAQYSQNQTNMSTNVPF--
NGLPHNSNMQ---IQQQPM---SYNNNMGMSNQQ-------------QQT----------
PKPQNFSGSVQ----PSPSMDNLSAPSGVYA---PSAPN----------------------TGSAPY--------------------
PSTACPEKVDSSS--AEFPNNISAAETEAMIQQLN--------QDRIFSAELEKLARLSR----------NP--TAEDFIADP-TG--
ATPVSGTGEPAKSSCAGSSTFQSPTSIDELLDGGGCVASSAGTENPQ--CVVNSNVNLSHANSH-----
MQSPSASVSG-SFQSGSGVVANQ---SQSDQGASSS-SAPHTPNSTANAGKSAQNKAR-----------------
LKSDKVQDCDINQGKAVEGN---ENSVHHLQRMT-Q--------SIKDTSKQDDLMKVANSQHAEYLSHSS--SDN-
YGSQNCIAALSAACRNIIADMDSSMPKQNSGQKVQSPLGGKN---PKFDGASGFGSKSDALSGFGSGPPSV----------
---------NSMGDQFV-----APSSCMM-----TAPPNCMPMDS-----YAAFPNNFNGA-NPM--PMPDFQ-------------------------
---------------------GKYHDFLEQSLPMQMSGRRMEE---KPRKG---
--RRRRKSDEFSTEQLI-SCSMNP--------VKPKRKYRKRSTQNPPSVESMEGPLSVDNV---VFTPLSENGS-------
HQADDM----------------GYRSAT---QTPNSAPN--SW----GMD---------------------VKMSCSDSI---SMA--MSIADG-NQI-
--EHVSQ-------DLL-DS------VF-SPDTT------MSSTTNQD----------------------------MPESGNCVPQGSSPIMHH----
NQSPLGPPHLNNNHPLSADHKHNMFPPQSNN----------TVSST---------------GE---ANC-QFISTSTHAVNG--
NVKHKD------------VEEAHPLEILQAQIKIQRKQFNLGETQQSQAA---------------KNNEKNS---------------------------
KVENAL--------PSDLEMDNLLSSEET-AWYLSE-------DQ----------PKDQVGGGGGLWEDLCPKDF-
HHCDSVVPLAPLQSKTL---

>L.planeri

MSVADSWTLGP-SPHEAWVHHGQPHGTEYIGIQQQQQQQHQQQQQ------QQQQQQQQQHAWAMGEGRL-
PADSYP--S-PLAAPNPGGYEFASMPATSCVNAEYFGIQPQPQQP--------------------QQQQGPLFSGYMSAIGDHYEPI-
--------MNAFEGGLTEQQQQQQQQ----------------------------
QQQQQQGLLFSRGGAQPGGYGAAFPHESSVYDEFGSISVAPAFP-
GPGSTPGPAANQAQGPGPGATVGPAELQLQQQQQQQQ--------------QQQQQQQQQQQQQQQQQQ-------------------
---QQPPPPAF-------
DMNDGQGHFPAAPAAPAPPTQGRGRPRKGKAAAAAAAAAAAAAAAAAAAAANAPVSSTSSVAGGGGGGGGGAGG
GAGGGGREMIPAA-AWTDVTAYE-----HQQQQQLNALHQNTQQQQ---ESSSYLFGAPSRAPLLHQQLV-
QYERGGPPAPHSALRMRGGVGGLYHPPRGGVDALHTHLTEYPQ-----------------------QQQQSQQQQQQQH--
MHLQQQQHYGHAAKRARSEFYPHGVVGEQGPWL------------ASGVAPSP-------------------------
HHPAHHPAHHHPPHHPHHHQHHQQQQPLAY---GEAVPPLSH--------------SAFPARLSPSAHH--------------
HPHHQQQQ-----------QHP----------------QHPH------SQHHQQQQQ-AAAAAASGLGFGFRKQ------------------------
KLASGTAGAHC------------GAV---YP----------TLTPPPPPAAP--PAPPQ-----PPAAAAAASSLP----EQQAQ-----------------
---------------HNNNNINNNNVNNVNNVNGLTQHGFQGRPWPLPSDMLPRKGKLLLAESAKAGVHGGAGVGN----
NGGGGNGGGAIGGVVC-D-SIQQQ----------------------QLYAQQ-------------------QQQQQQQQHQLLGG--
SVLPAGLSNLH--FGQQQQQ--------LEQQMQSSRCGQLSLES---LRMQQHQQQ--QLQQQQFMP----
QMGPLSAMMHSEMCIPSKMSPSLYAAHHHHHQQQ-----HQQQQHQQHHHHHHHQQQQQQQQQLE-----
FAMLRGSPHL-RNMQQQQQQQGGA--CYPGAQAGVAM--APHGCRAVPHAAPTG------------------------GKF------------
----
GGTLALGALALGDAGGKDAAAIAGNGGNGGGAAAGGGAGGGGGAAGGGGAMSSSFGQSCLAALSTACQNMI
ASLGAP--------SLNVTYGKRQQQQQQQAGGGGGGGGGGTPSGDGKGRGDQE------------------
ALGGDFGPLSCAVETESPAHGGTATTPTAAHQQEGA-----
GNAHTPPTHHGDAGGGVLSSPGERAPATLAAAAGGGGGGGGGGGGGGGGGGGGGGGGG-----------------
IGTSSGRGGSEIVTTGRGRGRGR---GRRKNN----
NNNIISHGGGAA--------GTVATTS-

TAAAAVTSTPASQNCSPGLRNTATPRGGGGGGGDSSASTSSQITQSAASSSSSAAPPPPPPAASAAAAPG---
PEACTSSP-GGW----ASG----HPDLPAL----------GDKQSDLLLA--SLD--SGIQSL-SPP---SDASP-----LLEFCPDP--------
-GAIDG------VISSVALAAGGQ-------------------------PPPPGGGPPGGAPPPPAA------
APAAAGASAGASSLSASSSSSALPPPPPPLPPQ------SSSSSSAGGVGGGPPHSAEMGM---SSP-
GEQQSSGQIRVQSNDVLLQQ----QQHQQQHHQHHHHQQLLQHQHQLQQQQHHQLHHHHTQQQ-------------------
--QG------------HHLTLQQ-------QHTRRV--------YASSVLETSAPSDH----HLQ---------------------PPQDVA--
MSSSSNSSSSSSSSSSCPSSSSSTPG-----------VGGGGGGGGGGSPWFVHAADKGSLATSGG---
LGWHPVPGMHCGGFGSQGSSADGE-----------------------------------

>M.glutinosa

-------MSSC-EAWALG--VGAQRAWGPA-----------------------P-----
RAPPYCAEPSGPPP-------------------------------------CPCETH---CYYPG----
TTGYPRYPGGV--------------------GDPYVPPGAATAVLPTEPET---------------------------------QGYGTLYGRGAHSVPT---
--PGPFPYALTEPCRV------PFPGYEAAASGSL------
---------------------------LG--GGDAG---GFEAA------------DSHAA-RYLAGKLR------
GQPGTANRGAPGREPGWPGDGATYSH--GLGAQPF----------------------PSPYS--------
---------------------------------------PGAALIQQPGVPSTDVM--------------------------YGTHGQ--------LGF--------------------------
-----DP--------------------------------AREP--------AQQHRGSGEFPASAKRPRADF---------------------------------LAFPGHK------
------FPTDA-AP-------------NGLPGS-----------------GGRTWGGALA---SRRG--------
KAVPCELPEAQE--SLHMFAPGMPGGFYRSQDAMALS------------------------------------
--------------NRAKQQQQQQQQQQ------------QH------------------NQSTFTHGLPSEGYPSR-----------------------------------
GLTE--------------GLPESYCMPGP-----GFQQHQQHQ-------------QPE-------FGLRASPLLL---------------------
SGCFPHSPGPLDLAGAPRGR--GSTGKLCRMSMG--------PLAV---------
HPGAGHGGGGSSEVKENAGSSYGQSCLAALSTACQNMIASLGAP--------SLNVTYGKRP---P-------PGESEARGR--
---------------------------VPGGESAPARDETPTLSTSPG-------ARVSETGT-----TDCAGPPGAAAEICG--LSSPGD-----------
--PSSGRGESRAT---RGRGR--
---GRRKGG----------VRTGSP--------GVKLAPA------------------PGDDVL--GGPGET---
GGEGLGSP--GW----GGA-------------PSDTTGAGDK-PSDLLLA--SLD--SGIQSL-SPP---SDASP-----LLEFC-AE---------
--GTVSGGNPELGV----------------G-------------------------------VIQAP---------------
-----GVGAGTPPSAGALMPQ--DHRAVGPA--------------------CSQ-------------------------
-----------------MTSMDPTHVQIVGDGIPHGLPR--------------------PDEGAG----------GFGV-LEGDGKAPVWYAD----------
----RPGAGYSWHGPALHCG----------------WQSM--

>L.reissneri

MSVADSWTLGP-
SPHEAWVHHGQPHGTEYIGIQQQQQQQHQQQQQQQQQQQQQQQHVWAMGEGRLPADSYPSPLAAP---------
NPGGYEFASMPATSCVNAEYFGIQPQPQQP--------------------QQQQGPLFSGYMSAIGDHYEPI---------
MNAFEGGLTEQQQQQQQ----------------------------
QQQQQQGLLFSRGGAQPGGYGAAFPHESSVYDEFGSISVAPAFP-GPGSTPGPAANQAQGPGP------
GATVGPAELQLQQQQQQQ---------QQQQQQQQQQQQQQQQQQ---
--QQPPPPAF-------DMNDGQGHFPAAPAAPAPPTQGRGRPRKGKAAAAAAAA--
AAAAAAAAAANAPVSSTSSVAGGGGGGGGAGGG----GREMIPAA-AWTDVTAYEHHHQQQQQQLNALHQNT-
QQQQQQQQDSSSYLFGAPSRAPLLHQQLV-QYERGGPPAPHSALRMRGGVGGLYHPPRGGVDALHTHLAEYPQ-
------------------------QQQQSQQQQQQQHMHLQQQQQQHFGHAAKRARSEFYPHGVVGEQGPWL-------------
ASGVAPPP--------------------------HHPAHHPAHHHPPHHPHPHHHQHHQQQQQPSAYGEAVPPLSH---------------
SAFPARLSPSAHH---------------HPHHQQQH--------------------------------PQHP-----HSQHQQQQQV--
AAAAAAGLGFGFRKQ-------------------------KLASGTAGAHC-------------GAV---YP------------------TLTP--PPPAA-----
PPAPPQPPAAASSLPEQQAQ----------------------------------HNNNINNN----
NVNNNNGLTQHGFQGRPWPLPSDMLPRKGKLLLAE---SAKAGVHGGAGVGNNGGGGSNGGGAIGGVVC-
DSIQQQQ--LYAQQQQQHQLLGG--SVLPAGLSNLHFGQQQQQQL--------
EQQMQSSR-CGQLSLES---LRM----QQ--HQQQQQFMP----
QMGPLSAMMHSEMCIPSKMSPSLYAAHHHHHQQQHQQHQQQHQQHHHH---HHQQQQQHQQQLEF-----
AMLRGSPHLRNMQQQQQGGACYP-----GAQAGVAM--APHGCRAVPHAAPPG-------------------------GKF---------------
-----GGTLALGALALGDAGGKDAAAIAGGGNGGGGTAAAGGGAGGGGGAAGGAMSS-
SFGQSCLAALSTACQNMIASLGAP--------SLNVTYGKRQ---QQQQQQQAGGGGGGGGTPSGDGKGRGEQE--------
--------ALGGDFGPLSCAVETESPAHGGTATTPTAAHQQEEA-----

GNAHTPPTHHGDAGGGVLSSPGERAPAALAAAAAGGGGGGGGGGGGGGGGGGGGGGGGGGGGGASGGIGT--------
----SSGRGGSEIVTTGRGRGRGR---GRRKNNNNIISH----
GGGGAA--------GTVATTSTAAAAAATSTPASQNCSPGLRNT-
ATPRGGGGGGGDSSASTSSQITQSASSSSLSSAPPPPPSAASAAAPG---PEACTSSP-GGW----ASG----HPDLPAL-----
-----GDKQSDLLLA--SLD--SGIQSL-SPP---SDASP-----LLEFCPDP---------GAIDG------VISSVALAAGG-----------------
--------QPPPGGGPPGGAPPPPAA--------AAPAAAAGASSLSASSSSSSALPPPPPPLPP------
QSSSSSAGGVGGAPPHSAEMGM---SSP-GEQQSSGQIRVQSNDVLLQQ---------
QQHHQHHHQQQLLQHQHQLQQQQHHHQLHHHHHTQ--------------QQQQQGHH------------LTLQQQQ-------
QHTRRV--------YGSSVLETSAPSDH----HLQP-------------------PQD-----VAMSSSSNSSSSSCPSSSSSTPGVGG---------
-----GGGGGGGGGSPWFVHAADKGSLATSGG---
LGWHPVPGMHCGGFGSQGSSADGESRLQCTRVRTHTRSTTHTRVSITRVLPHTHVYL

>P.marinus

MSVADSWTLGP-SPHEAWVHHGQPHGTEYVGIQQQQQQHQQQQQQQQQQQHAWAIGEGRLPADSYPSPLA-
APN-------------PGGYEFASMPATSCVNAEYFGIQPQPQQQ--------------------QQQGPLFSGYMSAIGDHYEPI---------
MNAFEGGLTEQQQQQ------------------------------
QQQQQQGLLFSRGGSQPGGYGAAFPHESSVYDEFGSISVAPAFP-GPGSTPGPAANQAQGPGPG---------------------
----------ATVGPAELQQQQQQQQQQ---QQPPPPTF-------
DMNDAQGHFPAAPAAPAPPTQGRGRPRKGKAAAAAAAAA--
AAAAAAAAAAAAAANAPVTPSVTGGGGGGPGGG----GREMIPAA-
AWTDVTAYEHHHQQQQQLNALHQNTQQQQQQQ---ESSSYLLGAPSRAPLLHQHLV-QYERGPHS----
ALRMRGGVGGLYPPRG-GVDALQTHLAEYPH-----------------------
QQQQQQQSQQQQQQHMHLQQQQQHFGHAAKRARSEFYPHGGVGEQGPWLGSGM----------------------------------
------APSPHHPAHHHPPHHPH--PHHHHQHHQHHQQPSAYGEAVPP-LSHSA--------FPARLSPSASAHH--------------
HPHHQQQQ------------------------------QQPQHPHSQQHHHHQQQQQQQAAAAAAGLGFGFRKQ------------------------
KVASGTAGAHC------------GAV---YP-----------------TLTP--PPPAP-----PPPPPPPQPPASAAASAASLLPEQQAHN-----
-------------------NNNN--------NGLTQHGFQGRP----WPLPSDMLPRKGKLLLAES---AKGAVHGGAGV----
GNNGGGGGGAIGGVVCDSIQQQQQ--QLYAQQQQQHQQQQQ--
HQLLGGSVLPA-----GLSN--------LHFGQQQQLEQQMQSSRCGQLSL----ESLRMQQQQQFMP----
QMGPLSAMMHSEMCIPSKMSPSLYAAHHHHHHQQ--QHQQQHQQHHHHHHQQQQQQQQQQQLEFA------
MLRRSPHLRNMQQQQQGGACYP-----GTQAGMAM--APHGCRAVPHAQPMGGKFG-------------------
GTLALGALALGNADGKDAAAIAGGGGNGGNGGNG--------
GGGGGGGAAAGGGAGGGGGAAGGGGGGAGGMSSSFGQSCLAALSTACQNMIASLGAP--------SLNVTYGKRQ-
--QQQQQQQQAGGAGGAGGGGGGGTPSGEGKGRGEQEAL--------GGGGDFVP--LSCAVETESPA----
HGGTATTPTGAHQQEGAGNAHTPTQHGDAGGGLLSSPADRAPGTLTAAAAAGGGGGGGGGGGGGGGGGGGGG
GGGGGGASGGIGT------------SSGRGGSEIVTTGRGRGRGR--
GRRKNNNNINNIISHGGGGGAT--------GTVATTSKAAAAAATSTSASQNCSPGLRNT-
ATPRGGGGSVGDSSASTSSQIPQSS------SSSAAPPPSAASAAPS---TEACISSPGGGW----ASG-------------
HPDLPALGDK-QTDLLLA--SLD--SGIQSL-SPP---SDASP-----LLEFC-PD------PC-AIDGV-------ISSVALSAGG--------
------------------PPPPGGGPPGGAPPAAAA-----
AAPAAAGASAGPSSLSASSSALPPPPPPLPPQSSSSSAGGVGGGPPHSTEM---------GM---TSP-
GEQQSSGQIRVQSNDVLLQQQQQHQQHQQQHHQHHHHQQQLLQHQHQLQQQHHQLHHHQQQQQQ-------------
-QGNQMSPSGHHRGVMSGQMASDHHTQQQQQGHHLSLQQQQQHTRRVYGMTTSSASSVMETSAPSDHHLQP--
-----------------PQDGAM--SSSSNSSSSTSSSSSSSSSCPSSSSS----------
TPGVGGGGGGGGGSPWFVHAADKGSLATSGG---
LGWHPVPGMHCGGFGSQGSSADGLVDEGMTSLRNGLRSLAEIPGRL-----------

>B.bombina

-------MFGL-EQFEPQ--------INSRS--------AGQGER------SF-TQPGM----NMSSHFK-SSA-FH----------AG-----G-A--P-
ASVDP-AIGAMNEPS--M-MG--------MNLNL-N--GEPY-GYH-AR-GHLDAH-------------AGGM-----QQ------------
QQP---------------VH----GFFNN---QQHH-HGHHSSHP--------HQ-HHPHFS-SNFGGPDATA-SCLHGGRLM----GY--
NNNL---GN---Q----------QAFGE--GYE-QMAEN-Q-------------------SGEGFG----------------QQ---------------------
RSGNIAEF--------QHPNTNSSNHAVPAPCLPLD----QSPNRAASFHGLP--SS---------------------SSTDTHNLE----QRR-
IHSQ-G--SVDPLE------------YNYPT-DGPSG---HFEVPVFS-PSD-S--EG----HYGAGRQA--PGG-SFSG-SSVL--
PRPPGI--VGMTKVH--P------------------------------QQ---QH--GVFFERF--GGARKMSVGMEPG--VNS-RHPLM-PQQ---
-----QQ------------------------------QTGLLARQNSCPPAITR--QQQTEANTSNANL-QDNG-PVMQ-NQHTQ--------

FEYPIHRLENRNM-------------HPY--TES---MFNM---QQAP----------P----QQPP------NQRLQHFDT-
PYINVTKRPRFEFPNN-H----G-VDNCAAWSN-----N-NIHNAGIESHL-----------SPS-T-YP------------GLPGEYTP--
QVPEG---F-PPGPTLQHPGS----DHQSL-------Q----------------------QRQN--------AAMMIKQMASRN-----------QQQR-
MR-QTN---LQQLGHHGDVN----QS-GIVHGGQVGNMSQ-P-AFDRE----------------------GGRIG------------------
NFDPQNP-HVAQEN--AWFPG----PH---PPGDIL--------QRRMGGSNLPSEPTPHD---INL----PQ--NGSNMLFRS--G--
VNRM-GIQD-S----LG-LP--GEGHVQALHSP-----GIHSQFGNN----MANLSQMQSPGGG-----VGITSTSSDR-R---
IPPEFAAP---SIGGQAGFPF-VGSNRQ-STPH-NPPGVNSSP------------------SSY--PPQSDF--QASQR----
STASKLGALSLG--------SFSK---------SNA-----------KEN-IFGQSCLAALSTACQNMIASLGAP--------
NLNVTFNKKS---Q-AEGKRKLSQTETDLN--S-TSG-N------------------NANSDYFT--GGSSQSSQVPG----
VSNANSKSTGQ------SSTSQPPQGD--TS--LSPNYN---------------------------------------I---------EA-NPGNDGK--
PVT--SGGRGR---GRRKRD----------SGHVSP--------GTYFDKY-
-----------SADSGGAIVSP-GQQSQPASSGE----------------------------SGGTAH---D-KPLTSP--SW----GKG--G---DLL-L-
---------SD-QPD-LMS--SLD--SGIQSV-TKS---DSSSP-----HVDFP-ED-VNT--TY-GNEDE------VSSSSDNN--------------
-------------ISKPNSCPLVTGSPKIQR------GEHGLLN--GQKPLGLNLLNN-----------------TTSLP----DSYGL---SS--S---
GHP-GTPGME-QVRTP---TSTST------------QDEIHPLEILQAQIQLQRQQFSISEDQPLGIK--------------NKKNDCTA---
---QNVD--SELNSCC---S---DNVKNS--------MSTIDLDSLMAEHNS-TWYMPN-------EK-SLME-----VEEDKS--
ITPWEKSKSQQT-NKEGSSQNKTSAA---------------AQNGSHLQCLSVHCTDDIGESKGRT-
SVPTWRSLHSDISNRFGTFVAALT---------------------------------

>G.evgoodei

-------MFGL-EQFEPQ--------MSSRN--------AGQGER------NF-TQAGLT----MNSHFK-SAT-FH----------SG-----G-P------
ADP-AISALAEPP--I-LG----------M---NMTGEAYG-FH-AR-GHSELH-------------AGGM------Q------------AQP------------
---LH----GFFGN---QQPH-HGHPTTHH-P------HQ-HHPHFS-GNF-GSEPSA-SCLHGGRLM----SY---NSM---GS---Q----
------QAFAE--GYE-HMAEN-Q------------------GGEGFG------Q---------Q--------------------RSGNMPDF--------Q-
HNSSASNHAVPAPCLPLD----QSPNRAASFHALP--AS--------------------SSSDSHSLE----QRR-LPNQ-G--GVDSLE---
---------YNYPS-DGPSG---HFDLPVFS-PSE-S--DGQLP-HYGAGRQV--PGGGSFPG-TSVL--PRAPGM--AGMSKVH--
P----------------------Q----------QQH--GVFFERF--GGARKMSVGMEPG--LNA-RHPLM--QQ--------QQ-------------------
-----------QTGLLARQNSCPPTIPR--QQQTEANTPNPNL-QDNG-PIMQ-NQHAQ--------FEYPIHRLENRNM-------------
-HPY--TDP---VFNM----QHP---P-------P----QQQP------NQRLQHFDA-PYMNVAKRPRFDFPGN-P----A-
VDRCASWNN-----S-LH-SAGLENHL------------SPS-A-YP------------GLPGEFTP--PVPES---F-APGPPLQHAGP----
DPQAL-----Q-------------------------QRQN--------AALMIKQMASRN-----------QQQR-LR-QPS---LQQLGHHADVG----
PSSLV-HAGPVGNMPQ-P-GFERE----------------------SGGRGP------------------NFEPQAP-HLAPDS--GWFPG----PP---
PPGELL--------PRRMGG---PAEPG--------L----PQ--NGSGLLFRP--A--V---------------------EGHVPALHSP-----
GVHAPFG-------AGLAPLQSPGAG-----VGLPSAPAER-R---PQPDFAAP---PLGGPAGFAF-GASGRA-APPHSA----
SASP-------------------GAF-PAAPPEF--P----------ASKLGALSLG--------SFAK----------PA------------KEN-
VFGQSCLAALSTACQNMIASLGAP--------NLHVTFAKKS---P-PEGKRKLGPPEPDG--------------------------
GPAPGPDFFP--G-------PAA----------KAP-------------P-----ESS--LSPGYA--P---------E-
-APAGEGKAAAAAAAAGGRGR--GRRKRD----------
SGHVSP--------GGFFDKF---------------------P------PAEGGGA----------------------------GSPGPP---D-KPLASP--SW----
AKG-----GELL------------LAEQPD-LLA--SLD--SGIQSA-SKS---DGGSP-----RGDFP-DE-PSP--AY-GHEDE------
VSSSSDSA---------------------------LAKPTRSPLLGGSPKLPR------AEHALLG--AQKPLALGLLGA------------------
AAAAP----DGYGL------GGAG-AHP-ATPGLE-QVRTP---TSTSA------------
QDEIHPLEILQAQIQLQRQQFSISEDQPLGMK--------------SKKAECPG------QNGE--GELNSCC---S---DNVKGA------
--MSTIDLDSLMAEHNS-TWYMPS-------EK-SLMEG----QEEDKP--LAPWEKSKPQNP-SKEAHDLPQNKTS-----------
--AAAQTGSHLQCLSVHCTDDMGESKGRT-AVPTWRSLHSDISNRFGTFVAALT---------------------------------

>G.flavomarginatus

-------MFGL-EQFEPQ--------MSSRN--------AGQGER------NF-TQAGLT----MSSHFK-SAA-FH----------SG-----G-P------
ADP-AISALAEPP--I-LG--------MNM---NMAGEAYG-FH-AR-GHSELH-------------AGGM------Q------------AQP--------
-------LH----GFFGS---QQPH-HGHPTTHH-P------HQ-HHPHFS-GNF-GSEPSA-SCLHGGRLM----SY---NSM---GS---Q-
---------QAFAE--GYE-HMAEN-Q------------------GGEGFG------Q---------Q--------------------RSGNMPDF--------Q-
HNSSASNHAVPAPCLPLD----QSPNRAASFHALP--AS--------------------SSSDSHSLE----QRR-LPNQ-G--GVDSLE---
---------YNYPS-DGPSG---HFDLPVFS-PSE-S--DGQLP-HYGAGRQV--PGGGSFPG-TSVL--PRAPGM--AGMSKVH--
P----------------------Q----------QQH--GVFFERF--GGARKMSVGMEPG--LNA-RHPLM--QQ--------QQ-------------------
-----------QTGLLARQNSCPPAIPR--QQQTEANTPNPNL-QDNG-PIMQ-NQHAQ--------FEYPIHRLENRNM-------------
-HPY--TDP---VFNM---QQHP---P-------P----QQQP------NQRLQHFDA-PYMNVAKRPRFDFPSN-P----A-

VDRCPSWNN-----S-LH-SAGLENHL------------SPS-A-YP------------GLPGEFTP--PVPES---F-APGPPLQHAGP----
DPQAL-----Q-------------------------QRQN--------AALMIKQMASRN-----------QQQR-LR-QPS---LQQLGHHADVG----
PSSLV-HAGPVGNMPQ-P-GFERE---------------------SGGRGP------------------SFEPQAP-HLAPDS--GWFPG----PP---
PPGELL--------PRRMGG---PAEPGPHE---LSL----PQ--NGPGLLFRP--P--V--------------------EGHVPALHSP-----
GVHAPFG-------AGLAPLQSPGGG-----VGLPSAPAER-R---PQPDFAAP---PLGGQAAFAF-GASGRA-APPHSA----
SASP------------------GAF-PAAPPEF--PAAPR----AAASKLGALSLG--------SFAK----------PA------------KEN-
VFGQSCLAALSTACQNMIASLGAP--------NLHVTFAKKS---P-PEGKRKLGPPEPDG-------------------------
GPAPGPDFFP--G---A--AAAA----------KAPPG----------AP-----ETG--LSPGYA--P-----
----E--APAGEGK---AAAAAGGRGR---GRRKRD----------
SGHVSP--------GGFFDKF--------------------P------PAEGGGA---------------------------GSPGPP---D-KPLSSP--SW----
AKG-----GELL------------LAEQPD-LLA--SLD--SGIQSA-SKS---DGGSP-----RGDFP-DE-PSP--AY-GHEDE------
VSSSSDSA---------------------------LAKPTRSPLLGGSPKLPR------PEHALLG--AQKPLALGLLGA-----------------
AAAAP----DGY--------GG---AHP-GTPGLE-QVRTP---TSTSA------------
QDEIHPLEILQAQIQLQRQQFSISEDQPLGMK-------------SKKAECPG------QNGE--GELNSCC---S---DTVKGA------
--MSTIDLDSLMAEHNS-TWYMPS-------EK-SLMEG----QEEDKP--MAPWEKSKPQNP-SKEAHDLPQNKTS----------
---AAAQTGSHLQCLSVHCTDDMGESKGRT-AVPTWRSLHSDISNRFGTFVAALT---------------------------------

>P.raffonei

-------MFGL-EQFEPQ--------GSGRS----------------------GERGGGFGPGMGSHFK-PPP-FH----------------G----
GTPGGEAGTLGALSEPASAM-LA--------MN--L-NLPGEAY-GFHASRGGHADLQ-------------QAQP--------------------
----------------VH----GFFGG---QQPS-HGAHQQPP-----------PPSHFG-SGGFGAEPSA-SCLHGGRLLSYPGGQ------------
-------------QSFAAD-GYEQHLAEGQA-------------------GADGFGQQ-----------------------------------RAGPLQDF---------
QPPQQHHNPGVPAPCLPLD----QSPNRAASFHGLP--GA--------------------ATSESHGLD----SQRRLPSQ-
AAAAVESLE------------YSYPS---GES---HFDLPVFS-PSE-A--DGQLP-HYGASRQQVPPGGGNFPGPSSAL--TRGPG-
---GLGKVH--P----------------------------QQQQQQQHGGGVFFERF--GGARKMSVGLEPS--LGAGRHPLM------------
QQPPP----------------------------PGALLARQNSCPPALPR-QQLCPEGNAANPGL-QDGG-PLLQ-GQHAQ--------
FEYPIHRLENRSL-------------QHPYGPGEP---VFSV---HHHPPA-------------QQPP------
SQRLQHFDAPPYMNVAKRPRFDSWSG--------GGS-----------GAGMHSASLDSHL------------SPSAA-YP------------
GLPGDFTP--PGPES---FQPPGADHQAALQ----QQQQ-------------------------------QRQN--------AALMIKQMAASR----
-----SQQQQQQRLR-QPS---LQQLSHH--------------HAGH---------HHHHA--
GHHHGEPAFEAQEG--AWFPA----PH---PPSA-----------------------------------AP--GAGDLLFRP--G--
AGGMPGLQEPPPP--PLRIPSGGEGHVP---SPG----SLHGQFGLS--------------------------PPSERSR--PGPPDFATQ-------
-QGFPF-GAASRQ-ATPHSASPASYGPP-------------------TDF--QPSGPP--PPPRP---PQSSSKLGALSLG--------
SFSKGSVAGGVAGPPGS---GGGGAPGPKESSGLFGQSCLAALSTACQNMIASLGAP--------NLNVTFGKKG---
APAAGSGGVGAG--------------------------------SEGPKRSKLGPAEPPE---------PGSGGPQPPAP-------APPAPGESG-----
-LSPNYS--P------------GPGPDAKAG------GGRGR---
---------------------------GRRKRD----------SGHVSP-AAGGGGGGSSFFDKY-------------GPTAGVE-----GGSPGQGAER---
-------------------------GGGTPHLHEPHKALSSPPSAW----AKG--GGGGDLLLPPPPPP-----LSEQPE-LLP--SLE----------
KA---DSCSP---RGAGDFPDEA---------GNEDQ------VSSSSDNQ------------------------------LAKGCPAGRSPVQPR-----
PGDHALLN--GQKASLAQLSLHAGSN--------------STSSP----DSY--------GG---GPPAGAPV---------------------------
QDEIHPLEILQAQIQLQRQQFSISEDQPLGLK------------
SAKKPPDGPAGAAGGGSQSGDSAELSSCCAAASAAVESGKGA--------MSTIDLDSLMAEHSA-AWYLPS-------
DK-ALLEG---QDDDDKA--LAPWEKAKPPNP-SKEAHDLPSSKSS-------------
AAAQSGSHLQCLSVHCTDDMSEAKGRT-AVPTWRSLHSDISNRFGTFVAALT----------------------------------

>Z.vivipara

-------MFGL-EQFEPQ--------GSGRSGERGGGGGGGFGPS-------------------MGSHFK-PSP-FH-----------------G----
GTPGGESGTLGALSEPSSAM-LA--------MN--L-NLPGEAY-GFHASRGGHAELQ-------------QAQP----------------------
--------------VH----GFFGS---QQPS-HGAHQPPP-------------PHFG-SGGFGSEPSA-SCLHGGRLLSYPGGQ----------------
---------QTFAAD-GYEQHLAEGQA-------------------GGDGFGQQ-----------------------------------RAGPLQDF---------
QPQQQHHNPGVPAPCLPLD----QSPNRAASFHGLP--GA--------------------ATSEPHGLD----SQRRLPSQ-
GAAAVESLE------------YSYPS---SES---HFDLPVFS-PSE-A--DGQLP-HYGASRQQVPPGGGNFPGPSSAL--PRGPG--
--GLGKVH--P----------------------------QQQQQQHGGGGGVFFERF--GSARKMSVGLEPS--LGAGRHPLM------------
QQHQPPPPP------------------------PGVLLARQNSCPPALPR-QQPCPEGNAANPGL-QDGG-PLLQ-GQHAQ--------
FEYPIHRLENRSL-------------QHPYGPGEP---VFSV---HHHPPA--------------QQPP------
NQRLQHFDAPAYMNVAKRPRFDSWSG--------GSG----------GAGMHSASLDSHL-----------SPSAA-YP------------

GLPGDFTP--PGPES---FPPPGADHQAALQ----QQQQQ-------------------------------QRQN--------AALMIKQMAASR----
--SQQQQQQQQQRLR-QPS---LQQLSHHHPGH----HHHH------------P--
GHHHGEPAFEAQEG--AWFPA----PH---PPSA------------------------------------AP--GAGELLFRP--G--
VGGMPGLQEPP---APLRIPPGGERHVP---SPG----SLHGQFGLS---------------------------PPLERSR--PGPPDFAAQ------
--QGFPF-GASSRQ-ATPHSASPASYGPP-------------------TDF-QPSGPPP--QPPRP---PQSSNKLGALSLG--------
SFSKGSVVGGVAGPPGSGASSSGAPGPKESSG-LFGQSCLAALSTACQNMIASLGAP--------NLNVTFGKKG---
APAAVSGGVGAG---------------------------------SEGPKRSKLGPAEPPE---------PGSGGPQPPAA-------APPAPGESG----
--LSPNYS---P------------GPGPDAKAG------GVRGR---
----------------------------GRRKRD----------SGHVSP-AAGAGGGGGFFDKY--------------GPPAGVE-----GGSPGQGTER--
---------------------------GGGTPQLHEPHKALSSPPSAW----AKG---GGGDLLLPLPPPPPP---LSEQPE-LLP--SLE----------
KA---DSCSP---RGAGDFPDEA---------GNEDE------VSSSSDNQ-------------------------------LAKGCPAGRSPVQPR-----
PGDHALLN--GQKASLAQLSLHAGGGSN------------STSSP----DSY--------GG-------GPPTSA----------GGPV------------
QDEIHPLEILQAQIQLQRQQFSISEDQPLGLK------------SAKKPPDGPAGAGGGSTQNGDSAELSSCC---
AAAAESGKGA--------MSTIDLDSLMAEHSA-AWYLPS-------DK-ALLEG---QDDDDKA--LAPWEKAKSLNP-
SKEAHDLPSSKTS-------------AAAQSGSHLQCLSVHCTDDMGEAKGRT-AVPTWRSLHSDISNRFGTFVAALT------
----

>C.carcharias

-------MFGL-DQFSRS---------GGQG-----------GER------NF-GQ-VALGMSGMSGHYK-SPS-FP----------GG-----S-G--S-
AMEEQ-AINPLAEPP--M-LG--------MGMSP-ALGGEQY-AFH-PR-GHSELH------------GAG-------------------PQPPP----
-----------AH----GYFPNGHHHHPHPHPHPHAHH------------H--FS-GSFCGTDPGP-SCLHGGRLLGT-PGYSSNPLS---
GQ---Q----------QAFGE--SYD-PMAES-QG-----------GGGGGGGGGEAFA------Q---------QQPP-----------------
PGRSGNLTDY--------HQHHTPSSNH---TPCLPLD----QSPNRAASFHGLP--SS--------------------SSSDAHGLE----
QRR-LQNQ-P--AVDSME------------YNYQN-DPPSG---PFDMPVFS-PSE-S--SAQLP-HYGTSRHV--PSS-NFPT-
NPAM--PRAPGM--VNLGKVH--P-----------------------------QQ---QH--GVFYERF--GNARKMSVGMEPG--VAA-
RNPLM------------QQ-------------------------------QAGLLARQNSCPPAIPR--QQQAEAGAPNSSL-QDNG-TMLQ-
SQHAP--------FEYPIQRLENRNM--------------HPY--SEP---MFNI-----------------------QQQP------NQRLQHFDA-
PYLNVAKRPRFDFPNN-H----N-VDSCATWSS-----N-SMHNASLENHL------------SPS-A-YP-------------GLPNEFSP--
PGPEG---F-PPGPPLQHPGT----DQQSL-------Q-----------------------QRQN-------MLMMFKQMVSRN------------
QRHR-MR-QPD---LQHLGHHGDVN----QN-NIVHSNQVGNMSQ-P-NFERE----------------------SGGRMP----------------
---SFDPQNP-QIGQEN--AWFPG----HH---PPGDML--------QRRMGGSAVPPDGSPHESVQMNL----QQ--
NGSSMLFRP--S--GNGL-GMQE-P----LR-MP--GDGHVQGLHSP-----GMHSQFGNN----MGNVTQMQSPSGG-----
MGLPNTATDR-R---PGPDFPGP---QMGGQPGFPF-GGPNRPA-NPHNPPPGVPPSP-------------------GNY--PPQSEF--
QPNQR----PSMSKIGSLSLG--------SFSK----------PGS------------KDNTIYGQSCLAALSTACQNMIASLGAP--------
NLNVTFNKKN---Q-NEGKRKLSQTEQDNSNGGPNNGAGGSGGSV-----GGVSSGSTGNGPEYFQ--
SNTPQNNPMGV----PGNGSGKLTTSAAQNTTQGPNQPSQPE--CN--LSPNYA---L---
------EA-IP-SESK--GQT----GRGR---GRRKRD----------
SGHVSP--------GNFFDKY-----------SAENVNPGVS-P-GQQGQSTHAGD------------------------------RGGTPH---D-
KSLTSP--SW----AKG-----NDLL------------LPDQPD-LMS--SLD--SGIQSV-TKS---DGSSP-----QVDFS-DD-VGN--NY-
GNEDE------VSSSSDNN----------------------------ISKPSR--LVTSSPKLQR------ADHGLMS--
SQKPMGHGLLNAHPNSN-------------TTSNTGPVTDSFGL---SSTGG---GHP-GTPGME-QVRTP---TSTST------------
QDEIHPLEILQAQIQLQRQQFSISEDQPLAMK--------------NKKAECTG------QNGD--TELAACG---T---DNSKAA-----
---ISTIDIESLMAEHNS-TWYMPN-------DK-AMMDS----QDEDKQ--MAPWEKAKPAST-NKEAHELSQNKAS--------
------SVQTGSHLQCLSVHCTDDLTDSKGRS-PMQTWRSLHSDISNRFGTFVAALT----------------------------------

>H.ocellatum

-------MYSL-DQFSRS---------GGGPG-----------GER------NF-SQAAAL---AMSAHYK-SPT-FP----------GA-----S-G--S-
AMDEQ-TLGPL-EPP--M-LG--------LGMNP-ALGGEAY-SFH-PRGGHSELH------
QAPAAAAAAAAGSAAAPPPGGPQQPQAPPPPPPAAAA----PPPQPQAAH----GYFPGGP--------HPHGHP------------
H--FS-GSFCGTEPGH-SCLHGGRLLGTAAAYSGNPLA---GQ---P----------HAFGD--TYD-PLGEN-QA------
GGGAAAAAAGGGPGEGFA------Q---------QAPQ-----------------VGRSGNLSDY--------HQHHTPSSNH---TPCLPLD---
-QSPNRAASFHGLP--AS---------------------SSSEAHGLE----QRR-LQSQ-P--AVDSME------------YNYQN-EPSAR---
PFDMPVFS-PSE-S--GAQLP-HYGPGRHV--PGG-SFPA-NTAM--PRTPGL--VSLGKVH--P------------------------QQ----
QQ---QH--GVFYERF--GNARKMPVGMESS--VAA-RNPLM-----------QQ------------------------------
QAGLLARQNSCPPAIPR--QQQAETGPPTSSL-QDSG-AMMP-NQHAP--------FEYPIQRLENRNM--------------HPY--
TDP---MFNI----------------------QQQP------NQRLQHFDA-PYLNVAKRPRFDFPNN-H----N-VDNCATWSS-----N-

SMHNASLENHL------------SPS-A-YP-------------GLPNEFSP--PGPEG---F-PPGPPLQHPGT----DQQSL-------Q-----------------------QRQN--------MLMMFKQMVSRN------------QRHR-MR-QPE---LQHLSHHADVN----QN-SIVHSGQVANMSQ-P-GFERE----------------------SGGRMP-------------------SFDPQNP-QMGQEN--AWFPG----HH---PSGEML--------QRRMGGSAVPPEGSPHESVQMNL----QQ--NGSGMLFRP--G--GNGL-GMQE-P----MR-MP--GDGHVQGLHSP-----GMHSQFGNS----MGNVAQMQSPSGG-----MGLPSTSTDR-R---AGPDFQGN---PMGGQPGFPF-GGPNRPS-NPHNNPPGVPPSP-------------------GNY--PPQSEY--QANQR----PSMSKIGSLSLG--------SFSK----------PGS------------KDNTIYGQSCLAALSTACQNMIASLGAP--------NLNVTFNKKN---Q-NEGKRKLSQTEQDSV--GPSSGSGG--GNV-----GGPPTGSTGNGPEYFQ--GSAQQNSQMGV----SGGGSGKLGTSTVQNSTQGPNPPSQPE--CN--LSPNYA--L---------EA-IP-SEGK--GQT----GRGR--GRRKRD----------SGHVSP--------GNFFDKY-----------SAENVNPGVS-P-GQQGQSSHAGD-----------------------------RGGTPQ---D-KSLTSP--SW----AKG-----NDLL------------LPDQPD-LMS--SLD--SGIQSV-TKS---DGSSP-----QVDFS-DD-VSN--NY-GNEDE------VSSSSDNN------------------------------IPKATRLVTSSPKLQR------ADHGLLS--AQKPLGHGMLNAHPNSN-------------TTSNTGPGADSFGL------GSTGSGHP-GTPGME-QVRTP---TSTST------------QDEIHPLEILQAQIQLQRQQFSISEDQPLAMK--------------NKKAECPG------QNGD--TELAACS---T---DNSKAA--------ISTIDIESLMAEHNS-TWYMPN-------DK-AMMDP----QDEDKQ--MAPWEKAKSAST-NKEAHELSQNKAS--------------SVQTGSHLQCLSVHCTDDLTDSKGRS-PMQTWRSLHSDISNRFGTFVAALT-----------------------------------

>C.idella.mn1b

-------MFGL-EQFGPQ--------INNRN--------FGHTEK------NF-NQPRV----SMNSHYK-SPG-FH----------AG-----G-P--Q-GTVEP-GMGPLNEPP--V-IE--------MNINM-N-GGEQYGGFQ--R-GHSELH-------------VGNL---QQQQ------------QAS---------------MH----GFFNP---QRP--HNHPHGHQ-T----HS---HHQHFG-GNFG-PEPGS-SCLHSGRIM----GY--NSGM---GL---Q----------QGFTE--GFD-PLSEG-Q-------------------SADGFSQQQS-QQ---------QQ---------------------RPGSMPDF--------QHHGPPSGNHPVPAPCLPLD----QSPNRAASFHGL-SSSS---------------------SSSESHNLE----PRR-MPPP-G--GVDGLD------------YNYSN-EPSSG---HFEVSVYS-PSE-S--DSQLS-HFGPGRQV--PGP-NFPG-NPGL--SRAPGM--QGISKEH--P------------------------HAPPQQQQPSAQH--NVFFERF--GGGRKIPVGIEP----GS-RHPLL------------QQ-------------------------------QPGLIGRQNTCPPSLPQ--PPQSETGSANAGM-QEGG-VMMP-GQHNQ--------FEYPIHRPENRRM--------------HSY--GDP---MFNM---QQQP-----------PPP--QQPS------NQRLQHFDS-PYLNMAKRPRFDFPNATH----G-GESCGSWNS-----G-MHNPPGMENNL------------SPA-A-YP-------------GLPGEFTP--PVTDG---F-STGPSLQLTGP----EQQSM-------Q-----------------------QQQN--------AASMIKQMASRS------------QQQR-MR-QPN---LQQLGHHSDVP----QG-PLGHGGPVGGMPQ-S-SFERE---------------------NGGRMV------------------NFDGRNP-HITLES--GWFPG----PH---PPGEML--------GHRM-GPG--GEVGAHE---M------QQ--NGPGMMFRA--G--VNGM-GMQE-P----MR-IP--GEGHVQPLLSP-----NIHSQFNNG----MGNLSQMQSPSTG-----VGLPNTPSER-R----PNDFPGP---PMGGPSSFPY-GGSNRQ-GASLCNSQGVSTSP-------------------GSF--TSQSDF--PTSQR----SSVSKLGGLSLG--------NFSK----------TSG------------KDN-VFGQSCLAALSTACQNMIASLGAP--------NLNVTFNKKT---Q-GEGKRKLSQTEQDLN--N-SAV-NG----------------TGNAGTEYFP-SIAAPQNGQIPP----AGNSNTKPPGQ---------NHTVQGE-AST--LSPNYN---M---------DT-TPCSEGK--AAT--GSGRGR--GRRKRD----------SGHVSP--------GIFFPSE--------------NSNPVVS-P-GQQVASAAGVGER----------------------------CTGTPQ---E-KPHTSP--SW----GKG-----GDLL------------LGDQAD-LMS--SLD--SGIQSV-SKS---EVCSP-----RMDFT-DD-VGT--HY-SNEDE------VSSSSDAPS---------------------------TVKAGRSPLL-GSPKLQR-------DNGLIG--GQKGQGMGLSNH------------------TTSTS----DGFG-------GV---GHP-GTPGME-QARTP---SSTSG------------QDEIHPLEILQAQIQLQRQQFSISEDQPLAVK------------NNKKSSDCSG------QNGD--GELSSCS---P---DAGKGS--------VGTIDLDTLMAEQHA-TWYVPS-------DK-SLLED----SEEEKS----VWEKNKVQGT-IKEEVDLSQSKTT------GGAGSSGTTGGMGSHLQCLSVHCTDELGESKGRGGPVPSWRSLHSDISNRFGTFVAALT-----------------------------------

>C.idella.mn1a

--MNSNYN-SAG-FH----------MK-----G-P--S-VAVEP-MMGPLNESP--M-QG--------LNFVS-N--RDQY-GFQ-TH-GHGDML-------------AMGV--QPQHQ-----------------------------IHM--QGPFNH---QPPN-HEQH---------------SHLYQD---------SVPSCLHGDRHM----GF-NSTNA---GH---P----------HMFEG--GFGQQLAEA-Q-------------------SRECISQQQHQ-----------Q---------------------RMAAMPEF--------QPHGHPNGNNAVPAPCLPLD----QSPNRAASFHGLP---S---------------------SSPETHRLE----HYR-LFPQ-G--RMGGSE------------HCFPC-DPLTG---NFDMTGFSTADN-S--EHKLP-YCETGNQV--AGG-HFPTCNRSG--SRGPMM---GGSKVD--Q----------------------------------QLPQQ--NVFSDRF--GNRGK----MDPG--VNA-RHHLM---A--------QQ-------------------------------RPGPVARQNPGSPALPR-FYHTPDYVANNAD-------PMVH-VQHGH--------

LDRPVHRLNNHNM--------------HPF--GEP---VFDV---PQLA----------------PQQP-----HHPHLSSL---
PYLNMAKRPRFDLPNG------SAGESCSPLSN-----S-LHNRPNLENHL------------SPS-A-FP-------------SPMGDFTS--
HVTDG---F-PSGPLPLSSGP-QQQQQQQQ-------Q----------------------RRQN--------AAMMIKQMASRS------------
QQQR-MR-QPD---LQQLSLHGDVT----SNG-MVCRGPLGSVSQ-S-NFEKK---------------------HNFHG-------------------
-NFD--SP-HLPQEN--SWFPE----PQ------------------------QHCRETNTHA---LEQ----AE--NGHNIIFRQ----------GVTS-----
----------MDMQSLNSP-----GAHHPFENN----VSNPLQMQSPDES-----NMQSGAPTDR-R----PAEFGGM---
AMRRQHSFPP-GGPSQQ-GAPQSNPPGFSSSP------------------GNY--PAHPEY--LSSQH----LSVNKLGALSLG--------
NLNK----------AST------------KDS-VFGQSCLAALSTACQNMIASLGAP--------NLNVTFNKKS---Q-
NEAKRKAGQVEQDINS---SGSS--------------------GPGAEYFQ--SNASQNSQTPC----SGNNNNTTTGQ------
SGTGQMAKRE-AST--LSPNDN--M---------ES---GCEGK--MAT----GKGR------------
--GKKRRD----------SGHISP--------GNFSPPC--------------GGGNPVVSP-
SQQGSALGMGMES---------------------------RGKTPE-----RSLVSP--SF----G-------------------------KPD-LAT--SMD--
SGIQSV-GKS---DGVSP-----CMDYL-DE-ASP--NY-NAEDP--
RPCRAGVKCNSENRA--GYS-DAPCME-QVRTP---LSNTG---
---------QDEVHPLEILQAQIQLQRQQFSISEDQPMGGK-------------TGKKADCQA-----GLNGE--CALASSS---P---
VTGKGS--------VNTIDLDSLMTEQHA-TWYVPG-------NK-ALIED----PGNDKC--LGFWDRARGQSD-NKEGHG-----
----

>E.atami

-------MSSC-EAWALG---VGAQRAWGPA----------------------PRAPQYCAEP-----
SGPPP----------------------------------CPCETH---CYYPG----
TTGYPRYPGGV-------------------GDPYVPPGAATAVLPTEPET------------------------------QGYGTLYGRGAHSVPT---
--PAPFPYALSEPCRV------PFPGYEAAAPGSL-------
------------------------LG--TGDAG---GFEAT-----------DSHAV-RYLAGKLR------
GQPGTANRGAPGREPGWPGEGANYSH---GLGAQPF--------------------PGPYS-------
-------------------------------------PGAALIQQSGVPSTDAM------------------------YGTHGQ--------LGF------------------------
------DP------------------------------AREP--------AQQHRGSGEFPASAKRPRADF------------------------------LAFPGHK-----
-------FPTDA-AP------------NGLPGS----------------GGRTWGDALA--SRRG--------
KAAPCELPEAQE---SLHMFASGMPGGFYR---SQDAM-------------------------------------
----------ALSNRAKQQQQQQQ---------QQQH----------------NQSSFSHGLPSEGYSSR---------------------------------
GLTE-------------APPESYCMPGP-----GFQQQQQQQ----------QQHQQAE-----FGLRSSPLLL--------------------
SGCFPHSPGPLDLAGVPRGR--SSTGKLCRLSLG--------PLAV---------
HSGAGHGGSGSSEVKENTGASYGQSCLAALSTACQNMIASLGAP--------SLNVTYGKRP---P-------PGESEGRGR--
-------------------------VPGGESAPARDETPTLSTSPG-------ARVSETGT-----TDCAGPPGAVAEICG--LSSPGD-----------
---PSSGRGESRAT---RGRGR--
---GRRKGG----------VRTGSP--------GVKLTPA-----------------PGDDIL---GGSGET---
GGEGLGSP--GW----GGA-------------PSETAGAGDK-PSDLLLA--SLD--SGIQSL-SPP---SDASP-----LLEFC-AE---------
--GTVSGGNPELGV---------GVMQ---
GP---GVGAGTPPTSAALMPP---DHRTVGPA------------------CSQ----------------------
-----------------MTSMDPTHVQIVGDGIPRSLPR-----------------------------PAESAVGFGM-LDGEGKAPVWYAD---------
-----RSGAGYSWHGPALHCG---------------WQSM--

>E.burgeri

-------MSSC-EAWALG---VGVQRAWGPA----------------------PRAPQYCAEP-----
SGPPP----------------------------------CPCETH---CYYPG----
TTGYPRYPGGV-------------------GDPYVPPGAATAVLPTEPET------------------------------QGYGTLYGRGAHSVPT---
--PAPFPYALSEPCRV------PFPGYEAAAPGSL-------
------------------------LG--TGDAG---GFEA---------T--DSHAV-RYLAGKLR------
GQPGTANRGAPGREPGWPGEGANYSH---GLGAQPF--------------------PGPYS-------
-------------------------------------PGAALIQQSGVPSTDAM------------------------YGTHGQ--------LGF------------------------
------DP------------------------------AREP--------AQQHRGSGEFPASAKRPRADF------------------------------LAFPGHK-----
-------FPTDA-AP------------NGLPGS----------------GGRTWGDTLS--SRRG--------
KAAPCELPEAQE---SLHMFASGMPGGFYRSQDAMALS-----------------------------------
-------------NRAKQQQQQQQQQQ-----------QH-----------------NQSSFSHGLPSEGYSSR---------------------------------
GLTE-------------APPESYCMPGP-----GFQQQQQQ--QQQQQQQQHQHQQAE-----FGLRSSPLLL--------------------
SGCFPHSPGPLDLAGAPRGR--SSTGKLCRLSLG--------PLAV---------

HSGAGHGGSGSSEVKENTGASYGQSCLAALSTACQNMIASLGAP--------SLNVTYGKRP---P-------PGESEGRGR--
-------------------------VPGGESAPARDETPTLSTSPG-------ARVSETGT-----TDCAGPPGAVAEICG--LSSPGD-----------
---PSSGRGESRAT---RGRGR--
---GRRKGG----------VRTGSP--------GVKLTPA----------------PGDDIL--GGSGET---
GGEGLGSP--GW----GGA-------------PSETAGAGDK-PSDLLLA--SLD--SGIQSL-SPP---SDASP-----LLEFC-AE---------
---GTVSGGNPELGV---------GVMQ---
GP---GVGAGTPPTSAALMPP--DHRTVGPA-------------------CSQ----------------------
-----------------MTSMDPTHVQIVGDGIPRSLPR----------------------------PAESAVGFGM-LDGEGKAPVWYAD---------
-----RSGAGYSWHGPALHCG---------------WQSM--

>X.laevis.L

-------MFGL-EQFEPQ--------INSRS--------AGQGER------NFPAQPGM----NMSSHFK-SAASFH----------SA-----G-A--P-
GSVDS-ALGALNENS--M-LG--------MNLNL-N--GEPY-GFH-SR-GQADMH------------PGGM------Q-----------PQP---
------------VH----GFFNN--Q--PH-HGHPSTHPHP----STHQ-HHPHFSGGSFNGADAAA-SCLHGARFM----GY--NNNI--
-AN---Q----------QAFAE--GYE-QMAEN-Q-------------------AGEGFV----------------QQ--------------------RSANMSEF-----
---QHPNSSATNNAVPAPCLPLD----QSPNRAASFHGLP--TS----------------------STSDSHNQE----QRR-IHNQ-G--
GVEPLE------------YNYPG-DGPSG---HFEVPVFS-PSD-S--DG----HYGAGRQV--PSS-SFPG-TSVL--PRPPGL--
VGMGKVH--P------------------------------QQ---QQ--GVFFERF--NNARKISVGMDPA--VNG-RHPLL--QQ--------QQ-----
--------------------------QTGLLARQNSCPPAIPR--QQQTEANTSNPNL-QDNG-PVMQ-SQHAQ--------
FEYPIHRLENRNM--------------NPY--SES---VFSM---QQTP-----------P----QQPP------NQRLQHFDA-
PYMSVTKRPRFDFPNN-H----T-VDNCAAWNN-----S-NIHNAAIESHL------------SPS-T-YP------------GLPGEYNP--
QVPES---F-PPGPALQHPGS----DHQSL-------Q------------------------QRQN--------AAMMIKQMASRN------------QQQR-
MR-QAN---IQQLGHHGDVN----QS-SIVHGGHVGGMQQ-P-NFDRE----------------------GGRIG-------------------
TFDPQNP-HVAQEN--AWFPG----TH---PPGDIL--------QRRMGGSNLPADPSSHD---MNL----QQ--NGSNLLYRP--G--
VNRM-GIQE-P----LG-IP--GEGHVQALHSP-----GMHSQFGSN----MANLSQMQSPGGG-----VGLSSAPADR-R---
GPADFTAS---SIGGQTGFPF-VGANRQ-STPH-NPPGVNSSP-------------------SSY--PPQSDF--QASQR----
STASKLGALSLG--------SFSK----------SNT-----------KES-MFGQSCLAALSTACQNMIASLGAP--------
NLNVTFNKKS---Q-TEGKRKLSQTETDIN--S-NSG-N-------------------NTSSDYFP--GASSQNNQVSA----
SSNANNKSTGQ------VGASQPTQGE--TS--LSPNYN--I---------EV-TPGNDGK--
PVT--AGGRGR--GRRKRD----------SGHVSP--------
GNYFEKY------------SADSGGAVVSP-GQQGPTANSGE------------------------------SGGVPH---D-KPLTSP--SW----
GKG-----GELL------------LGDQPD-LMS--SLD--SGIQSV-TKS---DSSSP-----DVDFG-ED-VNT--TY-GNEDE------
VSSSSDNN---------------------------IAKPNSCPIVTGSPKIQR------GDHGLLN--GQKSMGINLLNN-----------------
TTSLP----DSYGL---SSTGG---GHP-GTPGME-QVRTP---TSTST------------
QDEIHPLEILQAQIQLQRQQFSISEDQPLGMK--------------NKKNDCTA------QNVD--SELNSCC---S---DNVKNS------
--MSTIDLDSLMAEHNS-TWYMPN-------EK-SLMEG----DEEDKS--ITPWEKSKSQLT-NKEALDLPQNKTS-----------
--AAAQNGSHLQCLSVHCTDDIGEPKGRT-PVPTWRSLHSDISNRFGTFVAALT----------------------------------

>X.laevis.S

-------MFGL-EQFEPQ--------INSRS--------AGQGER------NFPAQPGM----NMSSHFK-NAA-FH----------SA-----G-A--P-
GSVDS-AMGALNEPS--M-LG--------MNLSL-N--GETY-GYH-AR-GQSDMH-------------PAGM------Q------------PQP---
------------VH----GFFNN--QPHPH-HGHPSTHP--------HQ-HHPHFS-GNFNGPDSAA-SCLHGGRFM----GY--NNNI---
GN---Q----------QAFGE--GYE-QMAEN-Q-------------------PGEGFV----------------QQ--------------------RSGNMSEF------
--QHPNSSATNQAVPAPCLPLD----QSPNRAASFHGLP--TS--------------------SNSDSHNHE----QRR-IHNQ-G--
GVEPLE------------YNYPS-DGPSG---HYEVPVFS-PSD-S--DG----HYGAGRQV--PGS-SFPG-TSVL--PRPPGL--
VGMGKVH--P-----------------------------QQ---QQQHGVFFERF--NNARKMSVGMDPT--VNA-RHPLL--QQ--------QQ-
------------------------------N-GLLARQNSCPPAITR--QQQTEANTSNPNL-QDNG-PVMQ-SQHTQ--------
FEYPIHRLENRNM--------------NPY--SES---VFSM---QQAP-----------P----QQPQ------NQRLQHFDA-
PYMSVTKRPRFDFPNN-H----NGVDNCAAWNN-----S-NIHNAAIESHL------------SP--T-YP------------GLPGEYNP--
QVPES---F-PPGPSLQHPGS----DHQSL-------Q-----------------------QRQN--------AAMMIKQMASRN-----------QQQR-
IR-QAN---IQQLGHHGDVN----QS-NIVHGGQVGGMQQ-P-NFDRE----------------------GSRIG-------------------
TFDPQNP-HVTQEN--AWFPG----SH---PPGDIL--------QRRMGGSNLPADPSSHD---MNL----QQ--NGSNLLFRP--G--
VNRM-GIQE-P----LG-IP--GEGHVQTLHSP-----GMHSQFGSN----MANLSQMQSPGGG-----VGLSSAPADR-R---
GPADFTAS---SIAGQTGFPF-VGSNRQ-STPH-NPPGVNSSP-------------------SSY--PPQSDF--QASQR----
STASKLGALSLG--------SFSK----------PST------------KES-MFGQSCLAALSTACQNMIASLGAP--------
NLNVTFNKKS---Q-TEGKRKMSQTETDIN--S-NSG-N------------------NTSSDYFP--GASTQNNQVSG----

SSNANNKSTGQ------VGASQTTQGE--TS--LSPNYN---------------------------------------I---------EV-TPGNDGK--
PVT--AGGRGR--GRRKRD----------SGHVSP--------
GNYFEKY------------SADSGGAVVSP-GQQGPSANSGE-----------------------------SGGAPH---D-KPLTSP--SW----
GKG-----GELL------------LGDQPD-LMS--SLD--SGIQSV-TKS---VSSSP-----DVDFG-ED-VNT--TY-GNEDE------
VSSSSDNN----------------------------IAKPNSCPMGTGSPKIQR------GEHGLLN--GQKSMGLNLLNN-----------------
TTSLP----DSYGL---SSNGG---GHP-GTPGME-QVRTP---TSTST------------
QDEIHPLEILQAQIQLQRQQFSISEDQPMGMK--------------NKKNDCTA------QNVD--NESNSCC---S---DNVKNS-----
---MSTIDLDSLMAEHNS-TWYMPN-------EK-SLMEG----DEEDKS--ITPWEKSKSQQT-NKEALDLPQNKTS----------
---AAAQNGSHLQCLSVHCTDDIGESKGRT-PVPSWRSLHSDISNRFGTFVAALT----------------------------------

>P.annectens

-------MFGL-EQFEPP--------ASNRS--------GGQAER------NF-SQPGLT---TMSSLYK-NAP-YH----------SS-----G-P--P-
GSVES-GMNPLSESQ--MPLG--------MNMSL-N--GEPY-GFH-AR-GHAELH-------------AGSM------Q------------QQT---
------------VH----GFFNN---QQPH-PGHPHAHH-A----HPHQ-HHPHYS-GSFAGPDPSN-SCLHGGRLM----NY--NSTL--
-GG---Q----------QAFSE--GYD-NVGEN-Q-------------------GGEGFG----------------QQ--------------------RPNNIPDF-------
-QHHNPSSS-HAVPAPCLPLD----QSPNRAASYHGLT--ST---------------------TTSEPHGIE----PRR-LQNP-G--
GVDSVE------------YTYPS-DAPSG---HFDMPVFS-PTD-S--EAQLP-HYSAGRQV--PGG-NFPN-NSSL--PRAPGV--
VGMSKVH--P------------------------------PQ---QH--GVFFERY--GSTRKMPVGMEPG--MNN-RHPLM--QQ--------QQ---
----------------------------QAGLLARQTSCPSAMPR--QQQTENGASNSSL-QDNG-QMIP-GQHAQ--------
FEYPIQRLENRSM--------------HQY--GDA---VFNM---QQ-------------S----HQPS------SQRLQHFDA-
SYMNVTKRPRFDFPNN-H----N-VDSCATWNN-------GMHGTGMENHL------------SPS-A-YS-------------GLPGEFTP--
PVPDG---F-PPGQPLQHPGP----DQQNL-------Q------------------------QRQN--------AAMMIKQMASRN------------
QQQR-MR-QPS---LQQLGHHGEVG----QN-NMVHGGPVGTLTQ-P-SFERE----------------------GGGRIS------------------
-SYDPQNS-HMPQDN--AWFSG----PH---PPGDML--------QRRIGGSTLPADANPHE---INL----QQ--TASNMMFRP--G-
-VNGM-SMQD-S----IG-MP--GEGHVQALHSP-----GMHAQFGNN----MGSLSQMQSPGGG-----VGIPSASGDR-R---
PPPDFQVP---PMG-QPGYPF-GPPNRQ-TTSHNNPSGVNGSP-------------------SSF--SQQSEF--QSSQR----
STASKLVDLSLG--------SFNK----------PNS------------KDS-MFGQSCLAALSTACQNMIASLGAP--------
NLNVTFNKKN---Q-NEGKRKLSQTEQDLN--N-NSGSNV----------------NNGTGSDFFP--SGASQSNQMLS----
AGNSSGKPAGQ------NGVTQPAHGE--SN--LSPNYN---------------------------------------I---------EV-TAGNDGK--
PLA--GSGRGR--GRRKRD----------SGHVSP--------GSFFDKY-
-----------SADNGATVVSP-GPQCQAANVGE-----------------------------QGGTPH---D-KSLTSP--SW----AKG--S---ELL-
L----------SD-QQD-LMS--SLD--SGIQSV-TKS---DSSSP-----HVDFP-DD-IST--NY-GNEDE------VSSSSDNN-------------
---------------LSKPNRSPLVTSSPKIQR------GDHGLLS--GQKSMGLAMHNN------------------ATSTP----DSYGL---
TSTGS---GHP-STPGME-QVRTP---TSTST------------QDEIHPLEILQAQIQLQRQQFSISEDQPVGMK--------------
NKKAECPG------QNGE--GELASCG---T---DSGKNA--------MSTIDLDTLIAEHNS-TWYMPN-------DK-TLIEG----
QDEDKS--MAPWEKTKPPSS-SKEAHDLSQNKTS-----------TAAAAQNGSHLQCLSVHCTDDIGESKGRT-
PMPTWRSLHSDISNRFGTFVAALT----------------------------------

>A.mississippiensis

---MPRSMFGL-EQFEPQ--------PPGRS---------------------LERPFGAALQPLGAHFKAAPG-FP-----------------------------
ADAALDPPL----LG--------MN----MSLAPDY-GFHAPR-GAGLEH-------------PPPPPQQPQPQ------------PQPQPQ-------
-----PQPQQHGFFGA---QQPPGAAHPHPHPPF---------GGGGGGGTGGFGPDPGA-SCLHGGRLL----
GYGGQGQGQGQPQ--------------AAFAE--GYEQHLADPQA------------------AAADGF--
APPPRADF-QQPPPPPPPHHGPVSGHAVPAPCLPLD----QSPNRAASFHGAA---------------------------DSHG------
LERRLPAP-----PDGLD------------YSYP--DGAAA---HFDLPGFS-PSE-P--DAQAP-LPAYGRP-------GFPV-------
PRGPAL---GLAKPPQHP----------------------------PPQQQPAPH--GVFYERF---GGRKLPAAMEPGGRHGLMQPP-------
--QPGLLARQSSCPPALPR--
QPAAEASVPDGAGAGQGPHAQHQHAQHQHQHQHQHAAFEYPIHRLENRAV---------------PY--GDA---VFGV--
GQPPP----------------PPPA-----PSQRLQHFDA-PYLSAPKRPRFDFPER-----------CAAWGG---------GGLALDTHL-------
-----SPS-A-YA-------------GLPGDFTP--PGPDGFAPA-LQAPDLQQQQQQQQQQQQHQ-------------------------------
QRHNAAAAAAAAAALMIKQMASRS------------QHQPPQRLRPPG--LQALGHPGDV-----------HGGGGGGG-----
GFERA---------------------DGGGARA-------------------AFEAAHV-----DG--AWFPGGELLAP-----------------PRR------------
------------PA--EAHDLLFRP----------GLEA--------------------LPSP-----GLHAPFAP-----------MPSPGAE--------
RRAPSDFAPPPPQPPQQPPPQQQPQQQPPGFPF-GPPGRQ-PTPHGASPDFAPAP---------------------------------------
RPAASKLGALSLG--------SFGK----------AGG------------KDSAVFGQSCLAALSTACQNMIASLGAP--------
NLNVTFGKKS---P-PDGKRKLGAPEPDGAAP-------------------------GPGPDYYP--------------------AAKPAGA-------

GQALPGESS------LSPGYA---LEAAPGPDGKAAAAA--AGGRGR-------------
--GRRKRD----------SGHVSP--------GSFFDKL---------
AADGGGGAAGVS-P-GQP--PPPPQPPQQQQQQQAPSGER----------------GGGTPH---D-KPLTSP--SW----GKG--G---
ELL-L----------GE-QPE-LLA--SLD---GAAKP-------DGASP-----PVDFA-ED-VSS--SY-AAEDE------VSSSSDK-----------
---------------------PARSPLLGGSPKLPP---PPRPEHALLN--GQKPLALGLLST------------------STSTP----DSYGL------SS-
--THP-ATPGLE-QVRTP---SSTSA------------QDEIHPLEILQAQIQLQRQQFSISEDQPLGLK--------------SKKAECAG--
----QNGD--GDLGACC---P---DGGKGA--------MSTIDLDSLMAEHNS-TWYMPG-------DK-ALLDG----PDEDKP--
VAPWEKAKPQNP-SKEAHDLPQNKTS-------------ATAQTGSHLQCLSVHCTDDMGDAKGRT-
AVPTWRSLHSDISNRFGTFVAALT----------------------------------

>P.spathula.Chr22

-------MFGL-EQFEHQ--------ISSRN--------VGHGHR------NF-NQPGL----NMSAHYK-NPT-FH----------PS-----G-P--P-
GAVEQ-GMGTLNEPS--M-LG--------LNMNL-N--GEQY-GFH-PR-GHSEIH-------------AGGL------Q------------QQP----
-----------MH----GFFNN---QQPH-HGHPHGHH-P----HAHQ-HHPHFG-GNFSGPDPSA-SCLHGGRLM----GY-NSSSM-
--GP---Q----------QGFAE--GFD-PMAEN-Q-----------------GAGGEGFG----------------HQ-------------------RPSNMPEF---
-----QHHNSQATNHAVPAPCLPLD----QSPNRAASFHGVP--ST---------------------TSSDAHSLE----QRR-MPAQ-A--
GVETLE------------YSYPS-ETPSG---HFDMPVFS-PSE-S--DAQLP-HYGAGRQV--PGG-NFPG-NPAM--PRAPGM--
PGISKAH--P------------------------------QQ---QH--GVFFERF--GNARKMSVGMEPG--INA-RHPLM---Q--------QQ-------
------------------------QGGLLARQNSCPPAIPR--QPPTEASAPNPNL-QDNG-VMMP-GQHNQ--------
FEYPIHRLENRSM--------------HPY--SDP---MFNM---QQQP-----------P----QHPP------NQRLQHFDT-
PYLNVAKRPRFDFPNN-H----N-PENCATWNS-----N-IHNPPGMENHL------------SPS-A-YP-------------GLPGEFTP--
PVPEG---F-PPGPALQHTGP----DQQSL-------Q------------------------QRQN--------AAMMIKQMASRN------------QQQR-
MR-QPS---LQQLGHHGDVN----QN-SMVHGGQVGNMPQ-P-NFERE----------------------SGGRMA-------------------
NFDSQNP-HMAQEN--TWFPG----PH---PPGEMI--------QRRMGTSNVPGEASPHE---MNM----QQ--NGANMLFRP--
G--VNGM-GMQE-P----MR-MP--GDGHVQALHSP-----GM--QFGNT----MGNLSQIQSPGAG-----VGLPNAPSDR-R---
-PPDFPTP---PMGAQPGFPF-SGTNRP-GASHNNPPGVNASP-------------------GNY--PSQSEF--QQGQR----
SSVSKLGALSLG--------SFSK----------TNA------------KDN-MFGQSCLAALSTACQNMIASLGAP--------
NLNVTFNKKS---Q-NEGKRKLSQTEQDIN--STNAGATG----------------NSSSGPEYFQ--SSASQNSQMPG----
AGNSNSKLSGQ------SGTSQPTQGE--AN--LSPNYN---M---------DA-TPVNEGK--
SAI--GSGRGR---GRRKRD----------SGHVSP--------GIFFDKF---
---------SSDCGNSVVSP-GQQGPPASAVEL----------------------------GGGTPH---D-KPLTSP--SW----GKG-----SDLL----
--------LSDQPD-LMS--SLD--SGIQSI-TKS---DNSSP-----HVDFP-DD-VST--NY-GNEDE------VSSSSDNN----------------
------------IPKPNRSPLVTSSPKIQR------GDHGLLN--GQKQMGLGMLNN------------------TTSTP----DSYGL---
SSTGG---GHP-GTPGME-QVRTP---SSTST------------QDEIHPLEILQAQIQLQRQQFSITEDQPLAMK--------------
NKKPDCPS------QNGD--NELINCV---T---DGGKNA--------MSTIDLDSLMAEQHA-TWYIPS-------DK-SLMEG----
QEDDKS--MAPWEKTKPQNN-NKEAQDLPQNKPS-------------AAGQNGSHVQCLTVHCNEDMGEAKGRT-
PVPPWRSLHSDISNRFGTFVAALT----------------------------------

>P.spathula.Chr25

-------MFGL-EQFEHQ--------INSRN--------VGHGER------NF-NQPGL----NMSAHYK-NPT-FH----------PS-----G-P--P-
GAVEQ-GMGTLNEPS--M-LG--------LNMTL-N--GEQY-GFH-PR-GHSEIH-------------AGGL------Q------------QQP-----
----------MH----GFFNN---QQPH-HGHPHGHH-P----HAHQ-HHPHFG-GNFSGPDPSA-SCLHGGRLM----GY-NNSSM-
--GP---Q----------QGFAE--GFD-PMAEN-Q-----------------GAGGEGFG----------------HQ-------------------RPSNMPEF---
-----QHHNSQATNHAVPAPCLPLD----QSPNRAASFHGVP--ST--------------------TSSDAHSLE----QRR-MPAQ-A--
GVEPLE------------YSYPS-ETPSG---HFDMPVFS-PSE-S--DAQLP-HYGAGRQV--PGG-NFPG-NPAM--PRAPGM--
PGISKAH--P------------------------------QQ---QH--GVFFERF--GNTRKMSVGMEPG--INA-RHPLM---Q--------QQ--------
-----------------------QGGLLARQNSCPPTIPR--QPPTEASAPNPNL-QDNG-VMMP-GQHNQ--------FEYPIHRLENRSM-
-------------HPY--SDP---MFNM---QQQP-----------P----QHPP------NQRLQHFDT-PYLNVAKRPRFDFPNN-H----N-
PESCATWNS-----N-MHNPPGMENHL------------SPS-A-YP-------------GLPGEFTP--PVPEG---F-PPGPPLQHAGP----
DQQSL-------Q------------------------QRQN--------AAMMIKQMASRN------------QQQR-MR-QPS---LQQLGHHGDVS---
-QN-SMVHGGQVGNMPQ-P-NFERE----------------------SGGRMA-------------------NFDSQNP-HMAQEN--TWFPG----
PH---PPGEML--------QRRMGTSNVPGEASPHE---INM----QQ--NGSNMLFRP--G--VNGM-GMQE-P----MR-MP--
GDGHVQALHSP-----GM--QFGNN----MGNLSQMQSPGAG-----VGHPNAPSDR-R----PPDFPTP---PMGAQSGFPF-
SGANRP-GAPHNNPPGVNASP-------------------GNY--PPQSEF--QQGQR----PSVSKLGALSLG--------SFSK----------
TNA------------KDN-MFGQSCLAALSTACQNMIASLGAP--------NLNVTFNKKS---Q-NEGKRKLSQTEQDIN--
STNAGATG----------------NSSSGSEYFQ--SGASQNSQMAG----AGNSNSKPSGQ------SGTSQPTQGE--AN--

LSPNYN---M---------DA-TPANEGK--SVT--GSGRGR--------------------------------------
------------------------------GRRKRD----------SGHVSP--------GIFFDKF------------SSDCGNPVVSP-GQQGPPTSAVER---
-------------------------GGGTPH---D-KPLTSP--SW----GKG-----SDLL------------LSDQPD-LMS--SLD--SGIQSV-TKS---
DSSSP-----HVDFP-DD-VST--NY-GNEDE------VSSSSDNN---------------------------ISKPNRSPLVTSSPKIQR------
GDHGLLN--GQKQMSLGMLNN------------------TTSTP----DSYGL---SSTGG---GHP-GTPGME-QVRTP---SSTST------
------QDEIHPLEILQAQIQLQRQQFSISEDQPLAMK-------------NKKPDCPS------QNRD--NELVNCV---T---
DSGKNA--------MSTIDLDTLMAEQHA-TWYIPN-------DK-SLIEG----QEDDKS--MAPWEKTKPQNN-
NKEAQDLPQSKPS-------------AAGQNGSHVQCLTVHCNEDMGESKGRT-PVPSWRSLHSDISNRFGTFVAALT------
----

>C.dromedarius

-------MFGL-DQFEPQ--------INSRN--------AGQGER------NF-NEAGLS----MNAHFK-APA-FH----------AG-----G-P--P-
GPVDP-AMSGLGEPP--I-LG--------VNM-------EPYG-FH-AR-GHSELH------------AGGL------Q-----------AQP---------
-----VH----GFFGG---QQPH-HGHPGGHH-P------HQ-HHPHFG-GNFGGPDPGA-SCLHGGRLL----GY--GGSA---
SGLGSQ----------PPFAE--GYD-HMAES-Q-------------------GPESFG------P---------Q---------------------RPGNLPDF-------
---HSSSASGHAVPAPCLPLD----QSPNRAASFHGLP--AS---------------------GGSDSHSLE----PRR-VANQ-G--
AVDSLE------------YNYPA-EAPSG---HFDM--FS-PSD-S--EGQLP-HYAAGRQV--P-GGTFPG-ASAM--PRAAGM--
VGLSKMH--A--QQQQQQQQQQQQQQQQQQQQQQQQQQQQQQQQQQQQH--SVFFDRF--GGTRKMPVGLEPG--
VGS-RHPLM--QP--------PQQAPPPPQQQPP------Q-QPQQPPPPPPPPPPPGLLVRQNSCPPALPR--
PQQGEAGTPSGGL-QDGG-PMLP-SQHAQ--------FEYPIHRLENRSM--------------HPY--SEP---VFNM----QHP---P-----
--P----QQAP------NQRLQHFDAPPYMNVAKRPRFDFPGS-A----G-VDRCASWNG-----S-MH-NGALDNHL------------
SPS-A-YS-------------GLPGEFTP--PVPDS---F-PSGPPLQHPAP----DHQSL-----QQQQQ------------QQQQ------QRQN---
-----AALMIKQMASRN------------QQQR-LR-QPN---LAQIGHTGDVG----QG-GLVHSGPVGGLAQ-P-NFERE-----------
-----------SGGAGA---------------GRLGTFEQQAP-HLTQES--AWFPGPH---P---PPGDLL--------
PRRMGGSGLPADCGPHD---PGLAPPPPP--GGSGVLFRG--S--------LQE-P----LR-MP--GEGHVPALPSP-----GL--
QFGGS----LASLGQLQSPGAG-----VGLPSAPSER-R--PPPPDFTAP---ALGGQPGFPF-GAANRQ-ATPHSG-
PGVNSPPSTGGGGGS-TGGGGGSGG--AY--PPQPDF--QPSQR----TSASKLGALSLG--------SFNK----------PSS---------
---KDN-LFGQSCLAALSTACQNMIASLGAP--------NLNVTFNKKN---P-PEGKRKLSQNENDGA------------------------
AVASNPGSDYFP--GGTAPGAPGPGGPSGTSSSGSKPSGP--------PNPPAQGD-GTS--LSPNYT----------------------------
---------------L---------ES-TSGNDGK---PVPGGGGRGR--
GRRKRD----------SGHVSP--------GTFFDKY--------SAAPDSGGAPGVS-P-GQQQAPGAAVVG-----GSSTGEAR--------
--------GAPTPH---E-KALTSP--SW----GKG-----AELL------------LGDQPD-LMG--SLD--GG-----AKS---DGSSP----
HVGEFASDE-VST--SY-ANEDE------VSSSSDNPPA-------------------------LAKASRSPLVTGSPKLPP-
RGVGAGEHGPKA--PPPPLGLGILST------------------STSTP----DSYG-------GG-------GTPGLE-QVRTPTSSSGAPP-----
-------PDEIHPLEILQAQIQLQRQQFSISEDQPLGLK-------------GGKKGECAVGASGGVQNGD--SELGSCC---S---
EAVKSA--------MSTIDLDSLMAEHSA-TWYMPA-------DK-ALVDG----PEDDKT--
LAPWEKAKPQNPNSKEALDLPANKAS--------------ATQPGSHLQCLSVHCTDDVGDAKARA-
SVPTWRSLHSDISNRFGTFVAALT-----------------------------------

>A.prasina

-------MFAL-DPFDPH-----GGGGSGRS--------GVPGER---GGGGF-GPG-------AGSQFK-PSP-FH----------GGS----S-S---
-GGGEPASVNALAEPSSTL-LA--------MNL---TLSGEGYGGFHGPRGGPPELG----------------------Q-----------AQP----
-----------IH----GFFGS---PPPG-PAAHQQSQ-P---------PVSHFG-GGF-GTEPSA-SCLHGGRLL----GY--SGG---------Q---
-------QAFAADGGYEQHLADG-Q------------------AGGDGFG------Q---------Q---------------------
RAGPLQDFQPPPPPPHHLHNPG-----VPAPCLPLD----QSPNRAASFHGLP--GA---------------------ASSEPHGLDS---
QRR-LPSQ-GAAAVDALE------------YSYAN---SES---HFDLPVFS-SSE-A--EGQLP-
QYGSGRQAAPPGGGNFPGHSAAL--PRVPGS----LGKVP--S---------------------------------QQSPH--GVFFDRF--
GGARKMSLGLEPG--LSAGRHHLV---Q--------Q----QPP------------------------PSALLARQNSCPPALPR-
PQQCPEGSAANANL-QDGGAPI---SQHGQ--------FEYPIHRLENRGLQHHPHHSQQQHQQHPYVPGEP---LFNV---
PHQHQQHQ-------H----QQPP------NQRLQHFDAPTYVNLPKRPRFD-----------------SWSGGG-GAG-
MHGAAALESHL------------SPSAA-YP-------------GLPGDFTP--PVPEH---F------PPPPGS----DPQAA-----L------Q--------
-----------QRQN--------AALMIKQMAATR-----G------QAQR-LR-PPG---LQQVSHHQHPH----GAGGHGHHQSPHQ-----
----------------------------HPGEAA---------------------------FEAQEG--AWFPP----PH---PP--
A--GSGDLLFRP--G--MG---GLQEPPPPPALR-MSSGAEGHVP---SP----GGLQGQFG--------------------------LSPPSER-
R--PSHPDFASQ-------AQSFPF-GGPSRQ-ATPHSASPGSFGPP-------------------TDFQSSAPPPS--Q-PRP---
SPASSKLGALSLG--------SFPKG-----GVGAPPG-------SAGPKESSGLFGQSCLAALSTACQNMIASLGAP--------

NLNVTFGKKG---A-AAAV------GG-G--------------------------AGGEGAKRSKLS--PAEAAETNGAG------------SQP------
SLPAATVPAG-ESG--LSPNYS--P------------GSGPEAK------AGSGRGR-----------------
---GRRKRD----------SGHVSPAAGATSGAGGFFEKY--------------GPTSGVE--
---SGSPGQGGER------------------------------SGGTPHLHEPPKALSSPPSTW----AKG----------------------GGGGDLLLP--
PPPEQADLLPSLDQA---ESCSPRGGGGGGDFPGEA---------ENEDE------VSSSSDNQ--------------------------------
GAKGCPAGRSPLQPP---GRPVDHPLLN--GQKASALAQLGLHGGGSGSST---------ATSSP----ESY--------GA-------
GPPGPV---------------------------QEEIHPLEILQAQIQLQRQQFSISEDQPLGLK------------SAKKGPEGSG----
GGQNGDS-GELSSCC-----EGAGAKGA--------VSTIDLESLMAEHSA-TWYLPG-------DK-ALLDG----EPDEKV--
LVPWEKAKAPTA-GKEAHDLPSSKAL-------------APAQTGSHLQCLSVHCTDDMGDAKGRT-
AVPTWRSLHSDISNRFGTFVAALT------------------------------------

>B.gargarizans

-------MFGL-EQFEPQ--------INSRS--------AGQGDR------SF-SQP-M----NMSSHFK-NPA-FH----------SA-----G-S--S-
AAVDP-TIGPLNEPS--M-MG--------MNLNM-N--GEPY-GYH-AR-GHSDIH-------------AGGI--------------------QP-------
--------VH----GFFNN-QQQQHH-HGH----P--------HQ-HHPHFS-GNFGGPDATA-SCLHGGRLM----GY--NNNL---GN--
-P----------QAFGE--GYE-QLAEN-Q--------------------AGEGFG----------------QQ---------------------RTGNLPEF--------
QHPNSTASNHAVPAPCLPLD----QSPNRAASFHGLP--SS---------------------TSSDSHSLE----QRR-IHNQ-A--
SVDPLE------------YNYPS-DGPAG---HFEVPVFS-PSD-S--EG----HYGAGRQV--SAG-SFPG-ASVL--PRPPGI--
VGMTKVH--P-------------------------------QQ---QH--GVFFERF--GGARKMPVGMEPA--VNA-RHPLL-QQQ--------QQ--
------------------------------QTGLLARQNSCPPAITR-QQQQTEGSTSNPNL-PDSG-PVMQ-TQHSQ--------
FEYPIQRLENRNM--------------HPY--SES---MFNM---QQGP-----------P----QQPP------NQRLQHFDA-
PYMNVTKRPRFDFPNN-H----G-VDNCAAWNN-----S-SIHNAGMDSHL------------SPS-T-YP-------------GLPSDYTP--
QVPES---F-PPVPALQHPGP----DHQSI-------Q-------------------------QRQN--------AAMMIKQMASRN------------QQQR-
MR-QAN---LQQLGHHGDVN----QS-SIVHGGQVGSMPQ-P-NFDRE------------------------GGRIA-------------------
NFDPQNP-HVGQEN--AWFPG----PH---PPGDIL--------QRRMGGSSIPADPASHD---ISL----QQ--NGSNMLFRP--G--
VNRM-GLQE-S----LG-MA--GEGHVQALHSP-----SMHSQFGNN----ITNLTQMQSPGGG-----VGINSSTSDR-R---
GPPDFTTP---GIGQQSGFPF-VGSNRQ-STPH-NPTGVNSSP--------------------SSY--PPQSDF--QASQR----
STASKLGALSLG--------SFNK----------ASA------------KEN-MFGQSCLAALSTACQNMIASLGAP--------
NLNVTFNKKS---Q-AEGKRKLSQTENELN--N-SSG-N-------------------GTSSEYFP--GASSQANQGPG----
TSNNNSKPAGQ------NGASQPTQGE--TS--LSPNYN--I---------EV-TPGNDGK--
PVT--GGGRGR--GRRKRD----------SGHVSP--------
GNYFDKY------------SADSGGAVVSP-GQQGQSANAVE-------------------------------PGGTPH---D-KPLTSP--SW----
AKG-----GELL------------LGDQPD-LMS--SLD--SGIQSV-TKS---DSSSP-----HVDFS-ED-VNT--TY-GNEDE------
VSSSSDNN-----------------------------ISKPSNCPLVTGSPKIQR------NEHGLLN--GQKPMGLNMLNN------------------
TTSLP----DSYGL---SSTGA---GHP-GTPGME-QVRTP---TSTST------------
QDEIHPLEILQAQIQLQRQQFSISEDQPLGLK--------------NKKTDCTA------QNVD--SELNSCC---S---DNVKNA------
--MSTIDLDSLMAEHNS-TWYMPN-------EK-SLMEG----EEDDKS--ITPWDKSKSQPT-NKEAHELPQNKTS-----------
--AAAQNGSHLQCLSVHCTDDIGESKGRT-PVPTWRSLHSDISNRFGTFVAALT------------------------------------

>C.plagiosum

-------MYSL-DQFSRS--------GGGPG-----------GER------NF-SQAAAL---AMSAHYK-SPT-FP----------GA-----S-G--S-
AMDEQ-TLGPL-EPP--M-LG--------LGMNP-ALGGEAY-SFH-PRGGHSELH------QAP---
AAAAGGSAAAPPPGGPQQPQAPPPPPPAAAA----PPPQPQATH----GYFPGGP--------HPHGHP------------H--FG-
GSFCGTEPGH-SCLHGGRLLGTAAAYSGNPLA---GQ---P----------HAFGD--TYD-PLGEN-QA------
GGGAAAAAAAGGGPGEGFA------Q---------QAPP-----------------VGRSGNLSDY--------HQHHTPSSNH---TPCLPLD---
-QSPNRAASFHGLP--AS---------------------SSSEAHGLE----QRR-LQSQ-P--AVDSME------------YNYQN-EPSAR---
PFDMPVFS-PSE-S--GAQLP-HYGPGRHV--PGG-SFPA-NTTM--PRTPGL--VSLGKVH--P--------------------------Q----
QQ---QH--GVFYERF--GNARKMPVGMESS--VAA-RNPLM------------QQ--------------------------------
QAGLLARQNSCPPAIPR--QQQAETGPPTSSL-QDSG-AMMP-NQHAP--------FEYPIQRLENRNM--------------HPY--
TDP---MFNI------------------------QQQP------NQRLQHFDA-PYLNVAKRPRFDFPNN-H----N-VDNCATWSS-----N-
SMHNASLENHL------------SPS-A-YP-------------GLPNEFSP--PGPEG---F-PPGPPLQHPGT----DQQSL-------Q-----------
-------------QRQN--------MLMMFKQMVSRN------------QRHR-MR-QPE---LQHLSHHADVN----QN-
SIVHSGQVANMSQ-P-GFERE----------------------SGGRMP-------------------SFDPQNP-QMGQEN--AWFPG----HH---
PSGEML--------QRRMGGSAVPPEGSPHESVQMNL----QQ--NGSGMLFRP--G--GNGL-GMQE-P----MR-MP--
GDGHVQGLHSP-----GMHSQFGNS----MGNVAQMQSPSGG-----MGLPSTSTDR-R---AGPDFQGT---
PMGGQPGFPF-GGPNRPS-NPHNNPPGVPPSP-------------------GNY--PPQSEY--QTNQR----PSMSKIGSLSLG--------

SFSK----------PGS------------KDNTIYGQSCLAALSTACQNMIASLGAP--------NLNVTFNKKN---Q-
NEGKRKLSQTEQDSV--GPSSGSGG--GNV-----GGPPTGSTGNGPEYFQ--GSAQQNSQMGV----
SGGGSGKLGTSTVQNSTQGPNPPSQPE--CN--LSPNYA---L---------EA-IP-SEGK--
GQT----GRGR---GRRKRD----------SGHVSP--------GNFFDKY--
---------SAENVNPGVS-P-GQQGQSSHAGD------------------------------RGGTPQ---D-KSLTSP--SW----AKG-----NDLL--
----------LPDQPD-LMS--SLD--SGIQSV-TKS---DGSSP-----QVDFS-DD-VSN--NY-GNEDE------VSSSSDNN-----------
-----------------IPKATRLVTSSPKLQR------ADHGLLS--GQKPMGHGMLNAHPNSN-------------TTSNTGPGADSFGL-
-----GSTGSGHP-GTPGME-QVRTP---TSTST------------QDEIHPLEILQAQIQLQRQQFSISEDQPLAMK-------------
NKKAECPG------QNGD--TELAACS---T---DNSKAA--------ISTIDIESLMAEHNS-TWYMPN-------DK-AMMDP----
QDEDKQ--MAPWEKAKSAST-NKEAHELSQNKAS--------------SVQTGSHLQCLSVHCTDDLTDSKGRS-
PMQTWRSLHSDISNRFGTFVAALT----------------------------------

>T.alba

-------MFGL-EQFEPQ--------SSSRS--------GGQAER------GF-GQPGLS----MSAHFK-APA-FP----------GG-----G-PAAA-
AAVDP-ALGALGEPP--L-LG--------MNM---SLAGDAYG-FP-GR-GPAELH-------------GGGM------Q------------P-P-----
----------VH----GFFGG---QQPH-GGHGGAHH-P------HQ-HPPHFG-GNF-GPDPGA-SCVHGGRLL----GY--SGAL---
GG---Q----------TAFAD--GYE-HMAES-Q-------------------GGEGFG------Q---------Q---------------------RAGTLPDF-------
-QHHSAGASSHAVPAPCLPLD----QSPNRAASFHGLP--AA---------------------GSSEPHGLE----QRR-LPAQ-G--
GVDSLE------------YNYPG-DGPAG---HFELPVFS-PSE-P--EGQLPQHYGGGRQV--PGGGSFAG-APAL--PRAPGM--
A-VAKAH--P-----------------------P----------QQH--GVFFERF--GGARKMSASLEPG--ASA-RHPLM--QQ--------QQ---
PPPP-PPQ-------------------QPPGLLARQNSCPPAIPR--QPQTEANAPNPNL-QDNG-PIMQ-NQHAQ--------
FEYPIHRLENRNM--------------HPY--ADP---VFNM----QHP---P-------P----QQPP------NQRLQHFDA-
PYVSVAKRPRFDFPGN-P----G-VERCASWAG-----G-MH-GPAMESHL------------SPT-A-YP-------------GLPGEFTP--
PAPEA---F---GAPLPHGGP----EHPAL-----A-------------------------QRQN--------AALVMKQMASRS-------------QQR-
LR-PPS---LQQLGHHGEVG----PP---------GGLPP-P-AFERE--------------------PGGGGGR-------------------GFDPPTP-
HMAPDG--AWFAG----PP---PPGELL--------PRRMAAPGLPAEAAPHE---LGL----QP--GGAAVLFRP--G--AGGL-
GLQE-P----LR-MA--GEGPAQALPSP-----GVHPPFAPA----MGGLSQLQSPGGG-----VALPSAPAER-R---
GPADFAAQ---P-----GFPF-AAAARQ-PAAHGAAPALSASP-------------------GAY-PPPPPEF-PPPPPP---
RPAASKLGALSLG--------SFSK----------PAS------------KDN-VFGQSCLAALSTACQNMIASLGAP--------
NLNVTFNKKS---P-AEAKRKLSQAEPDP---------------------------PPPAAPDYFP--AGPAAGAGGAG----------KAVGA--
--------APLLPA-ESS--LSPGYA---L---------EP-AAGGEGK------AGGGRGR-------------
---GRRKRD----------SGHVSP--------GTFFEKF----------SAAEGGGAGVS-P-
GQPAVPAAAGGP-----PGAAGAER----------------GGGTPH---D-KPLTSP--SW----GKG-----GELL------------LGEQPD-
LMS--SLD--SGIQSV-TKS---DGSSP-----HVDFP-DE-VST--SY-GNEDE------VSSSSDNA---------------------------
ASKPTRSPLLGGSPKLPR------GEHALLN--GQKPLALGLLST------------------STSTP----DSYGL---STTAG---AHP-
GTPSME-QVRTP---TSTSA------------QDEIHPLEILQAQIQLQRQQFSISEDQPLGLK--------------SKKGECAG------
QNGD--SDLGSCC---S---EGVKGA--------MSTIDLDSLMAEHNS-TWYLPG-------EK-ALMEG----QEEDKP--
MAPWEKPKPPNP-SKEAHDLPPSKTS-------------AAAQTGSHLQCLSVHCTDDVGEAKGRT-
AVPTWRSLHSDISNRFGTFVAALT----------------------------------

>B.musculus

-------MFGL-DQFEPQ--------INSRN--------AGQGER------NF-NEAGLS----MNAHFK-APA-FH----------AG-----G-P--P-
GPVDP-TMSGLGEPP--I-LG--------MNM-------EPYG-FH-AR-GHSELH-------------AGGL------Q------------AQP----------
-----VH----GFFGG---QQPH-HGHPGGHH-P------HQ-HHPHFG-GNFGGSDPGA-SCLHGGRLL----GY--GGAA---
GGLGSQ----------PPFAE--GYD-HLAES-Q-------------------GPESFG------P---------Q---------------------RPGNLPDF-------
---HSSGASGHAVPAPCLPLD----QSPNRAASFHGLP--AS---------------------SGSDSHSLE----PRR-VANQ-G--
AVDSLE------------YNYPG-EPPSG---HFDM--FS-PSD-S--EGQLP-HYAAGRQV--P-GGSFPG-ASAM--PRAAGM--
VGLSKMH--A--------------------------QQQQQQQQQH--GVFFERF--GGTRKMPVGLEPA--VGS-RHPLM--QP--------
PQQAPPPPPQQQP--PPPPP-PPQQQQPPPPPPPPGLLVRQNSCPPALPR--PQQSEAGTPSGGL-QDGG-PMLP-
SQHAQ--------FEYPIHRLENRSM--------------HPY--SEP---VFNM----QHP---P-------P----QQTP------
NQRLQHFDAPPYMNVAKRPRFDFPGS-A----G-VDRCASWNG-----S-MH-NGALDNHL------------SPS-A-YS----------
---GLPGEFTP--PVPDS---F-PSGPPLQHPAP----DHQSL-----QQQQQQQQQQQQQQQQQQQQQQ------QRQN--------
AALMIKQMASRN------------QQQR-LR-QPN---LAQLGHPGDVG----QG-GLVHSGPVGGLAQ-P-NFERE---------------
-------SGGAGA---------------GRLGTFEQQAQ-HLAQES--AWFPGPH---P---PPGDLL--------
PRRMGGSGLPADCGPHD---PGLAPPPPP--GGSGVLFRG--S--------LQE-P----LR-MP--GEGHVPALPSP-----GL--
QFGGS----LASLGQLQSPGAG-----VGLPSAPSER-R--PQQPPDFTAP---ALGGQPGFPF-GAANRQ-ATPHSG-

PGVNSPPSAGGGGGS-T-GGGGGGG--AY--PPQPDF--QPSQR----TSASKLGALSLG--------SFNK----------PSS---------
---KDN-LFGQSCLAALSTACQNMIASLGAP--------NLNVTFNKKN---P-PEGKRKLSQNETDGA-----------------------
AMASNPGSDYFP--GGTAPGAPGPGGQSGTSSSGSKASGP--------PNPPAQGD-GTS--LSPNYT--------------------------
-----------------L---------ES-TSGNDGK---PVPGGGGRGR--
GRRKRD----------SGHVSP--------GTFFDKY--------SAAPDSGGAPGVS-P-GQQQAPGAAVGG------SSTGEAR---------
-------GAPTPH---E-KALTSP--SW----GKG-----AELL------------LGDQPD-LMA--SLD--GG-----AKS---DGSSP----
HAGEFASDE-VST--SY-ANEDE------VSSSSDNPPA--------------------------LAKASRSPLVTGSPKLPP-
RGLGAGEHGPKA--PPAPLGLGILST------------------STSTP----DSY--------GGGGTGHP-GTPGLE-
QVRTPTSSSGAPP------------PDEIHPLEILQAQIQLQRQQFSISEDQPLGLK-------------GGKKGECAV-
GASGAQNGD--SELGSCC---S---EAVKSA--------MSTIDLDSLMAEHSA-TWYLPA-------DK-ALVDG----ADEDKT--
LAPWEKAKPQNPNSKEAHDLPPNKAS--------------ATQPGSHLQCLSVHCTDDVGDAKARA-
SVPTWRSLHSDISNRFGTFVAALT----------------------------------

>B.taurus

-------MFGL-DQFEPQ--------INSRN--------AGQGER------NF-NEAGLS----MNAHFK-APA-FH----------AG-----G-P--P-
GPVDP-AMSGLGEPP--I-LG--------MNM-------EPYG-FH-AR-SHSELH-------------AGGL------Q------------AQP----------
-----VH----GFFGG---QQPH-HGHPGGHH-P------HQ-HHPHFG-GNFGGPDPGA-SCLHGGRLL----GY--GGAA---
GGLGSQ----------PPFTE--GYD-HMAES-Q-------------------GPESFG------P---------Q----------------------RPGNLPDF------
----HSSGASGHAVPAPCLPLD----QSPNRAASFHGLP--AS---------------------GGSDSHSLE----PRR-VANQ-G--
AVDSLE------------YNYPG-EPPSG---HFDM--FS-PSD-S--EGQLP-HYAAGRQV--P-GGSFPG-ASAM--PRAAGM--
VGLSKMH--A------------------QQQQQQQQQQQQQQQQQQQH--GVFFERF--GGTRKMPVGLEPG--VGS-RHPLM--
QP--------PQQAPPPPQQPPP--QQAPP-PPQQPPPQQPPPPPGLLVRQNSCPPALPR--PQQGEAGTPSGGL-QDGG-
PMLP-NQHAQ--------FEYPIHRLENRSM--------------HPY--SEP---VFNM-----------------------QQAP------
NQRLQHFDAPPYMNVAKRPRFDFPGS-A----G-VDRCASWNG-----S-MH-NGALDNHL------------SPS-A-YS----------
---GLPGEFTP--PVPDS---F-PSGPPLQHPAP----DHPSL-----QQQQQQQQ---------QQQQ------QRQN--------
AALMIKQMASRN------------QQQR-LR-QPN---LAQLGHPGDVG----QG-GLVHSGPVGGLGQ-P-NFERD---------------
-------SGGAGA---------------GRLGTFEPQAQ-HLAQES--AWFPGPH---P---PPGDLL--------
PRRMGGSGLPSDCGPHD---PGLAPPPPP--GGSGVLFRG--S--------LQE-P----LR-MP--GEGHVPGLPSP-----GL--
QFGGS----LASLGQLQSPGAG-----VGLPSAPSER-R--PQPPDFTAP---ALGGQPGFPF-GAANRQ-ATPHSG-
PGVNSPPSAGGGGGS-TGGGGGAGGGGAY--PPQPDF--QPSQR----TSASKLGALSLG--------SFNK----------PSS------
------KDN-LFGQSCLAALSTACQNMIASLGAP--------NLNVTFNKKN---P-PEGKRKLSQNETDGA---------------------
--AVAGNPGSDYFP--GGTAPGAPGPGGPSGTSSSGSKASGP--------PNPPAQGD-GTS--LSPNYT-------------------------
------------------L---------ES-TSGNDGK---PVPGGGGRGR--
GRRKRD----------SGHVSP--------GTFFDKY------SAASAPDSGGAPGVS-P-GQQQAPGAAVGG-------NSGEVR-------
---------GAPTPH---E-KALTSP--SW----GKG-----AELL------------LGDQPD-LMA--SLD--GG-----AKS---DSSSP----
HVGEFASDE-VST--SY-ANEDE------VSSSSDNPPA--------------------------LAKASRSPLVTGSPKLPP-
RGVGTGEHGPKA--PPPPLGLGILST------------------STSTP----DSY--------GGGGTGHP-GTPGLE-
QVRTPTSSGGAPP------------PDEIHPLEILQAQIQLQRQQFSISEDQPLGLK-------------GGKKGECAV-
GASGAQNGD--SELGSCC---S---EAVKSA--------MSTIDLDSLMAEHSA-TWYMPA-------DK-ALVDG----TEEDKT--
LAPWEKAKPQNPNSKEAHDLPANKAS--------------APQPGSHLQCLSVHCTDDVGDAKARA-
SVPTWRSLHSDISNRFGTFVAALT----------------------------------

>G.gallus

-------MFGL-EQFEPQ--------MSSRS--------GGQGER------GF-GQPGLS----MSAHFK-APA-FP----------GG-----G-P--A-
AAVDP-ALGALGEPP--L-LG--------MNM---SLPGDGYG-FP-GR-GPAELH-------------GAGM------Q------------P-P------
---------VH----GFFGG---QQPH-GGHGGAHH-PHQHQHQHQ-HQPHFG-GNF-GPDPGA-SCVHGGRLL----GY--
NGAL---GG---Q----------TAFAD--GYE-HMAES-Q-------------------GAEGFG------Q---------Q----------------------
RAGNLPDF--------QQHSAGASGHAVPAPCLPLD----QSPNRAASFHGLP--AA--------------------GSSEPHGLE----
QRR-LPAQ------DSLE------------YNYPG-DGPAA---HFELPVFS-PSE-P--EGQLP-HYGGGRQV--PAGGGFAG-T-AL--
PRAPGM--A-VAKAQPPP----------------------PPPPPPQQPQQQQH--GVFFERF--GGARKMPASLEPG--ASA-RHPLM-
-QQ--------QQ---------PQ-------------------QPPGLLARQNSCPPAIPR--QQQTEANAPNPNL-QDNG-PIMQ-NQHAQ-----
---FEYPIHRLENRNM--------------HPY--ADP---VFNM----QHP---P-------P----QQPP------NQRLQHFDA-
PYVSVAKRPRFDFPGN-P----G-VERCASWAG-----G-MH-GPAMESHL------------SPT-A-YP-------------GLPGDFTP--
PAPDA---F---GGPLPHGGP----EHPAL-----A-------------------------QRQN--------AALVMKQMASRS-------------QQR-
LR-PPS---LQQLGHHGEVG----PP---------GSLPP-P-GFERE----------------------AAAAAR-------------------GFDAPAP-
HLAPDG--TWFAG----PP---PPGELL--------PRRMAGPGLPAEAAPHE---LGL----QP--GGAAVLFRP--G--AGAL-

GLQE-P----LR-MA--GEGPAQALPSP-----GVHPPFAPA----VGGLSQLQSPGGG-----VALPSAPAER-R---
GPADFAAQ---P-----GFPF-GAAARQ-PPAHGNAPALSASP-------------------GAY-PPPPPEFPPPPPPP---
RPAASKLGALSLG--------SFSK----------PAG------------KDN-VFGQSCLAALSTACQNMIASLGAP--------
NLNVTFNKKS---P-AEAKRKLSQAEPDP--------------------------PPPAAPDYFP--AGPTAG-GGAG----------KA----------
---APLLPA-ESS--LSPGFA---L---------EP-AAGGEGK------AGGGRGR-------------------
--GRRKRD----------SGHVSP--------GTFFEKF----------SAAEGGGAGVS-P-
GQPAPPAAGGPP------GAAGAER----------------GGGTPH---D-KPLTSP--SW----GKG-----GELL------------LGEQPD-
LMS--SLD--SGIQSV-TKS---DGSSP-----HVDFA-DE-VST--SY-GNEDE------VSSSSDNA---------------------------
GPKPTRSPLLGGSPKLPR------GEHALLN--GQKPLALGLLNT------------------STSTP----DSYGL---SSTAG---AHP-
GTPGME-QVRTP---TSTSA------------QDEIHPLEILQAQIQLQRQQFSISEDQPLGLK--------------SKKGECAG------
QNGD--SDLGGCC---P---EGVKNT--------MSTIDLDSLMAEHNS-TWYLPG-------EK-ALMEG----QEEDKP--
MAPWEKPKPPNP-SKEAHDLPPSKTS-------------ATAQTGSHLQCLSVHCTDDVGEAKGRT-
AVPTWRSLHSDISNRFGTFVAALT----------------------------------

>S.meridionalis

---MNTNYN-GSG-FD----------MK-----A-P--S-VGVEP-VLGPLNEPP-
-M-QG--------LGFTS-G--REQY-GFQ-PH-SHGDML-------------NIGLQQQQQQQ-----------------------------LHM--
PLPFNN---QQLN-PDQP---------------SHPYQD---------GVTSCLHGDRHI----GF-SGSNT---GH---Q----------HMFET--
EFN-HFTEA-Q-------------------SRECHSQQQ------------Q---------------------RLASMPDY--------
QLHSHPNGNHAVPAPCLPLD----QSPNRAASFHGLP---S---------------------SSPESNRLE----HYR-LFPQ-G--
RVGGSQ------------YCYPC-DPISG---HFDMAGFSTPDS-T--EPKLS-YCEPGNQM--VGG-NFPTFNQSG--SRAPMI---
GGSKVD--Q--------------------------------QLPQQ--NTYSERF--GNRGK----LEPG--VTP-RHHLM---A--------QQ------------
-------------------RAGPMARQNPGSTVLPR-LYHTPEFVPNSPDM-QNNN-VVL----HGQ--------MDHPIHRLNNHNM---
-----------HPF--GEH---MFN----PQFG----------------PQPP-----HQQHLNSF---PYLNTAKRPRFDAPNG------
SAGESCCPLRS-----G-LHNRPGLENHL------------SPS-A-FP-------------TPMGEFTT--HVTDG---F-QSGPPPLSCGS---
HQQQPL-------A------------------------RQQN--------AAMMIKQMASRN------------QQQR-MR-QAD---LQPINQHGDVS-
---QNG-MVHRGPIGGMSH-L-NFDKK----------------------HNFHG--------------------NFDTQNH-HLPHEN--SWFSD----
PH------------------------QQCREANIHG---MEQ----SQ--NGHD-IFRS----------GVTT---------------VEMQSLNSP-----
GPQNQFENS----MNNPLQVQTSDDG-----TMQPNAPMNR-R----QGDFGGA---AIRRQHSFPP-GGPNQQ-
GTPQSNPPGFNSSP-------------------GNY--PSHPEY--ISSQH----LSVNKLGALSLG--------NLNK----------AST------------
KDS-VFGQSCLAALSTACQNMIASLGAP--------NLNVTFNKKS---Q-NEAKRKPGQVEKDINSSGGVGAC------------
--------GPGAEYFQ--SSASQNSQTPC----SGNNNNAAAGQ------SALGQMAKRE-AAT--LSPDNA-------------------------
------------------V---------DS---GNEGK--AAI--GNGRGR---
GKRRRD----------SGHISP--------GNFSPSC--------------SSNPVVS-P-GQQASSISIGAEG---------------------------
RGRTPE-----SVLVSP--SF----GKP--------------------------D-LTT--SVD--SGIQSM-GKS---DGVSP-----CMDYL-DD-
ASP--NY-VNEDV--RTNRTSVKCSSDN-RS--
---------------------GYS-DTPCME-QVRTP---LDTSA------------QDEVHPLEILQAQIQLQRQQFSISEDQPLSGK------------
--TGKKPDCQS-----GLNGD--CAMASCS---P---EPGKGT--------VNTIDLDSLMTEQRA-TWYGPS-------NK-ALIED---
-SRNGKC--MGFWDRARGQSD-NKEGHG---

>L.chalumnae

-------MFGL-EQFEPQ--------INNRN--------VGQGDR------NF-SQPGL----NMSSHYK-NPP-FH----------SG-----G-P--P-
GAVEQ-SINSLNEPP--M-LG--------MNMGL-N--GEQY-GLH-AR-GHSDIH------------TGGI------Q------------QQG-----
----------IH----GFFNN---QQPH-HGHPHAHH-P----HPHQ-HHPHFS-GNFAGPDPST-SCLHGGRLM----GY-NNSNL---
GA---Q----------QPFPE--GFD-PMAEN-Q-------------------GGEAFG----------------QQ--------------------RSGNMPDF-------
-QHHNPAAANHAVPAPCLPLD----QSPNRAASFYGLP--SS---------------------ASSDAHGLE----QRR-MQNQ-G--
GVESLE------------YNYPN-DPPSG---HFDMPVFS-PSE-S--DAQLP-HYNAGRQV--PAG-TFTG-NSTL--PRAPGM--
VGMSKVH--P-----------------------------QHQ---QH--GVFFERF--GSTRKMSVGMEPG--VNS-RHPLM--QQ--------QQ---
----------------------------QAGLLARQNSCPPAIPR--QQQPEANVPNPNL-QDNG-AMMQ-TQHAQ--------
FEYPIHRLENRNM--------------HPF--GDP---MFSM----------------------QQPP------NQRLQHFDA-
PYLNVPKRPRFDFPSN-H----N-VEGCPTWNN-----S-NMHNAGIENHL------------SPS-A-YP-------------GLPSEFTP--
PVPEG---F-PPGPPLQHPGP----DQQSL-------Q------------------------QRQN--------AAMMIKQMASRN------------QQQR-
MR-PPN---LQQLAHHGDVN----QN-NMVHGGQVGNLSQ-P-NFERD---------------------NGGRMN-------------------
NFDPQNP-HMSQEN--AWFPG----PH---PPGEML--------QRRMGGSNHPTETSPHE---INL----QQ--NGPNMIFRP--G--
VNGM-GMQE-T----MR-MP--GEGHVQSLHSP-----GMHTQFGNN----MGNLSQMQSPGGG-----VGISSTPSDR-R---

PPSDFPPP---SMGGQPGFPF-GPSNRQ-ATPHTNPAGVGTSP-------------------GTY--PPQSEF--QSSQR----
TSASKLGALSLG--------SFSK----------PSS------------KDN-MFGQSCLAALSTACQNMIASLGAP--------
NLNVTFNKKN---Q-NEGKRKLSQTEQDIN--G-NGG-NN---------------GSNSGSEYFQ--GGTTQSNQMPS----
AGNGNSKSTGQ------NGTNQPNQGE--TS--LSPNYN---M---------EA-TPGNDGK--
PVT--GGGRGR--GRRKRD----------SGHVSP--------
GNFFDKY------------SADSGSTVVSP-GQQGQPANVGE-----------------------------RGGTPH---DNKSLTSP--SW----
GKG-----NDLL------------LNDQPD-LMS--SLD--SGIQSV-TKS---DSSSP-----HVDFP-DD-VST--NY-GNEDE------
VSSSSDNN----------------------------ISKPNRSPLVAGSPKMQR------GDHGLLN--GQKPMGLGMLNN-----------------
TTSNP----DSYGL---SSTGG---GHP-GTPGLE-QVRTP---TSTST------------
QDEIHPLEILQAQIQLQRQQFSISEDQPLGMK--------------NKKTECPG------QNGD--NELSSCG---T---DNGKNP------
--MSTIDLDSLMAEHNS-TWYMPS-------DK-SMMEG----QEDDKS--MAPWEKTKPQNT-NKEAHDLPQNKTS--------
-----AAAQTGSHLQCLSVHCTDDIGESKGRT-PMPTWRSLHSDISNRFGTFVAALT------------------------------------

>T.truncatus

-------MFGL-DQFEPQ--------INSRN--------AGQGER------NF-NEAGLS----MNAHFK-APA-FH----------AG-----G-P--P-
GPVDP-AMSGLGESP--I-LG--------MNM-------EPYG-FH-AR-GHSELH-------------AGGL------Q------------AQP---------
------VH----GFFGG---QQPH-HGHPGGHH-P------HQ-HHPHFG-GNFGGSDPGA-SCLHGGRLL----GY--GGAA---
GGLGSQ----------PPFAE--GYD-HLAES-Q-------------------GPESFG------P---------Q----------------------RPGNLPDF-------
---HSSGASGHAVPAPCLPLD----QSPNRAASFHGLP--AS--------------------SGSDSHSLE----PRR-VANQ-G--
AVDSLE------------YNYPG-EPPSG---HFDM--FS-PSD-S--EGQLP-HYAAGRQV--P-GGSFPG-ASAM--PRAAGM--
VGLSKMH--A--------------------------QQQQQQQQQH--GVFFERF--GGTRKMPVGLEPA--VGS-RHPLM--QP--------
PQQAPPPPPQQPPQQPPPPP-PQQQQPPPPPPPPPGLLVRQNSCPPALPR--PQQGEAGTPSGGL-QDGG-PMLP-
SQHAQ--------FEYPIHRLENRSM--------------HPY--SEP---VFNM----QHP---P-------P----QQTP------
NQRLQHFDAPPYMNVAKRPRFDFPGS-A----G-VDRCASWNG-----S-MH-NGALDNHL------------SPS-A-YS----------
---GLPGEFTP--PVPDS---F-PSGPPLQHPAP----DHQSL-----QQQQQQQQQQQQQQQQ-QQQQ------QRQN--------
AALMIKQMASRN------------QQQR-LR-QPN---LAQLGHPGDVG----QG-GLVHSGPVGGLAQ-P-NFERE---------------
-------SGGAGA---------------GRLGTFEQQAQ-HLAQES--AWFPGPH---P---PPGDLL--------
PRRMGGSGLPADCGPHD---PGLAPPPPP--GGSGVLFRG--S--------LQE-P----LR-MP--GEGHVPALPSP-----GL--
QFGGS----LASLGQLQSPGAG-----VGLPSAPSER-R--PQPPDFTAP---ALGGQPGFPF-GAANRQ-ATPHSG-
PGVNSPPSAGGGGGS-TGGGGGGGG--AY--PQQPDF--QPSQR----TSASKLGALSLG--------SFNK----------PSS--------
----KDN-LFGQSCLAALSTACQNMIASLGAP--------NLNVTFNKKN---P-PEGKRKLSQNETDGA-----------------------
AMASNPGSDYFP--GGTAPGAAGPGGQSGTSSSGSKASGP--------PNPPAQGD-GTS--LSPNYT--------------------------
-----------------L---------ES-TSGNDGK---PVPGGGGRGR--
GRRKRD----------SGHVSP--------GTFFDKY--------SAAPDSGGAPGVS-P-GQQQAPGAAVGG------SSTGEAR---------
-------GAPTPH---E-KALTSP--SW----GKG-----AELL-----------LGDQPD-LMA--SLD--GG-----AKS---DGSSP----
HAGEFASDE-VST--SY-ANEDE------VSSSSDNPPA--------------------------LAKASRSPLVTGSPKLPP-
RGLGAGEHGPKA--PPAPLGLGILST------------------STSTP----DSY--------GGGGTGHP-GTPGLE-
QVRTPTSSSGAPP------------PDEIHPLEILQAQIQLQRQQFSISEDQPLGLK-------------GGKKGECAV-
GASGAQNGD--SELGSCC---S---EAVKSA--------MSTIDLDSLMAEHSA-TWYLPA-------DK-ALVDG----ADEDKT--
LAPWEKAKPQNPNSKEAHDLPPNKAS--------------ATQPGSHLQCLSVHCTDDVGDAKARA-
SVPTWRSLHSDISNRFGTFVAALT----------------------------------

>R.temporaria

------MMFGL-EQFEPQ--------INSRSSSTSSSSAAGQGER------NF-A---------MSAHFK-SAA-FH----------PG-----G-T--S-
GAVDP-AMGALGEPA--M-LG--------INLNL-NGGGEPY-GYH-SR-GHSDLH-------------PAGM--------------------QP----
-----------VH----GFFNN-QPPHHH-HSHPN--P--------HQ-HHPHFP-ANFAGPEPTA-SCLHGGRLM----AY--NNNL---
GN---Q----------QGFGE--GYE-QMPEGQQ-------------------SGEGFG----------------QQ--------------------RSGNLPDF------
--QHANSTASNHAVPAPCLPLD----QSPNRASSFHGLP--AS--------------------TSSDSHNLE----QRR-IHNQ-A--
GVDALE------------YNYPS-DGPSG---HFEVPVFS-PSE-S--EG----HYGAGRQV--PNS-AFPG-ASVL--PRPPGM--
VGMSKVH--P-----------------------QQQQQQQQ---QH--GVFFERF--GGARKIPVGMEPA--VNA-RHPLL-QQQ--------
QQ-------------------------------QTGLIARQNSCPPAIAR--QQQTEGNTSNPNL-PDNG-PVMQ-SQHTQ--------
FEYPIQRLENRNM--------------HPY--SES---MFNM---QQGP-----------P----QQPP------NQRLQHFDA-
PYMNVTKRPRFDFSNN-H----G-VENCAAWNN-----N-SIHNAGIDSHL------------SPS-T-YP-------------GLPGEYPP--
QVPDS---F-PPGPALQHPGS----DHQSL-------Q-----------------------QRQN--------AAMMIKQMASRN------------
QQQR-MR-QAN---LQQLGHHGDVN----QS-GIVHGGQVGSIPP-P-NFDRE-----------------------GGRIG-------------------
SFDPQNP-HVGQEN--AWFPG----PH---PPGDIL--------QRRMAGSNLPTDPTSHD---INL----QQ--NGSNMLFRP--G--

VNRM-GMQE-P----LG-LP--GEGHVPALHSP-----GMHSQYGNN----MANLSQMQSPGGG-----VGMNSTPADR-R---GAPDFAAP---AIGQQPGFPF-VGSNRQ-TTPH-NQPGVNSSP------------------NSY--PPQSDF--QASQR----STASKLGALSLG--------SFSK----------ANA------------KEN-MFGQSCLAALSTACQNMIASLGAP--------NLNVTFNKKS---Q-AEGKRKLSQTETELN--G-NSG-N------------------SSTSDYFS--GGSSQGNQGPG----ATTNNSKSTGQ------SGTSQPTQGE--TS--LSPNYN--I---------EV-TPGNDGK--PVT--GGGRGR--GRRKRD----------SGHVSP--------GTYFDKY------------SADSGGAVVSP-GQQGQTANPVE------------------------------PGGTPH---D-KPLTSP--SW----GKG-----NELL------------LSDQPD-LMS--SLD--SGIQSV-TKS---DSSSP-----HVDFS-ED-VNT--TY-GNEDE------VSSSSDNN----------------------------ISKPNNCPLVTGSPKIQR------SEHGLLN--GQKPMGLNLLNN------------------TTSLP----DSYGL---SSTGA---GHP-GTPGME-QVRTP---TSTST------------QDEIHPLEILQAQIQLQRQQFSISEDQPLGMK-------------NKKSDCTA------QNVD--SELNSCC---S---DNVKNS--------MSTIDLDSLMAEHNS-TWYLPN-------EK-SLMEG----EEDDKS--ITPWEKSKSQQT-NKEAHDLPQNKTS-------------AAAQNGSHLQCLSVHCTDDIGESKGRT-PVPTWRSLHSDISNRFGTFVAALT----------------------------------

>B.bufo

-------MFGL-EQFEPQ--------INSRS--------AGQGDR------SF-SQP-M----NMSSHFK-SPA-FH----------PA-----G-S--S-AAVDP-TIGPLNEPS--M-MG--------MNLNM-N--GEPY-GYH-AR-GHSDIH-------------AGGM-------------------QP---------------VH----GFFNNQQQQQHH-HGH----P--------HQ-HHPHFS-GNFGGPDATA-SCLHGGRLM----GY--NNNL---GN---P----------QAFGE--GYE-QLAEN-Q------------------AGEGFG----------------QQ--------------------RTGNLPEF--------QHPNSTASNHAVPAPCLPLD----QSPNRAASFHGLP--SS---------------------TSSDTHSLE----QRR-IHSQ-G--SVDPLE------------YNYPS-DGPAG---HFEVPVFS-PSD-S--EG----HYGAGRQV--SAG-SFPG-ASVL--PRPPGI--VGMTKVH--P-------------------------------QQ---QH--GVFFERF--GGARKMPVGMEPA--VNA-RHPLLQQQQ--------QQ------------------------------QTGLLARQNSCPPAITRQQQQQTEGSTSNPNL-PDSG-PVMQ-TQHSQ--------FEYPIQRLENRNM--------------HPY--SES---MFNM---QQGP-----------P----QQPP------NQRLQHFDA-PYMNVTKRPRFDFPNN-H----A-VDNCAAWNN-----S-SIHNAGMDSHL------------SPS-T-YP-------------GLPSDYTP--QVPES---F-PPVPALQHPGP----DHQSI-------Q------------------------QRQN--------AAMMIKQMASRN------------QQQR-MR-QAN---LQQLGHHGDVN----QS-SIVHGGQVGSMPQ-P-NFDRE-----------------------GGGIG-------------------NFDPQNP-HVGQEN--AWFPG----PH---PPGDIL--------QRRMGGSSIPADPASHD---ISL----QQ--NGSNMLFRP--G--VNRM-GLQE-S----LG-MA--GEGHVQALHSP-----SMHSQFGNN----ITNLTQMQSPGGG-----VGINSSTSDR-R---GPPDFATP---GIGQQSGFPF-VGPNRQ-STPH-NPTGVNSSP------------------SSY--PPQSDF--QASQR----STASKLGALSLG--------SFNK----------TSA------------KEN-MFGQSCLAALSTACQNMIASLGAP--------NLNVTFNKKS---Q-AEGKRKLSQTENELN--N-SSG-N------------------GTSSEYFP--GASSQANQGPG----TSNNNSKPAGQ------NGASQPTQGE--TS--LSPNYN---I---------EV-TPGNDGK--PVT--GGGRGR---GRRKRD----------SGHVSP--------GNYFDKY------------SADSGGAVVSP-GQQGQSANAVE------------------------------PGGTPH---D-KPLTSP--SW----AKG-----GELL-----------LGDQPD-LMS--SLD--SGIQSV-TKS---DSSSP-----HVDFS-ED-VNT--TY-GNEDE------VSSSSDNN----------------------------ISKPSNCPLVTGSPKIQR------NEHGLLN--GQKPMGLNMLNN------------------TTSLP----DSYGL---SSTGA---GHP-GTPGME-QVRTP---TSTST------------QDEIHPLEILQAQIQLQRQQFSISEDQPLGMK--------------NKKTDCTA------QNVD--SELNSCC---S---DNVKNS--------MSTIDLDSLMAEHNS-TWYMPN-------EK-SLMEG----EEDDKS--ITPWDKSKSQPT-NKEAHELPQNKTS-------------AAAQNGSHLQCLSVHCTDDIGESKGRT-PVPTWRSLHSDISNRFGTFVAALT----------------------------------

>P.muralis

-------MFGL-EQFEPQ--------GSGRSGERGGGGGGGFGPG-------------------MGSHFK-PPP-FH-----------------G----GTPGGEAGTLGALSEPASAM-LA--------MN--L-NLPGEAY-GFHASRGGHADLQ-------------QAQP-------------------------------------VH----GFFGG---QQPS-HGAHQQ-P-----------PPSHFG-SGGFGAEPSA-SCLHGGRLLSYPGGQ-------------------------QSFAAD-GYEQHLAEGQA-------------------GADGFGQQ-------------------------------------RAGPLQDF--------QPPPQQHHNPGVPAPCLPLD----QSPNRAASFHGLP--GA---------------------ATSEPHGLD----SQRRLPSQ-AAAAVETLE------------YSYPS---GES---HFDLPVFS-PSE-A--DGQLP-HYGASRQQVPPGGGNFPGPSSAL--PRGPG----GLGKVH--P-----------------------------QQQQQQQHGGGVFFERF--GGARKMSVGLEPS--LGTGRHPLM------------QQPPP----------------------------PGALLARQNSCPPALPR-QQPCPEGNAANPGL-QDGG-PLLQ-GQHAQ--------FEYPIHRLENRSL-------------QHPYGPGEP---VFSV---HHHPPA-------------QQPP------SQRLQHFDAAPYMNVAKRPRFDSWSG--------GGS-----------GAGMHSASLDSHL------------SPSAA-YP------------GLPGDFTP--PGPES---FPPPGADHQAALQ---QQQQQQ--------------------------------QRQN--------AALMIKQMAASR---------SQQQQQQRLR-QPS---LQQLSHHHAGH----------------------HHHHA---GHHHGEPAFEAQEG--AWFPA----PH---PPSA------------------------------------AP--GAGDLLFRP--G--VGGMPGLQE-PPP--PLRIPSGGEGHVP---SPG----SLHGQFGLS--------------------------PPSERSR--PGPPDFAAQ--------QGFPF-

GASSRQ-ATPHSASPASYGPP-------------------TDF--QPSG-P--PPPRP---PQSSSKLGALSLG--------
SFSKGSVAGGVAGPPG----GGGGTPGPKESSGLFGQSCLAALSTACQNMIASLGAP--------NLNVTFGKKG---
APAAGSGGVGAG--------------------------------SEGPKRSKLGPAEPPE---------PGSGGPQPPAP-------APPAPGESG-----
-LSPNYS---P------------GPGPDAKAG------GGRGR--
---------------------------GRRKRD----------SGHVSP-AAGGGGGSSFFDKY--------------GPTAGVE--------GGSPGQG------
----AER----------------GGGTPHLHEPHKALSSPPSAWAKGGGGG--G---DLL-LPPPPL-----SE-QPE-LLP--ALE----------
KA---DSCSP---RGAGDFP-DE------A--GNEDE------VSSSSDNQ-------------------------------LAKGCPAGRSPVQPR-----
PGDHALLN--GQKA-SLAQLSLHAGAGSN-----------STSSS----DSY--------GG---GPPAGAPV----------------------------
QDEIHPLEILQAQIQLQRQQFSISEDQPLGLK------------SAKKPPEGPAGAAGGGSQSGDSAELSSCC----
AAVESGKGA--------MSTIDLDSLMAEHSA-AWYLPS-------DK-ALLEG---QDDDDKA--LAPWEKAKPPNP-
SKEAHDLPSSKSS-------------AAAQSGSHLQCLSVHCTDDMGEAKGRT-AVPTWRSLHSDISNRFGTFVAALT------
----

>V.ursinus

-------MFGL-DQFEPP--------ISNRN--------AGQGER------NF-GEPGLS----MNGHFK-APA-FH----------AG------------
GPADP-TIGALGEPP--L-LG--------MNM---GMAGEAYG-FH-GR-GHSELH-------------AGGL------Q------------PQP-----
----------VH----GFFGS---QQPH-HGHPGGHH-P------HQHHHPHFG-GSFAGPDPGA-SCLHGGRLL----GY--GGGG---
GGLGSQ----------PGFAE--GYE-HMADT-Q-------------------GGEGFG------Q---------Q---------------------RPGNIPEF-----
---QHHSSGPSGHAVPAPCLPLD----QSPNRAASFHGLP--AS--------------------SSSDSHSLE----ARR-MANQ-G--
GVESLE------------YNYPG-DGPSG---HFDLPVFS-PSE-S--EGQLP-HYGTGRQV--P-GGAFPG-ASAL--PRAPAM--
VAMSKIH--P---------------QQQQQQQQQQQQQQQQQQQQHGG--GVFFERF--GGARKMSVGMEPG--VNAARHPLM-
------------QQAPQPPQPQPP---PPQP-QPQPPPP---QQQPSLLARQNSCPPAIPR--QPQAEGNPAGGSL-QDSG-PILQ-
NQHAQ--------FEYPIHRLENRSM--------------HPY--SDP---VFTM----QHP---PPPPAPQQP----PQAP------
NQRLQHFDA-PYLNVAKRARFDFPST-P----T-VDRCASWNG-----S-LHSSGALDNHL------------SPS-A-YP------------
GLPGDFTP--PLPES---F-PPGPALQHPGP----DPQALQQQQQQQQQQQQQ---------QQQQ------QRQN--------
AALMMKQMASRTQQQQQQQQQQQQQQQQR-LR-QPG---LAPLSHPGDVG----PGSGLVHGGPVASLAQ-A-
NFERE---------------------GGGGGG-G------GGGGGGAGRMGSFDPQNP-HLAPES--AWFPGPHPPPP---PPGDLL------
--PRRLGSSNLPPDGSPHE---LGLA----Q--GGPGMLFRG--P-GVTGL-GLQE-S----LR-MP--GEGHVPALHSP-----
GLHSQFGGS----LGGLGQLQSPGAG-----VGLPSAPSDR-R--APPPDFAAP---PLGGQPGFPF-GGTSRQ-ATPLSN-
PGVSASP---------GGGGGGGGGGGAY--PPQPDF--QPSQR----ASASKLGALSLG--------SFNK----------PNS------------
KDN-LFGQSCLAALSTACQNMIASLGAP--------NLNVTFNKKN---Q-SESKRKLSQTDSDGAVGSGGG----------------
--GGAGSSGPDYFP--SGTAPGGPGPGGPGGTSGGGGKAPGP--------PNPPAPGD-GTS--LSPNYT------------------------
-------------------I---------EA-ASGNDGK---PVPGGGGRGR---
GRRKRD----------SGHVSP--------GTFFDKF------SSAAAAADSGGSGVS-P-GQQAAPGPPGGS-------SGGESR---------
-------GAPTPH---D-KALTSP--SW----GKG-----AELL------------LGDQAD-LMS--SLD--SGIQSG-AKS---AGSSP----
HVGDFA-DE-VSA--GY-ANEDE------VSSSSDNAASA------------------------LAKPSRSPLVTGSPKIPR------
GPAGEHG--QKAAPGLGGLLTT-----------------STSTP----DSYGLSSSS--GA---AHP-GTPGLE-QVRTP--TSSTSP-------
-----QDEIHPLEILQAQIQLQRQQFSISEDQPLGPK------GGSGGGGGGKKAECAG-----GQNGD--SELGACC---P---
EAVKSA--------MSTIDLDSLMAEHGS-TWYMPG-------EK-ALLDG----PEDDKT--
LAPWEKSKPQNPVSKEGHDLPQNKTS-------------AAAQTGSHLQCLSVHCTDDVGEAKGRT-
SVPTWRSLHSDISNRFGTFVAALT----------------------------------

>P.guttatus

-------MFAL-DPFDPHGGGGGGGGGSGRS--------GVPGER----GGGF-GPG-------AGSQFK-PSP-FH----------GG-----S-
S----GGGEAAAVNALGESSSTL-LA--------MNL---TLSGEGYGAFHGPRGGPPELG----------------------P------------
AQP---------------IH----GFFGS---PPPG-PAAHHQTQ-P---------PVSHFG-GGF-GPEPSA-SCLHGGRLL----GY--SGG----
-----Q----------QTFAADGGYEQHLADG-Q------------------AGGDGFG------Q---------Q----------------------
RAGPLQDFQPPPPPPHHLHNPG-----VPAPCLPLD----QSPNRAASFHGLP--GA--------------------ASSEPHGLDS---
QRR-LPSQ-GAAAVDALE------------YSYPN---SEN---HFDLPVFS-SSE-A--EGQLP-
QYGSGRQAAPPGGSNFPGHSAAL--PRAPGS----LGKVP--S-------------------------------QQPPH--GVFFDRF--
GGARKMSLGLEPG--LGAGRHHLV---Q--------Q----QPP-------------------------PPALLARQNSCPPALPR-
PQQCPEGSAANANL-QDGGGPILP-AQHGQ--------FEYPIHRLENRGLQHHPHHPQQ---QQHPYGPAEP---LFNV---
AHQHQ--Q-------P----QQPP------NQRLQHFDAPPYVNLPKRPRFD-----------------SWSGGG-GAG-MHGAAALESHL-
-----------SPSAA-YP-------------GLPGDFTP--PVPEH---F----PPPPPPGS----DPQAA-----L------Q-------------------QRQN---
-----AALMIKQMAATR-----G------QSQR-LR-PPG---LQQVGHHQHPH----AAGGHGHHQSPHQ---------------------------
------HPGEAA--------------------------FEAQEG--AWFPP----PH---PP--------------------------------------A--GSGDLLFRP--

G--MG---GLQE--PPPALR-MSSGGEGHVP---SP----GGLQGQFG--------------------------LSPPSER-R--PSHPDFAAQ----
---AQSFPF-GGPSRQ-ATPHSASPGSFGPP-------------------TDFQSSAPPPS--Q-PRP---SPASSKLGALSLG--------
SFPKG-----GVGAPPG-------SAGPKESSGLFGQSCLAALSTACQNMIASLGAP--------NLNVTFGKKG---A-PAGV----
--GG-G------------------------AGGEGAKRNKLS--PAETPETNGAG-----------PQP------PLPAATVPAG-ENG--LSPNYS-
---P------------GSGPEAK------AGSGRGR---
-------------GRRKRD----------SGHVSP---AAGATGGFFEKY-------------GPTAGVE-----SGSPGQGGERS------------------
----------GGGTPHLHEPPKALSSPPSTW----AKG----GGDLLLQP---------PAEQAD-LLP--SLD----------QA---ESCSP-
RGGGGGDFPGEA---------ENEDE------VSSSSDNQ------------------------------GAKGCPAGRSPLQPP---
GRPVDHPLLN--GQKPSALAQLGLHGGGST------------ATSSP----ESYGA---------------GPPGPV--------------------------
QEEIHPLEILQAQIQLQRQQFSISEDQPLGLK------------SAKKGPEGSG----GGQNGDS-GELSSCC-----EGAGAKGA-
-------VSTIDLESLMAEHSA-AWYLPG-------DK-ALLDG----EPDEKV--LVPWEKAKAPTA-GKEAHDLPSSKAL------
-------APAQTGSHLQCLSVHCTDDMGDAKGRT-AVPTWRSLHSDISNRFGTFVAALT----------------------------------

>D.clupeoides.mn1a

---MSSYYN-SPC-LH----------VR-----A-P--S-FAAEP-DVNSISIPP--P-
QA--------FRMGA-N--GETY-EFQ-PR---RH-----LMGS---QQAS-
FGPSQD-------------LLPRAC-------QLGT-PCPQDDGRA----GY--GSGS-------------------TAHQQ--GFG-RAAEG-L-------
------------AGECFS----------------QQ---------------------RLLAMTNF--------QPSGRCNSSHPVPAPCLPLD----
QSPNRAASFHGVQ---P---------------------LSPDGRHPE----LHR-LPPQ-A--TVRPTE-----------FGFPC-DPLPG---
SFEVAGFP-VPD-S--TSDFR-YCWRGDQL---GGSGYLG-ISGV--SQAPMVFNSKAARQL--P-------------------------------
----QQ--DAYLEVI--ENRRRRSLSEELD---GSVPYSMV-----------EQ---------------------------------------QSSPGLPA-
MFQTSDY--SNSTM-RNGD-ACFH-GHQ----------VVYPAQRPDT----------------------ANP---GINR---QQ------------------
MIGP-----TSQQTHPF---GHSTLFKRPRFDV-----------GESRGLVNN---------RASMVDDHL------------SPS-I-FP----------
---GSVGEFTS--QMMDG---F-SSGPQLLAAGP----EQ-----------------------------------RCQN--------ATIMIKQMASRS-----
-------QQQR-MS-------L-PLSHHGDPT----SV-GLEFRRQVQNMPQ-P-SPEKK----------------------LSLHG------------------
----------NPSQEN--SWFLG----PH-----------------QQCRGGAEQ------------------IQ--NGHVGLFRQ----------GLTA------
VN-VLRSNKVPQEGFGHP----HDAHSSLEKG----MSN-EQMQGLGEG----TMKQISTPVGR-------------------
AHHSYFS-GLANRH-AASHHPPTGLHSSP-------------------TRF--SPPMEQ--PSGQT------LGKLGAFSLG--------SSNK-
---------GAS------------KDS-VFGQSCLAALSTACQNMIASLGAP--------NLSVTFSKKS---Q-
NEAKRKPGQVQEDINGNICSGVH--------------------GPGGEYLH--CNVPHNSQLPP----SGNSNNAATSQ------
NGTSYIGTTE-ANS--VSLDSS--M-----------NSRGEDK--APT--GSGRGR--------------
--GKRRRD----------SGHMSP--------ANFSPPC--------------SSGLGIS----
PKCPSMVNVGV-----------------------------EGQSKT---IDASLVST--AF----G-------------------------KPD-LRA--SLD--
SGIQSV-GKS---DTFSP-----RVDYL-ED---
---ASPTFCEPARTSFGGLGAVG--------------
EVHPLEILQAQIQLQRQQFSISEDPSPGGK-------------TGKKGDCQI-----PQNSD--CALANGS---S---DTGEGS--------
LNTVDLDSLMAEQHA-TWYGPG-------IK-PQLKA----PGFDKC--IAFWGSAKDQHD-NNQGHG-------------------------
----

>D.clupeoides.mn1b

---MSAHYK-SPG-FH----------AG-----GPP--P-GAVEP-GIGPMNEPP-
-M-LG--------LGMNM-N-GEYSGGGFH-PR-AHADMH------------AGGQ-----QQ-----------QAP--------------MH----
GFFNN---QQPHGHGHPHGHQ-G----HPHQ-HHPHFA-GGFGGPEPGS-SCLHGGRMM----GY--AGGM---GP---H------
----QGFAE--GFD-ALAEG-Q-------------------AGDGF------PQ---------QQ--------------------RPGSMPDF--------
QHHGPSSGNHPVPAPCLPLD----QSPNRAASFHGLPSSSS---------------------SSSDSHSLE----PRR-LPPQ-G--
GMEGLD------------YSYPT-DPPTG---HFDVSVFS-PSD-T--ESQLP-HFGAGRQV--AGG-NFPG-NPAM--SRPPGM--
QGMSKGH--A------------------------QPPSSQQQPPTQHGGGGFFERF--GNGRKMPVGMEPG--VGA-RHPLM---Q------
--QQ------------------------------QTGMLGRQNSCPPSLPR--PPQTDSGSSNPAM-QDG---MMP-GQHNQ--------
FEYPIHRLENRGM--------------HPY--GDP---MFNM---QQQ-----------APH--GPPP------NQRLQHFDA-
PYINMAKRPRFDFPGGAHHHSTT-GDGCGSWPG-----GNMHNQPGMENHL-----------SPS-T-YP------------
GLPGDFNP--QVTDS---F-PPGPPLQHPGH----EAPAT-----LQQ------------------------QRQN--------AAMMIKQMASRS--
----------QQQR-MRPQPS---LQQLGHHADVA----QG-PMVHTGPVPGMPQ-P-GFERE---------------------NGGRMV-----
--------------NFDAQNP-HMAQEN--TWFPG----PH--PPPGDMM--------GRRMGGPG--GEAGVHE---M------QP--
NGTGMMFRP--G--MNGL-GMQD-P----MR-LP--GDTHVQPLHSP-----SLHSQFGSN----MGNLGPMQSPGGG-----
VGLPNPPSDR-R----PGDFSAP---PMGAQPSFPY-GGANRQ-GPPHGNPSGVSTSP-------------------GSF--SAQSDF--
PVNQR----SSASKLGALSLG--------SFSK----------SSG-----------KDN-VFGQSCLAALSTACQNMIASLGAP--------

NLNVTFNKKS---Q-SDGKRKLSQTEQDVN--A-GAA-N------------------GNNGNEFFPGGGGGSQGGQIPG----T----
AKPAGQ---------NQPVQGE-ASA--LSPNYS--------------------------------------M---------DA-TPCSEGK--AAT--
GGGRGR---GRRKRD----------SGHVSP--------GVFFSSD---------
-----NSNPVVS-P-GQQAPPSAGLGE------------R----------------GGGTPH---E-KPLRSP--SW----GKG--G---DLML---------
---GDQAD-LMS--SLD--SGIQSV-SKS---DGCSP-----HVDFP-EDVSGP--HY-GNEDE------VSSSSDAAV------------------
---------SNKAGRSPLVGGSPKLAR-------LEGPPG--ALKGQAMGVANH------------------TTSTS----DGYGL------
GSGGAAHP-GTPVLE-QVRTP---TSSSG------------QDEVHPLEILQAQIQLQRQQFSISEDQPLAMK-------
NGGSAGKKGGADCAV------QNGD--VDLAGCS---P---DAGKAA--------MGTIDLDTLMAEQHA-TWYVPG-------
DK-SLMEG----TEEDKA--LATWEKTKSQTS-LKEEVDLSQAKGG-------
GVVGAPGSGAGGAHLQCLSVHCTDELGDNKGRGGPVSSWRSLHSDISNRFGTFVAALT-------------------------------
----

>C.lupus.familiaris

-------MFGL-DQFEPQ--------INSRN--------AGQGER------NF-NEAGLS----MNAHFK-APA-FH----------AG-----GPP----
GPVDP-AMSALGEPP--I-LS--------MN-------MEPY-GFH-AR-GHSELH-------------AGGL------------------QAQP----------
-----VH----GFFGG---QQPH-HGHPGGHH--------PHQHHPHFG-GNFGGPDPGA-SCLHGGRLL----GY--
GGAAGGLGS---Q----------PPFAE--GYD-HMAES-Q-------------------GPESFGPQ-------------------------------------
RPGNLPDF----------HSSGASGHAVPAPCLPLD----QSPNRAASFHGLP--AP---------------------SGSDSHSLE----PRR-
VANQ-G--AVDSLE------------YNYPG-EAPSG---HFDMFS---PSD-S--EGQLP-HYAAGRQV--PGG-SFPG-ASAM--
PRAAGM--VGLSKMH--A-----------QQQQQQQQQQQQQQQQQPQQQQQQQQH--GVFFERF--GGARKMPVGLEPG--
VGS-RHPLM------------QPPQQAPPPPPQQPPQQP--PQPQQQQQPPAPPPPGLLVRQNSCPPALPR--
PQQGEAGTPSGGL-QDGG-PMLP-SQHAQ--------FEYPIHRLENRSM--------------HPY--SEP---VFNM---QHPP---------
-------PQQA-----PNQRLQHFDAPPYMNVAKRPRFDFPGS------AGVDRCASWNG-------NMHNGALDNHL------------
SPS-A-YS-------------GLPGEFTP--PVPDS---F-PSGPPLQHPAP----DHQSLQQQQQQQQQQQQQQQQQQQQQQQQQ--
------QRQN--------AALMIKQMASRN------------QQQR-LR-QPN---LAQLGHPGDVG----QG-GLVHSGPVGGLAQ-P-
NFERE------------------GAGAGAGRLG-------------------AFEPQAP-HLAQES--AWFPG----PHPPPPPGDLL--------
PRRMGGSGLPADCGPHD---PGLAPPPPP--GGSGVLFRG----------PLQE------PLRMP--GEGHVPALPSP-----GL--
QFGGS----LAGLGQLQSPGAG-----VGLPSAPSER-R--PPPPDFTAP--AALGGQPGFPF-GAATRQ-ATPHSG-
PGVNSPPSAGGG----AGGGGGGGGGGAY--PPQPDF--QPSQR----ASASKLGALSLG--------SFNK----------PSS--------
----KDN-LFGQSCLAALSTACQNMIASLGAP--------NLNVTFNKKN---P-PEGKRKLSQNEADGAAAAG---------------
---------NPGSDYFP--GGAAPGAPGPGGPPGTGSGGSKASGP--------PNPPAQGD-GTS--LSPNYT-------------------------
----------------------------LESTSGNDGKPVPGG---GGRGR---
GRRKRD----------SGHVSP--------GTFFDKY--------PAAPDSGGAPGVSPGQQQPAQGAAVGGS-----SASAGEAR-----
-----------GAPTPH---E-KALTSP--SW----GKG-----AELL------------LGDQPD-LMA--SLD--GG-----AKS---DGSSP----
HVGEFASDE-VST--SY-ANEDE------VSSSSDNPPA-------------------------LAKASRSPLVTGSPKLPP-
RGVGAGEHGPKAPPPPPPLGLGIMST------------------STSTP----DSY--------GGGGAGHP-GTPGLE-
QVRTPTSSSGAPP------------PDEIHPLEILQAQIQLQRQQFSISEDQPLGLK-------------GGKKGECAV-
GSSGAQNGD--SELGSCC---S---EAVKSA--------MSTIDLDSLMAEHSA-TWYMPA-------DK-ALVDG----ADDDKT--
LAPWEKAKPQNPNSKEAHDLPANKAS--------------ATQPGSHLQCLSVHCTDDVGDAKARA-
SVPTWRSLHSDISNRFGTFVAALT-----------------------------------

>F.catus

-------MFGL-DQFEPQ--------INSRN--------AGQGER------NF-NEAGLS----MNAHFK-APA-FH----------AG-----GPP----
GPVDP-AMSALGEPP--I-LS--------MN-------MEPY-GFH-AR-GHSELH-------------AGGL------------------QAQP----------
-----VH----GFFGG---QQPH-HGHPGGHH--------PHQHHPHFG-GNFGGPDPGA-SCLHGGRLL----GY--
GGTAAGLGS---Q----------PPFAE--GYD-HMAES-Q-------------------GPESFGPQ-------------------------------------
RPGNLPDF----------HSSGASGHAVPAPCLPLD----QSPNRAASFHGLP--AS---------------------SGSDSHSLE----PRR-
VANQ-G--AVDSLE------------YNYPG-EAPSG---HFDMFS---PSD-S--EGQMP-HYAAGRQV--PGG-SFPG-ASAM--
PRAAGM--VGLSKMH--AQQQQQQQQQQQQQQQQQQQQQQQQQQQQQQQQQQQQH--GVFFERF--
GGARKMPVGLEPG--VGS-RHPLM------------QPPQQAPPPPQQQPPQQP--QQP-
PPPPPQQPPPPGLLVRQNSCPPALPR--PQQGEAGTPSGGL-QDGG-PMLP-SQHAQ--------FEYPIHRLENRSM--------
------HPY--SEP---VFNM---QHPP----------------PQQA-----PNQRLQHFDAPPYMNVAKRPRFDFPGS------
AGVDRCASWNG-------SMPNGALDNHL------------SPSAA-YS-------------GLPGEFTP--PVPDS---F-PSGPPLQHPAP--
--DHQSLQQQQQQQQQQQQQQQQ----------------QRQN--------AALMIKQMASRN------------QQQR-LR-QPN---
LAQLGHPGDVG----QG-GLVHSGPVGGLAQ-P-NFERE--------------------SAGAGRLG-------------------TFEQQAP-
HLAQES--AWFPG----PH--PPPGDLL--------PRRLGGSGLPADCGPHD---PGLAPPPPP--GGSGVLFRG----------PLQE-

-----PLRMP--GEGHVPALPSP-----GL--QFGGS----LAGLGQLQSPGAG-----VGLPSAPSER-R--PPPPDFTAP---
ALGGQPGFPF-SAANRQ-ATPHSG-PGVNSPPSAGGG----GGGTGGGSGGGAY--PPQPDF--QPSQR----
TSASKLGALSLG--------SFNK----------PSS------------KDN-LFGQSCLAALSTACQNMIASLGAP--------
NLNVTFNKKN---P-PEGKRKLSQNETDGAAVAG-----------------------NPGSDYFP--
GGTTPGAPGPGGPSGTSNSGSKASGP--------PNPPAQGD-GTS--LSPNYT--
LESTSGNDGKPVPGG---GGRGR---GRRKRD----------
SGHVSP--------GTFFDKY--------SAAPDSGGAPGVS-P-GQQQAPGAAVGG------SSTSEAR----------------GAPTPH---
E-KALTSP--SW----GKG-----AELL------------LGDQSD-LMA--SLD--GG-----AKS---DGSSP----HVGEFASDE-VST--
SY-ANEDE------VSSSSDNPPA--------------------------LAKASRSPLVTGSPKLPP-RGVGAGEHGPKA--
PPPPLGLGIMST------------------STSTP----DSYGG------GA---GHP-GTPGLE-QVRTPTSSSGAPP------------
PDEIHPLEILQAQIQLQRQQFSISEDQPLGLK-------------GGKKGECAV-GSSGAQNGD--SELGSCC---S---EAVKSA-
-------MSTIDLDSLMAEHSA-TWYMPA-------DK-ALADG----ADDDKT--LAPWEKAKPQNPNSKEAHDLPANKAS--
------------ATQPGNHLQCLSVHCTDDVGDTKARA-SVPTWRSLHSDISNRFGTFVAALT-----------------------------------

>A.calliptera

-------MFGL-EQFGSQ--------INSRN--------PGQSER------NI-NQQRL----NMGSHYK-GPG-FH----------AG-----G-P--P-
GAVEP-GMGPLSEPQ--M-LG--------LNMNM-N--GEQYGSFH-PR-GHSDMH-------------AGSG-LQQQQG------------
QGP---------------MH----GFFNN---QQPH-QGHPHGHQ-P----HPHQ-HHPHFG-GNFGGPEPGS-SCLHGGRLM----
GYNNNNGM---GP---Q----------QGFGE--GFD-PLAEG-Q-------------------TGDGFPQQQQQQP---------QQ----------------
-----RPGNMPDF--------QHHGPPSGNHAVPAPCLPLD----QSPNRAASFHGLPSSSS---------------------SSSESHGLE----
PRR-LPNQ-G--AVEGLE------------YNFPS-EPPSG---HFDVPVFS-PSE-S--ESQLP-HFGPGRPV--PGG-NFPG-NAGM--
PRTPGM--PGISKGH--Q------------------------PPP---QPQQPQH--GVFFERF--GNGRKVPVGMEPG--VNP-RHPLI--QQ-
-------QQ-------------------------------QAGLIARQNSCPPGLPR--PPQAEPGTTNPNI-LDGG-VMMP-GQHNQ--------
FEYPIHRLENRGL--------------HPY--GDP---MFNM---QQPA-----------PPPS-QQPA------NQRLQHFDS-
PYMNMAKRPRFDFPNA-H----G-GEG---WCG------------GMDNHL------------SPS-A-YP-------------GLPGEFTP--
PVNEG---F-GPGP-LQHPGP----EQQSL-------Q------------------------QRQN--------AAMMIKQMASRN------------QQQR-
MR-QPS---LQQLGHHGDVP----PG-PMVHGGPVGSMPH-P-GFDRE----------------------NSGRMP-------------------
NIDGQNP-HVTQEN--SWFQG----SH---PPGEMM--------SRRMGGAG--NESGPHD---MGL----QQ--NGAGIMFRP-----
--GI-GMQE-P----MR-IP--GDGHVQNLHSP-----GMHSQFSGN----MGNLTQMQSPGAG-----AGHPNAPAER-R----
PADFPAP---SMGAQPAFPY-GGANRQ-GPAHSAPQGVSTSP-------------------GNY--PPQSEF--PSGQR----
SSVSKLGNLSLG--------NFSK----------TSS------------KDS-VFGQSCLAALSTACQNMIASLGAP--------
NLNVTFNKKN---Q-NEGKRKLSQTEQDIN--S-STS-NG----------------TGSAGPEYFQ--SSTSQNSQMPG----
TGNSNSNSKPASQ---------SQTVQGE-ASA--LSPNYN---------------------------------------M---------DA-TPCSEGK--
ATT--GSGRGR---GRRKRD----------SGHVSP--------GIFFSSD---
-----------NSNPVVS-P-GQQTPSAGVGER-----------------------------GGGTPH---E-KQLQSP--SW----GKG-----GDLM-----
-------LGDQAD-LMS--SLD--SGIQSV-AKS---DSSSP-----RVDFS-ED-VST--HY-GNEDE------VSSSSDAGGAS---------
----------------ASKPNRSPIINGSPKMQR------SDHGLIN--GQKPLGMGINNH------------------TTSTP----
DTYGLNAGVGTGASGVSHP-GTPGVE-QVRTP---SSTSG------------QEEIHPLEILQAQIQLQRQQFSISEDQPLAMK-
-----------TGKKNGDCPS------QNGD--NELASCS---P---DAGKGS--------MGTIDLDTLMAEQHA-TWYVPS-------DK-
VMMDG----SEDDKA--TGPWEKNKSQNS-SKEESELTQSKPG-----
AGAPGAVGGGSSGGTHLQCLSVHCTDELGDSKGRGGPVSSWRSLHSDISNRFGTFVAALT---------------------------
----

>T.aculeatus

-------MFGLGDQFEPP--------LPGRS--------AGPPHR-------HLHESGL----GMNGHFK-APA-FQ---------------------------
DPAALGALGDGP--L-LG--------MN---LGMNGEPY-GYHPPR-GPADLH-------------AGAL----------------------------------
---QPVP--GFFGG---QQPL-PGHP-------------------HFG-AGFGGPDPGA-SCLHGGRLL----GYGAGGGQ-------------------
PAFAE--GYD-PLADH-P-------------------GGEGFGQQPQQQQQ------------------------------RPGNLPDF-------
QQPHGSGGSGPAVPAPCLPLD----QSPNRAASFHGLP--AS---------------------GSSDPHGLE----PRRVANPG----
GVDSLE------------YNYPG-DGPAG---HFDLPVYS-PSE-A--DGQLP-HYGAARQM--PAG-TFPG-TNAL--PRAPGM--
GGMAKAQ-----------------------------------QQQQH--GVFFERF--GAARKMSVGMETG--VNA-
RHPLMQQQQAPQQQQQQQQQQQAPQQQAPQQQQAPQQQQQ----------PPGMLPRQNSCPPAIPR--
PPPPDAGAPNPSLHQDPG-PLLQ-NQHAQ--------FEYPIHRLENRNM--------------HPY--GDP---VFNLQPPAPPPPQ---
-----------QQQP-----PNQRLQHYDA-PYLNVAKRPRFDFANN------PAVDRCPSWSG-----------AGLDSHL------------
SPS-A-YP-------------GLPGDFTP--PGPEG---F-GPGPPLQHPAQ----DPQALQHQQHQQQQHQHQQQ-----------------
QRQN--------AALMMKQMASRT------------QQR-LR-PPT---LPQLGHPGDVG----P----VHGGPPAGLPQ-P-PFERD----

------------GPGPGGVGGRLP-------------------GFEAQAPHHLGPDS--AWFPGPP--PP---PPGDLL--------
PRRLGAPPLPADGGPHE---LGL-----PPGAGSGLLFRGPG---GAGGLGLQE--------PLRLAGEGHGPGLPSP---A-
GLHAQFGGG-------LAQLQSPGAG-----VGMPGAPSDRRP--PPPPDFAGP---GLGGQPGFPF-GPGSRQ-NTPHGT-
PGLSASPGGGTAGGTGPGGGGGAAAYPPPPPPPPPPPPDFPSSQR---AAAASKLGALSLG--------SFSK----------PSS---
---------KDN-LFGQSCLAALSTACQNMIASLGAP--------NLNVTFNKKS---Q-
AEGKRKLSPAEPDGAGGGGAGAGP-------------------AGGPDYFPVGKAPGPAAHASG----------------------------GE-
GTG--LSPGYS--LEPSPGHDGKPGPGG-GSAGRGR-----------------------------
--------------------------------------GRRKRD----------SGHVSP--------GAFFDKF----------------
GPGAGGPAADGGPGLSPGRQ-------GGAEPR----------------GAPTPH---DAKAPTPP--AW----AKA-----GDLV----------
--LGEPPADLCPRWTAA--CGRPPSRAGPPRGPVTSP-----TRPAT-DTRTRCRPAPTAPRDP-------------------------------
----------RPGPAAAPRTPAPPRSRG-GPGPPGDPGLHN--GQKPLGLGLLAAAAAAAAATAN--------STSTP----DGY---
----------GPP-GTPGLEGQGRTPGPGGGGGG------NGSTPPQDEIHPLEILQAQIQLQRQQFSISEDQPVGMK------
AGGAGKTAAGAADCSA-----GQNGD--SELNGCC---S---EAVKSA--------MSTIDLDSLMAEHGS-AWYLPA-------
DK-ALMDG----PDDDKA--LAPWDKAKPQTP-SKEAHDLPQNKTS-------------
ASAQPGSHLQCLSVHCTDDVGEAKGRT-AVPTWRSLHSDISNRFGTFVAALT----------------------------------

>H.capensis

-------MFAL-EQFEPP------GSSGSRS--------GAPGER---------GGGGGGFAQGMSSHFK-PSP-FH----------HG-----GTP---
--GAEP-ALSALGHESPSAMLA--------MN--L-SLPGDAY-SFHARGGGPSELQ-------------QAQP--------------------------
----------AH----GFFGN---QQPSHSAHQ---------------PPSHFG-GGF-GPEPST-SCLHGGRLLNYPGGQ---------------------
---QAFTAD-GYEQHLADSQD------------GGGVSSGGGGGFGQQ-----------------------------------RAGPLQDF------
QPPPPQQQPHNPGVPAPCLPLD----QSPNRAASFHGLP--GA---------------------SSAESHGLD----SQRRLPSQ-
GAAALEPLE------------YSYPS---SEA---HFDLPVFS-PSE-A--DGQLP-HYGAGRHQVAAGGGSFPGPSSAL--
ARAPGGGMGPMAKVH--P------------------------HQQQQQQQQQPPH--GVFFERF--GGARKMPVSLEPG--LSA-
RHPLMQHQP--PPALLGRQNSCPSALPRPPPPCPEGSAANPGL-QDGGAPLLQ-
GQHAQ--------FEYPIHRLENRTL-------------QHPYGPGEP---VFSVHHHPPQ-----------------PPPP-----
HNQRLQHFDAPPYMNAAKRPRFDSWSG----------------------GGGMHGASLESHL------------SPS-S-YT-------------
GLPGDFTP--PGPDS---F-PAGPPLQPPAGADHQAALQQ--------------------------------QRQN--------AALMIKQMAASR-
------GQQQQQQQQRLR-QPS---LQQLGHH--------------------------HQQQH---
HHPHGEPAFEAQEG--AWFPA----PP-------------------------HPAAAGPGD------------------LLFRP--G--
VGGMPGLQEPP----LR-MPSGGEGHVP---SPG----SLHGQFGLS---------------------------PPSER-R---PGPDFAAQ--------
QGFPFGGASSRQ-ATPHSASPGSYGPS-------------------TDFQASGPPPA--PQPRQ---PPASSKLGALSLG--------
SFPKGGGAGPGVGAPPG----GGGGAGPKESSGLFGQSCLAALSTACQNMIASLGAP--------NLNVTFGKKG---A-
VAAAAAAGAGGGVGA----------------------------GEGGKRSK---LSPAEPPEPG----SAGPPPLPPPP---------PPSAESG---
---LSPNYS---P------------GPGADVK------VGGGRGR--
------------------------------GRRKRD----------SGHVSP----AGGGGGYFDKF-------GPTGGAEGGSPGQG---
GERAGPGGGTTP----------TTP----------------HLHEPH---N-KALSSPPSAWTKGGGGGGGSSAAAELL-LPPP-------PP-
EPPELLP--PLD----------KA---DSCSP----RSGDFP-DE------A--GNEDE------VSSSSDNQL---------------------------
PKGPGRSPLQPRPGD-----------PSLLNGQKAALAQLGLHGGN-----------------ATSSP----DSYG----------------GAPPTS-
-------TTSAPV------------QEEIHPLEILQAQIQLQRQQFSISEDQPLGLK----------
SSKKGPDGGGGGGGGSAGLQNGDS-GDLSSCC----GGGESNKGA--------MSTIDLDSLMAEHSA-SWYLPG-------
DK-GLLEGPDEQEDDKAA--LAPWEKAKPANP-SKEAHDLPPSKTS-------------
AAAQTGSHLQCLSVHCTDDMGEAKGRT-AVPTWRSLHSDISNRFGTFVAALT----------------------------------

>P.senegalus

-------MFGL-EQFEPQ--------IGSRN--------VAQGER------SF-SQPGL----NMSAHYK-SPA-FH----------PA-----G-P--
SAAAVEA-GMGGLNEPP--M-LG--------LGMNL-N--GDQY-SFH-AR-GHSDMH-------------TGGL------
QQPPPPQQQQQPPPPP---------------MH----GFFNG---QQPH-HGHQHGHH-P----HAHQ-HHPHFG-
GSFNGADSGGTSCLHGGRLM----GY-NNGGL---GP---Q----------QNFAE--GFE-PMADN-S-----------------G-
AGEGFG----------------QP---------------------RPNNMPEF--------QHHNPQGSGHSVPAPCLPLD----QSPNRAASFHGLP-
-SS---------------------SSSEAHGLE----QRR-MPAQ-A--GVDSLE------------YSYPA-DNPSG---HFDMPVFS-PSD-S--
DTQLP-HYGASRQV--P-S-NFSG-SPVM--PRAPGM--AGISKVH---------------------------------TP---QH--GMFFERY--
GNGRKMSVGMEPG--VNS-RHPLM--QQ-----PPPPQ-------------------------------QASLLARQNSCPPAIPR--
QSQVEPAAANPNL-QENG-VIMP-GQHNQ--------FEYPIHRLENRTM--------------HPY--SDP---MFNM-QQQQQP-----
------PPPSSQQPP------NQRLQHFDG-PYLNMAKRPRFDFPSN-H----N-ADNCSTWN------N-MHNPAGMENHL--------
----SPS-A-YS-------------GLPGEFTP--PVPES---F-SQGPPLQHTGS----EQQSL-------Q-----------------------QRQN--------

AAMMIKQMASRN------------QQQR-MR-QPS---LQQLGHHGDVT----QS-SMVHGGQVGSLPQ-P-NFDRE------------
---------GGGRMA-------------------AFDSQNP-HMPPEN--TWFPG----PH---PPGEML--------PRRMGSSGVPGEASPHE--
-MGI----QQ--NGSNMLFRS--A--VNGM-GMQE-P----MR-MP--GDGHVQGLHSP-----GMHSQFGPN----
MGGLSQMQSPGTG-----VGLPGAGSDR-R----PSDFPAP---PTG----FSF-GGANRPAAAPHSNPSGVSASP--------------
----GNY--PPQTDF--QPGQR----PSVSKLGALSLG--------SFSK----------TTT------------KDN-
VFGQSCLAALSTACQNMIASLGAP--------NLNVTFNKKS---Q-NEGKRKLSQTEPDGS--GASAPGGA---------------
NGGTGPEYFQ--PSMPPNSQMAG----TG-GGAKPAGP------GGPSQPAPGE--PN--LSPNYT--------------------------------
----------I---------DA-ATGNDGK--PPT--GSGRGR---GRRKRD---
-------SGHVSP--------GIFFDKF-----------STDSGNPGVS-P-GQQGPSASVGER----------------------------GRSTPH---D-
KPLTSP--SW----GKG-----SDLL------------MGDQQD-LMS--SLD--SGIQSV-TKS---DTSSP-----HVDFP-DD-VST--HY-
GNEDE------VSSSSDNV----------------------------TSKPSRSPLVTGSPKMQR------GDHGLIG--GQKPMGLGMLNN-----
-------------STSTA----DSYGL---SSTGA---GHP-GTPGME-QVRTP---SSTST------------
QDEIHPLEILQAQIQLQRQQFSISEDQPLAMK--------------NKKAECPA------QNGD--NELGSCG---T---DGGKNA-----
---MSTIDLDSLMAEQHA-TWYIPN-------DK-ALMEG----QEEEKA--MAPWEKTKPPNN-SKEVPEHQNKTPA--------
------AGQNGSHLQCLSVHCTDDIGEAKART-PVPTWRSLHSDISNRFGTFVAALT----------------------------------

>H.grypus

-------MFGL-DQFEPQ--------INSRN--------AGQGER------NF-NEAGLS----MNAHFK-APA-FH----------AG-----GPP----
GPVDP-AMSALGEPP--I-LS--------MN-------MEPY-GFH-AR-GHSELH-------------AGGL------------------QAQP----------
-----VH----GFFGG---QQPH-HGHPGGHH--------PHQHHPHFG-GNFGGPDPGA-SCLHGGRLL----GY--
GGAAGGLGS---Q----------PPFAE--GYD-HMAES-Q-------------------GPESFGPQ-------------------------------------
RPGNLSDF----------HSSGASGHAVPAPCLPLD----QSPNRAASFHGLP--AS---------------------SGSDSHSLE----PRR-
VANQ-G--AVDSLE------------YNYPG-EAPSG---HFDMFS---PSD-S--EGQLP-HYAAGRQV--PGG-SFPG-ASAM--
PRAAGM--VGLSKMH--A----------------QQPPPPPPPQQPQPQPQPQQH--GVFFERF--GGARKMPVGLEPA--VGS-
RHPLM------------QPPQQAPPPPPQQPPQPPQQPQPQPQPQPPPPPPPGLLVRQNSCPPALPR--PQQGEAGTPSGGL-
QDGG-PLLP-SQHAQ--------FEYPIHRLENRSM--------------HPY--PEP---VFNM---QHPP----------------PQQA-----
PNQRLQHFDAPPYMNVAKRPRFDFPGS------AGVDRCASWNG-------SMHNGALDNHL------------SPS-A-YS--------
-----GLPGEFTP--PVPDS---F-PSGPALQHPAP----DHQSLQPQQQHQQPQQQ--------------------QRQN--------
AALMIKQMASRN------------QQQR-LR-QPS---LAQLGHPGDVG----QG-GLVHSGPVGGLAQ-P-NFERE---------------
-----SAGAGRLG-------------------TFEQPAP-HLAQES--AWFPG----PH--PPPGDLL--------PRRMGGSGLPADCGPHD---
PGLAPPPPP--GGSGVLFRG----------PLQE------PLRMP--GEGHVPALPSP-----GL--QFGGS----LAGLGQLQSPGAG---
--VGLPSAPSER-R--PPPPDFTAP---ALGGQPGFPF-SAANRQ-ATPHSG-
PGVNSPPSAGGGGGGSTGGGGGGGGGGGAY--PPQPDF--QPSQR----ASASKLGALSLG--------SFNK----------PSS----
---------KDN-LFGQSCLAALSTACQNMIASLGAP--------NLNVTFNKKN---P-PEGKRKLSQNETDGAAVAG------------
------------NPGSDYFP--GGTAPGAPGPAGPSGTSNSGSKASGP--------PNAPAQGD-GTS--LSPNYT----------------------
-------------------------------LESTSGNDGKPVPGG---GGRGR---
GRRKRD----------SGHVSP--------GTFFDKY--------SAAPDSGGAPGVS-P-GQQQAPGAAVGA------SSTSEAR---------
-------GAPTPH---D-KALTSP--SW----GKG-----AELL------------LGDQPD-LMA--SLD--GG-----AKS---DGSSP----
HVGEFASDE-VST--SY-ANEDE------VSSSSDNPPA--------------------------LAKASRSPLVTGSPKLPP-
RGVGAGEHGPKA--PPPPLGLGIMST------------------STSTP----DSY--------GGGGTGHP-GTPGLE-
QVRTPTSSSGAPP------------PDEIHPLEILQAQIQLQRQQFSISEDQPLGLK-------------GGKKGECAV-
GSSGTQNGD--SELGSCC---S---EAVKSA--------MSTIDLDSLMAEHSA-TWYMPA-------DK-ALVDG----ADDDKT--
LAPWEKAKPQNPNSKEAHDLPANKAS--------------ATQPGSHLQCLSVHCTDDVGDAKARA-
SVPTWRSLHSDISNRFGTFVAALT----------------------------------

>C.mydas

-------MFGL-EQFEPQ--------ISSRN--------AGQGER------NF-SQAGLT----MGSHFK-SPA-FH----------SG-----G-P------
ADP-AISALGEPP--L-LG--------MNM---NMAGEAYG-FH-AR-GHSELH-------------AGGM------Q------------AQP--------
-------VH----GFFGN---QQPH-HGHPSTHH-P------HQ-HHPHFS-GNF-GSDPSA-SCLHGGRLM----SY--SSSM---GS---
Q----------QAFAE--GYE-HMAEN-Q-------------------GGEGFG------Q---------Q----------------------RSGNMPDF--------
QHHNSSASNHAVPAPCLPLD----QSPNRAASFHGLP--AS---------------------SSSDSHSLE----QRR-LPNP-G--
GVDSLE------------YNYAS-DGPSG---HFDLPVFS-PSE-S--EGQLP-HYGAGRQV--PGGGSFPG-TSVL--PRAPGL--
AGMSKVH--P-----------------------Q----------QQH--GVFFERF--GGARKMSVGMEPG--VNA-RHPLM--HQ--------QP---
----------------------------QTGLLARQNSCPPAIPR--QQQADANAPNPGL-QDNG-PVLQ-NQHAQ--------
FEYPIHRLENRNM--------------HPY--TDP---VFNM----QPP---P-------P----QQPP------NQRLQHFDA-
PYMSVAKRPRFDFPSS-P----A-VDRCAAWSN-----P-LH-GAGMENHL-----------SPS-A-YP------------GLAGEFTP--

PAPES---F-APGPPLQHPGP----DPQAL-----Q-------------------------QRQN--------AALMIKQMASRS------------QQQR-
LG-PPS---LQQLGHHGDLG----QSGLG-HAGPVGTMPQ-P-GFERE---------------------SGGRGP-------------------
GFEPPAP-HLAQDS--GWFPGP---PP---PGGELL--PPPPPPPRRLGG---PAEPGPHE---LGL----AP--GGSGLLFRP--A--
AGAL-GL------------A--GEGRGPALHSP-----GVHAPFG-------AGLAPLPSPGAGGG---VGLPSAPAER-R---
PPADFAAP---PLGGPAGFGF-GASGRP-APPPSA----SGSP-------------------GAF-P--PPEF--PPAPR---
AAAASKLGALSLG--------SFAK----------PA-------------KEA-AFGQSCLAALSTACQNMIASLGAP--------
NLHVTFAKRG---P-PEARRKPGPPEPDGGA-----------------------APGPAPGPDFFP--A-----------------------KA----------
AP-----EAS--LSPGFP---P---------EA-APGGEGK---AAAAAGGRGR---------------------
--GRRKRD----------SGHVSP--------GGFFDKF---------------------
PPAEGGGAASPGPG----------------------------PGAPPA--AHDRPLASP--SW----AKG-----GERP------------RGEQPD-
LLA--SLD--SGLPSA-SQS---DGGSP-----RGDFP-AE-PSP--AY-GHEDE------VSSSSDGGP--------------------------
AKPPTRSPLLGGSPP-----------QALLG--GQKPLALGLLGG------------------PGPAP----DSYGLG-----GGGGGGHP-
GTPGLE-QVRTP---GSSSA------------QDEIHPLEILQAQIQLQRQQFSISEDQPLGMK--------------SKKAECPS------
QNGE--GELNSCC---S---DTGKGA--------MSTIDLDSLMAEHNS-TWYMPS-------EK-SLMEG----QEEDKP--
MAPWEKSKPQNP-SKEAHDLPQNKTS-------------AAAQTGSHLQCLSVHCTDDMGESKGRT-
AVPTWRSLHSDISNRFGTFVAALT----------------------------------

>P.reticulata

-------MFGL-EQFGSQ--------INSRN--------PGQSER------NI-NQQRL----NMGSHYK-SPG-FH----------AG-----G-P--P-
GAVES-GMGPMNESQ--M-LG--------LNMNM-N--GEPYAGFH-PR-GHPDMH-------------TGGG-LQQQQQ------------
QGP---------------MH----GFFNN---QQPH-QGHPHAHQ-P----HPHQ-HHPHFG-GNFGGPDPGS-SCLHGGRLM----GY-
NSNGM---GP---Q----------QGFGE--GFD-PLAEG-Q-------------------AGEGFPQQQPQ-----------QQ---------------------
RSANIPEF--------QHHGPPSGNHPVPAPCLPLD----QSPNRAASFHGLPSSSS---------------------TSSEPHTME----PRR-
MPNQ-G--AVEGLE------------YNFPS-DPPSG---HFDVPVFS-PSE-T--ESQLP-HFGPGRPV--PSG-NFPG-SSGM--
PRTPGM--QGISKGH--Q------------------------PPP---QPQQPQH--GVFFERF--GNGRKMPVGMEPG--VNA-RHPLM--
QP--------QQ-------------------------------QTGLIARQNSCPPGLPR--PPQAEPGNTNPNI-LDGG-VMIP-GQHNQ--------
FEYPIHRLENRGL--------------HPY--GDP---MFNM---QQPA-----------PPPS-QQPP------NQRLQHFDS-
PYMNMAKRPRFDFPNS-H----G-GDG---WCS------------SMDNHL------------SPS-A-YP-------------GLPGEFTP--
PVNEG---F-APGP-LQHPGP----EPQSL-------Q-----------------------QRQN--------AAMMIKQMASRN------------QQQR-
MR-QPS---LQQLGHHGDVP----PV-PMTHGGPVGNMPQ-P-NFDRE---------------------NSGRMP-------------------
NIDGQNP-HVTQEN--SWFQG----SH---PPGEIM--------SRRMGGAG--NESGPNE---MGL----QQ--NGAGMMFRP------
-GI-GMQE-P----MR-IA--GDGHVQTLHSP-----GMHSQFSGN----MGNLSQMQSPGAG-----GGHPNAPAER-R----
PADFPAP---PMGAQPTFPY-GGANRQ-GPPHSAPQGVSTSP-------------------GSY--PPQSEF--PSGQR----
SSVSKLGALSLG--------NFSK----------TSS------------KDS-VFGQSCLAALSTACQNMIASLGAP--------
NLNVTFNKKN---Q-NEGKRKLSQTEQDIN--S-STS-NG----------------TGSAGPEYFQ--SSASQNSQMPG----
TGNSNSKPVSQ---------SQTVQGE-ASA--LSPNYN---M---------DA-TPCSEGK--
ATT--GSGRGR---GRRKRD----------SGHVSP--------GIFFSSD---
-----------NGNPVVS-P-GQQTPSAGVGER-----------------------------GGGTPH---E-KHLQSP--SW----GKG-----GDLM----
--------LGDQAD-LMS--SLD--SGIQSVAAKS---DSSSP-----RVDFP-DD-VST--HY-GNEDE------VSSSSDAGGAS-------
------------------ATKPNRSPMITGSPKMQR------SDNSLLN--GQKPLGMGINNH------------------TTSAP----
DSYGLNSGVSTGASGVSHP-GTPGVE-QVRTP---SSTSG------------QEEIHPLEILQAQIQLQRQQFSISEDQPLAMK--
----------NGKKSSDCPS------QNGD--NELPGCS---P---DAGKGS--------MGTIDLDTLMAEQHA-TWYVPS-------DK-
TMMDG----SEDDKA--MGPWEKNKSQNS-SKEESELSQSKTG---
AVAPGAGGGGGGSSGGNHLQCLSVHCTDELGDSKGRGGPVSSWRSIHSDISNRFGTFVAALT------------------------
----

>H.harpyja

-------MFGL-EQFEPQ--------SSSRS--------GGQAER------GF-GQPGLS----MSAHFK-APA-FP----------GG-----G-P-AA-
AAVDP-ALGALGEPP--L-LG--------MNM---SLAGDAYG-FP-GR-GPAELH-------------GGGM------Q------------P-P-----
----------VH----GFFGG---QQPH-GGHGGAHH-P------HQ-HPPHFG-GNF-GPDPGA-SCVHGGRLL----GY--SGAL---
GG---Q----------TAFAD--GYE-HMAES-Q-------------------GGEGFG------Q---------Q---------------------RPGNLPDF-------
-QHHSAGASSHAVPAPCLPLD----QSPSRAASFHGLP--AA---------------------GSSEPHGLE----PRR-LPAQ-G--
GVDSLE------------YNYPG-DGPAG---HFELPVFS-PSE-P--EGQLP-HYGGGRQV--PAGSGFAG-APAL--ARAPGM--
A-VAKAH--P-----------------------P----------QQH--GVFFERF--GGARKMSASLEPG--ASA-RHPLM--QQ--------QQ---
PPPPQPPQ-------------------QPPGLLARQNSCPPAIPR--QQQTEANAPNPNL-QDNG-PIMQ-NQHAQ--------
FEYPIHRLENRNM-------------HPY--TDP---VFNM----QHP---P-------P----QQPP------NQRLQHFDA-

PYVSVAKRPRFDFPGN-P----G-VERCASWGS-----G-MH-GPTMESHL------------SPT-A-YP------------GLPGEFTP--
PAPEA---F---GGPLPHSGP----EHPAL-----A-------------------------QRQN--------AALVMKQMASRS------------QQR-
LR-PPS---LQQLGHHGEVG----PP---------GGLPP-P-AFERE---------------------AGGSSR------------------GFDPPAP-
HLAPDS--AWFAG----PP---PPGELL--------PRRMAAPGLPAEAAPHE---LGL----QP--GGAAVLFRP--G--TSGL-
GLQE-P----LR-MA--GEGPAQALPSP-----GVHPPFAPA----MGGLSQLQSPGGG-----VALPSAPAER-R---
GPTDFAAQ---P-----GFPF-AAAARQ-PVAHGATPALSASP------------------GAY-PPPPPEF-PPPPPP---
RPAASKLGALSLG--------SFSK----------PAS------------KDN-VFGQSCLAALSTACQNMIASLGAP--------
NLNVTFNKKS---P-AEAKRKLSQAEPDP--------------------------PPPAAPDYFP--AGPPAGGGGTG----------KAAGT---
-------APLLPA-ESS--LSPGYT---L---------EP-AAGGEGK------AGGGRGR--------------
---GRRKRD----------SGHVSP--------GTFFEKF----------SATEGGGAGVS-P-
GQPAVPVVAGGP-----PGAVGAER----------------GGGTPH---D-KPLTSP--SW----GKG-----GELL------------LGEQPD-
LMS--SLD--SGIQSV-TKS---DGSSP-----HVDFP-DE-VST--SY-GNEDE------VSSSSDNA---------------------------
ASKPTRSPLLGGSPKLPR------AEHALLN--GQKPLALGLLST------------------STSTP----DSYGL---STTAG---THP-
GTPSME-QVRTP---TSTSA------------QDEIHPLEILQAQIQLQRQQFSISEDQPLGLK-------------SKKGECAG------
QNGD--SDLGSCC---S---EGVKGA--------MSTIDLDSLMAEHNS-TWYLPG-------EK-ALMEG----QEEDKP--
MAPWEKPKPPNP-SKEAHDLPPSKTS-------------ATAQTGSHLQCLSVHCTDDVGETKGRT-
AVPTWRSLHSDISNRFGTFVAALT----------------------------------

>E.caballus

-------MFGL-DQFEPQ--------INSRN--------AGQGER------NF-NEAGLS----MNAHFK-APA-FH----------AG-----G-P--P-
GPVDP-AMSALGEPP--M-LG--------MNM-------EPYG-FH-AR-GHSELH-------------AGGL------Q------------AQP--------
-------VH----GFFGG---QQPH-HGHPGVHH-P------HQ-HHSHFG-GNFGGPDPGA-SCLHGGRLL----GY--GGAG---
GGLGSQ----------PPFAE--GYD-HMAES-Q-------------------GPESFG------P---------Q---------------------RPGNLPDF------
----HSSGASGHAVPAPCLPLD----QSPNRAASFHGLP--AS---------------------SGSDSHSLE----PRR-VANQ-G--
AVDSLE------------YNYPG-EAPSG---HFDM--FS-PSD-S--EGQLP-HYAAGRQV--P-GGSFPG-ASSM--PRAAGM--
VGLSKMH--A---------------------QQQQQQQQQQQQQQH--GVFFERF--GGARKMPVSLEPG--VGS-RHPLM--QP---
-----PQQAPPPPQQPPP-QQP-------QQPQQPQPPPPGLLVRQNSCPPALPR--PQQGEAGTPSGGL-QDGG-PMLP-
SQHAQ--------FEYPIHRLENRSM--------------HPY--SEA---VFNM----QHP---A-------P----QQAP------
NQRLQHFDAPPYMNVAKRPRFDFPGS-A----G-VDRCASWNG-----S-MH-NGALDNHL------------SPS-A-YS----------
---GLPGEFTP--PVPDS---F-PSGPPLQHPAP----DHQSL-----QQQQQQQ---------QQQQQQ----QRQN--------
AALMIKQMASRN------------QQQR-LR-QPS---LAQLGHPGDVG----QG-SLVHGGPVGGLAQ-P-NFERD--------------
-------SGGAGA---------------GRLGTFEQQAP-HLAQES--AWFPGPH---P---SPGDLL--------
PRRMGGSGLPADCGPHD---PGLAPPPPP--GGSGVLFRG--P--------LQE-P----LR-MP--GEGHVPALPSP-----GL--
QFGGS----LASLGQLQSPGAG-----VGLPSAPSER-R--PPPPDFSAP---ALAGQPGFPF-GAANRQ-ATPHSG-
PGVNSPPSTGGGGGS-TGGGGGGGGGGGAY--PPQPDF--QPSQR----SSASKLGALSLG--------SFNK----------PSS------
------KDN-LFGQSCLAALSTACQNMIASLGAP--------NLNVTFNKKN---P-PEGKRKLSQNETEGA-----------------------
-TVAGNPGSDYFP--GGAAPGAPGPGGPSGTSSSGSKASGP--------PNPPAPGD-GTS--LSPNYT--------------------------
-----------------L---------ES-TSGNDGK---PVPGGGGRGR---
GRRKRD----------SGHVSP--------GTFFDKY--------SAAPDSGGAPGVS-P-GQQQAPGAAVGG------SSTGEAR---------
-------GAPTPH---E-KALTSP--SW----GKG-----AELL------------LGDQPD-LMA--SLD--GG-----AKS---DSSSP----
HVGEFASDE-VST--SY-ANEDE------VSSSSDNPPA-------------------------LAKASRSPLVTGSPKLPP-
RGVVAGEHGPKA----PPLGLGIMST------------------STSTP----DSYG-------GGGGAGHP-GTPGLE-
QVRTPTSSSGAPP------------PDEIHPLEILQAQIQLQRQQFSISEDQPLGLK-------------GGKKGECAV-
GASGAQNGD--SELSSCC---S---EAVKSA--------MSTIDLDSLMAEHSA-TWYMPA-------DK-ALVDG----ADDDKT--
LAPWEKAKPQNPNSKEAHDLPTNKAS--------------AAQPGSHLQCLAVHCTDDVGDAKARA-
SVPTWRSLHSDISNRFGTFVAALT----------------------------------

>M.musculus

-------MFGL-DQFEPQ--------INSRH--------AGQGEG------NF-NEAGLS----MNAHFK-APA-FH----------AG-----P-P--T-
GPVDP-AISALGEPP--I-LG--------LNM-------EPYG-FH-AR-SHSELH-------------AGGL------Q------------AQP------------
---VH----GFFGG---QQPH-HSHPGGHH-P------HQ-HHPHFG-GNFGGPDPGA-SCLHGGRLL----GY--GGAA---
GGLGSQ----------PPFAE--SYE-HMAES-Q-------------------GPEGFG------P---------Q---------------------RPGNLPDF------
----HSSGTSGHAVPAPCLPLD----QSPNRAASFHGLS--AS---------------------SGSDSHSLE----PRR-VTNQ-G--
AVDSLE------------YNYPS-EPPSG---HFDM--FS-PSD-S--EGQLP-HYAAGRQV--P-GGAFPG-ASAM--PRASGM--
VGLSKMH--S-----------------------QPPQPPPQQQPQH--GVFFERF--GGARKMPVGLEPA--VGS-RHPLM--QP--------
PQQAPPPPQQPPP---------------QQQPPPPGLLVRQNSCPPALPR--PQQGEAGTPSGGL-QDGG-PMLP-SQHAQ--------

FEYPIHRLENRSM--------------HPY--SEP---VFSM----QHP---P-------P----QQAP------
NQRLQHFDAPPYMNVAKRPRFDFPGS-A----G-VDRCASWNG-----S-MH-NGTLDNQL------------SPS-A-YP----------
---GLPGEFTP--PVPDS---F-SSGPPLQHPGP----DHQSL-----QQQQQQQQQQQQQQQQQQQQQQQQQQ--QRQN--------
AALMIKQMASRN------------QQQR-LR-QPN---LAQLGHPGDVG----QG-GLVHGGSVGGLAQ-T-NFERE--------------
--------GGSAGA---------------GRLSGFEQQAP-HLAQES--AWFPGPH-------PPGDLL--------
PRRMGGAGLPTDCGPHD---PALAPPPAP--GGSGVLFRG--S--------LQE-P----LR-MP--GEGHVPALASP-----GL--
QFGGS----LAGLGQLQSPGAG-----VGLPNAPSER-R--PPPPDFPAP---ALGGQPGFPF-GSGSRQ-ATPHSA-
PGVNSPPSAGSG----SSGAGGG----AY--PPQPDF--QPSQR----NSASKLGALSLG--------SFNK----------PSS------------
KDN-LFGQSCLAALSTACQNMIASLGAP--------NLNVTFNKKN---P-PEGKRKLSQNEPDSA------------------------
VAAGNPGSDYFP--GGTTPGAPGPGGPSGTSGGGSKASGP--------PNPPIQGD-STS--LSPNYT---------------------------
---------------L---------ES-TSGNDGK---PVPGGSGRGR---
GRRKRD----------SGHVSP--------GTFFDKY--------STAPDSGGAPGVS-P-GQQQAPGSAAGG-------SSVNEA----------
------RGPTPH---E-KALTSP--SW----GKG-----AELL------------LGDQPD-LMA--SLD--ST-----AKS---DGSSP----
HVGEFASDE-VST--SY-ANEDE------VSSSSDNTTA-------------------------LAKASRSPLVTSSPKLPP-----
RGVGAGEHTPKASALGLGILST------------------STSTP----DSYG-------GGVGTGHP-GTPGLE-QVRTPTSSSGAQP---
---------PDEIHPLEILQAQIQLQRQQFSISEDQPLGLK-------------GSKKAECAV-GASGAQNGD--SELGSCC---S---
EAVKSA--------MSTIDLDSLMAEHST-TWYMPP-------DK-ALVDG----GDEDKT--
LAPWEKAKSQNPNNKEAHDHPTNKAS--------------ATQPGSHLQCLTVHCTD--GDPKART-
SVPTWRSLHSDISNRFGTFVAALT----------------------------------

>H.sapiens

-------MFGL-DQFEPQ--------VNSRN--------AGQGER------NF-NETGLS----MNTHFK-APA-FH----------TG-----G-P--P-
GPVDP-AMSALGEPP--I-LG--------MNM-------EPYG-FH-AR-GHSELH-------------AGGL------Q------------AQP---------
------VH----GFFGG---QQPH-HGHPGSHH-P------HQ-HHPHFG-GNFGGPDPGA-SCLHGGRLL----GY--GGAA---
GGLGSQ----------PPFAE--GYE-HMAES-Q-------------------GPESFG------P---------Q---------------------RPGNLPDF------
----HSSGASSHAVPAPCLPLD----QSPNRAASFHGLP--SS---------------------SGSDSHSLE----PRR-VTNQ-G--
AVDSLE------------YNYPG-EAPSG---HFDM--FS-PSD-S--EGQLP-HYAAGRQV--P-GGAFPG-ASAM--PRAAGM--
VGLSKMH--A-----------------QPPQQQPQQQQQPQQQQQQH--GVFFERF--SGARKMPVGLEPS--VGS-RHPLM--QP-
-------PQQAPPPPQQQPP----------QQPPQQQPPPPPGLLVRQNSCPPALPR--PQQGEAGTPSGGL-QDGG-PMLP-
SQHAQ--------FEYPIHRLENRSM--------------HPY--SEP---VFSM----QHP---P-------P----QQAP------
NQRLQHFDAPPYMNVAKRPRFDFPGS-A----G-VDRCASWNG-----S-MH-NGALDNHL------------SPS-A-YP----------
---GLPGEFTP--PVPDS---F-PSGPPLQHPAP----DHQSL-----QQQQQQQQQQQQQQQQQQQQQQQQQQQQQQRQN-----
---AALMIKQMASRN------------QQQR-LR-QPN---LAQLGHPGDVG----QG-GLVHGGPVGGLAQ-P-NFERE-----------
-----------GGSTGA---------------GRLGTFEQQAP-HLAQES--AWFSGPH---P---PPGDLL--------
PRRMGGSGLPADCGPHD---PSLAPPPPP--GGSGVLFRG--P--------LQE-P----MR-MP--GEGHVPALPSP-----GL--
QFGGS----LGGLGQLQSPGAG-----VGLPSAASER-R--PPPPDFATS---ALGGQPGFPF-GAAGRQ-STPHSG-
PGVNSPPSAGGGGGS-SGGGGGGG---AY--PPQPDF--QPSQR----TSASKLGALSLG--------SFNK----------PSS----------
--KDN-LFGQSCLAALSTACQNMIASLGAP--------NLNVTFNKKN---P-PEGKRKLSQNETDGA------------------------
AVAGNPGSDYFP--GGTAPGAPGPGGPSGTSSSGSKASGP--------PNPPAQGD-GTS--LSPNYT---------------------------
----------------L---------ES-TSGNDGK---PVSGGGGRGR---
GRRKRD----------SGHVSP--------GTFFDKY--------SAAPDSGGAPGVS-PGQQQASGAAVGGS-------SAGETR---------
--------GAPTPH---E-KALTSP--SW----GKG-----AELL------------LGDQPD-LIG--SLD--GG-----AKS---DSSSP----
NVGEFASDE-VST--SY-ANEDE------VSSSSDNPQA-------------------------LVKASRSPLVTGSPKLPP-
RGVGAGEHGPKA--PPPALGLGIMSN------------------STSTP----DSYG-------GGGGPGHP-GTPGLE-
QVRTPTSSSGAPP------------PDEIHPLEILQAQIQLQRQQFSISEDQPLGLK-------------GGKKGECAV-
GASGAQNGD--SELGSCC---S---EAVKSA--------MSTIDLDSLMAEHSA-AWYMPA-------DK-ALVDS----ADDDKT--
LAPWEKAKPQNPNSKEAHDLPANKAS--------------ASQPGSHLQCLSVHCTDDVGDAKARA-
SVPTWRSLHSDISNRFGTFVAALT----------------------------------

>C.anna

-------MFGL-EQFEPQ--------SNSRS--------GGQAER------GF-GQPGLS----MSAHFK-APA-FP----------GG-----G-P-AA-
AAVDP-ALGALGEPP--L-LG--------MNM---SLAGDAYG-FP-GR-GPSELH-------------GGGM------Q------------P-P------
---------VH----GFFGG---QQPH-GGHGGAHH-P------HQ-HPPHFG-GNF-GPDPGA-SCVHGGRLL----GY--SGAL---
GG---Q----------TAFAD--GYE-HMAES-Q-------------------GGEGFG------Q---------Q---------------------RPGNLPDF-------
-QHHSAGGSSHAVPAPCLPLD----QSPSRAASFHGLP--AA---------------------GSSEPHGLE----QRR-LPAQ-G--
GVDSLE------------YNYPG-DGPAG---HFELPVFS-PSE-P--EGQLP-HYGGGRQV--PTGGSFAG-APAL--PRAPGM--

A-VAKAH--P----------------------P----------QQH--GVFFERF--GGARKMSASLEPG--ASA-RHPLI--QQ--------Q-----
PPQ-PPQ-------------------QPPGLLARQNSCPPAIPR--QQQTEANAPNPNL-QDNG-PIMQ-NQHAQ--------
FEYPIHRLENRNM--------------HPY--TDP---VFNM----QHP---P-------P----QQPP------NQRLQHFDA-
PYVSVAKRPRFDYPGN-P----G-VERCASWGS-----G-MH-GPAMESHL------------SPT-A-YP------------GLPGEFTP--
PAPEA---F---GGPLPHGGP----EHPAL-----A---------------------------QRQN--------AALVMKQMASRS-------------QQR-
LR-PPS---LQQLGHHGEVG----PP---------GGLPP-P-AFERE-----------------------AGGGR-------------------GFDPPAP-
HLAPDS--AWFAGP---PP---PPGELL--------QRRMAAPGLPAEAAPHE---LGL----QP--GGAAVLFRP--G--AGGL-
GLQE-P----LR-MA--GEGPAQALPSP-----GVHPPFAPA----MGGLSQLQSPGGG-----VALPSAPAER-R---
GPTDFAAQ---P-----GFPF-AAAARQ-PASHGPTPALSASP-------------------GAY-PPPPPEF-PPPPPP---
RPAASKLGALSLG--------SFSK----------PAS------------KDN-VFGQSCLAALSTACQNMIASLGAP--------
NLNVTFNKKS---P-AEAKRKLSQAEPDP---------------------------PPPAAPDYFP--AGPPSGTAGAG----------KVSGP----
------APLIPA-ESS--LSPGYA---L---------EP-AAGGEGK------AGGGRGR-----------------
--GRRKRD----------SGHVSP--------GTFFEKF----------SAAEGGGAGVS-P-
GQPAVPAAAGGP-------PGPAER----------------GGGTPH---D-KPLTSP--TW----GKG-----GELL------------LGEQPD-
LMS--SLD--SGIQSV-TKS---DGSSP-----HVDFP-DE-VST--SY-GNEDE------VSSSSDNA----------------------------
ASKPTRSPLLGGSPKLPR------GEHTLLN--GQKPLALGLLST------------------STSTP----DSYGL---STTAG---AHP-
GTPSME-QVRTP---TSTSA------------QDEIHPLEILQAQIQLQRQQFSISEDQPLGLK--------------SKKGECAG------
QNGD--SDLGSCC---S---EGVKGA--------MSTIDLDSLMAEHNS-TWYLPG-------EK-TLMEG----QEEDKP--
MAPWEKPKPPNP-SKEAHDLPPSKTP---------------AQSGSHLQCLSVHCTDEVGEAKGRT-
AVPTWRSLHSDISNRFGTFVAALT-----------------------------------

>C.japonica

-------MFGL-EQFEPQ--------MSSRS--------GGQGER------GF-GQPGLS----MSAHFK-APA-FG----------GG-----G-P--A-
AAVDP-ALGALGEPP--L-LG--------MNM---SLPGDGYG-FP-GR-GPAELH-------------GAGM------Q------------P-P------
---------MH----GFFGG---QQPH-GGHGGAHH-PHQHQHQHQ-HQPHFG-GNF-GPDPGA-SCVHGGRLL----GY--
NGAL---GG---Q----------TAFAD--GYE-HMAES-Q-------------------GAEGFG------Q---------Q----------------------
RAGNLPDF--------QQHSAGASGHAVPAPCLPLD----QSPNRAASFHGLP--AA-------------------GSSEPHGLE----
QRR-LPAQ------DSLE------------YNYPG-DGPAA---HFELPVFS-PSE-P--EGQLP-HYGGGRQV--PAGGGFAG-T-AL--
PRAPGM--A-VAKAQ--P----------------------PPPPPQQQQQQQH--GVFFERF--GGARKMPGSLEPG--PGG-RHPLM-
-QQ--------QQ---------PQ-------------------QPPGLLARQNSCPPAIPR--QQQTEANAPNPNL-QDNG-PIMQ-NQHAQ-----
---FEYPIHRLENRNM--------------HPY--ADP---VFNM----QHP---P-------P----QQPP------NQRLQHFDA-
PYVSVAKRPRFDFPGN-P----G-VERCASWAG-----G-MH-GPAVESHL------------SPT-A-YP-------------GLPGDFTP--
PAPDA---F---GGPLPHGGP----DHPAL-----S--------------------------QRQN--------AALVMKQMASRS-------------QQR-
LR-PPS---LQQLGHHGDVG----PP---------GSLPP-P-AFERE----------------------AAAAAR-------------------GFDAPAP-
HLAPDG--TWFAG----PP---PPGELL--------PRRMAGPGLPAEPAPHE---LGL----QP--GGAAVLFRP--G--TGAL-
GLQE-P----LR-MA--GEGPAQALPSP-----GVHPPFAPS----VGGLSQLQSPGGG-----VALPSAPSER-R---GPADFAAQ-
--P-----GFPF-GAAARQ-PPSHGNAPALSASP-------------------GAY-PPPPTEF-PPPPPP---RPAASKLGALSLG--------
SFSK----------PAG------------KDN-VFGQSCLAALSTACQNMIASLGAP--------NLNVTFNKKS---P-
AEAKRKLSQAEPDP---------------------------PPPAAPDYFP--AGPTAG-GGAG----------KA-------------APLLPA-ESS--
LSPGFA---L---------EP-AAGGEGK------AGGGRGR---
----------------------------GRRKRD----------SGHVSP--------GTFFEKF----------STAEGGGAGVS-P-GQPAPPAAGGPT----
--GASGAER----------------GGGTPH---D-KPLTSP--SW----GKG-----GELL------------LGEQPD-LMS--SLD--SGIQSV-
TKS---DGSSP-----HVDFA-DE-VST--SY-GNEDE------VSSSSDNA----------------------------GPKPTRSPLLGGSPKLPR--
----GEHALLN--GQKPLALGLLNT------------------STSTP----DSYGL---SSTAG---AHP-GTPGME-QVRTP---TSTST-----
-------QDEIHPLEILQAQIQLQRQQFSISEDQPLGLK--------------SKKGECAG------QNGD--SDLSGCC---S---
EGVKNT--------MSTIDLDSLMAEHNS-TWYLPG-------EK-ALMEG----QEEDKP--MAPWEKPKPTNP-
SKEAHDLPPSKTS-------------AAAQTGSHLQCLSVHCTDDVGDAKGRT-AVPTWRSLHSDISNRFGTFVAALT------
----

>S.habroptila

-------MFGL-EQFEPQ--------SSSRS--------GGQAER------GF-GQPGLS----MSAHFK-APA-FP----------GG-----G-PAAA-
AAVDP-ALSALGEPP--L-LG--------MNM---SLPADAYG-FP-GR-GPAELH-------------GGGM------Q------------P-P------
---------VH----GFFGG---QTAH-GGHGGGHH-P------HQ-HPPHFG-GNF-GPDPGA-SCVHGGRLL----GY--SGAL---
GG---Q----------TAFAD--GYE-HMAEG-Q-------------------GGEGFG------Q---------Q---------------------RPGNLPDF------
--QHHSAGGSSHAVPAPCLPLD----QSPNRAASFHGLP--AA-------------------GSSEPHGLE----QRR-LPAQ-G--
SVDSLE------------YNYPG-DGPAG---HFELPVFS-PSE-P--EGQLP-HYGGGRQV--PAGGSYAG-APAL--PRAPGM--

A-VAKAH--P----------------------P----------QQH--GVFFERF--GGTRKMSASLEPG--ASA-RHPLM--QQ--------QQ---
PPPQPPQQ-------------------PPPGLLARQNSCPPAIPR--QQQTEANAPNPNL-QDNG-PIMQ-NQHAQ--------
FEYPIHRLENRNM--------------HPY--TDP---VFNM----QHP---P-------P----QQPP------NQRLQHFDA-
PYVSVAKRPRFDFPGN-P----G-VERCTSWGS-----G-MH-GPAMESHL------------SPT-A-YP-------------GLPGDFTP--
PAPEA---F---GGPLPHGGP----EHPAL-----A--------------------------QRQN--------AALVMKQMASRS-------------QQR-
LR-PPS---LQQLGHHGEVG----PP---------GGLPP-P-AFERE----------------------AGGGR-------------------GFDPPAP-
HLAPDS--AWFAG----PP---PPGELL--------PRRMVAPGLPAEAAPHE---LGL----QP--GGTAVLFRP--G--AGGL-
GLQE-P----LR-MA--GEGPAQALPSP-----GVHPPFAPA----MGGLSQLQSPGGG-----VALPSAPAER-R---
GPADFAAQ---P-----GFPF-AAAARQ-PAAHGAAPALSASP-------------------GAY-PPPPPEF-PPPPPP---
RPAASKLGALSLG--------SFSK----------PAS------------KDN-VFGQSCLAALSTACQNMIASLGAP--------
NLNVTFNKKS---P-AEAKRKLSQAEPDP---------------------------PPPAAPDYFP--PGPPAGGGGAG----------KAPGT---
-------APLLPA-ESS--LSPGFA--L---------EP-AASGEGK------AGGGRGR----------------
--GRRKRD----------SGHVSP--------GTFFEKF----------SATEGSGAGVS-P-
GQPAAAGGPPGA--------AGTER----------------GGGTPH---D-KPLTSP--SW----GKG-----GELL------------LGEQPD-
LMS--SLD--SGIQSV-TKS---DGSSP-----HVDFP-DE-VST--SY-GNEDE------VSSSSDNA----------------------------
ASKPTRSPLLGGSPKLSR------GEHALLN--GQKPLALGLLST------------------STSTP----DSYGL---STTAG---THP-
GTPSME-QVRTP---TSTSA------------QDEIHPLEILQAQIQLQRQQFSISEDQPLGLK--------------SKKGECAG------
QNGD--SDLGSCC---S---EGVKGT--------MSTIDLDSLMAEHNS-TWYLPG-------EK-ALMEG----QEEDKP--
MVPWEKPKPPNP-SKEAHDLPPSKTT--------------AAQTGSHLQCLSVHCTDDVGEAKGRT-
AVPTWRSLHSDISNRFGTFVAALT----------------------------------

>P.cinereus

-------MFGL-DQFEPP--------INNRN--------AGQGER------NF-GEPGL----SMNGHFK-APA-FH----------AG------------
GPADP-TIGALGEPP--L-LG--------MNM---GMAGEPY-GFH-GR-GHSELH-------------AGGL------------------QPQP-----
----------VH----GFFGS---QQPH-HGHPGGHH--------PHQHHPHFG-GNFAGPDPGA-SCLHGGRLL----GYGGGG-----
GGLSNQ----------PGFAE--GYE-HMADT-Q-------------------GGEGFGQQ------------------------------------RPGNIPEF-----
---QHHSSGPSGHAVPAPCLPLD----QSPNRAASFHGLP--AS---------------------SSSDSHSLE----ARR-MANQ-G--
GVESLE------------YNYPG-DGPSG---HFDLPVFS-PSE-S--EGQLP-HYGTGRQV--PGG-AFPG-ASAL--PRAPGM--
VAMSKIH-----------------------------PQQQQQQQQHGGGVFFERF--GGARKMSVGMEPG--
VNAARHPLMQQAPQPPQPQPLPPQPQPQPPRPQQQQQ-----------------QPSLLARQNSCPPALPR--
QPQAEGNPAGGSL-QDGG-PILP-NQHAQ--------FEYPIHRLENRSM--------------HPY--SDP---VFTIQHPPPPP----------
-APQQQQQPPPPPQAPNQRLQHFDA-PYLNVAKRARFDFPST------PTVDRCASWNG-------TLHSGALDNHL--------
----SPS-A-YP-------------GLPGDFTP--PVPES---F-PPGPALQHPGP----DPQALQQQQQQQQQQQ-------------------
QRQN--------AALMMKQMASRT----------QQQQQQRLR-QPG---LPPLSHPGDVG----PGSGLVHGGPVASLAQ-A-
NFERE-----------------------GGGGGGGGGGGSSGGGGGGAGRMGNFDPQNP-HLAQES--AWFPGPHPPPP---
PPGDLL--------PRRLGGSNLPPDGSPHE---LGL----AQ--GGSGMLFRG-PG--VAGL-GLQE------SLRMP--
GEGHVPALHSP-----GLHSQFGGS----LGGLGQLQSPGAG-----VGLPSTPSDRRA---PPPDFAAP---PLGGQPGFPF-
GGTSRQ-TTPLSN-PGVSASP---------GGGGGGGGGGGTY--PPQPDF--QPSQR----ASASKLGALSLG--------SFNK----
------PNS------------KDN-LFGQSCLAALSTACQNMIASLGAP--------NLNVTFNKKN---Q-
SESKRKLSQTDSDGAVGSGGGGSGGSGGAG-------------NSGPDYFP--SGTAPGGPGPGGPGGTSGGGGKAPGP----
----PNPPAPGD-GTS--LSPNYT---IEATSGNDGKPVPGG---GGRGR------------
---GRRKRD----------SGHVSP--------GTFFDKF----
SAAAAAAAADSGXSGVS-P-GQQAAPGPPGGS-------SGGEPR----------------GAPTPH---D-KALTSP--SW----GKG----
-AELL------------LGDQAD-LMS--SLD--SGIQSG-AKS---AGSSP----HVGDFA-DE-VST--GY-ANEDE------
VSSSSDNAASA-------------------------LAKPSRSPLVTGSPKIPR------GPAGEHG--QKAAPGLGGLLTT-----------------
STSTP----DSYGLSSSS--GA---AHP-GTPGLE-QVRTP--TSSTSP------------
QDEIHPLEILQAQIQLQRQQFSISEDQPLGPK-------GGGNGGGGKKAECAG-----GQNGD--SELGACC---P---
EAVKSA--------MSTIDLDSLMAEHSS-TWYMPG-------EK-ALLDG----SEDDKT--
LAPWEKSKPQNPVSKEGHDLPQNKTS-------------AAAQTGSHLQCLSVHCTDDVGEAKGRT-
SVPTWRSLHSDISNRFGTFVAALT----------------------------------

>L.erinacea

-------MFGL-NQFSRS---------SGQG-----------AER------NF-GQ-AAL---GMSTHYK-GPG-FPGGGA--GGGGG-----G-
G--S-SMGEQ-GISGLNEPP--M-LS--------MGMSP-GLGGDQY-PFH-PR-GHSELH-----------------------------
QPPPPPPPPPQPQS-----QAPAPAAH----VYYPNGHQHH-----HPHAHP------------HHQYS-GAFCGSEPSP-
SCLHGGRLLAA-PGYASNPLS---GQ---T----------PGFGE--SYD-AMGEN-A----------------GGGGGEAFGQQQQQQ--

-------QQPPSQQQQQQQQHQQAPAGPGRAGTLTDY--------HQHHTPASSH---TPCLPLD----QSPNRAASFHALP--
SS--------------------APSEAPGLE----QRR-LHTQ-P--PVDSMD------------YNYQG-DP------HFDMPVFS-PSE-A--
GAQLP-HYGSGRQV--PGGSSFPG-SPAV--PRTTGM--VNMGKVH--P-----------------------------PQ---QQ--
GIFYERFGGGNARKMPVGLEAG--AGGVRNSLL--QQ--------QQ------------------------------
QAGLLGRQNSCPPAIPR--QEAGAATPSGS---QDSG-EMMQ-PQHAP--------FEYPIQRLENRNL--------------HPY--
SEP---MFNV-----------------------QQQP------NQRLQHFDA-PYLNVAKRARFDFANS-H----G-VDNCTNWSG-----N-
GLHNPALDSHL------------SPT-ATYP-------------GLPAEFSP--PGPEG---F-QPGPPLQHPGS----EQ----------Q------------
-----------HRHN--------MLMMFKQMVSRN------------QRHR-MR-QPD---LQPMGHHGDAG----PS-
GLVHGGQVGGMVQ-G-SFERDGVGGGS----------GGGGGGGGGRMP-------------------GFDAQNP-AMAQES--
SWFPG----PH---PPADLL--------PRRMGGGG--GERSPHESVQMNL----QQ--NGSNLLFRT--A--GNGL-ALQE-P----
MR-MP--SEGHVQGLHSP-----GIHAQFGGP----MGGLPQMQSPGPS-----IGLPNASGDR-R--AAAADFPGP---
QMGGQAGFPF-AGPSRPP----NNPPGVPPSP-------------------GGY--PGQPDF--QAGQR----PSMSKIGSLSLG--------
SFSK----------PGA------------KDNTMYGQSCLAALSTACQNMIASLGAP--------NLNVTFNKKN---
QGSDGKRKLSQTEQEGV--GPASGGGAAAGAGAGAGGGGTSSGGAGNGPEYFQ--SNAPQNNQM-------GGG-
GKLAAQ-----TQGP--AAQPE--CN--LSPNYG--M---------EA-AP-VEAK--GQT----
GRGR---GRRKRD----------SGHVSP--------GNFFDKY-----------
SAENVNPVVS-P-GPQCQPTLAGER-----------------------------GGGTPQ---D-KGLTSP--SW----AKG-----SDLL-----------
-LPDQPD-LMS--SLD--SGIQSV-AKS---DGSSP-----HVDFP-DE-VGP--NY-GNEDE------VSSSSDNN---------------------
---------LSKPGQLMTSSPKLPR-----------VEQHGLKPMGHSMLTTN-----------------TTSNPGPGADSF-------------GHP-
GTPGIE-QVRTP--TSTSSS------------QDEIHPLEILQAQIQLQRQQFSISEDQPLAMK--------------NKKGECPA------
QNGD--TELAACG---T---DNGKAA--------ISTIDIESLMAEHNS-TWYMPS-------DK-AMMDP----QEDDKQ--
MAPWDKVKNSTT-NKEAHEPSQNKAP--------------SVSADSHLQCLSVHCTDDLTDSKGRS-
PMQTWRSLHSDISNRFGTFVAALT-----------------------------------

>C.caretta

-------MFGL-EQFEPQ--------ISSRN--------AGQGER------NF-SQAGLT----MGSHFK-SPA-FH----------AG-----G-P------
ADP-AISALGEPP--I-LG--------MNM---NMAGEAYG-FH-AR-GHSELH-------------AGGM------Q-----------AQP--------
-------VH----GFFGN---QQPH-HGHPTTHH-P------HQ-HHPHFS-GNF-GSDPSA-SCLHGGRLM----SY--SSSV---GS---
Q----------QAFAE--GYE-HMAEN-Q-------------------GGEGFG------Q---------Q---------------------RSGNMPDF--------
QHHNSSASNHAVPAPCLPLD----QSPNRAASFHGLP--AS---------------------SSSDSHSLE----QRR-LPNP-G--
GVDSLE------------YNYAS-DGPSG---HFDLPVFS-PSE-S--EGQLP-HYGAGRQV--PGGGSFPG-TSVL--PRAPGL--
VGMSKVH--P---------------------QQQ----------QQH--GVFFDRF--GGARKMSVGMEPG--VNA-RHPLM--QQ--------QP-
------------------------------QAGLLARQNSCPPAIPR--QQQTEASAPNPSL-QDNG-PLLQ-NQHAQ--------
FEYPIHRLENRNM--------------HPY--PDP---VFNM----QPP---P-------P----QQPP------NQRLQHFDA-
PYMSVAKRPRFDFPSS-P----A-VDRCAAWSN-----P-LH-GAGMENHL------------SPS-A-YP-------------GLAGEFTP--
PVPES---F-APGPPLQHPGP----DPQAL-----Q--------------------------QRQN--------AALMIKQMASRS------------QQQR-
LG--PS---LQQLGHHGDLG----QGGLA-HAGPVGTMPQ-P-GFERD----------------------SGGRGP-------------------
GFEPPAP-HLAQDS--GWFPGP---PP---PGGELLPPPPPPPPPRRLGG---PAEPGPHE---LSL----AP--GGSGLLFRP--A--
AGGL-GL------------A--GEGRGPALHSP-----GVHAPFG-------AGLAPLPSPGAGGGGGVVGLPSAPAER-R---
PPADFAAP---PLGGPAAFGF-GASGRP-APPPSA----SGSP-------------------GAF-P--PPEF--PPAPR---
AAAASKLGALSLG--------SFAK----------PA-------------KEA-AFGQSCLAALSTACQNMIASLGAP--------
NLHVTFAKRG---P-PEARRKPGPPEPDGGA-----------------------APGPAPGPDFFP--A-----------------------KA----------
AP-----EAS--PSPGFP--P---------EA-APGGEGKAAAAAAAAGGRGR-------------------
--GRRKRD----------SGHVSP--------GGFFDKF----------------PPAEG---
GGAASPGPGPGP-----------------------------GAPPAA---HDRPLASP--SW----AKG-----GERP------------RGEQPD-LLA--
SLD--GGLPSA-SKS---DGGSP-----RGDFP-AE-PSP--AY-GHEDE------VSSSSDGGP---------------------------
AKPPTRSPLLGGSPP-----------QALLG--GQKPLALGLLGG------------------PGPAP----DGYGL------GGGGGGHP-
GTPGLE-QVRTP---GSSSA------------QDEIHPLEILQAQIQLQRQQFSISEDQPLGMK--------------SKKAECPS------
QNGE--GELNSCC---S---DSVKGA--------MSTIDLDSLMAEHNS-TWYMPS-------EK-SLMEG----QEEDKP--
MAPWEKSKPQNP-SKEAHDLPQNKTS-------------AAAQTGSHLQCLSVHCTDDMGESKGRT-
AVPTWRSLHSDISNRFGTFVAALT-----------------------------------

>N.scutatus

-------MFAL-DPFDSH------GGGSGRS--------GVPGER----GGGF-GPG-------AGSQFK-PSP-FQ----------GGSGGSGG-
G----GGGEPAAVNALGEPSSTL-LA--------MNL---TLSTEGYGGFHAPRGGPPELG-----------------------Q------------
AQP---------------IH----GFFGS---PPPG-PGAHHQTQ-P---------PVSHFG-GGF-GPEPSA-SCLHGGRLL----GY--SGG----

-----Q----------QTFATDGGYEQHLADG-Q------------------AGGDGFG------Q---------Q---------------------
RAGPLQDFQPPPPPPHHLHNPG-----VPAPCLPLD----QSPNRAASFHGLP--GA--------------------ASSEPHGLDS---
QRR-LPNQ-GAAAVDSLE------------YSYPN---SES---HFDLPVFS-SPE-A--EGQLP-
QYGSGRQAAPPGGSNFPGHSAAL--PRAPGS----LGKVP--T--------------------------------QQSPH--GVFFDRF--
GGARKMSLGLEPG--LGAGRHHLV--PQ--------Q----PPP-------------------------PPALLARQNSCPPALPR-
PQQCPEGSAANANL-QEGGGPILQ-AQHGQ--------FEYPIHRLENRGLQHHHHHQQQQ--QQHSYGPGEP---LFNV--
-PH-----Q-------P----QQPP------NQRLQHFDAPPYVNLPKRPRFD-----------------SWSGAG-GGG-MHGAAALESHL----
--------SPSAA-YP-------------GLPGDFTPPVPVPEH---F------PPPPGP----DPQAA-----L------Q-------------------QRQN-----
---AALMIKQMAATR-----G------QSQR-LR-PPS---LQQVGHHQHPH----TAGGHGHHQSPHP------------------------------
---HPGEPA--------------------------FEAQEG--AWFPP----PH---PP---------------------------------------A--GSGDLLFRP--G--
MG---GLQEPPPPPALR-MPSGGEGHVS---SP----GGLQGQFG---------------------------LSPPSER-R--PSHPDFAAQ-------
AQSFPF-GGPSRQ-ATPHSASPGSFGPP-------------------TDFQSSAPPPS--Q-PRP---SPASSKLGALSLG--------SFPKG-
----GVGAPPG-------SAAPKESSGLFGQSCLAALSTACQNMIASLGAP--------NLNVTFGKKG---A-PAAV------GG-G-
------------------------AGGEGAKRSKLS--PAEPPETNGAG------------PQP------PLPAATVPAG-ESG--LSPNYS------------
-------------------------------P------------GSGPDAK------AGSGRGR---
--GRRKRD----------SGHVSPAAGTTGSAGSFFEKY--------------GQTTGVD--------SGSPGQG----------GERS-------------
--GGGTPHLHEAPKALSSPPSTW----AKN--G--GDLL-LPPP-------PE-QAD-LLP--SLD------QV-------ESCSP----
RGGDFPADA---------ENEDE------VSSSSDNQ--------------------------------GAKGCPAGRSPLQPP---GRPADHPLLN--
GQKTSTLAQLGLHTGSST------------ATSSP----DSY--------GA---GPP-GPV-----------------------------
QEEIHPLEILQAQIQLQRQQFSISEDQPLGLK---------------SAKKGPEGSGGGQNEDS--GELSSCC-----EGAGAKGA-
-------VSTIDLESLMAEHSA-AWYLPG-------DK-ALLDG----EPDEKV--LVPWEKAKAPTP-GKEGHDLPSSKAL------
-------APAQTGSHLQCLSVHCTDDMGDTKGRT-AVPTWRSLHSDISNRFGTFVAALT-----------------------------------

>A.platyrhynchos

-------MFGL-EQFEPQ--------MSSRS--------AGQGER------GF-GQPGLS----MSAHFK-APA-FP----------GG-----G-
PAAA-AAVDP-ALGALGEPP--L-LG--------MNM---P-GGDAYG-FP-GR-GPAELH-------------GGGM------Q------------
P-P---------------VH----GFFGG---QQPH-GGHGGAHH-P------HQ-HHPHFG-GNF-GPDPGA-SCVHGGRLL----GY--
SGAL---GG---Q----------TAFAD--GYE-HMAES-Q-------------------GAEGFG------Q---------Q---------------------
RPGNLPDF--------QHHSAGASSHAVPAPCLPLD----QSPNRAASFHGLP--AA--------------------GSSEPHGLE----
QRR-LPAQ-G--GVDSLE------------YNYPG-DGPAG---HFELPVFS-PSE-P--EGQLP-HYGGGRQV--PAGGSYAG-
TPSL--PRPPGM--A-VAKAH--P-----------------------P----------QQH--GVFFERF--GGARKMSASLEPG--AST-RHPLM--
QQ--------QQ---QQPP-QPQ-------------------QPPGLLARQNSCPPAIPR--QQQTEANAPNPNL-QDNG-PIMQ-NQHAQ--
------FEYPIHRLENRNM--------------HPY--TDP---VFNM----QHP---P-------P----QQPP------NQRLQHFDA-
PYVSVAKRPRFDFPSN-P----G-VERCASWGG-----S-MH-GPAMESHL------------SPS-A-YP-------------GLPGEFTP--
PAPEA---F---GGPLPHGGP----EHPAL-----A-------------------------QRQN--------AALVMKQMASRS-------------QQR-
LR-PPS---LQQLGHHGEVG----PP---------GGLPP-P-AFERE-----------------------AGSGR-------------------GFDPPAP-
HLAPDG--AWFAG----PP---PPGELL--------PRRMAAPGLPAEAAPHE---LGL----QP--GGAAVLFRP--G--AGGL-
GLQE-P----LR-MA--GEGPAQALPSP-----GVHPPFAPA----MGGLSQLQSPGGG-----VSLPNAPAER-R---
GPADFAAQ---P-----GFPF-GAAARQ-PAAHGAAPALSASP-------------------GAY-PPPPPEF-PPPPPP---
RPAASKLGALSLG--------SFSK----------PAS------------KDN-VFGQSCLAALSTACQNMIASLGAP--------
NLNVTFNKKS---P-AEAKRKLSQAEPDP---------------------------PPSAAPDYFP--AGPPAGGGSAG----------KAAGA---
-------APLLPA-ESS--LSPGYA---L---------EP-AASGEGK------AGGGRGR---------------
---GRRKRD----------SGHVSP--------GTFFEKF----------SATEGSGAGVS-P-
GQPAPPAAGAPP------GAAGTER----------------GGGTPH---D-KPLTSP--SW----GKG-----GELL------------LGEQPD-
LMS--SLD--SGIQSV-TKS---DGSSP-----HVDFP-DE-VST--SY-GNEDE------VSSSSDNT----------------------------
VSKPTRSPLLGGSPKLPR------GEHALLN--GQKPLALGLLNT------------------STSTP----DSYGL---STTAG---AHP-
GTPGME-QVRTP---TSTSA------------QDEIHPLEILQAQIQLQRQQFSISEDQPLGLK--------------SKKGECAG------
QNGD--SDLSSCC---S---EGVKGA--------MSTIDLDSLMAEHNS-TWYLPG-------EK-ALMEG----QEEDKP--
MVPWEKPKPPNP-SKEAHDLPPSKTS-------------AAAQTGSHLQCLSVHCTDDVGEAKGRT-
AVPTWRSLHSDISNRFGTFVAALT-----------------------------------

>D.gliroides

-------MFGL-DQFEPP--------ISNRN--------AGQGER------NF-GEPGLS----MNGHFK-APA-FH----------AG------------
GPADP-TIGALGEPP--L-LG--------MNM---GMAGEPYG-FH-GR-GHSELH-------------AGGL------Q------------PQP-----
----------VH----GFFGG---QQPH-HGHPGGHH-S------HQ-HHPHFG-GNFAGPDPGA-SCLHGGRLL----GY--GGGG---
GGLGNQ----------PGFAE--GYE-HMAET-Q-------------------GGEGFG------Q---------Q---------------------RPGNIPEF-----

---QHHSSGPSGHAVPAPCLPLD----QSPNRAASFHGLP--AS--------------------SSSDSHSLE----ARR-MANQ-G--
GVESLE-----------YNYPG-DGPSG---HFDLPVFS-PSE-S--EGQLP-HYGTGRQV--P-GGAFPG-ASAL--PRAPGM--
VAMSKIH--P----------------------QQQQQQQQQHGG--GVFFERF--GGARKMSVGMEPG--VNTARHPLM-----------
--QQAPQPPQPQPP---PPQP-QPQPPPPQQQQQQPSLLARQNSCPPAIPR--QPQAEANPASGSL-QDGG-PILQ-
NQHAQ--------FEYPIHRLENRSM-------------HPY--SDP---VFSM----QHP---PPPSAPQQQ----PQAP------
NQRLQHFDA-PYMNVAKRARFDFPST-P----T-VDRCASWNG-----S-LH-SGALDNHL-----------SPS-A-YP------------
GLPGDFTP--PVPDS---F-PPGPALQHPGP----DPQAL------QQQQQQQ---------QQQQ------QRQN--------
AALMMKQMASRT------QQQQQQQQQR-LR-QPG---LQPLSHPGDVG----PGSGLVHGGPVAGLAQ-A-NFERE------
----------------GGGGGGSGGGGGASSGGGGGAGRLGNFDPQNP-HLAQES--AWFPGPHPPPP---PPGDLL--------
PRRLGGSNLPPDGSPHE---LGLA----Q--GGSGMLFRG--P-GVAGL-GLQE-S----LR-MP--GEGHVPALHSP-----
GLHSQFGGS----LGGLGQLQSPGAG-----VGLPSTPSDR-R--APPPDFTAP---PLGGQPGFPF-GGTSRQ-ATPLSN-
PGVSASP---------GGGGGGGGGGGTY--PPQPDF--QPSQR----ASASKLGALSLG--------SFNK---------PNS-----------
KDN-LFGQSCLAALSTACQNMIASLGAP--------NLNVTFNKKN---Q-PESKRKLSQTDSDGAVGGGGGG------------
SGSSGGAGNSGPDYFP--SGTAPGGPGPGGPGGTSGGGGKAPGP--------PNPPAPGD-GTS--LSPNYT-----------------
-----------------------I---------EA-TSGNDGK---PVPGGGGRGR---
GRRKRD----------SGHVSP--------GTFFDKF--------SAAAADSGGSGVS-P-GQQAAPGPPGGS-------SGGEPR-----------
-----GAPTPH---D-KALTSP--SW----GKG-----AELL-----------LGDQAD-LMS--SLD--SGIQSG-AKS---AGSSP----
HVGDFA-DE-VST--GY-ANEDE------VSSSSDNAAPA-----------------------LAKASRSPLVTGSPKIPR------
GPAGEHG--QKAAPGLGGLLTT----------------STSTP----DSYGLNSSS--GT---AHP-GTPGLE-QVRTP--TSSTSP-------
-----QDEIHPLEILQAQIQLQRQQFSISEDQPLGPKGGGSGGGSSGGGGGGKKAECAG-----GQNGD--SELGACC---P-
--EAVKSA--------MSTIDLDSLMAEHSS-TWYMPG-------DK-ALLDG----PEDDKT--
LAPWEKPKSQNPVSKEGHDLPQNKTS-------------AAAQTGSHLQCLSVHCTDDVGEAKGRT-
SVPTWRSLHSDISNRFGTFVAALT----------------------------------

>N.parkeri

-------MFGL-EQFEPQ--------INSRS--------AGQGDR------NF-AQPGI----NMSAHFK-NAA-FH----------SG-----G-T--S-
GAVDP-AMGALSEPS--M-LP--------MNLTL-N--GEPY-GYH-AR-GHSELH------------TAGM-------------------QP------
---------VH----GFFNN-Q-QQQH-HTHPNSHP--------HQ-HHPHFP-GNFAGPEATA-SCLHGGRLM----GY--NNNL---
GN---Q----------QGFAE--GYE-PIPES-Q------------------SGEGFG---------------QQ-------------------RSGNLPDF--------
QHPNSTASNHAVPAPCLPLD----QSPNRAASFHGLP--AS--------------------TSSDSHNLE----QRR-IHNQ-G--
GVDALE-----------YNYPG-DGPSG---HFEVPVFS-PSD-S--EG----HYGAGRQV--PSS-AFPG-ASVL--PRPPGL--
VGMPKVH--P------------------------QQQQQQ---QH--GVFFERF--GGARKIPVGMEPA--VNG-RHPLL--QQ--------QQ-
-----------------------------QTGLLARQNSCPPAITR--QQQTEGSTSNPNL-PDNG-PVMQ-SQHSQ--------
FEYPIQRLENRNM-------------HPY--SES---MFSM---QQGP-----------P----QQPP------NQRLQHFDA-
PYMNVTKRPRFDFPNN-H----G-VDNCAAWNN-----N-SIHNAGIDSHL-----------SPS-T-YP------------GLPGEYPP--
QVPDS---F-PPGPALQHPGS----DHQSI-------Q-----------------------QRQN--------AAMMIKQMASRN-----------QQQR-
MR-QAN---LQQLGHHGDVN----QS-AIVHGGQVGSIAQ-P-NFDRE----------------------GGRIA------------------
NFDPQNP-HVGQEN--AWFPG----PH---PPGDIL--------QRRMGGSNLPADPTSHD---INL----QQ--NGTNMLFRP--G--
VNRM-GMQE-P----LG-IP--GEGHVPALHSP-----GMHSQYGNN----MANLSQMQSPGGG-----VGMNSTPADR-R---
GAPDFAPP---AIGQQSGFPF-VGSNRQ-TTPH-NPPGVNSSP------------------NSY--PPQSDF--QASQR----
STASKLGALSLG--------SFSK----------TNP-----------KEN-MFGQSCLAALSTACQNMIASLGAP--------
NLNVTFNKKS---Q-AEGKRKLSQTETELN--G-NSG-N------------------STTSDYFS--GGSSQGNQGPG----
ATTNNSKSTGQ------SGPSQPTQGE--TS--LSPNYN--I---------EV-TPGNDGK--PVT-
-GGGRGR--GRRKRD----------SGHVSP--------GNYFDKY-------
-----SADSGGAVVSP-GQQGQTANPIE-----------------------------PGGPPH---D-KPLTSP--SW----GKG-----NELL---------
---LSDQPD-LMS--SLD--SGIQSV-TKS---DSSSP-----HVDFS-EE-VNT--TY-GNEDE------VSSSSDNN-------------------
-------ISKPNNCPLVTGSPKIQR--------SELLN--GQKAMGLNLLNN------------------TTSLP----DSYGL---SSTGA---
GHP-GTPGME-QVRTP---TSTST------------QDEIHPLEILQAQIQLQRQQFSISEDQPLGMK--------------NKKSDCTA--
----QNVD--SELNSCC---S---DNVKNS--------MSTIDLDSLMAEHNS-TWYLPN-------EK-SLMEG----EEDDKS--
ITPWEKSKSQQT-NKEAHDLPQNKTS-------------AAAQNGSHLQCLSVHCTDDIGESKGRT-
PVPTWRSLHSDISNRFGTFVAALT----------------------------------

>C.olor

-------MFGL-EQFEPQ--------MSSRS--------AGQGER------GF-GQPGLS----MSAHFK-APA-FP----------GG-----G-
PAAA-AAVDP-ALGALGEPP--L-LG--------MNM---SLAGDAYG-FP-GR-GPAELH------------GGGM------Q-----------
P-P--------------VH----GFFGG---QQPH-GGHGGAHH-P------HQ-HHPHFG-GNF-GPDPGA-SCVHGGRLL----GY--

SGAL---GG---Q----------TAFAD--GYE-HMAES-Q-------------------GAEGFG------Q---------Q---------------------
RPGNLPDF--------QHHSAGASSHAVPAPCLPLD----QSPNRAASFHGLP--AA--------------------GSSEPHGLE----
QRR-LPAQ-G--GVDSLE------------YNYPG-DGPAG---HFELPVFS-PSE-P--EGQLP-HYGGGRQV--PAGGSYPG-
TPSL--PRAPGM--A-VAKAH--P-----------------------P----------QQH--GVFFERF--GGARKMSTSLEPG--AST-RHPLM--
QQ--------QQ---QQPP-QPQ-------------------QPPGLLARQNSCPPAIPR--QQQTEANAPNPNL-QDNG-PIMQ-NQHAQ--
------FEYPIHRLENRNM--------------HPY--TDP---VFNM----QHP---P-------P----QQPP------NQRLQHFDA-
PYVSVAKRPRFDFPSN-P----G-VERCASWGG-----S-MH-GPAMESHL------------SPS-A-YP------------GLPGEFTP--
PAPEA---F---GGPLPHGGP----EHPAL-----A-------------------------QRQN--------AALVMKQMASRS-------------QQR-
LR-PPS---LQQLGHHGEVG----PP---------SGLPP-P-AFERE-----------------------AGSGR-------------------GFDPQAP-
HLTPDS--AWFAG----PP---PPGELL--------PRRMAAPGLPAEAAPHE---LGL----QP--GGAAVLFRP--G--AGGL-
GLQE-P----LR-MA--GEGPAQALPSP-----GVHPPFAPA----MGGLSQLQSPGGG-----VSLPNAPAER-R---
GPADFAAQ---P-----GFPF-GAAARQ-PAAHGAAPTLSASP-------------------GAY-PPPPPEF-PPPPPP---
RPAASKLGALSLG--------SFSK----------PAS------------KDN-VFGQSCLAALSTACQNMIASLGAP--------
NLNVTFNKKS---P-AEAKRKLSQAEPDP---------------------------PPSAAPDYFP--AGPPAGGSSAG----------KVAGA---
-------APLLPA-ESS--LSPGYA---L---------EP-AASSEGK------AGGGRGR----------------
---GRRKRD----------SGHVSP--------GTFFEKF----------SATEGSGAGVS-P-
GQPAPPAAGAPP------GAAGTER----------------GGGTPH---D-KPLTSP--SW----GKG-----GELL------------LGEQPD-
LMS--SLD--SGIQSV-TKS---DGSSP-----HVDFP-DE-VST--SY-GNEDE------VSSSSDNT---------------------------
VSKPTRSPLLGGSPKLPR------GEHALLN--GQKPLALGLLNT------------------STSTP----DSYGL---STTAG---AHP-
GTPGME-QVRTP---TSTSA------------QDEIHPLEILQAQIQLQRQQFSISEDQPLGLK--------------SKKGECAG------
QNGD--SDLSSCC---S---EGVKGA--------MSTIDLDSLMAEHNS-TWYLPG-------EK-ALMEG----QEEDKP--
MVPWEKPKPPNP-SKEAHDLPPSKTS-------------AAAQTGSHLQCLSVHCTDDVGEAKGRT-
AVPTWRSLHSDISNRFGTFVAALT-----------------------------------

>O.niloticus

-------MFGL-EQFGSQ--------INSRN--------PGQSER------NI-NQQRL----NMGSHYK-GPG-FH----------AG-----G-P--P-
GAVEP-GMGPLSEPQ--M-LG--------LNMNM-N--GEQYGSFH-PR-GHSDMH-------------AGSG-LQQQQG------------
QGP---------------MH----GFFNN---QQPH-QGHPHGHQ-P----HPHQ-HHPHFG-GNFGGPEPGS-SCLHGGRLM----
GYNNNNGM---GP---Q----------QGFGE--GFD-PLAEG-Q-------------------TGDGFPQQQQQQP---------QQ----------------
-----RPGNMPDF--------QHHGPPSGNHAVPAPCLPLD----QSPNRAASFHGLPSSSS---------------------SSSESHGLE----
PRR-LPNQ-G--AVEGLE------------YNFPS-EPPSG---HFDVPVFS-PSE-S--ESQLP-HFGPGRPV--PGG-NFPG-NAGM--
PRTPGM--QGISKGH--P------------------------PPP---QPQQPQH--GVFFERF--GNGRKVPVGMEPG--VNP-RHPLM--
QQ--------QQ------------------------------QAGLIARQNSCPPGLPR--PPQAEPGTTNPNI-LDGG-VMMP-GQHNQ--------
FEYPIHRLENRGL--------------HPY--GDP---MFNM---QQPA-----------PPPS-QQPA------NQRLQHFDS-
PYMNMAKRPRFDFPNA-H----G-GEG---WCG-----------SMDNHL------------SPS-A-YP------------GLPGEFTP--
PVNEG---F-GPGP-LQHPGP----EQQSL-------Q------------------------QRQN--------AAMMIKQMASRN------------QQQR-
MR-QPS---LQQLGHHGDVP----PG-PMVHGGPVGNMPH-P-GFDRE---------------------NGGRMP-------------------
NIDGQNP-HVTQEN--SWFQG----SH---PPGEMM--------SRRMGGAG--NESGPHD---MGL----QQ--NGAGIMFRP-----
--GI-GMQE-P----MR-IP--GDGHVQNLHSP-----GMHSQFSGN----MGNLTQMQSPGAG-----AGHPNAPAER-R----
PADFPAP---SMGAQPAFPY-GGANRQ-GPAHSAPQGVSTSP-------------------GNY--PPQSEF--PPGQR----
SSVSKLGNLSLG--------NFSK----------TSS------------KDS-VFGQSCLAALSTACQNMIASLGAP--------
NLNVTFNKKN---Q-NEGKRKLSQTEQDIN--S-STS-NG----------------TGSAGPEYFQ--SSTSQNSQMPG----
TGNSNSKPASQ---------SQTVQGE-ASA--LSPNYN--M---------DA-TPCSEGK--
ATT--GSGRGR--GRRKRD----------SGHVSP--------GIFFSSD---
-----------NSNPVVS-P-GQQTPSAGVGER-----------------------------GGGTPH---E-KQLQSP--SW----GKG-----GDLM-----
-------LGDQAD-LMS--SLD--SGIQSV-AKS---DSSSP-----RVDFS-ED-VST--HY-GNEDE------VSSSSDTGGAS----------
---------------ASKPNRSPIITGSPKMQR------SDHGLIN--GQKPLGMGINNH------------------TTSTP----
DTYGLNAGVGTGASGVSHP-GTPGVE-QVRTP---SSTSG------------QEEIHPLEILQAQIQLQRQQFSISEDQPLAMK-
-----------TGKKNGDCPS------QNGD--NELASCS---P---DAGKGS--------MGTIDLDTLMAEQHA-TWYVPS-------DK-
VMMDG----SEDDKA--TGPWEKNKSQNS-SKEESELTQSKAG-----
AGAPGAVGGGSSGGNHLQCLSVHCTDELGDSKGRGGPVSSWRSLHSDISNRFGTFVAALT----------------------------
----

>S.bombifrons

-------MFGL-EQFEPQ--------INNRS--------AGQGER------NF-NQPGM----NMSSHFK-NPA-FH----------SG-----G-A--S-
GTVDP-AIGALSDPA--M-LG--------MNLNL-N--GETY-GYH-AR-GHSEMH------------AGGM------Q-----------PQP---

------------VH----GFFNN---QQHH-HGHPSAHP--------HQ-HHPHFS-GSFGGPDATA-SCLHGGRLM----GY--NNNL---
GS---Q----------QAFGE--GYE-QMADN-Q------------------SGEGFG---------------QQ-------------------RSGNISEF-------
QHPNSSASNHAVPAPCLPLD----QSPNRAASFHGVP--SS--------------------TSSDSHNLE----QRR-IHSQ-G--
SVDPLE------------YNFPS-DAPSG---HFEVPVFS-TSD-S--EG----HYGAGRQV--PGG-SFPG-TSAL--PRPPGI--
VGMSKVH--P------------------------------QP---QH--GVFFERF--GGARKMPVGMEPA--VNA-RHPLM--QQ--------QQ---
---------------------------QTGLLSRQNSCPPGITR--QQQTEASTPNPNL-QDNG-PVMQ-NQHAQ--------
FEYPIHRLENRNM--------------HPY--SES---MFNM---QQGP-----------P----QQPS------NQRLQHFDS-
PYMNVTKRPRFDFPNN-H----S-VENCAAWNN-----G-GIHNAGIDSHL------------SPS-A-YP-------------GLPGDYTP--
QVPES---F-PPGPALQHPSS----DHQSL-------Q------------------------QRQN--------AAMMIKQMASRN------------QQQR-
MR-QAN---LQQLGHHGDVN----QS-NIVHGAQVGNIPQ-P-AFDRE----------------------GGRIG-------------------
NFDPQNP-HVAPEN--PWYPG----PH---PPGDIL--------QRRMGGSNLPPDPTPHD---INL----QQ--NGSNMLFRP--G--
VSRM-GIQE-P----LG-MP--GEGHVQALHSP-----GIHSQFG-N----MTNISQMQSPGGG-----VGITSTPTDR-R---
GAPDFTAP---PIGGQPGFPF-VGPNRQ-STPH-NPPGVNSSP-------------------SSY--PAQSDF--QASQR----
STASKLGALSLG--------SFSK----------SNT------------KES-MFGQSCLAALSTACQNMIASLGAP--------
NLNVTFNKKS---Q-AEGKRKMSQTETDIN--S-NGG-N-------------------NTASDYYP--AGSSQNNQVPG----TS--
NTKCTGQ------SGASQPTQGE--TS--LSPNYS--I---------EA-TPGNDGK--PVT--
GGGRGR--GRRKRD----------SGHVSP--------GNYFDKY--------
----SADSGGTVVSP-AQQGQSANSGE-------------------------------AGGTPH---D-KPLTSP--SW----GKG-----GELL---------
---LSDQPD-LMS--SLD--SGIQSV-TKS---DSSSP-----HVDFA-ED-VNT--TY-GNEDE------VSSSSDNN--------------------
--------ISKPSSCPLVTGSPKIQR------GEHGLLN--GQKPMNLNLLNN------------------TTSLP----DSYGL---SSTGS---
GHP-GTPGIE-QVRTP---TSTST------------QDEIHPLEILQAQIQLQRQQFSISEDQPLGMK--------------NKKNDCTA---
---QNVD--SELNSCC---S---DNVKNS--------MSTIDLDSLMAEHNS-TWYMPN-------EK-SLME-----GEEDKS--
ITPWEKSKSQQT-NKEAHDHPQNKTS-------------AAAQNGSHLQCLSVHCTDDIGESKGRT-
PVPTWRSLHSDISNRFGTFVAALT-----------------------------------

>O.anaticus

-------MFGL-DQFEPP--------IHGRP--------AGPGER------NL-HESGL----SMNAHFK-APA-FH----------AG-----G----
PAGPVDPAALGALGDGP--M-LG--------MNM---GMNGEPY-GYHPSR-GHAELH-------------AGAMQPVP-------------
-------------------------GFFGG---QQPH-HGHPGP------------HHPHFG-GGFGGPDPGA-SCLHGGRLL----GYGGGGGQ--
-----------------PAFAE--GYE-PLAEN-P-------------------GGEGFGQQ-------------------------------------
RPGNLPDFQPQQQQQQQHHGSGGSGHAVPAPCLPLD----QSPNRAASFHGLP--AS---------------------GSSDPHSLE--
--PRRISNPG----GVDSLE------------YNYPG-DGPAG---HFDLPVFS-PSE-A--DGQLP-HYGAGRQM--PAG-AFPG-
PNSM--PRAPAM--GGMAKAH----------------------------QQQQPPPPPPQQQHGVFFERF--GAGRKMSVGMETG--
VNA-RHPLMQQQQQAAQQQQQQQQQQAAQQQQQPPP------------------PPGLLSRQNSCPPAIPR--
PQPTDAGPPNPSLQQDPG-PILQ-NQHAQ--------FEYPIHRLENRNM--------------HPY--GDP---VFNL---QHPP----------
------PQPP------NQRLQHYDA-PYLSVAKRPRFDFATN------PAVDRCASWSG-----------AGLDSHL------------SPS-A-
YP-------------GLPGDFTP--PGPEG---F-APGPPLQHPAQ----DPQTLQQQHQQQQQQQHQQHQQQ------------
QRQN--------AALMMKQMASRT-------------QQR-LR-PPT---LPQLGHPGDVG----P----VHGGPPAGLPQ-P-TFERD----
----------GPGPGPGPGGGRLA-------------------GFEAQAS-HLGPES--AWFPG----PP---PPGDLL--------
PRRLGGPPLPADGGPHE---LGL----PPG-GGSGMLFRG----PGVATLGLQE--------PLRLAGEGHVPGLHSP----
AGLHAQFGGG-------LAQLQSPGGG-----VGMPGAPSDR-RPPPPPPDFAGP---ALGGQPAFPF-GPGSRQ-ATPHGT-
PGLSASPGGAGAGAGGAGGAGSGGGGGAY--PPQPDF--PSSQR---AAAASKLGALSLG--------SFSK----------PSS---
---------KDN-LFGQSCLAALSTACQNMIASLGAP--------NLNVTFNKKS---Q-AEGKRKLSQAEAEGPGGGGGPGA--
-------------------GGGSDYFP-------GGGGPG----PAGGLGKAPGP-------ASATAAGPGEGTG--LSPTYS---------------------
--------------------------------LETSPGTDGKALPGG---AGRGR--
GRRKRD----------SGHVSP--------GTFFDKF--------PPGTGTGGGPAVEGGGGGGGPGVSPGQQ-------GGAEPR--------
--------GAPTPH---D-KALTSP--SW----GKA-----GDLL------------LGDQAD-LMS--SLD--SGIQSA-AKS---DGSSP----
RAGDFPDEV------AY-GNEDE------VSSSSDHAPG--------------------------PAKSSRSPPLAGSPKIPR-
GAGGPGDHGLHN--GPKPLGLGLLAAA-----------------STSTP----DSY-------------GHP-
GTPGLEGQVRTPGGGGGGGG---
SGGNGSTPPQDEIHPLEILQAQIQLQRQQFSISEDQPVGLKGGGAGGTGSGPGPGPGGGGKKAADCSAGQNGD--
SELNGCC---S---EAVKSA--------MSTIDLDSLMAEHGS-TWYLPA-------DK-ALMDG----PDDDKA--
LAPWDKSKPQTP-SKEAHDLPQNKTS-------------ASAQPGSHLQCLSVHCTDDVGEAKGRT-
AVPTWRSLHSDISNRFGTFVAALT----------------------------------

>P.nattereri.mn1a

--MNANYN-TSG-FH----------MK-----A-P--S-VAVEP-VLDPLNEPP-
-M-QG--------LNFMP-G--GEPY-GFQ-PH-SHGDML-------------TTGL--QQQHQ-------------------------------LHM--
PPPFNS---QQLN-PDQP---------------SHPYQD---------GVASCLHGDRHI----GF-SGRST---GH---Q----------QMFVS--
EFG-QLTEA-Q-------------------TRECLSQQQ-------------Q-----------------------RLASMPDY--------
HLHGHPNSNHAVPAPCLPLD----QSPNRAASFHGLP---S---------------------SSPESNRLE----HYR-LFPQ-G--
RVGGSQ------------YCFPC-DPLPG---HFDVTGFPTPDS-T--EPRFS-YCEAESQM--AGC-NFPTFTHGA--SRA-LI---
GSSKVN--Q---------------------------------QLPQQ--NTYSERF--GNRAK----LEPG--VSA-RHHLM---A--------QQ------------
-------------------R-GPMTRQNPGSPVLPQ-LYHAPDFVPNSPDM-QNGS-AVVH-AQHGQ--------
MDHPMHRLSNHGM--------------HSF--GEP---MFNV---PQLG----------------PQPP-----HQQHLNSF---
PYLNMAKRQRFDLPNG------SAGESCSTMSS-----S-LHNRPGLENHL------------SPS-A-FP-------------APMGDFTG--
HVIDG---F-PSGLPPLSCGP---HQQQPL-------P-------------------------RRQN--------AAMMIKQMASRN------------QQQR-
TR-QAE---LQTVSHQGDLT----PNA-MVQRGPLGSMSQ-V-NFEKK-----------------------HNFHG--------------------
NFEAQSP-HLPHEN--SWFPD----SH-------------------------QQCRETNIHA---MEH----TQ--NGHNPMFRP----------GVTT-
--------------GDMQSLNSP-----GPHNQFENG----MNNPLQVHLPNEG-----TMQPNTHLDR-R----HHEFGGA---
TMRRQHSFPP-GGPYQQ-GTPQSNPPGFSSSP-------------------GNY--QSHPEY--LSSQH----LSVSKLGALSLG--------
NLNK----------AST------------KDS-VFGQSCLAALSTACQNMIASLGAP--------NLNVTFNKKS---Q-
NEAKRKPGQVEQDVNSSGGGGAC---------------------GPGAEYFQ--SNASQNSQTSC----SGNNNNATAGQ------
SAPGQMAKRE-TST--LSPNNN---I---------DS---GNEGK--AAT--GSGRGR--------------
--GKRRRD----------SGHISP--------GNFSPSC--------------SSNPMVS-P-
GQQTSSLSIGIEG-----------------------------RGRTPD-----SSLVSP--SF----GKP---------------------------D-LAT--SMD--
SGIQSV-GKS---DGVSP-----CMDYL-DD-ASP--HY-SNEDP--RTNRTSMKCNSDN-
RT---GYP-DTPCME-QVRTP---LGSSG------------
QDEVHPLEILQAQIQLQRQQFSISEDQPLGGK-------------TGKEPDCAS-----GLNGD--CALTSGS---P---ESGKGS-----
---VNTIDLDSLMTEQHA-TWYGPS-------SK-ALIKD----HGNGKC--MGFWDRARGQSD-NKEGLG-----------------------
----

>P.nattereri.mn1b

-------MFGL-EQLGPQ--------INCRN--------AGHAEK------NL-SQPRV----TMSSHYK-SPG-FH----------SG-----G-P--P-
GTVEP-GMGPLNEPP--M-LA--------LNMNM-N-GGEQYGGFH-PR-GHSDMH-------------AGGLQQQQQQQ-----------
-QAP---------------MH----GFFNN---QQP--HNHPHGHQ-A----HPHQ-HHPHFG-GNFADPGPGS-SCLHGGRIM----GY--
GSSM---GP---Q----------QGFTE--GFD-PLSDG-Q--------------------SGDGFSQQQPQQQ---------QQ---------------------
RPGSMPDF--------QHHGPSSGNHPVPAPCLPLD----QSPNRAASFHGLPSSSS---------------------SSSESHNLE----SRR-
IPPQ-G--SVEGLD------------YNFPN-EPPSG---HFDVSVFS-PSD-P--DSQLP-HFGGGRQV--PAA-SFPG-NPGL--
SRASGM--QGISKGH--P-------------------------QAPPQQQPPTQH--SVFFDRF--GGGRKIPVGMEP----GA-RHPLM---
Q--------QQ--------------------------------QTGLMGRQNSCPPSHPR--PPQPEAGSTNASM-QEGG-VMMP-GQPNQ--------
FEYPIHRLENRGM--------------HPY--GDP---MFNM---QQQP-----------PPP--QQPP------SQRLQHFDS-
PYLNVAKRPRFDFPNA-H----G-GESCGSWNS-----G-MHNPPGMENHI------------SPS-A-YP-------------GLPGEFTP--
PVSDG---F-PPGPPLQHPGP----EQQSL-------Q-------------------------QRQN--------AAMMIKQMASRS------------QQQR-
MR-QPN---LQQLGHHGDVP----QG-PMVHGGPVGGMPQ-P-GFERD-----------------------GGGRMV--------------------
NFDGQNP-HMAPDS--GWFPG----PH---PPGEML--------GRRMGGPG--GEAGTHD---M------QQ--NGAAMMFRQ--
G--VNGL-GMSD-P----MR-IP--GEGHVQPLHSP-----SIHPQFGSS----MGNLAQMQSPGAG-----VGLPNTPSER-R----
PNDFSGP---PMGAQPSFPF-GGPSRQ-GAPHNNAQGVSTSP-------------------GSY--TSQSEF--PAGQR----
SSVSKLGSLSLG--------NFNK----------TSS------------KDS-VFGQSCLAALSTACQNMIASLGAP--------
NLNVTFNKKS---Q-GEGKRKLSQTEQDVN--N-STA-NG-----------------TGSAGPEFFP-GGVNPQSTQMPG----
AGNSNTKPTGT---------NQTVQGE-ASA--LSPSYN---M---------DA-TPCSEGK--
AAT--GSGRGR---GRRKRD----------SGHVSP--------GIFFPSD---
-----------NGNPVVS-P-GQQVTPTAGTGER-----------------------------GGGTPH---E-KPLTSP--SW----GKG-----GDLM----
--------LGDQAD-LMS--SLD--SGIQSV-SKS---ADCSP-----RVDFP-DD-IGP--HF-GNEDE------VSSSSDAPT--------------
-------------SAKAGRSPLLGGSPKLQR------VDNGLMA--GQKGQGIGLANH------------------TTSTS----EGYG-------
AV---GHP-GTPGME-QVRTP---SSTSG------------QDEIHPLEILQAQIQLQRQQFSISEDQPLAIK------------
NGKKGSDCNG------QSGD--GELASCS---P---DAGKGS--------VGTIDLDTLMAEQHA-TWYVPS-------DK-SLLEN---
-SEEEKT--LTAWEKTKGQGP-IKEDVDLSQNKSG-----
GGGGGGTPGPGGAGPHLQCLSVHCTDELGDPKGRSGPVPSWRSLHSDISNRFGTFVAALT-----------------------------
----

>C.elaphus

-------MFGL-DQFEPQ--------INSRN--------AGQGER------NF-NEAGLS----MNAHFK-APA-FH----------AG-----G-P--P-GPVDP-AMSGLGEPP--I-LG--------MNM-------EPYG-FH-AR-SHSELH------------AGGL------Q-----------AQP--------------VH----GFFGG---QQPH-HGHPGGHH-P------HQ-HHPHFG-GNFGGPDPGA-SCLHGGRLL----GY--GGAA---GGLGSQ----------PPFAE--GYD-HMAES-Q-------------------GPESFG------P---------Q---------------------RPGNLPDF----------HSSGASGHAVPAPCLPLD----QSPNRAASFHGLP--AS---------------------GGSDSHSLE----PRR-VANQ-G--AVDSLE------------YNYPG-EPPSG---HFDM--FS-PSD-S--EGQLP-HYAAGRQV--P-GGSFPG-ASAL--PRAAGM--VGLSKMH--A--------------------QQQQQQQPQQQQQQH--GVFFERF--GGTRKMPVGLEPG--VGS-RHPLM--QP--------PQQAPPPPQQPPA--QQPPP-PPQQPP---PPPPPGLLVRQNSCPPALPR--PQQGEAGTPSSGL-QDGG-PMLP-NQHAQ--------FEYPIHRLENRSM--------------HPY--SEP---VFNM----------------------QQAP------NQRLQHFDAPPYMNVAKRPRFDFPGS-A----G-VDRCASWNG-----S-MH-NGALDNHL-----------SPS-A-YS------------GLPGEFTP--PVPDS---F-PSGPPLQHPAP----DHPSL---------------------QQQQ------QRQN--------AALMIKQMASRN------------QQQR-LR-QPN---LPQLGHPGDVG----QG-GLVHSGPVGGLAQ-P-NFERD---------------------SGGAGA---------------GRLGTFEPQAQ-HLAQES--AWFPGPH---P---PPGDLL--------PRRMGGSGLPSDCGPHD---PGLAPPPPP--GGSGVLFRS--S--------LQE-P----LR-MP--GEGHVPGLPSP-----GL--QFGGS----LASLGQLQSPGAG-----VGLPSAPSER-R--PQPPDFTAP---ALGGQPGFPF-GAANRQ-ATPHSG-PGVNSPPSTGGGGGS-TGGGGGGGGGGSAY--QPQPDF--QPSQR----TSASKLGALSLG--------SFNK----------PSS------------KDN-LFGQSCLAALSTACQNMIASLGAP--------NLNVTFNKKN---P-PEGKRKLSQNETDGA------------------------AVAGNPGSDYFP--GGTAPGAPGPGGPSGTSSSGSKASGP--------PNPPAQGD-GTS--LSPNYT---------------------------------------L---------ES-TSGNDGK---PVPGGGGRGR--GRRKRD----------SGHVSP--------GTFFDKY------SAAAAPDSGGAPGVS-P-GQQQAPGAAVGG-------NSGEAR----------------GAPTPH---E-KALTSP--SW----GKG-----AELL-----------LGDQPD-LMA--SLD--GG-----AKS---DGSSP----HVGEFASDE-VST--SY-ANEDE------VSSSSDNPPA-------------------------LAKASRSPLVTGSPKLPP-RGVGAGEHGPKA--PPPPLGLGILST------------------STSTP----DSY--------GGGGTGHP-GTPGLE-QVRTPTSSGGAPP------------PDEIHPLEILQAQIQLQRQQFSISEDQPLGLK-------------GGKKGECAV-GASGAQNGD--SELGSCC---S---EAVKSA--------MSTIDLDSLMAEHSA-AWYMPA-------DK-SLVDG----TDEDKT--LAPWEKAKPQNPNSKEAHDLPANKAL--------------APQPGSHLQCLSVHCTDDVGDAKARA-SVPTWRSLHSDISNRFGTFVAALT----------------------------------

>E.calabaricus

-------MFGL-EQFEPQ--------IGSRN--------VAQGER------SF-SQPGL----NMSAHYK-SPA-FH----------PA-----G-P--SVAAVEA-GMGGLNEPP--M-LG--------LGMNL-N--GDQY-SFH-AR-GHSDMH------------TGGL------QQPPPPQQQ----PPP---------------MH----GFFNG---QQPH-HGHQHGHH-P----HAHQ-HHPHFG-GSFNGADSGGTSCLHGGRLM----GY-NNGGL---GP---Q----------QNFAE--GFE-PMADN-P-----------------G-AGEGFG----------------QP--------------------RPNNMSEF--------QHHNPQASGHSVPAPCLPLD----QSPNRAASFHGLP--SN--------------------SSSEAHGLE----QRR-MPAQ-A--GVDSLE------------YSYPA-DNPSG---HFDMPVFS-PSD-S--DTQLP-HYGAGRQV--P-S-NFSG-SPVM--PRAPGM--AGISKVH----------------------------------TP---QH--GMFFERY--GNGRKMSVGMEPG--VNS-RHPLM--QQQQPPPPPPPQ-------------------------------QASLLARQNSCPPAIPR--QPQVEPAAANPNL-QENG-VIMP-GQHNQ--------FEYPIHRLENRSM--------------HPY--SDP---MFNMQQQQQQP-----------PPPSPQQPP------NQRLQHFDG-PYLNMAKRPRFDFPSN-H----N-ADNCSTWN------N-MHNPAGMENHL------------SPS-A-YS-------------GLPGEFTP--PVPES---F-SQGPPLQHTGS----EQQSL-------Q-----------------------QRQN--------AAMMIKQMASRN------------QQQR-MR-QPS---LQQLGHHGDVT----QS-SMVHGGQVGSLPQ-P-NFDRE----------------------GGGRMA-------------------AFDSQNP-HMPPEN--TWFPG----PH---PPGEML--------PRRMGSSGVPGEASPHE---MGI----QQ--NGSNMLFRS--A--VNGM-GMQE-P----MR-MP--GDGHVQGLHSP-----GMHSQFGPN----MGGLSQMQSPGTG-----VGLPGAASDR-R----PSDFPAP---PAG----FSF-GGANRPAAAPHSNPSGVSASP-------------------GNY--PPQSDF--QPGQR----PSVSKLGALSLG--------SFSK----------TTA------------KDN-VFGQSCLAALSTACQNMIASLGAP--------NLNVTFNKKS---Q-NEGKRKLSQTEPDGS--SASAPGGA----------------NGGAGPEYFQ--PSAPPNSQMAG----TG-SGAKPAGP------GGPSQPAPGE--AN--LSPNYT--M---------DA-ATGNDGK--PPT--GSGRGR---GRRKRD----------SGHVSP--------GIFFDKF-----------SADSGNPGVS-P---GQQGPS------PLTSP--SW----GKG-----SDLL------------MGDQQD-LMS--SLD--SGIQSV-TKS---DTSSP-----HVDFP-DD-VST--HY-GNEDE------VSSSSDNV--------------------------TSKPSRSPLVTGSPKMQR------GDHGLIG--GQKPMGLGMLNN------------------STSTA----DSYGL---SSTGA---GHP-GTPGME-QVRTP---SSTST------------QDEIHPLEILQAQIQLQRQQFSISEDQPLAMK--------------NKKAECPA------QNGD--NELGNCG---T---DGGKNA--------MSTIDLDSLMAEQHA-TWYIPN-------DK-ALMEG----QEEEKA--MAPWEKTKPPNN-SKEVPEHQNKTPA--------------AGQNGSHLQCLSVHCTDDIGEAKART-PVPTWRSLHSDISNRFGTFVAALT----------------------------------

>S.canicula

-------MYGL-DQFSRS---------GGQG-----------GER------NF-GQ-TAL---GMSGHYK-SPA-FP----------GG-----S-G--N-ALEEQ-ALNPLAEPP--M-LG--------MGMSP-ALGGEQY-GFH-PR-GHSELH------------GAGAGAMQQQQQQPAPPQPQPQPQPPPQPQA-----QP-PPAVH----GYFPNGPHPHPQQH-HPHA-H------------H--FS-GTFCGTEPGP-SCLHGGRLLGT-PGYSSNPLS---GQ---Q----------QAFGE--SYE-PLAEN-Q---------------GGGAGGEAFSQQQQQQ---------QQPP-----------------PGRSGTLTDY--------HQHHTPSSNH---TPCLPLD----QSPNRAASFHGLP--SS---------------------SSSEAHGLE----QRR-LQNQ-P--AVDSME------------YNYQS-DAPSG---PFDIPVFS-PSE-S--SAQLP-HYGTSRHV--PSS-NFPA-NPAM--PRAPGM--VSLGKVH--P------------------------------QQ---QH--GVFYERF--GNARKMSVGMEPG--VAA-RNPLM------------QQ------------------------------QAGLLARQNSCPPAIPR--QQQAEAGAPNSSL-QDNG-TMMQ-SQHAP--------FEYPIQRLENRNM--------------HPY--SEP---MFNI-----------------------QQQP------NQRLQHFDA-PYLNVAKRPRFDFPNS-H----N-VDNCATWSS-----S-NMHNASLENHL------------SPS-A-YP-------------GLPNEFSP--PGPEG---F-PPGPPLQHPGT----DQQSL-------Q-----------------------QRQN--------MLMMFKQMVSRN------------QRHR-MR-QSD---LQHLGHHGDVN----QN-NIVHSNQVGTMPQ-P-NFERE----------------------SGGRMP-------------------TFDPQNP-QIGQEN--AWFPG----HH---PPGDML--------QRRMGGSAVPADGSPHDSVQMNL----QQ--NGSGMLFRP--G--GNGL-GMQE-P----MR-MP--GDGHVQGLHSP-----GMHSQFGNN----MGNVTQMQSPSGG-----MGLPNATTDR-R---PGPDFPGP---QMGGQPGFPF-GGPNRPA-NPHNNPPGVPPSP-------------------GNY--PPQSEF--QPNQR----PSMSKIGSLSLG--------SFSK----------PGS------------KDNTIYGQSCLAALSTACQNMIASLGAP--------NLNVTFNKKN---Q-NEGKRKLSQTDQDSSNGGPNSGSGGSGG-----------GASTGNGPEYFQ--SNTPQNNPMGV----SGNGSGKLATPAAQNTTQGPNQPSQPE--CN--LSPNYA---L---------EA-IP-SEGK--GQT----GRGR---GRRKRD----------SGHVSP--------GNFFDKY-----------SAENVNPGVS-P-GQQGQSTHAGD------------------------------RGGTPH---D-KSLTSP--SW----AKG-----NDLL------------LPDQPD-LMS--SLD--SGIQSV-TKS---DGSSP-----QVDFS-DD-VGN--NY-GNEDE------VSSSSDNN---------------------------ISKPSR--LVTSSPKLQR------ADHGLMS--SQKPMGHSMLNAHPNSN-------------TTSNTGPVTDSFGL---SSTGS---GHP-GTPGME-QVRTP---TSTSA------------QDEIHPLEILQAQIQLQRQQFSISEDQPLAMK--------------NKKAECPG------QNGD--TELAACA---T---DNSKAA--------ISTIDIESLMAEHNS-TWYMPN-------DK-AMMDS----QDEDKQ--MAPWEKAKSANT-NKEAHELSQNKAS--------------SVQTGSHLQCLSVHCTDDLTDSKGRS-PMQTWRSLHSDISNRFGTFVAALT----------------------------------

>P.pectinata

-------MFGL-NQFSRN---------GGQG-----------AER------NF-GQ-AAL---GMSGHYK-GPG-FP----------GG-----G-G--S-AMGEQ-GISALTEPP--M-LG--------MGMNP-ALGGEQY-AFH-TR-GHSELH------------GAG-----AMPQPPPPPQPQPPPPPPPPQPQA-----QPQPSAAH----GYFPNGHHHH-----HQHAHH------------H--FS-GSFCGSEPGP-SCLHGGRLLGA-PGYNGNPLS---GQ---Q----------PAFGE--TYD-PMAEN-Q----------------GGGGGEAFG---QQQQ---------QQPA-----------------PGRSANLTDY--------HQHHTPSSNH---TPCLPLD----QSPNRAASFHGLP--SS---------------------SSSETHGLE----QRR-LQTQ-P--PVDSMD------------YNYQS-DP------PFDMPVFS-PSE-S--NAQLP-HYGAGRQV--PSS-NFPG-SPAM--PRTPGM--VNMGKVH--P------------------------------AQ---QH--GVFYERF--GSARKMSVGMEPG--VAA-RNPLM------------QQ------------------------------QAGLLARQNSCPPAIPR--QQQAEAGAPNSNV-QDNG-PMIQ-NQHAP--------FEYPIQRLENRNM--------------HPY--SEP---MFNI-----------------------QHQA------NQRLQHFDA-PYLNVAKRPRFDFANN-H----S-VDNCATWSG-----N-SLHNASLENHL------------SPS-A-YP-------------GLPSEFSP--PGPEG---F-PHGPPLQHPGT----DQQSL-------Q------------------------QRQN--------MLMMFKQMVSRN------------QRHR-MR-QSD---LQHLGHHGDVN----QS-SMAHGNQVGTMSQ-P-NFERE----------------------SSGRMS-------------------NFDPQNP-PMAQEN--SWFAG----PH---PPGDLL--------QRRIGGSAVATEGSPHESVQMNL----QQ--NGSSMLFRT--G--GNGL-GMQE-P----MR-MP--GEGHVQALHSP-----GMHSQFGSN----MGNVSQMQSPSAS-----IGLPNPSSDR-R---AGPDFPGP---QIGGQPGFPF-GGPSRSA-NPHNNPAGVPPSP-------------------GSY--PPQNEF--QPNQR----PSMSKIGSLSLG--------SFSK----------PGA------------KDNTIYGQSCLAALSTACQNMIASLGAP--------NLNVTFNKKN---Q-NEGKRKLSQTEQDGGAGGPSSGGGAGVGSV-----GGASTGSTGNGQEYFQ--SNTPQSNQMGG----SGNGSGKLAASAAQNCTQGQSQPTQPE--CN--LSPNYG---L---------EA-VP-SDGK--GQT----GRGR---GRRKRD----------SGHVSP--------GNFFDKY-----------SAENVNPVVS-P-GQQGQSTHAGD------------------------------RGGTPQ---D-KSLTSP--SW----AKG-----NDLL------------LPDQPD-LMS--SLD--SGIQSV-TKS---DGSSP-----QVDFS-DD-VSN--NY-ANEDE------VSSSSDNN------------------------------ISKSTRLMTSSPKLQR------VDHGLKQ------MGHAMLNAHPNSN-------------TTSNAGAVTDSFGL---SSSGS---GHP-GTPGIE-QVRTP---TSTST------------QDEIHPLEILQAQIQLQRQQFSISEDQPLAMK--------------NKKAECPG------QNGD--TELAACG---T---DNSKAA--------ISTIDIESLMAEHNS-TWYMPS-------DK-AMMDP----QDEDKQ--MAPWEKAKPAST-NKEAHELSQNKAS--------------SVQAGTHLQCLSVHCTDDLTDSKGRS-PMQTWRSLHSDISNRFGTFVAALT----------------------------------

>L.oculatus

-------MFVL-EQFEPQ--------INSRN--------AGQGER------NL-NQPRL----NMSSHYK-SPA-FH----------AA-----G-P--P-
GAVEH-AMGALNEPP--I-LG--------LNMNL-N--GEQY-SFH-PR-GHSEMH-------------AGGL------QP-----------PPP-----
----------MH----GFFNN---QQPH-HGHPHGHH-P----HAHQ-HHPHFG-GNFSSPDPTG-SCLHGGRLM----GY-NGSAI---
GP---Q----------QNFAE--GFE-PIAEN-Q-------------------TGEGFG-----QQ---------QQ--------------------RSSNMPEF-------
-QHHSAQNSSHAVPAPCLPLD----QSPNRAASFHGLP--SS---------------------SSSDAHSLE----QRR-MPPQ-G--
GGENLE------------YSYPS-ETPSG---HFDVPGFS-PSE-S--ESQHP-HYGPGRQV--AGS-SFPA-NPAM--PRAPGM--
PGIAKVH--H------------------------------QQ---QH--GVFFERF--GNGRKMSVGMEPG--VNA-RHPLM--QQ--------QQ----
---------------------------QAGLLARQNSCPPALPR--PPQSESSTANPNM-QENG-VMMP-GQHNQ--------
FEYPIHRLENRNM--------------HPY--GDP---MFNM---QQQP-----------P----QQPP------NQRLQHFDS-
PYLNVAKRPRFDFPNN-H----N-AENCGTWNS-----N-LHNPPGMENHL------------SPS-A-YP-------------GLPGEFTP--
PVPEG---F-PPGPPLQHAGP----DQQSM-------Q------------------------QRQN--------AAMMIKQMASRN------------
QQQR-MR-QPN---LQQLGHHGDVS----QS-SMVHGGQVGNMPQ-P-NFERE-----------------------GGRMA----------------
---SFDSQNP-HMPQEN--TWFPG----PH---QPGEML--------PRRMGGSSVPGEASPHE---MGL----QQ--NGSNMLFRP--
G--VNGM-GMQE-P----MR-MP--GDGHVQALHSP-----SMHSQFGNN----MGGLSQMQSPGAG-----VGLPNAPSDR-
R----APDFPAP---PMGAQPSFPF-GGSNRQ-GPPHSSTPGVSASP-------------------GNY--PPQPEF--PPGQR----
SSVSKLGALSLG--------SFSK----------SSS------------KDN-MFGQSCLAALSTACQNMIASLGAP--------
NLNVTFNKKS---Q-NEGKRKLSQTEQDIT--SGSTSGNG----------------SS----EYFQ--SNAAQSGQMPG----
TGNSNSKPTGQ------SGTSQPVQGDTTTT--LSPNYN--M---------DA-TPGSEGK--
PTT--GSGRGR---GRRKRD----------SGHVSP--------GIFFSSD---
-----------SGNPVVS-P-GQQGPPASAGER-----------------------------GGGTPH---E-KPLTSP--SW----GKG-----GDLL-------
-----LNDQPD-LMS--SLD--SGIQSV-TKS---DGGSP-----HVDFP-DD-VST--SYGGNEDE------VSSSSDNN----------------
------------VSKPNRSPLVTSSPKIQR------GDHGLMN--GQKQMSLSILNN------------------TTSTP----DSYGL---SSSGG-
--GHP-STPGME-QVRTP---SSTST------------QDEIHPLEILQAQIQLQRQQFSISEDQPLAMK--------------NKKADCSS-
-----QNGD--NELANCG---T---DSNKNT--------MSTIDLDSLMAEQHA-TWYIPN-------DK-SLIEG----QEDDKP--
MAPWEKTKPQNN-NKEAPDLPPNKSS-------------STGPNGSHLQCLSVHCTDDIGEAKSRG-
PVPSWRSLHSDISNRFGTFVAALT----------------------------------

>A.ruthenus.Chr11

-------MFGL-EQFEHQ--------INSRN--------VGHGER------NF-NQPGL----NMSAHYK-NPT-FH----------PS-----G-P--P-
GAVEQ-GMGTLNEPP--M-LG--------LNMNL-N--GEQY-GFH-PR-GHSEIH-------------AGGL------Q------------QQP----
-----------MH----GFFNN---QQPH-HGHPHGHH-P----HAHQ-HHPHFG-GNFSGPDPSA-SCLHGGRLM----GY-NSSSM-
--GP---Q----------QGFAE--GFD-PMAEN-Q-----------------GAGGEGFG----------------HQ--------------------RPNNMPEF---
-----QHHNSQATNHAVPAPCLPLD----QSPNRAASFHGVP--ST--------------------TSSDAHSLE----QRR-MPAQ-A--
GVETLE------------YSYPS-ETPSG---HFDMPVFS-PSE-S--DAQLP-HYGAGRQV--PGG-NFPG-NPAM--PRAPGM--
PGISKVH--H------------------------------QQ---QH--GVFFERF--GNTRKMSVGMEPG--INA-RHPLM--QQ--------QQ------
-------------------------QGGLLARQNSCPPAIPR--QPPTEASAPNPNL-QDNG-VMMP-GQHNQ--------
FEYPIHRLENRSM--------------HPY--SDS---MFNM---QQQP-----------P----QHPP------NQRLQHFDT-
PYLNVAKRPRFDFPNN-H----N-SENCATWNS-----N-MHNPPGMENHL------------SPS-A-YP-------------GLPGEFTP--
PVPEG---F-PPGPPLQHAGP----DQQSL-------Q------------------------QRQN--------AAMMIKQMASRN------------QQQR-
MR-QPS---LQQLGHHGDVS----QN-SMVHGGQVGNMPQ-P-NFERE----------------------SGGRMA-------------------
NFDSQNP-HMAQEN--TWFPG----PH---PPGEML--------QRRMGTSNVPGEASPHE---MNM----QQ--NGSNMLFRP--
G--VNGM-GMQE-P----MR-MP--GDGHVQALHSP-----GM--QFGNN----MGNLSQMQSPGAG-----VGLPNAPSDR-R-
---PPDFPTP---PMGAQSGFPF-SGANRP-GAPHNNPPGVNASP-------------------GNY--PPQSEF--QQGQR----
SSVSKLGALSLG--------SFSK----------TNA------------KDN-MFGQSCLAALSTACQNMIASLGAP--------
NLNVTFNKKS---Q-NEGKRKLSQTEQDIN--SMNAGATG----------------NSSSGPEYFQ--SGASQNSQMPG----
AGNSNSKPSGQ------SGTSQPTQGE--AN--LSPNYN--M---------DA-TPANEGK--
SVTGSGSGRGR---GRRKRD----------SGHVSP--------GIFFDKF-
-----------SSDCGNPVVSP-GQQGPPTSAVER-----------------------------GGGTPH---D-KPLTSP--SW----GKG-----SDLL--
----------LSDQPD-LMS--SLD--SGIQSV-TKS---DSSSP-----HVDFP-DD-VST--NY-GNEDE------VSSSSDNN-------------
---------------ISKPNRSPLVTSSPKIQR------GDHGLLN--GQKQMGLGMLNN------------------TTSTP----DSYGL---
SSTGG---GHP-GTPGME-QVRTP---SSTST------------QDEIHPLEILQAQIQLQRQQFSISEDQPLAMK--------------
NKKPDCPS------QNGD--NELVNCV---T---DSGKNA--------MSTIDLDSLMAEQHA-TWYIPN-------DK-SLMEG----
QEDDKS--MAPWEKTKPQNN-NKEAQDLPQNKPS-------------AAGQNGSHVQCLSVHCNEDMGESKGRT-
PVPPWRSLHSDISNRFGTFVAALT----------------------------------

>A.ruthenus.Chr21

-------MFGL-EQFEHQ--------INSRN--------VGHGER------NF-NQPGL----NMSAHYK-NPT-FH----------PS-----G-P--P-GAVEQ-GMGTLNEPP--M-LG--------LNMNL-N--GEQY-GFH-PR-GHNEIH-------------AGGL------Q------------QQP---------------MH----SFFNN---QQPH-HGHPHGHH-P----HGHQ-HHPHFG-GNFSGPDPSA-SCLHGGRLM----GY-NSSSM---GP---Q----------QGFAE--GFD-PMAEN-Q-----------------GAGGEGFG----------------HQ-------------------RPSNMPEF--------QHHNSQATNHAVPAPCLPLD----QSPNRAASFHGVP--ST---------------------TSSDAHSLE----QRR-MPAQ-A--GVETLE------------YSYPS-ETPSG---HFDMPVFS-PSE-S--DAQLP-HYGAGRQV--PGG-NFPG-NPAM--PRAPGM--PGISKVH--H------------------------------QQ---QH--GVFFERF--GNTRKMSVGMEPG--INA-RHPLM---Q--------QQ-------------------------------QGGLLARQNSCPPAIPR--QPPTEASAPNPNL-QDNG-VMMP-GQHNQ--------FEYPIHRLENRSM--------------HPY--SDP---MFNM---QQQP-----------P----QHPP------NQRLQHFDT-PYLNVAKRPRFDFPNN-H----N-PENCATWNS-----N-MHNPPGMENHL------------SPS-A-YP-------------GLPGEFTP--PVPEG---F-PPGPPLQHAGP----DQQSL-------Q-----------------------QRQN--------AAMMIKQMASRN------------QQQR-MR-QPS---LQQLGHHGDVS----QN-SMVHGGQVGNMPQ-P-NFERE----------------------SGGRMA-------------------NFDSQNP-HMAQEN--TWFPG----PH---PPGEML--------QRRMGTSNVPGEASPHE---INM----QQ--NGSNMLFRP--G--VNGM-GMQE-P----MR-MP--GDGHVQALHSP-----GM--QFGNN----MGNLSQMQSPGAG-----VGLPNAPLDR-R----PPDFPTP---PMGAQSGFPF-SGANRP-GAPHNNPPGVNASP-------------------GNY--PPQSEF--QQGQR----SSVSKLGALSLG--------SFSK----------TNA------------KDN-MFGQSCLAALSTACQNMIASLGAP--------NLNVTFNKKS---Q-NEGKRKLSQTEQDIN--STNAGATG----------------NSSSGPEYFQ--SGASQNSQMPG----AGNSNSKPSGQ------SGTNQPTQGE--AN--LSPNYN--M---------DA-TPANEGK--SVTGSGSGRGR---GRRKRD----------SGHVSP--------GIFFDKF------------SSDCGNPVVSP-GQQGPPTSAVER-----------------------------GGGTPH---D-KPLTSP--SW----GKG-----SDLL------------LSDQPD-LMS--SLD--SGIQSV-TKS---DSSSP-----HVDFP-DD-VST--NY-GNEDE------VSSSSDNN----------------------------ISKPNRSPLVTSSPKIQR------GDHGLLN--GQKQMGLGMLNN------------------TTSTP----DSYGL---SSTGG---GHP-GTPGME-QVRTP---SSTST------------QDEIHPLEILQAQIQLQRQQFSISEDQPLAMK--------------NKKPDCPS------QNGD--NELVNCV---T---DGGKNA--------MSTIDLDSLMAEQHA-TWYIPN-------DK-SLMEG----QEDDKS--MAPWEKTKPQNN-NKEAQDLPQNKPS-------------AAGQNGSHVQCLSVHCNEDMGESKGRT-PVPPWRSLHSDISNRFGTFVAALT----------------------------------

>G.aculeatus

-------MFGL-EQFASQ--------INSRN--------PRQSER------NI-NLSRL----NMGSHYK-SPG-FH----------AG-----G-P--P-GAVEP-GMGSLSEPQ--M-LG--------LNMNM-N--GEQYGGFH-PR-GHSDMH-------------AGSG-LQQQQQ------------QGP---------------MH----GFFNN---QQPH-QGHPHGHQ-P----HPHQ-PHPHFG-GNFGGPEPGS-SCLHGGRLM----GY-NNNGM---GP---Q----------QGFGE--GFD-PLAEA-Q-------------------AGDGFPPQQQQQQ---------QQ--------------------RPGNMPDF--------QHHGPPSVSHAVPAPCLPLD----QSPNRAASFHGLPSSSS--------------------SSSESHGLE----SRR-MPNQ-G--AVEGLE------------YNFPS-EPPSG---HFDVHVFS-PSE-S--ESQIP-HFGPGRPV--PSA-NFPG-NPGM--PRTSGM--PGIPKGH--Q------------------------PPPQPQQPQQPQH--GVFFERF--GNGRKVPVGIEPG--VNA-RHSLM-QQQ--------QQ--------------------------------QAGLIARQNSCPPGLPR--PPQAEPGATNPNI-LDGG-VMMP-GPHNQ--------FEYPIHRLENRGM--------------HPY--GDP---MFNM---QQPA-----------PPPS-QQPP------NQRLQHFDS-PYMNMAKRPRFDFPNA-H----G-GEG---WCG------------GMDNHL------------SPS-A-YA-------------GLPGEFTP--PVSEG---F-PPGP-LQHPGP----EQQSL-------Q-----------------------QRQN--------AAMMIKQMASRN------------QQQR-MR-QPS---LQQLGHHGDVP----PG-PMTHGGPVGNMPQ-P-GFDRE----------------------NAGRMP-------------------NIDGQNP-HVTQEN--SWFQG----NH---PPGEMM--------SRRMGGAG--NESGPHD---MGL----QQ--NGAGMIFRP-------GM-GMQE-P----MR-IP--GDGHVQALHSP-----GMHSQFSGN----MGNLSQMQSPGAG-----TGHPNAPAER-R----AAEFPAP---PMGAQPTFPY-GGANRQ-GPAHSAPQGVNTSP-------------------ASY--PSQSEF--PPGQR----PSVSKLGALSLG--------NFSK----------TSA------------KDS-VFGQSCLAALSTACQNMIASLGAP--------NLNVTFNKKN---Q-NEGKRKLSQTEQDIN--S-STS-NG----------------TGSAGPEYFQ--SSTSQNSQMPG----TGNSNSKPTSQ---------SQTVQGE-ASA--LSPNYN--M---------DA-TPCSEGK--ATT--GSGRGR---GRRKRD----------SGHVSP--------GIFFSPD--------------NGNPVVS-P-GQQTPSAGVGER-----------------------------GGGTPH---E-KHLQSP--SW----GKG-----GDLM------------LGDQAD-LMS--SLD--SGIQSV-AKS---ESSSP-----RVDFP-DD-VST--HY-GNEDE------VSSSSDAGGAS-------------------------ASKSNRSPMITGSPKMQR------SDHGLMN--GQKPLGMGINNH------------------TTSPP----DSYGLNAGGGTGASGVSHP-GTPGVE-QVRTP---SSTSG------------QEEIHPLEILQAQIQLQRQQFSISEDQPLAMK------------NGKKNGDCPS------QNGD--NELAGCS---P---DAGKGS--------MGTIDLDTLMAEQHA-TWYVPS-------DK-AMMDG----SEDDKA--MGPWEKNKGQNS-SKEESELSQSKVGAAAPGAGGGGGGGGGSSGGNHLQCLSVHCTDELGDSKGRGGPVSSWRSLHSDISNRFGTFVAALT----------------------------------

>A.radiata

-------MFGL-NQFSRS---------SGQG-----------AER------NF-GQ-AAL---GMSTHYK-GPG-FPGSGGAGGGGGGG-----G-
G--S-SMGEQ-GISGLNEPP--M-LS--------MGMSP-GLGGDQY-PFH-PR-GHSELH---------------------------
QQPPPPPPPPQPQS-----QAPAPAAH----VYYPNGHQHH-----HPHAHP------------HHQYS-GAFCGSEPSP-
SCLHGGRLLAA-PGYASNPLS---SQ---T----------PGFGE--SYD-AMGEN-A----------------GGGGGEAFGQQQQQQQ--
-------Q--PPSQQQQQQQQHQQAPAGTARAGTLTDY--------HQHHTPASSH---TPCLPLD----QSPNRAASFHALP--
SS---------------------APSEAPGLE----QRR-LHTQ-P--PVDSMD------------YNYQG-DP------HFDMPVFS-PSE-A--
GAQLP-HYGSGRQV--PGGSSFPG-SPAV--PRTTGM--VNMGKVH--P------------------------------PQ---QQ--
GIFYERFGGGNARKMPVGLEAG--AGGGRNSLL--QQ--------QQ-------------------------------
QAGLLGRQNSCPPAIPR--QEAGAATPSGS---QDSG-EMMQ-PQHAP--------FEYPIQRLENRNL--------------HPY--
SEP---MFNV-----------------------QQQP------NQRLQHFDA-PYLNVAKRARFDFANX-P----R-RGNCTNWSG-----N-
GLHNPALDSHL------------SPS-ATYP-------------GLPAEFSP--PGPEG---F-QPGPPLQHPGS----EQ----------Q-------------
-----------HRHN--------MLMMFKQMVSRN------------QRHR-MR-QPD---LQPMGHHGDAG----PS-
GLVHGGQVGGMVQ-G-SFERDGVGGGSGGGGGGGGGGGGGGGGGGRMP-------------------GFDAQNP-
AMAPES--SWFPG----PH---PPADLL--------PRRMGGGG--GERSPHESVQMNL----QQ--NGSNLLFRT--A--GNGL-
ALQE-P----MR-MP--SEGHVQGLHSP-----GIHAQFGGP----MGGLPQLQSPGPS-----IGLPNASGDR-R--
AAAADFPGP---QMGGQAGFPF-AGPSRPPNNPHNNPPGVPPSP-------------------GGY--PGQPDF--QAGQR----
PSMSKIGSLSLG--------SFSK----------PGA------------KDNTMYGQSCLAALSTACQNMIASLGAP--------
NLNVTFNKKS---QGGDGKRKLSQTEQEGV--GPVSGGGAAAGAGAGA--GGTSSGGAGNGPEYFQ--
SNAPQNNQMGV----GGGG-GKLAAQ-----TQGP--AAQPE--CN--LSPNYG---M-------
--EA-AP-VEAK--GQT----GRGR--GRRKRD----------SGHVSP--
------GNFFDKY-----------SAENVNPVVS-P-GPQCQPTLAGER-----------------------------GGGTPQ---D-KGLTSP--SW---
-AKG-----SDLL------------LPDQPD-LMS--SLD--SGIQSV-AKS---DGSSP-----QVDFP-DE-VGP--NY-GNEDE------
VSSSSDNN------------------------------LSKPGQLMSSSPKLPR-----------VEQHGLKPMGHSMLTTN-----------------
TTSNPGPGADSF-------------GHP-GTPGIE-QVRTP--TSTSSS------------
QDEIHPLEILQAQIQLQRQQFSISEDQPLAMK--------------NKKGECPA------QNGD--TELAACG---T---DNGKAA-----
---ISTIDIESLMAEHNS-TWYMPS-------DK-AMMDP----QEDDKQ--MAPWDKVKNSTT-NKEAHEPSQNKAP--------
------SVSADSHLQCLSVHCTDDLTDSKGRS-PMQTWRSLHSDISNRFGTFVAALT-----------------------------------

>C.tigris

-------MFAL-DPFDPH--GGGGGGGSGRS--------GVPDER----GGGF-GPG-------AGPQFK-PSP-FH----------GGSG---G-
G----GSGESAPVNALGEPSSTL-LA--------MNL---TLSGEGYGGFHAPRGGPPELG----------------------Q------------
PQP---------------IH----GFFGS---PPAG-PGAHHQTQ-P---------PPSHFG-GGF-GAEPSA-SCLHGGRLL----GY--SGG----
-----Q----------QTFAADGGYEQHLADG-Q------------------TGGDGFG------Q---------Q---------------------RAGPLQDFQ--
PPPPHHLHNPG-----VPAPCLPLD----QSPNRAASFHGLP--GT---------------------ASSEPHGLDS---QRR-LPGQ-
GAAAVDALE------------YSYPN---SES---HFDLPVFS-SSE-A--EGQLP-QYGSGRQAAPPAGGNFAGHSAAL--
SRAPGS----LGKVP--S--------------------------------QQSPH--GVFFDRF--GGARKMSLGLEPG--LGSGRHHLV---Q-------
-Q----QPP-------------------------PPALLARQNSCPPALPR-PQQCPEGSAANVNL-QDSGGPILQ-AQHGQ--------
FEYPIHRLENRGLQHQP---------QHSYGPGEP---LFNV---SHHHHHSQ-------P----QQPP------
NQRLQHFDASPYANLPTRPRFD-----------------SWSGGG-GAG-MHGAAALESHL------------SPSAA-YS-------------
GLPGDFTP--PVPEN---F------PPPPGP----DPQAA-----L------Q-------------------QRQN--------TALMIKQMAATR-----G--
----QPQR-LR-PPS---LHQVGHHQHPH----PAGGHGHHQSPHQ---------------------------------HPGEAA----------------------
-----FEAQEG--AWFPP----PH---PP---------------------------------------P--GGGDLLFRP--G--IG---GLQEPPPPP-LR-
MSSGGEGHVP---SP----GGLQGQFG---------------------------LSPPSER-R--PSHPDFAVQ-------TPSFPF-GPPSRQ-
PTPHSASPGSFGPP-------------------TDFQSSAPPQS--Q-PRP---SPASSKLGALSLG--------TFPKG-----SVGAPPG-------
SAGPKESSGLFGQSCLAALSTACQNMIASLGAP--------NLNVTFGKKG---A-PAAV------GS-G-------------------------
AGGEGAKRSKLS--PAEPPETNGAG------------PQP------PLTVAAAPAG-ESS--LSPNYS------------------------------------
-------P------------GSGPDAK------AGSGRGR--GRRKRD---------
-SGHVSPVAGATGGAGGFFEKY--------------GSTAAVE--------SGSPGQG----------GERS---------------
GGGTPHLHEPPKALSSPSSAW----AKA--G--GDLL-LQPP-------PE-QAD-LLP--SLD----------QA---ESCSP--
RGGGGDFS---------REVESEEE------VSSSSDNQ-------------------------------GAKGCPGGRSPLQPP---TRPVDHPLLN--
GQKASALAQLGLHAGSST------------ATSSP----DSY--------GA---APP-GPV-----------------------------
QEEIHPLEILQAQIQLQRQQFSISEDQPLGLK------------SAKQGPEGSG----GAQNGDGGGELSSCC-----
EGAGAKGA--------VSTIDLESLMAEHSA-AWYLPG-------DK-ALLDG----EPREKA--LVSWEKAKAPTP-
GKEAHDLPSSKAL-------------APAQTGSHLQCLSVHCTDDMGDAKGRT-AVPTWRSLHSDISNRFGTFVAALT-----
----

>M.unicolor

-------MFGL-DQFEPQ--------INSRN--------VGPGER------NF-SQPGL----SMSSHFK-NPA-FH----------SG-----G-P--A-GAVDP-AIKTLNEPP--M-LS--------MNMNL-N--GEPY-GFH-VR-GHSDIH-------------AGGM------L------------PQP---------------VH----GYFGN---QQPH-HGHPTAPP-P----HPHQ-HHPHYS-GSFSGSDPSA-SCLHGGRLM----NY--NSSL---GG---Q----------QAFGE--GYD-HMAEN-Q-------------------GEGFG----------------QQ--------------------RQNDMSEF--------QHHNSTAANHAVPAPCLPLD----QSPNRAASFHGLP--AS---------------------GSSDSHGLE----QRR-IQNP-G--GVESLE------------YSYPS-DGPSG---HFDMPVFS-PSE-S--DGQLP-HYGPGRQV--PGS-SFPG-PNGL--PRTPGI--VGMPKVH--P------------------------------QQ---QH--GVFFERF--GGARKMSV-MEPG--VNA-RLPLM------------QQ-------------------------------QTGLLARQNSCPPAIPR--QQQTESSAPNPSL-QDNG-PIIQ-NQHAQ--------FEYPIHRLENRNM--------------HPY--TDA---VFNL---QQ-------------P----QQPP------NQRLQHFDA-PYMNVPKRPRFDFPSS-H----N-VDSCATWSN-----N-GMHSTGLESHL------------SPS-T-YP-------------GLPGEFTP--PVPES---F-PPGPPLQHAGP----DHQSM-------Q------------------------QRQN--------AAMMIKQMASRN------------QQQR-MR-QPS---LQQLGHHGEVN----QS-GIVHGSQVGNVSQ-P-NFERE----------------------NGGRMA-------------------NFDPPNP-HMTQEN--AWFPG----PH---PPGDML--------QRRM-GSNLPADATSHD---INL----QQ--NGSNLLFRS--G--VNGM-GMQE-P----LR-MA--GEGHVQALHSP-----GMHSQFANN----MGNLSQMQSPGGG-----VGLPNAPSDR-R---VPADFAGP---PMGGQPGFPF-AAANRQ-AAPHSNAPGVNTSP-------------------SNY--PPQSDF--QASQR----STASKLGALSLG--------SFNK----------PNS------------KES-MFGQSCLAALSTACQNMIASLGAP--------NLNVTFNKKN---Q-TEGKRKLSQTEGDMS--N-NSA-N-------------------STGAEYFP--NGPSQNSQVAG----AGNNNSKSAGQ------GGLTQPTQGE--NN--LSPNYS---I---------EA-TPSNDGK--LVS--GGGRGR---GRRKRD----------SGHVSP--------GNFFDKY------------SADSGAAVVSP-GQQGQQASVGE------------------------------HGGTPH---D-KSLTSP--SW----GKG-----NELL------------LGDQSD-LMS--SLD--SGIQSV-SKS---DSGSP-----HVDFP-DD-VST--NY-GNEDE------VSSSSDNN----------------------------MSKPNRSPLVTGSPKLQR------GDHGLLN--GQKPMGLTLLNN------------------TTSTP----DSYGL---SSTGG---AHP-GTPGLE-QVRTP--TSTSTT------------QDEIHPLEILQAQIQLQRQQFSISEDQPLGMK--------------SKKPDCTG------QNGE--NELSNCC---S---DNGKNT--------MSTIDLDTLMAEHNS-TWYMPN-------EK-ALMEG----QEDEKS--MAPWEETKPQNP-NKEVHDLSQNKTS-------------ATPQNSSHLQCLSVHCTDDIGEAKGRT-PVPTWRSLHSDISNRFGTFVAALT----------------------------------

>S.punctatus

-------MFAL-QQYESP--------PGSRGA-------PGHQQQ---------HPAGAPERSYSGAHFK-AAA-FP-------------------------------AELAEPSGTM-LA--------LNLPG-GGGGEAY-GFHAGG-GMQGQP---VH---GGFFGP---QQQQPQQQH---------------PSPHFG-GSGFGAEPGA-SCLHGGRLM----GYSGGQ---------------------QTFASEGGYE-HMAES---------------------KAESFG--------------------------------------QPPPRDF---QQQQPPPPPPGTSSHAVPAPCLPLD----QSPNRAASFH---------------------------GSSEPHSLD----SGGGQQQQRRLAGPDALE------------YSYPGSEGPSA---HFDLPVFSSSEP----EGQLP-HYGPG-----SRGGSFPS---GL--ARAPGM----GGKVH--P-----------------------------QQQPQPH--GVFFERF--GGARKMPVGLEAG--VGA-RHPLLQPQQ--------QQPPPPQ---------------------------QPGLLGRQNSCPPALPR--QPPPDASPSL----QESG-PLLP-SQHAE--------FEYPIHRLENRGL--------------NPY--AEPSAAVFSM---QPPP----------------PPPP------SQRLQHFDAPPYLTVPKRARFDFP----------GERCASWTS----------HGGVESHL------------SPS------------------AYPGDFPP--PGPESFPP--PPGPSPEHQAA---LQQQQQ--------------------------------QRQN--------AALMIKQMAASR------------SQQQQRMR-PPS---LQHLGHPGDPG----------HPGAAGF---ELAQEG--AWFPG---------AAGELL--------TRRMGGSGLPPDGAPHD------------------LLFRP-AAAGVGGL-GLQDSP----LRSLA--AEGHVQALHSP-----GMHAQFGLS-------------PGAG-----------PSERRG---PPPDFSAG-------QPGFPF-GASSRQ-ATPHSASPGSYPPP---------------PGSGGDY--PASGPA--APPRG---APASSKLGALSLG--------SFAK-------------------------QPG-LFGQSCLAALSTACQNMIASLGAP--------NLNVTFGKKSQAAP-EGAKRKLSQTEPPAAPGPELA----------------------GPGPEYFP--AKPSPGGESS--------------------------------------LSPNYS--LEATPGNDGKPVPAG---GGRGR---GRRKRD----------SGHVSP--------GSFFDKY-----------------PGAAAESGGCAGVSPAGQG---------------------------PERAPQ---DKAPLTSP--SWPPPKGGG---GPPELL---------------QPE-LLA--SLD--GGGAPKSERG---SPAAT-----AADFA-EQ-LSPGGGY-GHQEQEAEEEAVSSSSDNH-------------------------------PQGKAPPRGSPKLPL----------LLNGPKPPCLALGLGPN------------------STSTP----DGY--------GS---GPPPGTPGLE-QVRTP---GGASG------------QEEIHPLEILQAQIQLQRQQFSISEDQPLGSG---------------KKPECGG-----AVGGD--SELSSCC---SAAAESVKSA--------MSTIDLDSLMAEHSSGGWYLPGPDGGQQQQQ-QEEEE----EEEEKG--LPPWEKAKPPIP-SKEAHDLPQNKSS---------------AQTGGHLQCLSVHCTDDMGEAKGRS-AVPTWRSLHSDISNRFGTFVAALT----------------------------------

>R.bivittatum

-------MFGL-EQFEPQ--------INSRN--------VGQGER------NF-NQPGL----NMSSHFK-NPA-FH----------SG-----G-P--A-
GTVDP-AIKTLNEPP--M-LT--------MNMNL-N--GEPY-GFH-VR-GHSDIH-------------AGAM------L------------PQP------
---------VH----GFFSN---QQPH-HGHPSAHP-P----HPPQ-HHPHFS-GSFGGPDPSA-SCLHGGRLM----NY--NSNL---GS-
--Q----------QAFGE--GYD-HMAEN-Q-------------------GGEGFG----------------QQ---------------------RPSNMADF--------
QHPNSTASNHAVPAPCLPLD----QSPNRAASFHGLP--SS---------------------SSSDSHSLE----QRR-IQNQ-G--
GVESLE------------YSYPN-DGPPG---PFDMPVFS-PSE-S--DGQLPHHYGAGRQV--PGG-SFPG--TVL--PRTSGI--
VGMSKVH--P-----------------------------QQQ---QH--GVFFERF--GGARKMSVGMEPG--VNA-RHPIM--QQ--------QQ---
----------------------------QTGLLARQNSCPPAIPR--QQQTEANTPNPSL-QDNG-PIMQ-NQHAQ--------
FEYPIHRLENRNM--------------NPY--TDA---VFNM---QQ-------------P----QQPP------NQRLQHFDA-
PYMNVAKRPRFDFSSS-H----N-VDSCATWNN-----N-SMHNPGMENHL------------SPS-A-YP-------------GLPGEFTP--
PVPES---F-PAGPPLQHPGP----DHQSI-------Q------------------------QRQN--------AAMMIKQMASRN------------QQQR-
MR-QPS---LQQLGHHGDVS----QS--IVHGGQVGNGSQ-P-NFERE----------------------NGGRMA--------------------
SFEPTNP-HMAQEN--AWFAG----PP---APGNML--------QRRI-GSSLPADATSHD---MSL----QQ--NGSNLLFRA--G--
VNGM-GMQE-P----LR-MA--GEGHGQALHSP-----GMHSQFGTN----MGNLSQMQSPGGG-----VGLPTAPSDR-R---
APADFAGP---PMGGQPGFPF-AASSRQ-AAPHSNAAGVNTSP-------------------GSY--PPQADF--QASQR----
SAASKLGALSLG--------SFSK----------PNS------------KES-MFGQSCLAALSTACQNMIASLGAP--------
NLNVTFNKKN---Q-TEGKRKLSQTEGDLT--N-NAG-N-------------------NTGSEYFP--NGSSQN----G----
AGNGSSKAAGQ------GGVTQPTPGE--TS--LSPSYS--I---------EA-TPGNDGK--
LVT--GGGRGR---GRRKRD----------SGHVSP--------
GNFFDKY------------SADSGTAVVSP-GQQGQPPHVGE-----------------------------HGGTPH---D-KTLTSP--SW----
GKG-----NDLL------------LSDQSD-LMS--SLD--SGIQSV-TKS---DSSSP-----HVDFP-DD-VST--NY-GNEDE------
VSSSSDNN----------------------------TSKPNRSPLVTGSPKIQR------GEHGLVN--GQKPMGLNLLNN------------------
TTSTP----DSYGL---SSTAG---GHP-STPGME-QVRTP---TSTST------------
QDEIHPLEILQAQIQLQRQQFSISEDQPLGMK--------------NKKTECAG------QNGD--NELSSCC---S---DNVKNA------
--MSTIDLDTLMAEHNS-TWYMPN-------EK-SLMEG----QEDEKS--MAPWEETKPQNP-NKEVHDLPQNKTS---------
----AAAQTSSHLQCLSVHCTDDIGEAKGRT-PVPTWRSLHSDISNRFGTFVAALT----------------------------------

>X.tropicalis

-------MFGL-EQFEPQ--------INSRS--------AGQGER------NF-TQPGM----NMSSHFK-NAA-FH----------SA-----G-A--P-
GAVDS-AMGALNEPS--M-LG--------MNLNL-N--GEPY-GYH-AR-GQSDIH-------------PGGM------Q------------PQP---
------------VH----GFFNN--QHNPH-HGHPNS----------HQ-HHPHFS-GNFNGPDATA-SCLHGGRFM----GY--NNNI---
GN---Q----------QAFGE--GYE-QIAEN-P-------------------SGEGFV----------------QQ---------------------RSGNISEF--------
QHPNSTASNHAVPAPCLPLD----QSPNRASSFHGLP--TS---------------------STSDSHNQE----QRR-IHNQ-G--
GVEPLE------------YNYPS-DNPSG---HFEVPVFS-PSD-S--DG----HYGAGRQV--PSG-SFPG-TSVL--PRPPGL--
VGMGKVH--P------------------------------QQ---QH--GVFFERF--NNARKMSVGMDPA--VNA-RHPLL--QQ--------QQ---
----------------------------T-GLLARQNSCPPAITR--QQQTEANTSNPNL-QDNG-PVMQ-SQHAQ--------
FEYPIHRLENRNM--------------HPY--SES---VFSM---QQAP-----------P----QQPP------NQRLQHFDA-
PYMNVTKRPRFDFPNN-H----N-VDNCAAWNN-----S-NIHNAAIESHL------------SPS-T-YP-------------GLPGDYNP--
QVPES---F-PPGPALQHPGS----DHQSL-------Q------------------------QRQN--------AAMMIKQMASRN------------QQQR-
MR-QAN---IQQLGHHGDVN----QS-SIVHGGQVGGMQQ-P-NFDRE-----------------------GGRIG--------------------
TFDPQNP-HVAQEN--AWFPG----PH---PPGDIL--------QRRMGGSNLPADPSSHD---MNL----QQ--NGSNLLFRP--G--
VNRM-GIQE-P----LG-IP--GEGHVQALHSP-----GMHSQFGSN----MANLSQMQSPGGG-----VGLSSTPADR-R---
GPADFTAS---SIGGQAGFPF-VGSNRQ-STPH-NPPGVNSSP-------------------SSY--PPQSDF--QANQR----
SAASKLGALSLG--------SFSK----------SSA------------KES-MFGQSCLAALSTACQNMIASLGAP--------
NLNVTFNKKS---Q-TEGKRKLSQTETDIN--S-NSG-N-------------------NNGSDYFP--GATSQSNQVSG----
SSNANNKSTGQ------VGASQPTQGE--TS--LSPNYN--I---------EV-TPGNDGK--
PVT--AGGRGR---GRRKRD----------SGHVSP--------GNYFEKF-
-----------SADSGGAVVSP-GQQGPSANSGE-----------------------------SGGAPH---D-KPLTSP--SW----GKG-----GELL--
----------LGDQPD-LMS--SLD--SGIQSV-TKS---DSSSP-----DVDFG-ED-VNT--TY-GNEDE------VSSSSDNN------------
----------------IAKPNSCPMVTGSPKIQR------GEHGLLN--GQKSMGLNLLNN------------------TTSLP----DSYGL---
SSTGG---GHP-GTPGME-QVRTP---TSTST------------QDEIHPLEILQAQIQLQRQQFSISEDQPLGMK--------------
NKKNDCTA------QSVD--SELNSCC---S---DNVKNS--------MSTIDLDSLMAEHNS-TWYMPN-------EK-SLMEG----
DEEDKS--ITPWEKSKSQQT-NKEALDLPQNKTS-------------AAAQNGSHLQCLSVHCTDDIGETKGRT-
PVPTWRSLHSDISNRFGTFVAALT----------------------------------

>R.typus

-------MFGL-DQFSRS--------GGGPG-----------GER------NF-SQ-AAL---VMSGHYK-SPT-FP----------GA-----S-G--S-
AMDEQ-ALGPL-EPP--M-LG--------LGMNP-SLGGEPY-AFH-PRGGHSELHGGAGALQPP--
QAPAAAGSTAAPPPGGPQPPQAPPPPPPTAAAAGPPPQPQPQAAH----GYFPGGP--------HPHGHP-----------H--FS-
GSFCGTEPGH-SCLHGGRLLGT-PGYSSNPLA---GQ---P----------HAFGD--SYD-PLGEN-QTA--
GGGGGGGGGGAGGGGPGEGFA------Q---------QAAP-----------------VGRPGNLSEY--------HQHHTPSSNH---
TPCLPLD----QSPNRAASFHGLP--AS--------------------STSEAHGLE----QRR-LQNQ-P--AVDSME-----------YNYQN-
DPPTR---PFDMPVFS-PSE-S--GAQLP-HYGPGRHV--PGG-NFPA-NAPM--PRTPGL--VSLGKIH--P---------------------
--------QQ---QH--GMFYERF--GNARKMQVGMESS--VAA-RNPLM-----------QQ----------------------------
QAGLLARQNSCPPAIPR--QQQAEAGAPNSSL-QDTG-AMMP-NQHAP--------FEYPIQRLENRNM-------------HPY--
SDP---MFNV----------------------QQQP------NQRLQHFDA-PYLNVAKRPRFDFPNN-H----N-VDNCATWSS-----N-
SMHNASLENHL------------SPS-A-YP-------------GLPNEFSP--PGPEG---F-PPGPPLQHPGA----DQQSL-------Q----------
--------------QRQN--------MLMMFKQMVSRN-----------QRHR-MR-QPE---LQHLSHHADVN----QN-
SIVHSGQVANMSQ-P-SFERE----------------------SSGRMP-------------------GFDPQNP-QMGQEN--AWFSG----HH---
PSGEML--------QRRMGGSAVPPEGSPHESVQMNL----QQ--NGSGMLFRP--G--GNGL-GMQE-P----MR-MP--
GDGHVQGLHSP-----GMHSQFANN----MANVAQMQSPSGG-----MGLPNTSTDR-R---AGPDFQGT---
PMGGQPGFPF-GGPNRPS-NPHNNPPGVAPSP-------------------GTY-PPPQSEF--QANQR----SSLSKIGSLSLG--------
SFSK----------PGS------------KDNTIYGQSCLAALSTACQNMIASLGAP--------NLNVTFNKKN---Q-
NEGKRKLSQTEQDSV--GPSSGGGG--GNV-----GGPSTGSTGNGSEYFQ--GSTQQNSQMGV----
SGSVSGKLGTSTAQNTTQGPNPPSQPE--CN--LSPNYA--L---------EA-IP-SEGK--
GQT----GRGR---GRRKRD----------SGHVSP--------GNFFDKY--
---------SAENVNPGVS-P-GQQGQSSHAGD------------------------------RGGTPQ---D-KSLTSP--SW----GKG-----NDLL--
----------LPDQPD-LMS--SLD--SGIQSV-TKS---DGSSP-----QVDFS-DD-VSN--NY-GNEDE------VSSSSDNN------------
------------------IPKTTRLVTSSPKLQR------ADHSLLS--GQKPMGHGMLNAHPNSN-------------TTSNTGPGADSFGL--
----GSTGSGHP-GTPGME-QVRTP---TSTST------------QDEIHPLEILQAQIQLQRQQFSISEDQPLAMK--------------
NKKAECPG------QNGD--TELATCG---T---DTSKAA--------ISTIDIESLMAEHNS-TWYMPN-------DK-AMMDP----
QDEDKQ--MAPWEKAKSAST-NKEAHELSQNKAS--------------SVQTGSHLQCLSVHCTDDLTDSKGRS-
PMQTWRSLHSDISNRFGTFVAALT-----------------------------------

>M.mutica

-------MFGL-EQFEPQ--------MSSRN--------AGQGER------NF-SQAGLT----MSSHFK-SPA-FH----------SG-----G-P------
ADP-AISALAEPP--I-LG--------MNM---NMAGEAYG-FH-AR-GHSELH-------------AGGM------Q------------AQP--------
-------VH----GFFGN---QQPH-HGHPTTHH-P------HQ-HHPHFS-GNF-GSEPSA-SCLHGGRLM----SY---NNM---GS---
Q----------QAFAE--GYE-HMAEN-Q-------------------GGEGFG------Q---------Q---------------------RSGNMPDF--------
QHHNSSASNHAVPAPCLPLD----QSPNRAASFHALP--AS---------------------SSSDSHSLE----QRR-LPNQ-G--
GVDSLE------------YNYPS-DGPSG---HFDLPVFS-PSE-S--DGQLP-HYGAGRQV--PGGGSFPG-TSVL--PRAPGI--
AGMSKVH--P-----------------------Q----------QQH--GVFFERF--GGARKMSVGMEPG--VNA-RHPLM--QQ--------QQ---
----------------------------QTGLLARQNSCPPAIPR--QQQTEANTPNPNL-QDNG-PIMQ-NQHAQ--------
FEYPIHRLENRNM--------------HPY--TDP---VFNM----QHP---P-------P----QQQP------NQRLQHFDA-
PYMNVAKRPRFDFPSN-P----A-VDRCPSWNN-----N-LH-NGGMENHL------------SPS-A-YP-------------GLPGEFTP--
PVPES---F-APGPPLQHAGP----EHQAL-----Q--------------------------QRQN--------AALMIKQMASRN------------QQQR-
LR-QPS---LQQLGHHGDVG----PSSLVQHAGPVGNMPQ-P-GFERE----------------------SGGRGP-------------------
NFEPQAP-HLAQDS--GWFPGPH--PH---PAGELL-------PPRRMGG---PAEPGPHE---LGL----PQ--NGSGLLFRP--P--
VGGL-GL------------A--GEGHVPALHSP-----GVHAQFG-------AGLAPLQSPGGG-----VGLPSAPAER-R---
PQPDFAAP---PLGGQAGFAF-GASGRA-APPHSA----SASP-------------------GAF-PPAPPEF--PPAPR----
AAASKLGALSLG--------SFAK----------PA-------------KEN-VFGQSCLAALSTACQNMIASLGAP--------
NLHVTFAKKS---P-PEGKRKLGPPEPDG---------------------------GPAPGPDFFP--GAAAA--AAAA----------KAPPG-----
-----AP-----ETS--LSPGYA--P---------E--APGGEGK--AAAAAAGGRGR-----------------
--GRRKRD----------SGHVSP--------GGFFDKF-------------------------
PPAEGGGGGGA-----------------------------GSPGPP---D-KPLTSP--SW----AKG-----GELL------------LAEQPD-LLA--
SLD--SGIQSA-SKS---DGGSP-----RGDFP-DE-PSP--AY-GHEDE------VSSSSDGA----------------------------
LAKPTRSPLLGGSPKLPR------AEHALLG--AQKPLALGLL--------------------GAAAP----DGYGLGGGGGGGG---
AHP-GTPGLE-QVRTP---TSTSA------------QDEIHPLEILQAQIQLQRQQFSISEDQPLGMK--------------SKKAECPG---
---QNGE--GELNSCC---S---DNVKGA--------MSTIDLDTLMAEHNS-TWYMPS-------EK-SLMEG----PEEDKP--
MAPWEKSKPQNP-SKEAHDLPQNKTS-------------AAAQTGSHLQCLSVHCTDDMGESKGRT-
AVPTWRSLHSDISNRFGTFVAALT-----------------------------------

>T.guttata

-------MFGL-EQFEPQ--------NSGRS--------GGQAER------GF-GQPGLS----MSAHFK-APA-FP----------GG-----G-P----
APADP-ALGALGEPP--L-LG--------MNM---SLAGDGYG-FP-GR-GPAELH------------GGGM------Q-----------P-P------
---------VH----GFFGG---QQPH-GGPGGAPH-P------HQ-HPPHFG-GGF-GPDPGA-SCVHGGRLL----GY--SGAL---GG-
--Q----------TAFAD--GYE-HMAEG-Q-------------------GGEGFG------Q---------Q---------------------RPGTLPDF--------
QHHGAGAASHAVPAPCLPLD----QSPNRAASFHGLP--AA---------------------GSSEPHGLE----PRR-LPAQ-G--
GVDSLE------------YNYPG-DGPAS---HFELPVFS-PSE-P--EGQLP-HYGGGRQV--PAGGSFAG-APAL--PRAPGM--
A-VAKAH--P-----------------------P----------QQH--GVFFERF--GGARKMSASLEPG--ANA-RHPLM--QQ--------
QQQQPPPPPQPPQ-------------------QPPGLLARQNSCPPAIPR--QQQTEANAPNSNL-QDNG-PIMQ-NQHAQ--------
FEYPIHRLENRNM--------------HPY--TDP---VFNM----QHP---P-------P----QQPP------NQRLQHFDA-
PYVSVAKRPRFDFPNT-P----G-VERCASWGG-----G-MH-GPAMESHL------------SPT-A-YP------------GLPGEFTP--
PAPEA---F---GGPLPHGGP----EHPAL-----A-------------------------QRQN--------AALVMKQMASRS-------------QQR-
LR-PPS---LQQLGHHGEVG----AP---------GSLPP-P-AFERE----------------------AGGGR-------------------SFEAPAP-
HLAPDS--AWFAG----PP---PPGELL--------PRRLAAPGLPAEAAPHE---LGL----QP--GGPAVLFRP--G--AGGL-
GLQE-P----LR-MA--GEGPAQALPSP-----GVHPPFTPA----MGGLSQLQSPGSG-----VALPSAPAER-R---
GPADFAAQ---P-----GFPF-GAAARQ-PAAHGAAPALSASP-------------------GAY-PPPPPEF-PPPPPP---
RPAASKLGALSLG--------SFSK----------AAS------------KDN-VFGQSCLAALSTACQNMIASLGAP--------
NLNVTFNKKS---P-AEAKRKLSQAEPD----------------------------PPPAAPDYFP--AGPPVGGGGTG----------KAAGA---
-------APLLPA-ESS--LSPGYA---L---------EPVAGGGEGK------AGGGRGR--------------
---GRRKRD----------SGHVSP--------GTFFEKF----------SAAEGGGAGVS-P-
GQPAVPVAAGAP-----PGAAGAER----------------GGGTPH---D-KPLTSP--SW----GKG-----SELL------------LAEQPD-
LMS--SLD--SGIQSV-TKS---DGSSP-----HVDFP-DE-VST--SY-GNEDE------VSSSSDNA----------------------------
TSKPTRSPLLGGSPKLPR------GEHALLN--GQKPLALGLLST------------------STSTP----DSYGL---STTAG---AHP-
GTPSME-QVRTP---TSTSA------------QDEIHPLEILQAQIQLQRQQFSISEDQPLGLK--------------SKKGECAG------
QNGD--SDLGSCC---S---EGVKGT--------MSTIDLDSLMAEHNS-TWYLPG-------EK-ALMEG----QEEDKP--
MAPWEKPKPPNP-SKEAHDLPPSKTS------------AAAAQTGTHLQCLSVHCTDDVGEAKGRT-
AVPTWRSLHSDISNRFGTFVAALT-----------------------------------

>D.rerio.mn1a

---MNSNYN-SAG-FH----------MK-----G-P--S-VAVEP-
MMGPLNESP--M-QG--------LNFVS-N--RDQY-GFQ-TH-GHGDML-------------GMGV--QPQHQ---------------------------
---LHM--QGPFNH---QPPN-HDQH---------------SHLYQD---------SVPSCLHGDRHM----GF-SSTNA---GH---P----------
HMFEG--GFSQQLAEP-Q-------------------SRECISQQQ-Q-----------Q---------------------RMAAMPEF--------
QPHGHPNGNHAVPAPCLPLD----QSPNRAASFHGLP---S---------------------SSPETHRLE----HYR-LFPQ-G--
RMGGSE------------HCFPC-DPLTG---NFDMTGFSTADS-S--EHKLP-YCETGNQV--VGG-NFSTCNRSG--SRGPMM--
-GSSKVD--Q--------------------------------QLPQQ--NVFSDRF--GNRGK----MDPG--VNT-RHHLM---A--------QQ---------
----------------------RPGPVVRQNPGSPALPR-FYHTPDFVANNTDV-QGGG-PMVH-VQHGH--------
LDRPIHRLNNHNM--------------HPF--GEP---VFDV---PQLA----------------PQPP-----H--HLSSL---
PYLNMAKRPRFDLPNG------SAGESCSPLSH-----S-LHNRPSLENHL------------SPS-A-FP------------SPMGDFTS--
HVTDG---F-PSGPLPLSSGPQQQQQQQQQ-------Q-----------------------RRQN--------AAMMIKQMASRS------------
QQQR-MR-QPD---LQQLNLHGDVT----SNG-MVCRGPLGSVSQ-S-NFEKK----------------------HNFHG-------------------
-NFD--SP-HLPQEN--SWFPE----PQ------------------------QHCRETNTHA---LEQ----AE--NGHNIIFRP----------SVTS------
---------MDMQSLNSP-----GAHHPFENN----VSNPLQMQSPDES-----NMQSGAPTDR-R----PAEFGGM---
VMRRQHSFPP-GGPSQQ-GAPQSNPPGFSSSP-------------------GNY--PAHPEY--LSSQH----LSVNKLGALSLG--------
NLNK----------AST------------KDS-VFGQSCLAALSTACQNMIASLGAP--------NLNVTFNKKS---Q-
NEAKRKAGQVEQDINS---SGSS--------------------GPGAEYFQ--SNASQNSQTPC----SGNNNNTMVGQ------
SGTGQMVKRE-AST--LSPNDS---M---------ES---GCEGK--MAT--GNGKGR-----------
---GKKRRD----------SGHISP--------GNFSPPS--------------GGNPVVS-P-
GQQGSNLSMGMES----------------------------RGKTPE-----RTLVSP--SF----G-------------------------KPD-LAT--SMD--
SGIQSV-GKS---DGVSP-----CMDYL-DD-ASP--NY-STEDP--
RPCRAGVKCNSENRAS---YA-DASCME-QVRTP---LSNTG---
---------QDEVHPLEILQAQIQLQRQQFSISEDQPMGGK-------------TSKKADCQA-----SLNGE--CTLANSS---P---
VTGKSS--------VNTIDLDSLMTEQHA-TWYVPG-------NK-ALIED----PSNDKC--LGYWERARGQSD-NKEGHG------
----

>D.rerio.mn1b

-------MFEL-EQFGPQ--------INNRN--------FGHTEK------NF-NKPKV----SMNSHYK-SPG-FH----------TG-----G-S--Q-NTAEP-GMGPLNETP--V-IE--------MNINM-S-RGEQYGGFQ--R-GHSELH------------TGNL-----QQ-----------QPS---------------MH----GFFNP---QQP--HNHSHGHQ-T----HP----HQHFG-GNFG-PEPGS-SCLHSGRVM----GY--NSSM---GL---Q----------QGFTE--GFD-SLSEG-Q------------------SADG---GFS-QQ---------QQ-------------------RTSSMSDF--------QHHGPPSGNHPVPAPCLPLD----QSPNRAASFHGL-SSSS---------------------SSSESHNLE----PRR-MPPP-A--AVEGLD------------YSFPN-EPSSR---HFDVPVYS-PSE-S--DSQLS-HFGTGRQV--PGS-NFPG-NTGL--SRAPGM--QGIAKEH--P------------------------HAPPQQQQPSAQH--SVFFERF--GGGRKIPVGIEP----GA-RHPLM---Q--------QQ-------------------------------QPGLIGRQNTCPPSLPQ--PPLSETASANVGM-QEGG-VMMP-GQHNQ--------FEYPIHRPENRRI--------------HSY--GDP---IFNM---QQQP-----------PPP--QQPP------NQRLQHFDS-PYLNMGKRPRFDFPNATH----G-RESCGSWNS-----------AMENNL------------SPA-A-YP------------GLPGEFTP--PVTDG---F-STGPSLQLTGS----EQQSM-------Q-----------------------QQQN--------AAMMIKQMASRS-----------QQQR-MR-QPS---LQQLGHHNDVS----QG-PLGPGGPVGGMPQ-S-SFERE----------------------NGGRMV-------------------NFDGRSP-HMTMES--GWFPG----PH---PPGEML--------GHRM-GSS--GEMGDRD---I------QQ--NGPGMMFRA--G--VSGM-GMQE-S----MR-VP--GEGHVQPLLSP-----NIHSQFNSG----MGSLSQMQSPSAG-----VGLPNTPSER-R----SNDFPGP---PMGGPSQFQY-GGSNRQ-GASHGNSQGVNTSP-------------------GSF--TCQSDF--PTSQR----SSVSKLGGLSLG--------NFSK----------TNG------------KDN-VFGQSCLAALSTACQNMIASLGAP--------NLNVTFNKKT---Q-AEGKRKLSQTEQDLN--N-SAV-NG----------------TGNTGTEYFP-SLTAPQNGQMPP----AGNSNAKPLSQ---------NQTVQGE-ASA--LSPNFN--M---------DT-TPCSEGK--AAT--GSGRGR---GRRKRD----------SGHVSP--------GIFFPSE--------------NSNPVVS-----PSQQVSSAER----------------------------STGTPH---E-KPHTSP--SW----GKG-----GDLL------------LGDQSD-LMS--SLD--SGIQSA-SKS---EVCSP-----RMDFT-DDAVVT--HY-SNEDE------VSSSSDAQ----------------------------SSVKPGCSPLLGSPKMPN---------GLMS--GQKGQGLSNQ--------------------TTSTS----DGFG-------GA---GHP-GTPGME-QVRTP---SSTSG------------QDEIHPLEILQAQIQLQRQQFSISEDQPLAVN-----------NNKKSSELGD-----------GELASCG---P---DAEKSS--------VGTIDLDTLMAEQHA-TWYVPS-------DK-CLLED----SEEEKS----TWEKNKAQAT-IKEEADLTQNKSG------GGAGSSGTAGGMGSHLQCLSVHCTDELGESKGRGGPVPSWRSLHSDISNRFGTFVAALT-----------------------------------

>S.fasciatum

-------MFGL-DQFSRS--------GGGPG-----------GER------NF-SQ-AAL---VMTGHYK-SPT-FP----------GA-----T-G--S-AMDEQ-ALGPL-E------LG--------LGMNP-SLGGEPY-AFH-PRGGHSELHGGAGALQPP--QAPAAAGSTAAPPPGGPQPPQAPPPPPPTAAAAGPPPQPQPQAAH----GYFPGGP--------HPHGHP------------H--FS-GSFCGTEPGH-SCLHGGRLLGT-PGYSSNPLA---GQ---P----------HAFGD--SYD-PLGEN-QTAGGGGGGGGGGGGAGGGGPGEGFA------Q---------QAAP-----------------VGRPGNLSEY--------HQHHTPSSNH---TPCLPLD----QSPNRAASFHGLP--AS---------------------STSEAHGLE----QRR-LQNQ-P--AVDSME------------YNYQN-DAPTR---PFDMPVFS-PSE-S--GAQLP-HYGPGRHV--PGG-NFPA-NAPM--PRTPGL--VSLGKIH--P------------------------------QQ---QH--GMFYERF--GNARKMPVGMESS--VAA-RNPLM-----------QQ------------------------------QAGLLARQNSCPPAIPR--QQQAEAGAPNSSL-QDSG-AMMP-NQHAP--------FEYPIQRLENRNM-------------HPY--GDP---MFNI-----------------------QQQP------NQRLQHFDA-PYLNVAKRPRFDFPNN-H----N-VDNCATWSS-----N-SMHNASLENHL------------SPS-A-YP------------GLPNEFSP--PGPEG---F-PTGPPLQHPGA----DQQSL-------Q------------------------QRQN--------MLMMFKQMVSRN-----------QRHR-MR-QPE---LQHLSHHADVN----QN-SIVHSGQVANMSQ-P-SFERE---------------------SSGRMP-------------------GFDPQNP-QMGQEN--AWFSG----HH---PSGEML--------QRRMGSSTVPPEGSPHESVQMNL----QQ--NGSGMLFRP--G--GNGL-GMQE-P----MR-MP--GDGHVQSLHSP-----GMHSQFANN----MANVAQMQSPSGG-----MGLPNTSTDR-R---AAPDFQGT---PMGGQPGFPF-GGPNRPS-NPHNNPPGVAPSP-------------------GTY-PPPQSEF--QANQR----SSLSKIGSLSLG--------SFSK----------PGS------------KDNTIYGQSCLAALSTACQNMIASLGAP--------NLNVTFNKKN---Q-NEGKRKLSQTEQDSV--GPSSGGGG--GNV-----GGPSTGSTGNGSEYFQ--GSTQQNSQMGV----SGTVSGKLGTSTAQNTTQGPNPPSQPE--CN--LSPNYA---L---------EA-IP-SEGK--GQT----GRGR---GRRKRD----------SGHVSP--------GNFFDKY-----------SAENVNPGVS-P-GQQGQSSHTGD------------------------------RGGTPQ---D-KSLTSP--SW----GKG-----NDLL-------------LPDQPD-LMS--SLD--SGIQSV-TKS---DGSSP-----QVDFS-DD-VSN--NY-GNEDE------VSSSSDNN-------------------------------IPKVTQLVTSSPKLQR------ADHGLLS--GQKPMGHGMLNAHPNSN-------------TTSNTGPGADNFGL------GSTGSGHP-GTPGME-QVRTP---TSTST-----------QDEIHPLEILQAQIQLQRQQFSISEDQPLAMK-------------NKKAECPS------QNGD--TELATCG---T---DTSKAA--------ISTIDIESLMAEHNS-TWYMPN-------DK-AMMDP----QDEDKQ--MAPWEKAKSAST-NKEAHELSQNKAS--------------SVQTGSHLQCLSVHCTDDLTDSKGRS-PMQTWRSLHSDISNRFGTFVAALT----------------------------------

>B.belcheri

-------MYGQ-RPHVPVESVGENGTGGYANGA------SGWWGR---EGGGYMNVVAH----HPQHAYHHEQG-FD--------
-----------P-QTCINLKGFEFSDLDHAL----LG-------------------ED--PAMRSHAARLH--
--------------------------QLGAQSQHGHAHA------------------AQGFHQDVSY-HAQTGGIAHG------------------------------
DPTGKFPGYQRSEYHAQQ--IQGGER----------
HQLYSNGANYPHFAGMHQNVAL--KHHPRNTVYQGM---------------------------------VD----MPQSVPGQ----
FVRTQE------------NEASARTQPTD---HWQQ------------HRNFP--------------------NQGM--PSHP----------------------------
--VHAQV--QNSAADEGARWPCEQPA----
-----------------QVMQTVPHTQ---------------------------------------QGP------------------------------AEPA------N---------
PLLD---RQAWELQQR-----------------------TGSVIEREQCTAW------------SPN---FP---
ASV---EQQH--------SVLPNHLQGEEN---
VFSSQ------------------------DSLQV-------------------GYDPSLPKVEREVE-----------IP-----------------
RLQMASPPVPYQIDMSR---LEA--------KDQNFQRQI--------QI-VQNTP--------
VVEGRGYAYQQEQPVPESPSFPTRHGDQFYPGYNTQSGLSIPAAS-----VNNVEGPKDG-----LPQDLVGP---
PSQQQRLSHPA----MPMVVEHDNSVDSKAAP---------------------NFMWPSDGMS--KSEQV---LSNSSQVPTSIVN------
--NYRE--------AVPSDLPT---------EATRPRTQNVLAALSTACHNLITDLQRS------TYHLYETVGKED---T-
TQGVLEHGGMAWDSNEQVSQE-MW--------------------KAEVDEVP--GLPSVGSNDEV----HKAAPVFNQAE--------
DSSCNGAG------LSASRK--NEAVPSRKRRDSEDN----AQTK------------------
--GKKLKNKGVEMVEQKQDRNVLT-----
DTLDKENSDFNSTFPIQDGMKQTTDTATGHSLI--DTTGGLNPATLQNTDTSLHLTIVN----------------GNVYPA---
SGPNLQPN--PV----GET-----------------------------------PQV--TEVPEETTES---SKAKA---------C-EG------NLPSEEEG------
------------------------------------LRTQVVPPLYTQQRSSET--------CQSES--GFESKCVAGYSQD------------------DQSIP-----
----------QL---ENP-VFVKSDQEDRSGAGDSRQVA-----------AGEDRHPLEILQAQIRLQQQQFNLAPTETALSR--------
--------------------------PSSAPCT-------TDSKEA----------------LLED----TWYQPS-------SP----------GPDDTN--
RAPWEKNRDK---
IIKIEELEPGSVDRRLLHKLNEQRRRDSLKSAIYCLKQAIPDCRDSSDQSQQNVLNKANNFILEMLDKQRQADQCR
EEVRNLRARNLTLQQSISALQREYSHLR---------

>L.pictus

---MSGYSYGR------------GTASQFGGGGVRGQASYPTNANASAARPSSEHSYGAMAYPHIPSINE-SSE-FG------------
-----G-N--YSDPHNSYQKQSHNPS-----QR--------MN--S-NMLGGDNRNFPAPN---MDAS------------------------------------
------------------AY------------NRHSNTGMQSGY-------------------GTMSGSTPNRRASVPPNVGIGSFSNMQRSHSY------
-------------SGSSTDSAYSNRNASMTQ---SSY--------
PQPNMSNQSQQYGEGFGPYA----SHHNQPAAY---NRTNAMMK----SSEMWD-------
-----------RTSQT---RMGQAPYPNPHP-V--NMHLP-HKANSNQ---YHQTQFPS-SQTQRQSTNSQLYAANNT-----P-----
-----------------------------------GMHSSDMNMTVAGDGSV---
SSDLGIRGNQCVNPGAM----------MGQAYGNNQAMYGSSVGARMR--------VPNQRSSMSRGGM--------------
YNSMTGGN------------------------------QVPG-----SLQKLRHYGPMP--NSSSGYSRTSTRP--------
GNMQNNVGTVRNTAGSASNTMGMQGVW------------QANSG-SQ-------------SGYAQYSQNQPNVSTNVPF--
NGLPQNSNMQ----MQQPM--NYPNNMGMSNQQ------------QQQA----------
QNQRNFSGSVP----SNAGMESLSAPSGMYG-Q-STTPN-----------------------TGSAPY--------------------
PSAGCTDKVDSSS--AEFPSNISAAETEAMIQQLN--------QDRIFSAELEKLARLSR----------NP--TADDFIADP-AV--
ATPVTGTGEPTKSSCSGSNTFQSTSIDELLDGGTCVASSVSEKPSCV----ANSNVNLSHANSH-----LQSPSTSVSG-
SFQSGSGAVMNQ--NQPDQIASSSS-SAPHTPNSTSNVGKPAQNQNKGHASDKNS----------------
ESDTNQGQASESN---ENSVQHLQRMT-Q--------SIKDTSKQDDLMKVANSQHAEYLSHSS--SDN-
YGSQNCIAALSAACRNIIADMDSSMPKQNSGQKVQSPLGKNT---K-FDGSSGFGTKSDALSGFGSGPPSV------------
-------NSMGDQFV-----APNSCMM-----TAPPNCMPMDS-----YAAFPNNFNGA-NPM--AMPDFQ--------------------------
--------------------GKYHDFLEQNLPMQMAGRRIDE---KPRKG---
-RRRRKSEEFSTEQLI-SCSMPP--------VKPKRKYRKRSTQNPPSVESMEGPLSVDNV---VFTPLSESGS-------
HQADDM----------------GYRSAT---QTPNSAPN--SW----GMD---------------------VKMSCSDSI---SMA--MSIADG-
NQM---EHVSQ-------DLL-DS------VF-SPDTT------MSSTTNQE-----------------------------MPESGNCVPQGSSPIMHN----
-QSPLGHHHLNNNHPIGADHKHNMYPPQSNN----------IGSST---------------GE---ANC-QFISTSTHSVNG--
NVKQKD-------------VEEAHPLEILQAQIKIQRKQFNLGESQQQSQG---------------AKNTEKIS----------------------------
KVENAL--------PSDLEMDNLLSSEET-SWYLSE-------DQ----------PKDQGG--
GGLWEDLCPKGQTNTSSKPLLLAMQS--

>L.variegatus

---MSGYSYGR------------GTASQFGGGGVRGQASYPTNANASAARPSSEHSYGAMAYPHIPSINE-SSE-FG-----------
-----G-N--YSDPHNSYQKQSHNPS-----QR--------MN--S-NMLGGDNRNFPAPN---MDAS-----------------------------------
------------------AY------------NRHSNTGMQSGY-------------------GTMGGSTPNRRASVPPNVGIGSFSNMQRSHSY-----
-------------SGSSTDSAYSNRNASMSQ--SSY--------
PQPNMSNQSQQYGEGFGPYA----SHHNQPAAY---NRTNAMMK----SSEMWD-------
-----------RTSQT---RMGQAPYPNPHP-V--NMHLS-HKANSNQ---YHQNQFPS-SQRQ--STNSQLYAANNT-----P-------
-------------------------------GMHSSDMNMTADGSM---
SSDLGIRGNQCVNPGAI----------MGQAYGNSQAMYGSSVGARMR--------VPNQRSSMSRGGM-------------
YNSMTGGN------------------------------QVPG-----SLQKLRHYGPMP--NSSSGYSRTSTRP--------
GNMQNNVGTVRNTAGSASNTMGMQGVW------------QANSG-SQ-------------SGYAQYSQNQPNVSTNVPF--
NGLPQNSNMQ----MQQPM--NYPNNMGMSNQQ------------QQQV----------
QNQRNFSGSVP----SNAGLESLSAPSGMYG-Q-STTPN---------------------TGSAPY-------------------
PSTGCTDKVDSSS--AEFPSNISAAE--TEAMIQQLN------QDRIFSAELEKLARLSR----------NP--TSDDFIADP-AV--
ATPVSGTGEPTKSSCSGSNTFQSTSIDELLDGGTCVASSISEK--PS--CVANSNVNLSHANSH-----LQSPGTSVSG-
SFQSGSGAVASQ--NQPDQIASSSS-SAPHTPNSTSNVGKPAQNQSKGHASDKNS----------------
ESDTNQGKAGESN---ENSVQHLQRIT-Q--------SIKDTSKQDDLMKVANSQHAEYLSHSS--SDN-
YGSQNCIAALSAACRNIIADMDSSMPKQNSGQKVQSPLGKNT---K-FDGSSGFGTKSDALSGFGSGPPSV-----------
-------NSMGDQFV-----APNSCMM-----TAPPNCMPMDS-----YAAFPNNFNGA-NPM--AMPDFQ-------------------------
-------------------GKYHDFLEQNLPMQMAGRRIDE---KPRKG--
-RRRRKSEEFSTEQLI-SCSIPP--------VKPKRKYRKRSTQNPPSVESMEGPLSVDNV---VFTPLSESGS-------
HQADDM----------------GYRSAT---QTPNSAPN--SW----GMD---------------------VKMSCSDSI---SMA--MSIADG-
NQM---EHVSQ-------DLL-DS------VF-SPDTT------MSSTTNQE---------------------------MPESGNCVPHGSSPIMHN----
-QSPLGPHHLNNNHPIGADHKHNMYPPQSNN----------IGSST---------------GE---ANC-QFISTSTHSVNG--NVKQKD-
-----------VEEAHPLEILQAQIKIQRKQFNLGESQQQSQG--------------AKNNEKIS-------------------------KVENAL-------
-PSDLEMDNLLSSEET-SWYLSE-------DQ----------PKDQGGGGGGLWEDLCPKGQTNTSSKPLLLAMQS--------------
----

>A.rubens

-------MFAP-DHLQSAPHDQQHNTAVMTGNPSICG--PDFGVRSVTGAGGY--QARVAQSYHGGGMDA-SSG-FN----
------SY-----STTASTVDSMKGFECTALDSGLLNS-GG--------NN----SSGGIDGQMYITPNRTMMTTN-------------
STNM-------------------------------------TY----NEYGQ---NHSNAEGFGSQM------------TRSHAY-NNQYMSSASY-
NRQTRGVHGINNTSVHQHSLN-------------------SDYSTDSMTRRMSVPAAE---
--------------NNAATQSY------------ARANSYPTGTPYAENSLT--QFPSTSQSYSIG-------------------------GYSQTNFPE--
--LDSAVPANMA--ATAQMN-----------------RRQQVN---SYQQSYASGTVK----KTTYP-
TAAMGSGNEGWGDSSYPV-QQKF--PSHSSM--AKTHSVE-------------------------------------
SGESSSMYPQYQHGTQNTATMQGMQYT---NARLPGYT---
SSNAEMTANHYSSATNITGMCTPTSTSMTTS--NKFSKTFSGMSQQSR--------
QNMSYSQMPVGHMRTVGYKNVADS---QATAMGQS------------------------------NVAG-----SSQRLRHYGPMPT--
QAQNAQNMLYRN-------YNHSENSVNS-----GNSAVAHNVHEQY------------QHYDQ-YG---GATRHDR-
MRSLDGRFVS--SASSA-----HTGVGMPTSLSMQNTNQRPF---EPPAHQQTTTGT---
----------GHSSTGSANLSYYNNRTKPDAFSP----TSMQTAVGCQESFNHT-----YPE----------------------GQQRF----------
---------NSSVNNSNVMSHSN--CPPAYQIQQNDPVQSEADIR--------RNMIFSAELEKLARLSR----------DP--
MADEFPGSV-------GTVGVTSQPQETVQNLGQTVRNPHYPSTASG-NMGQTMYTNQGPN--
SSENSKTAVQGKSYQ-----TQAATAPPVSVPTNHSAKLTTQQ--PTVQESCAVIL-SAPSTP-
SSTAGNTQQTSPDSNSTRPVRTSTQTNQNPSAPAPAPVPSQE----KGKES---QNAVQQLQRLT-Q--------SLKE----
KKDDGTKTSQHMEYLIHNNESSSS-MGGQNCIAALSAACRNMIADMDNS--VPKLTPTTHSPIAMKS---
YMDNPGSGMGQKYVSTPQNQ-------------------------TSYGTQITDQMYSPANATGQ-----TISPPLVSMGE-----
SFGSPPYNTSN------VPQDYQ---TQFSDFLEQSVPMQMNTRRPEE---KPRKK-----
--GKRKKSDEVNDMTT--ASTIGP--------KKRRGRK--
KSSQNPLSVESLDHPNSLDNL--NVSTPMSEGQL-------PLVEES----------------PNRSAT---HTPNIMSN--SW----RTN--
D---------SK-------YT-SSE-LMP--GLL--TSQQTPSVDS---DSVAQ-------DFL-DS------VF-SPDTT-------
MSVTNQEAVDSFSQLTSVNQSHHSPASSLTSQQSMNETASTGSYSSQGGLMAR----
NNQCYQTDCIDQSKLATYSNQQMKSLKMSHG----------TVSST---------------GD---SVS-NPVSTSSDTTDA--
NSEKSA------------QEEIHPLEILQAQIQLQRQQFNLNDSRPLPLK---------------TALKKPA------------GVNPAKK-------

TGPSTM-------VQGEVDVNVLMAEEDS-TWYLPN-------EQ----------PKEPEV----PWENTRKNTK-GKDSSTVFP------
---WDWFAS--

>P.miniata

-------MFSP-GHLPPSASHEQHNSSTVMTGNPVTAGGVDYGVRSAVAGAGGYPARAAQAYHGGGGMDA-AAG-
FN----------SYGV---GAGSNTADSLKGFECSDLDNAL----LG--------SGGLH-STGGSDGQMYPVPN-RTMATN---------
----PANM------------------------------------PY----GEYGQ---GDTTSEGFGYGHV-----------SRSNAY-NNQYMSGSMY-
SRQTRGMNSMNT-TLQQHSSN-------------------SDYASENMTRRMSVPA---
-------------AESNVSSY------------TRSNSYPIGAPYENAHN---QFPPSSQSYSSMT------------------------GYNQTAFPE---
-----LDSNASSVAAGHMR------------------RTQGG---SYMQSYSSSAVK----KPSYP-TAGTGNDG--WADSGYSA-
QSKF--PSHSSMSNPKALSSL-----------------------------------DSVPVDSSSMYPQFPHRNQNSTTMQGMQYN---
NSRQTPYP---SANPEMTANQYPSVNSM-
DMYTPTSTSMNYNNKAAKTFAGGNGTAYNQ--------MAVGQSRAPGYK---------------HVTNSQQTAMGQSNM----
-------------------SASS-----QSQKLRHFGPMPN--QSQNAHHMPYRG-------YNTADTSVNN-----GQSVVTPNMDGQY--
----------QHYNQ-YSAGTTTTRHGR-LRSLDGRFVS--GASSM-----QAGMAAS-TFPVQNPNQRSF-----------------------
--------D-----------SVTHQQGVASHS-----------------FGNTNTAYYNNRTKTDAFSP----TTMQTNAGCQETYTNQ----
TYLE----------------------GQQRY--------------------NAMNCPNAIANEN--CPPAYQMPTDA---TQSEADIR------
RNMIFSAELEKLARLSR----------DP--MADDFALG------INPIDGAPQIPDTVSSLAQPVKNPPYPNATGGN--
MARGLFNNQGTN------SSDNSTGMGKN-----FQSPSASASSVTTNQSVKMTSAP--SSVTESCAAAL-SAPSTP-
SSNAGVAPQTSPDSNPTKPVRTSAPTNQSTPTPTTV--TSQD----KGKDS---QNAVQQLQRLT-Q--------SLKD-----
KKDDGVKGSHMEYLSHSSDSSSS-LGGQNCIAALSAACRNMIADMDSS--VPKLTPTSHSPVGMKA---
VMDNTGSILGQKYTPIPSNQ-------------------------TSYASQMPEQMFSPSTANGQ-----NISPPLVSIGD-----
SFGSPPYNTAN------IPTDYQ--VQFSDFLEQSVPMQMNTRRPEE---KPRKK------
---GKRKKSDEVNDMLT--ASTIGP--------KKRRGRK--
KSSQNPLSVESFDQPPSLENF--NASTPVSDGQL-------PLVEEN----------------PNRSAT---QTPNMLSN--CW----RTN--
D---------SK-------IN-SSE-SMP--GLL--SNHQPPSVDS---DSVTQ-------DFL-DS------VF-SPDTT-------
MSTTNQEPVDSFSQLTCINQSHHSPASSLTSQQSMNETASTGSYSSQGGLISR----
TNQNFQVENMEQSKLAVSQAMKASKTSNS------------TVSST---------------GD---FIS-NPVSTSSDTTDP--SSEKGA---
---------PEEVHPLKILQAQIQLQRQQFNLNDSRPLPLK---------------NASRKTA------------GVIPTKK-------TGPSSV----
----QGEVDVNVLMAEEDA-TWYMPS-------EQ----------PKEPEV----PWENTRKNAK-GKDSSTVFP----------------------
---------------------------WDWFAS---

>B.lanceolatum

-------MYGQ-RPHLPVESAEENGTGGYANGA------PGWWAR--GEGGGYMNMVAHHPQQQAAYHHE-QPG-FD----
---------------P-QTCINLKGFEFSDLDHAL----LG----------------------EDPAMRSHAARLH--------------------------------------
----------------RL------------SSQSQHGHP----------------------GQGYHQDVSYHLQTGGGVHS-------------------------------
DHTGKFPTYQRNEYQAQQ---IPGGERLQ--------
LYRNGNGANYSQFVGMHPNVAL--KHHPRNAAYQGLP---------------------------------D----MPSSIPGQ----FVRTQE---
---------NEAAARTQPAD---HWQ-------------HRNFP-NQGMAAH---PVHTQLQNNNAAM----------DSFEKIA-------------
----------------------QTEQRMRMLYAQQSAAAQQRSMLSPGQASAPSPVIQKPCA--
-AAGVWQPAPLVSRSSSI--------SSTHSNLSGTSGQCFSP---CMQ--------TPTPIPVSPCGPV---------------PQSPCQA----
---------------------------QHPP-------HRSPNLSS-LRAVQEEGARWSCEQP---------------------------TQVMQQTV--------------
---PQQVPAETPNPLLERQAWEL---------------------------------QQQR--------
TGSVVLNNVERE---------------QCTAWSPN---FSAEQQHSVLP--------------NHHLQGEENVFSSQ-----------------------
DGLQV-------------------GYDPSLPKVEREVE-----------IP-----------------RLQMASPPVPYQVDMSR---L----------
EAKDQNFQR----------QIQI------VQNTAVEGGGYPYQQEQPVPESPSFQTRHGDQFYPGFNTQSGLSIPAVS-----
VNNTEGPKEV-----LPQDLVVP----PSQEQRL---SHPVIPMVVEHAADSVDSKAA-------------------
PNFLWPSDDVSYSKSEQV---LSNSSQVPPSIVN--------NYRE-----AASDLPDA------------
EPTRPRTQNVLAALSTACHNLITNLQRS------TYNLYETVGKEE---R-AQAALEHGGMTWDGNEHIVSQDVL-------
------------KGEVDEVP--GLTSVVSNDEI----HKVPPVFNHTD----DSLSHTSCNGAG------LSTRVK-------------------------
---------------------------NEAAPTVRKRRASED---HVPTK---
GKKQKNKVMDTVEQRQNGNEMA--------DVLLDRE------NNDFHSTFATQDGITQS-
TDSTGRLNPATLQNTDTSLHLTIVN----------------GNVYPA---AGPNLQPN--PV----VET-------------------------------------
--PQVAKIQEET---TTNSS-----EAKVC-EG------NLACGGEG--
SVTQSVAPLYTQQRSLEA-------CQSESEFESKCVSGYSQEDQ------------------SSQEL--------------------ENS-
AFVKSDQGDRSAGDSRTVAG-------------EDRHPLEILQAQIRLQQQQFNLAPTETTSSR----------------------------------

PSSAPCT-------TDSKEA----------------LLDD----TWYQPS-------SP----------GPDDAN--RAPWEKNRDK---
MIKLEELEPGSVDRRLLHKLNEQRRRDSLKSASYCLKQAIPDCRDSSDQSQQNVLNKANNFILEMLDKQRQADQ
CREEVRNLRARNLALQQGISALQTEYSHLSKHP------

>A.planci.X1

-------MFSV--GHLPSSASHEQHSSTVMTGNPVGAGGVDYGVRSVAGAGGY--PSRATQAYHGGGMDA-GAG-FN--
--------SY-----G-ASNTADSLKGFECSDLDNAL----LG--------SGGFH-SNAGSDGQMYPMPN-RTMATN-------------
PANM-------------------------------------PY----GEYGH---GETNTEGFGYGHM-----------NRSHAY-NRQYMSSAVY-
GRQTRGMNSMSN-SLQQHPTN-------------------SDYSSEHMTRRMSIPAAE---
-------------SNVPTQSY------------ARSNSYPVGTPYENSHN---QYPSSSQSYSSMT------------------------GYNQTAFSD--
------LDTN----ASNMAD------------SQMRR-TQANS---Y--LQPYSSSAM-K--KPSYP-TAGTGAGSEGWSDSGYSA-
PAKF--PSHSPMSNAKALHLM------------------------------------DSAESSGMYPQFPHGNQNSTNTQGMQYT---
NTRQGTYS---SVNNEITTNHYPSVNSM-DICPPTSASMNYS--NKIAKPFAG-
GSHLR--------QGAAYSEMATGQSRAQGYKNFQDS---QASAMGQS------------------------------NVSA-----
GSQRLRHYGPMPNQNQSQGMHHLPYRA-------YGAADPSVNS-----GQSVVTPNMDGQY------------QNYDQ-FS-
GASSTRHER-LRSLDGRFVS--GAPSV-----QTGMGAS-TFPVQSQNQRSF--------------------------------E-----------
SATQQQGVSGQS------------FGST----NPA-YYNTRTKSDTFSP----TSVQTNAGCP--EMYT-N-QTYLE-------------------
----GQQRY--------------------NVMNCSSAVVNDN--CPPAYQMQSDA---AQTQADIR------RNMIFSAELEKLARLSR---
-------DP--MADDF---P-GG--LNAISGASQIQDSVSSLPQPVKNPPYLNG--TSANVAPGLFNNQGPT------
SVENSTSMGKS-----FQSPSATTTSITTNQSVKMTSAP--SSVTESCAAVS-SAPSTP-
SSNAAVAQQTSPDSNSARPIRTSPVTNPTTSTPTTV--TSQD----KGKDS---ENAVQQLQRLT-Q--------SLKE-----
KKDDGVKGSHMEYLSHSGDGSSS-IGGQNCIAALSAACRNMIADMDSS--VPKLTSTSHSPLGMKS---
VIDNPGNILGQKYTPTPSSQ-------------------------TSYGPQMPEQMFSPPGASGQ-----NISPPLVSMGD-----
SFGSPSYSTAS------VPSDYQ--M---QFSDFLEQSVPMQMNTRRPED---KPRKK-----
--GKRKKSDEVNDMLA--ASTIGP--------KKRRGRK--
KSSQNPLSVESFDQPPSLENL--SASTPLSDGQL-------PLVEEN----------------SNRSAT---QTPNMPSN--SW----RTS--
D---------TK-------LN-SSE-SVP--SLL--SNHQPPSVDS---DSATQ-------DFL-DS------VF-SPDTT-------
MSATNHEPVDSFSQLTCINQSHHSPASSLTSQQSMNETASTGSYSSQGGLTSR----
TSQNYQLDNMDQSKLAMSQAVKANRTSNS------------TVSST---------------GD---LVS-NPVSTSSDATDA--
NSEMGA------------SEEVHPLKILQAQIQLQRQQFNLNDSRPLPLK---------------NTSRKTA------------GVNPTKK------
-TGPSSV--------QGEVDVNVLMAEEDA-TWYVPS-------EQ----------PKEPEV----PWENTRKN---
AKGQQQADANPVDRRTQNKLLEQKRRDSMKNSIDNLKNALPDGRNLADQTQHSVLLKAQSFIKDMVGKQQRS
KSCLEDLKSVKSRNSMLEDNIALLKQEYDILSNME------

>A.planci.X2

-------MFSV--GHLPSSASHEQHSSTVMTGNPVGAGGVDYGVRSVAGAGGY--PSRATQAYHGGGMDA-GAG-FN--
--------SY-----G-ASNTADSLKGFECSDLDNAL----LG--------SGGFH-SNAGSDGQMYPMPN-RTMATN-------------
PANM-------------------------------------PY----GEYGH---GETNTEGFGYGHM-----------NRSHAY-NRQYMSSAVY-
GRQTRGMNSMSN-SLQQHPTN-------------------SDYSSEHMTRRMSIPAAE---
-------------SNVPTQSY------------ARSNSYPVGTPYENSHN---QYPSSSQSYSSMT------------------------GYNQTAFSD--
------LDTN----ASNMAD------------SQMRR-TQANS---Y--LQPYSSSAM-K--KPSYP-TAGTGAGSEGWSDSGYSA-
PAKF--PSHSPMSNAKALHLM------------------------------------DSAESSGMYPQFPHGNQNSTNTQGMQYT---
NTRQGTYS---SVNNEITTNHYPSVNSM-DICPPTSASMNYS--NKIAKPFAG-
GSHLR--------QGAAYSEMATGQSRAQGYKNFQDS---QASAMGQS------------------------------NVSA-----
GSQRLRHYGPMPNQNQSQGMHHLPYRA-------YGAADPSVNS-----GQSVVTPNMDGQY------------QNYDQ-FS-
GASSTRHER-LRSLDGRFVS--GAPSV-----QTGMGAS-TFPVQSQNQRSF--------------------------------E-----------
SATQQQGVSGQS------------FGST----NPA-YYNTRTKSDTFSP----TSVQTNAGCP--EMYT-N-QTYLE-------------------
----GQQRY--------------------NVMNCSSAVVNDN--CPPAYQMQSDA---AQTQADIR------RNMIFSAELEKLARLSR---
-------DP--MADDF---P-GG--LNAISGASQIQDSVSSLPQPVKNPPYLNG--TSANVAPGLFNNQGPT--
SVENSTSMGKS---------FQSPSATTTSITTNQSVKMTSAP--SSVTESCAAVS-SAPSTP-
SSNAAVAQQTSPDSNSARPIRTSPVTNPTTSTPTTV--TSQD----KGKDS---ENAVQQLQRLT-Q--------SLKE-----
KKDDGVKGSHMEYLSHSGDGSSS-IGGQNCIAALSAACRNMIADMDSS--VPKLTSTSHSPLGMKS---
VIDNPGNILGQKYTPTPSSQ-------------------------TSYGPQMPEQMFSPPGASGQ-----NISPPLVSMGD-----
SFGSPSYSTAS------VPSDYQ--M---QFSDFLEQSVPMQMNTRRPED---KPRKK-----
--GKRKKSDEVNDMLA--ASTIGP--------KKRRGRK--
KSSQNPLSVESFDQPPSLENL--SASTPLSDGQL-------PLVEEN----------------SNRSAT---QTPNMPSN--SW----RTS--

D---------TK-------LN-SSE-SVP--SLL--SNHQPPSVDS---DSATQ-------DFL-DS------VF-SPDTT-------
MSATNHEPVDSFSQLTCINQSHHSPASSLTSQQSMNETASTGSYSSQGGLTSR----
TSQNYQLDNMDQSKLAMSQAVKANRTSNS------------TVSST---------------GD---LVS-NPVSTSSDATDA--
NSEMGA------------SEEVHPLKILQAQIQLQRQQFNLNDSRPLPLK---------------NTSRKTA------------GVNPTKK------
-TGPSSV--------QGEVDVNVLMAEEDA-TWYVPS-------EQ----------PKEPEV----PWENTRKN---
AKGQQQADANPVDRRTQNKLLEQKRRDSMKNSIDNLKNALPDGRNLADQTQHSVLLKAQSFIKDMVGKQQRS
KSCLEDLKSVKSRNSMLEDNIALLKQEYDILR---------

>S.kowalevskii.X1

-------MGGS--RLQILFL-------------------------P--------------------------------------
----
GL---SISSIRIF-----------------------
CMTLP---WNWPS---------------------------------------

--RNPR------
RKRARKRDA---SLDGEEQP----------------------------
-----------------------------------KRQR---RKRKKADDTVS---------------------------------
----
--MEGENNSIES---GSI----
-------------------------IG--------SIDE-------------------------QTNKLLEEASLISESDLCGSHESNLGHH-------------------------
--EGATFFK-----------------TETTETMLPTP--

PDMQSSSPLDAVTMATISSDSP--------HMLTSPT-------SLNSPASQLPTSNEVP-SGPQHPLTPSNS------EHCSNNN---
-------------NNTVPK---NQQNSEVN--SL----CMS----------------------TEIAN-HIS--SCP--SSVNSISNDR---EDLSC-------
PVF-DG------ML-SRPDS-------SCSVEG----------------------------SHSNMNCELEDGESITPN--
SASIQNNGLCHPNTTVFMCTAGTASVTSTYS------------TSSTA-------------------
GLSAGVVCQQAPINSGQPPLNNNQ------------PEEAHPLEILQAQIQLQRQQFNISDTRPLPFK---------------
NQPKAPT------------SHKTKGT-------KTAKAA---------NQVDVETLLAEEDS-TWYMPT-------DA----------PKEPTN--
MLPWEHNKKT---
SDKGQSVDPNQVDRRTLHKISEQKRRESLKHSIDVLKRAVPDCRDLADQSQQNVLLRAHNYIVDMLGKQQRNK
MCAEEIESLKNCNNRLADEISMFRQEYEILSKLP------

>S.kowalevskii.X2

-------MGGS--RLQILFL-------------------------P--------------------------------------
----
GL---SISSIRIF-----------------------
CMTLP---WNWPS---------------------------------------

--RNPR------
RKRARKRDA---SLDGEEQP----------------------------
-----------------------------------KRQR---RKRKKADDTVS---------------------------------
----
--MEGENNSIES---GSI----
-------------------------IG--------SIDE-------------------------QTNKLLEEASLISESDLCGSHESNLGHH-------------------------
--EGATFFK-----------------TETTETMLPTP--

PDMQSSSPLDAVTMATISSDSP--------HMLTSPT-------SLNSPASQLPTSNEVP-SGPQHPLTPSNS------EHCSNNN---
-------------NNTVPK---NQQNSEVN--SL----CMS----------------------TEIAN-HIS--SCP--SSVNSISNDR---EDLSC-------
PVF-DG------ML-SRPDS-------SCSVEG----------------------------SHSNMNCELEDGESITPN--
SASIQNNGLCHPNTTVFMCTAGTASVTSTYS------------TSSTA-------------------
GLSAGVVCQQAPINSGQPPLNNNQ------------PEEAHPLEILQAQIQLQRQQFNISDTRPLPFK---------------
NQPKAPT------------SHKTKGT-------KTAKAA---------NQVDVETLLAEEDS-TWYMPT-------DA----------PKEPTN--
MLPWEHNKKT---SDKGQSVDPNQVDRRTLHKISEQKRRESLKHSIDVLKRAVPDCRDLADQSQQNVLLRVRG--
RDRIPQELQQQTCR-------------------------------------

>P.flava

-------MFTS---TNPA---------------------AGRGHHSQVGASTVDHTVPVQSYDFANQRVQ-QEG-YH---------
NNGANGIVAHSDALAENMKGFECSDLDNAL----LSGQAPPNGDFSFPFNNAQGHQVRAMHHGR-MPVDMN------
-------SGNQ------------------------------------TF----SPLTH---GRAGMDGYPYDDQ------------------
GSVQGRPRSNQPSVTDSAIGE---DFHQSTE--------------------SVFSP--GLHQNLHQGDC-------------------HVDSYG-----
----------------------------------RTRRVSD---------PCPQVTPGIQSHGAAYQRTNSFPTDNINMRTQYNNFPANRGITQN--
---------------GSNMGNNVQ----TFYPNPNQTGHSNTQNVD------TTLPGIYERSTDSQPPSISPHVHGQNLGYPHGRS--
AGNLTMNASVSKPMEYMPQGSVGHTTHGT--SQYQGMASNSTTVSN-----------------------------------
SRLHHLPPHIYQQY--GNPPPPQHSPNPAPTAQVRQYPPHMQPRL---------------------------------------
NHPQFTMGNGNPPYGSSVTFTNNNNNNQHNFQSVDCAKPMFH-GTQGN--------MNANVNMTSIGHV--------------
PNMQGLP-----------------------------RYPG-------NQTRGQPRYPAPQQGYMPNSGMPNA--------
VNKTGHVGINRGGMDFQNANPGASQRLRHFGPMLENGLAQPK---
YPPEQMQGYARHPTNAIPPNYTGMPPMGDSMGMADTRPPSSMTHPPIRTPATQTQR------------------------------
YRYNHPETYPPSSATANVIPSGQN------------QNFPESVSGNRRYSNERSARASRVS----
LAQEASFAKVLQAMAKRDYPTEQV--------------------NPTWAEVG-------------------PLQGGHPQSIPDGH--
HSLENSQEPPDLSAELGFSDDPELLEKRGDLVFSAELEKLAKLSRNPIFLN----YQ--GMTDEMAPP--
GASQPAQQGHGE------
IVMNTDQNFATGEELISNGQDIGSPNLEISENQLPSFEELVADRNPQQQHQVSKEEDVQHTTANTTTVSSTPTTTV
P-
TNNISSVPSIPTPPANDSPHSISNASIGDFQETNEHSDDTKIMPVEKSLSPAGEELSKQTEENFQTAENQNMESPNA
VKQLQRMTQ---------SLKD-----KQEEVSKSRHVQYLSHNS--DPATTSSQNCIAALSAACRNMIADLDSP-
VQRSINHQLYNVVSDNG---LHSPNYEAMPSQFPQMPGMVPSPFIQQN-----------------
PTFQGHQQPMDSLSSIGCSPVTDAGQQTPNCVSPGTFHLQYQNFLHEPVDMPVEPKQKRKPRAK--------------------
----
KRNLSQDGDDVPKKQRRKKKKTSDSGDFSALEGEVNSVESSVVGSIDEQTNKMLDDASLVVSESEHMETSRMT
SAESSATLPENSKSSIIENSSNVLSTPPDIQSSSPLDAVTLATISSESP--------
VMITSPPSLSSPRSQPIASASTPNEVPDGLIMMQQQPLHQQHQQPGTPNVESASNN----------------NNTIPN---
NKQNAETN--SF----VVS---------------------QE-TAN-NIS--SCP--GSVNSVNDKE---ERATG------KGYS-DT-
VQSRPSSQNSQDGKQAFFDLNCLPND-----------------------------IATSNNASIPQTTPSYPN---------
TSISMSSGAPTCVTPVCV------------------PTSCT--------------------SVP-PGMGNQAAMHPGYPVVNNNQ------------
PPEAHPLEILQAQIQLQRQQFNISESRPLPYK---------------NPPKVSS------------GPKSKTS-------KATKPQ---------
NPVDVEKLIAEEDS-TWYMPN-------EA----------PKEPTV----PWEQSKKN---
LEKAQNLDPNQVDRRTLHKISEQKRRESLKHSIDVLKRAVPDCRDLNDQSQQNVLLRAHNYIVDMLGKQQRSK
MCAEELDSLKNCNNRLQDEISILRQECELISKIP------

>A.japonica

-------MFSH---------------------------------AQQARGSNNYSGSFSLGPEQSGIGHNYGPDGHYG-------------------P--
MANMYDGYQSNPNATPM----KQ--------FGS---SIGNETNFLGNADSSGVTNMN---
----------NQ----AMFNA---GDGT-SGYAMQQQ-----------NFPSYPSSGYGIVGQDSASSQNRSFPMNS---------------------
--------TGINNFDQSKPVAYNSGG---IPGKTVGY-------
PMGPGTIGSRRVSAPPGIGQR----NFQQRSTSFTQEM--------------------------------------AFTGSDMNSGFTSDQHTM---
---------NSVTNRNYPSG---QYHVMQVNQSNMYV--PGELP-MVSEGNL---QQNYQQPKYSTSY--PNSTSM------------
--------------------------------------GMQGQKLNYGMPRPAVYSSGSSALTRTTSRPSI---
-------QRSFSTGRVEDSYNKMATGGHLMSASFELNRG--FMNGTEFGNMQM-----KGEPANRIPNVNM--------------
SSNLLDSMGQHVGNQGSIEQAQ----------------VNRV-----SSQRLRHYGPSP---SNHGMAFSNVQG--------
MDSTPGFPN-----ASFNPMQSTSGPL------------NSN--------------------PTNFES--
GMPNAMQSMSPPGSGPMNMDNMPMVNQPGY---MNTPSVYPDSDN---------------
--FNIPRSQRYPSKLQPDAYGS----GLPSMNGGDMMGAYDPNIQPMAMS--
KMNASNPMQFQPQI----------------SRRHTR--------TDRIFSQELEKLAKLSR----------NP--MSEPFTASP----
ITTPIDSAMNPQ----VNSFPVTPETISSPNNFP---KTSISPGFQPE---
QLPQPSQLPDNEAFVQNTQINCKNTNLKPIQNLPNTPHYSAPSTPNNQTAMDIPVSAASSTNNTTTSHTFSMTDSG
K------------------------SPSNEFIDSKVEIEDKKNAVHQLKRIT-H--------SLKD-------KQTEKGQQSEYISHGTEGNTE-
GGSQNCIAALSAACRNMIADMDST-----------MPKQKAP---NQGQGNSSLPSFETLTS----------------------------
SSTPNYSSNAIGRPPNNCGMANSTTTMPPNSFSNNQYNMNSDYLSSPPFGGT---NHYMGTDFQ-----------------------
----------------------LQYQDFLEQSLPMQMTHKRPDE---KPKRT---
---RRRKVSEDMSDQISNSSGQKRR--------RSRKKSQ---------------NPPSVDSI-GTP--QSIDTCATPAMSDHFLDDIT---
-------------NNGACN-----TPIQSM--SF-------------------G-------------------SLN--SHTPSPNCPN---

SKPSPGHDDLSGNFL-DS-DNFESVF-SPDTT-------AQNLTQDPDDV--------------------------
TNSGLLINPSPNASKPTTPNRPSSNYSS--ASLPIVSPKSGPLLNCQ------------PSNLP--------------TS---TSM-
EFIPSQVSSASEKVTQSQEA------------TNEAHPLEILQQQIQLQRQQFNLGETGSLIG----------------NDSTVVK--------
----TEKNSC--------NNSNHS--------QTDINVDALITDKNS-SWYLTN-------D----------SKKDEV--LLPWEDS------
KKAASTMPS------------------KGRSLQSLMCIS---

>B.floridae

-------MYGQ-RPHLPVELVEESGTGGFANGAA-----PGWWGR--GEGGGYMNVVAHHPQHTAPYHHE-QPG-FD-----
--------------P-QTCINLKGFEFSDLDHAL----LG---------------------EDPAMRSHAARLH---------------------------------------
---------------QL------------SAQSQHGHA---------------------VQGFHQEASYHPQMGGVVHG-------------------------------
DPTGKFPTYQRNDYHAQQ---IQGGERHQ--------
LYRNGNGADYSQFVGMHTNVAM--KHHPRNAVYQGLA---------------------------------D----MPGSVPGQ----
FARTQE------------TEASARTQPTD---HWQ-------------HRNFP-NQGMTIH---PAPAQLQN-NAGM----------DSFEKVA-
---------------------------------QTELQMRMLYAQQSAAAQQRSMLSPVQANTPSPAVQKQC---------------------------------
-------------AAGGWQPAPLVSRSSSI--------SSTHSNLSGTSGQCFSP---CMQ--------TPTPIPQSPCGPV---------------
PQSPCQA------------------------------QHPP-------HRSPNLSS-LRVVQEEGARWSCEQP--------------------------
TQVMQQTV---PQQGPADPPNPLLERQAWEL--------------------------------
QQQR--------TGSVVLNNTERE---------------QCTAWSPN-FPISTEQQHNVLP---------------NHMQGEENVFSSQ---------
-------------DGLQV-------------------GYNPSLPKVEREVE----------IP----------------RLQMASPPVPYQIDMSRL----------
---EAKDQNFQR----------QIHI------VQNTPVEGGGYTYQQEQSVPESPSFQTRHSDQFYSGFNTQSGLSLPATS-----
VSNTEVPKEV-----VHQDLVVP----LSQEQRLSH---PALPMVAENDTEGVDSKVA-------------------PNFLWPSDGVS--
KSEQV---ISNSSQVTTSIAN--------NYRD-----TASDLPDL------------ESTRPRTQNVLAALSTACHNLITNLQRS------
TYNLYETVGKEE---R-AQGTLEYGGLTWGSNEHISQD---------------------MWKGEVDEVSGLPSVGSNNEL----
HKVAPVCNHTD----DSLSQTSCNGAS------LSASLE--
NEAVPTRKRRASEDD----VPTK---
GKKLRSKGADVVKEKQDGNFTT--------DILDQEK-------GDFNASFPIQDGIKST--
DTTSSLNPVSLQNTDTSLHLTIVN----------------GNVYPA---SGPNPQPN--PV----------------------------------VVP--
QQV--AEVQEETTDSGEAKVCER-----------NM------QY-DREDS------VT-----------------------------------
PAVATLCTQMGSSETCQS-------DSGFEK--EYYLAGQD---------------------DQSIP---------------QL---ENS-
GLVKPDQEDRSAGESRPLAG-------------EDRHPLEILQAQIRLQQQQFNLAPTERASSR----------------------------------
PSSAPCT-------TDSKEA----------------LLED----TWYQPS-------SP----------GPGDTS--RAPWEKNREK---
MIKIEELEPGSVDRRLLHKLNEQRRRDSLKSAIYCLKQAIPDCRDSSDQSQQNVLNKANNFILEMLEKQRNADQC
REEVRNLRARNLALQQGISALQREYSHLSKHP------

>A.mexicanus.mn1a

--MNANYN-PSG-FH----------MK-----A-P--S-LAVEP-ALGPLSDPP-
-M-QS--------LNFMP-G--REQY-GFQ-PH-SHGDML-------------PAGLQQQQQHQ------------------------------
LHMPPPPPFNS---QQLN-PEQP---------------SHPYQD------SGPGVTSCLHGERHV----GF-SGNNSNSAGH---
QHPHHHQQQQHHMFET--EFS-QITEA-Q-------------------TRECLSHQQQQQQAQQHHHHHHQ----------------------
RLASMPEY-----MHGHLHGHPSSNHAVPAPCLPLD----QSPNRAASFHGLP---S--------------------SSPESNRLE----
HYRQLFPQ-G--RVGGSQ------------YTFPC-DPVPG---HFDMPGFSTPDS-S--DPKFS-YCETGGQM--TNN-
NFPTFNHGGGVSRA-MI---GSSKVD--Q--------------------------------QLPQQ--NLYSERF--GNRAK----LVPAENLSA-
RHHLL---T--------QQ-------------------------------R-GSLSRQNPGSPVLPR-LYHTPDFVPNSPDM-QNGG-
AVVHTAQHGQ--------IDNPIHRLNNHNM------------HPHPF--GEP---VFSV---PQLG----------------PQAP-----
HQQHLSSF---PYLNMAKRPRFDLPNG------SAGEICSPLRG-----G-LHNRPGLENHL------------SPS-G-FP-------------
APLGDFTA--QMMDG---F-PSGPLPLSCGP---HQQQPL-------P------------------------RRQN--------AAMMIKQMASRN--
----------QQQRIMR-QADLQQLQPLGHHGTVT----PSGPMVHRGALGSMSQ-MTNLTDK----------------------NHFHN--
------------------NFEQQSP-HLPHENSCSWFPD----SH-----------------------QQCRDTNLHA---MEQ----TQ--
NGHNLMFRP----------GVTA---------------SDMQPLNSP-----RQHSQFENS--NPLNSPLQVQPPGHG-----
TMPPNASANL-K----N-EFGGP---TVRQQHSFPP-GRPNQQ-GTPQSNPPAFNPSP-------------------GNY--QSHPEY--
ISNQQ----LSVNKLGALSMG--------NLNK----------ASA------------KDG-MFGQSCLAALSTACQNMIASLGAP--------
NLNVTFNKKS---Q-NEAKRKSGQVEQDANSNGGGGAC--------------------GPGAEYFQ--SNASQNSQTPC----
SGNNNNVTAGQ------SAPGQMAKRE---T--LSPNNN--SSNNNNNISIGDS---
GNEGK--C-----NGRMR---GKRRRD----------SGHISP--------
GNFSPSC-------------GNSNPVVS-P-GQQASSLGMGIES----------------------------RGRTPE-----GCLVSP--SF----VKA----
---------------------D-LAT--SMD--SGIQSV-GKS---DGVSP-----CMDYL-DD-ASP--NY-SNEDT------------------------------

--------------RTNRTGMKCNSDNIRT---GYP-DTPCME-QVRTP---LGSSG------------QDEVHPLEILQAQIQLQRQQFSLSEDQPLGGK-------------AGNKPDCQS-----GLNGD--CALAGCS---P---ESGKGS--------VNTIDLDSLMSEQHA-TWYGPG-------SKAALMDD----PGNGKC--VGFWDRARGQSD-NKEGLG--

>A.mexicanus.mn1b

-------MFGL-EQFGPQ--------INSRN--------AGHAEK------NL-SQARV----SMSSHYK-SPG-FH----------SG-----G-P--P-GTVEP-GLGPLNEPP--M-LA--------LNMNM-N-GGEQYGGFH-PR-GHSDMH-------------AGGL-QQQPQQ------------QAP---------------MH----GFFNN---QQP--HNHPHGHQ-T----HPHQ-HHPHFG-GNFGDPGPGS-SCLHGGRIM----GY--GSSM---GP---Q----------QGFTE--GFD-PLADG-Q-------------------SGDAFSQQQP-QQ---------QQ--------------------RPGSMPDF--------QHHGPSSGNHPVPAPCLPLD----QSPNRAASFHGLPSSSS--------------------SSSESHNLE----PRR-MPPQ-G--GVESLD------------YNYSN-ETPSG---HFDVSVFS-PSE-Q--DSQLP-HFGAGRQV--PGA-NFPG-NPGL--SRASGM--QGISKGH--P------------------------QAPPQQQQPPSQH--SVFFDRF--GGGRKIPVGMEP----GA-RHPLM---Q--------QQ-------------------------------QTGLMGRQNSCPPSLPR--PPQPEAGSTNASM-QEGG-VMMP-GQHNQ--------FEYPIHRLENRGM--------------HPY--GDP---MFNM---QQQP-----------PPP--QQPP------SQRLQHFDS-PYLNVAKRPRFDFPNA-H----G-GEGCGSWNS-----S-MHNAPGMENHI------------SPS-A-YP-------------GLPGEFTP--PVSDG---F-PPGPPLQHPGP----EQQSL-------Q------------------------QRQN--------AAMMIKQMASRS------------QQQR-MR-QPN---LQQLGHHGDVP----QG-PMVHGGPVGAMPQ-P-GFERD----------------------SSGRMV-------------------NFDGQNP-HMAPES--GWFPG----PH---PPGEML--------GRRLGGPG--GEAGAHD---M------QQ--NGAAMMFRP--G--VNGM-GMQE-P----MR-IP--GESHVQPLHSP-----SIHPQFGNS----MGNLTQMQSPGAG-----VGLSNAPSER-R----PNDFSGP---PMGAQPTFPF-GGSSRQ-GAPHNNGQGVSTSP-------------------GSY--TSQAEF--SAGQR----SSVSKLGALSLG--------SFNK----------TSS------------KDS-VFGQSCLAALSTACQNMIASLGAP--------NLNVTFNKKS---Q-GEGKRKLSQTEQDMN--N-SAA-NG----------------TGSAGPDFFP-GGATPQSTQMPG----AGNSNTKPTGP---------NQAVQGE-ASA--LSPSYN---M---------DA-TPCNEGK--AAT--GSGRGR---GRRKRD----------SGHVSP--------GIFFPSD--------------NGNPVVS-P-GQQVAPAAGTGER----------------------------GGGTPH---E-KPLTSP--SW----GKG-----GDLM------------LGDQAD-LMS--SLD--SGIQSV-SKS---ADCSP-----RVDFT-DD-IGP--HF-CNEDE------VSSSSDAPA---------------------------SAKAGRSPLLGGSPKLQR------VDNGLMG--GQKGQGMGLANH------------------TTSTS----EGYG-------GV---GHP-GTPGME-PVRTP---SSTSG------------QDEIHPLEILQAQIQLQRQQFSISEDQPLAIK------------NGKKGSDCSG------QSGD--GELASCS---P---DAGKGS--------VGSIDLDTLMAEQHA-TWYVPN-------DK-GLLEN----SEEDKS--LTAWEKSKGQGS-IKEDVELSQNKSG-GGGGGGGGGVTPGPGGTAPHLQCLSVHCTDELGDPKSRSGPVPSWRSLHSDISNRFGTFVAALT----------------------------------

>E.electricus.mn1a

---MNVNYN-SSV-FH----------VK-----T-P--S-VAVEP-ASGPPHDSS--M-QA--------HVFMP-G--REQY-GFQ-PH-GRGGML-------------STGP------Q------------------------------LHA--PSPFNG---QQLT-SEQP---------------VHPYQD---------GVASCLHGDRHP----GF-SGGDA---GP---Q----------HMFEA--EFG-QLSEA-Q-------------------ARDCLPLHQQ------------Q----------------------RLASGASG-----ASYQLHGHPIGNHAVPAPCLPLD----QSPNRAASFHGLP---S--------------------SSPESNSLE----HCR-LFPH-G--GTGGSQ------------YCFPC-DPLSG---HFDMGGFSNLDS-T--EPKFP-YCEAGSQM--AAG-SFPNFSHGG--SRAPMI---G-SKVN--P-------------------------------QLPQQ--NAYSERF--GNRGK----LEPG--LVA-RHQLV---A--------QQ-------------------------------RPGPMGRQNPGSPVVPR-LYHTTDFVPNSPDM-QNSS-IVT----HGQ--------LDPTMHKLSGHNM--------------RPF--GEP---VFSG---PQLG----------------PQPP-----HQRQLSPF---PYLNVAKRQRFDIPSG------PAGESYSPVGG-----G-LRNRPGLENHL------------SPS-A-FP-------------APMGDFTP--HVTDS---F-PSGPPPLSCGP---PQQQPL-------P------------------------RCPN--------AALMIKQMASRS------------QQHR-MR-QAD---LQPVNHQSDGT----LNG-MVHRGPLSQL-----NFEKR----------------------HNFHG--------------------NFEIQSP-HLPHEN--SWFPD----TH------------------------QQCREASIHT---TEQ----SQ--NGHGGIFRS----------GVTT---------------VDMQFLNSP-----GLHSQFENS----INNHLQVQPSEDG-----TMQPNTPMDR-R----PREFGEA---ALRRQRGFLP-GGSNQQ-GTPKSNPPGFGSSP-------------------GNY--PSHSEF--ASSQH----LSVNKLGALSLG--------NLNK---------GST------------KDS-VFGQSCLAALSTACQNMIASLGAP--------NLNVTFNKKN---Q-NEAKRKPGQVEQDMNS-GSGGAS--------------------GPGTEYFQ--SSASHNSQVPC----SGNNNN----N------TTVGQKAKRE-ARA--LSPNHN---T---------DS---GNEGK--ASV----XRGR--GKRRPD----------SGHXSP--------GNFSPQX---------------SSNTVVSP-GPQASALSVGAE----------------------------GQGRTS---E-GSLLSP--SF----VKA---------------------------D-LTT--SMD--SGIQSV-GKS---DGVSP-----CMDYL-DD-ASP--NY-GGEDA--RVGRSSVKCGPDG---

--RG---GFP-DTPCME-QARAP---LAGSA------------QDEVHPLEILQAQIQLQRQQFSISEDQPLGGK-------------
PGRKPDCPS-----GLNGD--CALAGCS---P---ESGKGS--------VNTIDLDSLMAEQHA-TWYGSS-------NK-ALMED----
PGNGQC--VGFWDRARGQSD-NKEGHG---

>E.electricus.mn1b

-------MFGL-EQFGPQ--------INSRN--------AGHAEK------TL-NQPRV----SMSSHYK-SPG-FH----------SG-----G-P--P-
GTVEP-GMGPLNEPP--M-LA--------LNMNM-N-GGEQYSGFH-PR-GHADMH-------------GGGLQQQPQQQ------------
QTP---------------MH----GFFNN---QQP--HNHPHSHQ-A----HPHQ-HHPHFG-GNFGDPGPGS-SCLHGGRIM----GY--
GSGM---GP---Q----------QGFTE--GFD-PLSES-Q-------------------SGDGFSQQPP-QQ---------QQ---------------------
RPGSMPDF--------QHHGPSSGNHPVPAPCLPLD----QSPNRAASFHGL--SSS---------------------SSSESHNLE----SRR-
MPPQGG--GVEGLD------------YTYPS-EPPSG---HFDVSVFS-PSE-P--DSQLP-HFGAGRQV--PGA-SFPG-NPGL--
SRAPGM--QGISKGH--P------------------------QA-PLQQQPPSQH--SVFFERF--GGGRKMPVGMEP----GA-RHPLM---
Q--------QQ-------------------------------QTGLMGRQN-CPPSLPR--PPQPEAGSANAGM-QEGA-VMMP-GQHNQ--------
FEYPIHRLENRGM--------------HPY--GDP---MFNM---QQQP-----------PPP--QQTP------SQRLQHFDS-
PYLNVAKRPRFDFPNA-H----G-GEGCGSWNS-----S-MHNPPGMENHI------------SPS-A-YP-------------GLPGEFTP--
PVTDG---F-PPGPPLQHAGP----EQQSL-------Q------------------------QRQN--------AAMMIKQMASRS------------QQQR-
MR-QPN---LQQLGHHGDVP----QG-PMVHTGPVGGMPQ-P-GFERD----------------------GGGRMV-------------------
NFDGQNP-HMAPDG--GWFSG----PH---PPGEML--------GRRMGGPG--GETGAHD---M------QQ--NGAAMMFRP--
G--VNGL-GMQE-S----MR-IP--GEGHVQPLHSP-----SIHPQFGSS----MGNLAQMQSPGAG-----VGLPNTPSER-R----
PNDFSGP---PMGAQPSFPF-GGSNRQ-GAPHNNGQGVSTSP-------------------GGF--ASQAEF--PAGQR----
PSVSKLGALSLG--------SFNK----------TSS------------KDS-VFGQSCLAALSTACQNMIASLGAP--------
NLNVTFNKKS---Q-GEGKRKLSQTEQDVN--N-STA-NG----------------TGGAGPEYFP-GGATQQSTQMTG----
PGNSNTKPAGP---------NQTVQGE-ASA--LSPSYN---M---------DA-TPCSEGK--
AAT--GSGRGR---GRRKRD----------SGHVSP--------GIFFPSD---
-----------NGNPVVS-P-GQQSAPTAGTAER----------------------------GTGTPH---E-KPLTSP--SW----GKG-----SDLV------
------LGDQAD-LMS--SLD--SGIQSV-SKS---ADCSP-----HVDFP-DD-IGP--HY-GNEDE------VSSSSDAAA--------------
-------------SAKAGRSPHLGGSPKMQR------AENGLMG--GQKGQGMGLANH------------------TTSTP----EGYG-------
GV---GHP-GTPGME-QVRTP---SSTSG------------QDEIHPLEILQAQIQLQRQQFSISEDQPLAIK------------
NGKKGSDCNG------QSGD--GELPSCS---P---DAGKGS--------MGTIDLDTLMAEQHA-TWYVPS-------DK-SLLES---
-SEEDKS--LAAWEKTKGQGT-VKEDVDISQSKGG-------
AGGVAPGPGGTGPHLQCLSVHCTDELGEPKGRGGTVPSWRSLHSDISNRFGTFVAALT--------------------------------
---
